# Phylogenomic analysis of *Paracidovorax citrulli* strains reveals the presence of two lineages in Brazil

**DOI:** 10.1590/1678-4685-GMB-2025-0046

**Published:** 2026-02-06

**Authors:** David F. Duarte, Lucas P. Lucena, Marcelo H.O. Gonçalves, Ana M. Benko-Iseppon, Flávia Aburjaile, Vasco Azevedo, Bertram Brenig, Marco A.S. Gama, Elineide B. Souza

**Affiliations:** 1 Universidade Federal Rural de Pernambuco, Departamento de Agronomia, Recife, PE, Brazil.; 2 Universidade Federal de Viçosa, Departamento de Fitopatologia, Viçosa, MG, Brazil.; 3 Universidade Federal de Pernambuco, Departamento de Genética, Recife, PE, Brazil.; 4 Universidade Federal de Minas Gerais, Escola de Veterinária, Departamento de Medicina Veterinária Preventiva, Belo Horizonte, MG, Brazil.; 5 Universidade Federal de Minas Gerais, Departamento de Genética, Ecologia e Evolução, Belo Horizonte, MG, Brazil.; 6 Georg August University, Institute of Veterinary Medicine, Department of Molecular Biology of Livestock, Göttingen, Germany.; 7 Universidade Federal Rural de Pernambuco, Departamento de Biologia, Recife, PE, Brazil.

**Keywords:** Bacterial fruit blotch, melon, pan-genome, phylogenomic

## Abstract

*Paracidovorax citrulli* is the causative agent of bacterial fruit blotch in melons and watermelons. This study used comparative genomic approaches of 17 Brazilian *P. citrulli* strains obtained from melons and watermelons to classify them into groups I and II and try to understand their genomic differences. The genomes of *P. citrulli* presented general characteristics similar to those shown for the genomes of the type strain of *P. citrulli* and reference strains of groups I and II. A phylogenomic analysis revealed two distinct groups of *P. citrulli*, in which most Brazilian *P. citrulli* strains were grouped with the strain representing group I. CRISPR-Cas analysis revealed the presence of two proteins, *Cas*3 and *Cas*10, in all Brazilian *P. citrulli* genomes. In addition, we observed the presence of two plasmids (pAMC6 and pAC53) in three Brazilian *P. citrulli* strains, all closely related to group I. The prediction of effector proteins revealed the *XopE*/*AvrPphe* protein as a differential between the strains of groups I and II. The present study will contribute to a more detailed understanding of aspects of host-pathogen interactions and will help improve the detection of strains from these groups, thus elucidating the population dynamics of Brazilian strains of *P. citrulli.*

## Introduction

Bacterial fruit blotch, caused by the Gram-negative bacterium *Paracidovorax citrulli* (formerly *Acidovorax citrulli*) (Du et al., 2023), is a disease with high destructive potential that threatens the global production of cucurbits (mainly melon and watermelon) for more than 50 years, since its first detection in watermelon in the United States in 1965 (Webb and Goth, 1965; Tian et al., 2020). In Brazil, bacterial fruit blotch was first reported of economic impact in 1997, in commercial melon fields located in the state of Rio Grande do Norte (Assis et al., 1999) and is currently distributed in eight other states (Bahia, Ceará, Minas Gerais, Pernambuco, Piauí, Rio Grande do Sul, Roraima, and São Paulo) (Assunção et al., 2019; EPPO, 2023). In addition, bacterial fruit blotch has also been reported in watermelon crops in Brazil (Macagnan et al., 2003; Halfeld-Vieira and Nechet, 2007), though it has not caused significant economic impact on production (Carvalho et al., 2013).

The disease can occur at different stages of melon crop. On cotyledonary and true leaves, symptoms start as oily spots and progress to necrotic spots (Oliveira et al., 2006; Wechter et al., 2011). Symptoms on fruits are characterized by small oily spots that expand and become brown. Internally, dry rot develops as a result of bacterial colonization of the pulp (Melo et al., 2015). Therefore, given that these symptoms make fruit commercialization infeasible (Assunção et al., 2019), the efficient management of bacterial fruit blotch in melon crops requires a set of control measures across three production stages: seeds, seedlings, fruits and adult plants (Assunção *et al*., 2019).

*Paracidovorax citrulli* strains show extensive intraspecies genetic diversity (Yang et al., 2023). This species is divided into two distinct groups based on variations in aggressiveness and host preference (Walcott et al., 2004). Group I strains have been primarily isolated from non-watermelon cucurbits and demonstrate moderate to high aggressiveness on several cucurbits, including melon and watermelon, while group II strains have been isolated from watermelon and are more aggressive on this host than other cucurbits (Yan *et al*., 2013; Yang *et al*., 2022). Strains of *P. citrulli* groups I and II can also be distinguished by their effector proteins, which play crucial roles in determining their host preferences (Eckshtain-Levi et al., 2014). Furthermore, there may be other substantial genomic differences between group I and group II strains, as the complete genomic sequence of a representative group I strain (M6) is approximately 500kbp smaller compared to that of a representative group II strain (AAC00-1) (Eckshtain-Levi *et al*., 2016; Yang *et al*., 2022).

Studies with Brazilian strains of *P. citrulli* using PFGE, MLSA of housekeeping and virulence-associated genes, rep-PCR, and pathogenicity tests on seedlings of different cucurbit species revealed the predominance of group I strains (Silva et al., 2016a, b). However, the genomic sequencing of Brazilian strains remains to be carried out. Therefore, we performed the genomic sequencing of 17 *P. citrulli* strains obtained from melon and watermelon fruit in Brazil. The main goal was to classify them within groups I and II and characterize the genomic differences between them using various methods, including average nucleotide identity (ANI) and DNA-DNA hybridization (*d*DDH) values, pan-genomics, phylogenomic, CRISPR-Cas analysis, plasmid detection, and analysis of type III and IV secretion system effector protein.

## Material and Methods

### Strains, growth conditions, and DNA extraction

Sixteen strains (CCRMAc8, CCRMAc9, CCRMAc12, CCRMAc1.12, CCRMAc1.43, CCRMAc1.45, CCRMAc1.73, CCRMAc1.78, CCRMAc1.83, CCRMAc5.1, CCRMAc5.3, CCRMAc5.28, CCRMAcMP2, CCRMAcR2, IBSBF1214, IBSBF1521) and one strain (IBSBF1627) of *P. citrulli* obtained from melon and watermelon fruits in Brazil, respectively, were used in this study. These strains belong to a collection of 40 strains and were selected based on pathological on seedlings and melon fruits, biochemical, molecular, and phylogenetic variability (Silva et al., 2016b). All these strains are deposited in the Phytobacteria Culture Collection from the Laboratory of Phytobacteriology of the Universidade Federal Rural de Pernambuco, Pernambuco, Brazil.

All strains were grown in nutrient yeast dextrose agar (NYDA, 10 g dextrose, 5 g peptone, 5 g yeast extract, 3 g meat extract, and 20 g agar, completing the volume with distilled water to 1,000 ml) and incubated for 36 hours at 29 ºC. DNA extraction from the strains was performed using the DNA MiniPrep bacterial extraction kit (Axygen Biosciences, MA), following the manufacturer’s recommendations. The DNA concentration was adjusted to 10 ng μl^−1^ using a Biodrop (BiodropTM, Cambridge, Canada).

### Whole-genome DNA sequencing, assembly, and annotation

The libraries were assembled according to the manufacturer and genome sequencing was performed on the Illumina Hiseq 2500 platform (San Diego, CA, USA), resulting in paired- end libraries with 150-bp reads. The quality of the reads was assessed using the FastQC software (Andrews, 2010). When necessary, sequencing adapters were removed from the reads and the sequences were subsequently trimmed to exclude portions with lower quality using Trimmomatic v. 0.32 (Bolger et al., 2014). The genomes were assembled using the de novo method using the SPAdes-V3.15.3 software (Bankevich et al., 2012). To evaluate the quality of the assemblies, the Quast v.5.0.2 software was used (Gurevich et al., 2013). Automatic genome annotation was performed using Rapid Annotations using Subsystems Technology (RAST) software (Overbeek et al., 2014).

### Taxogenomic and phylogenomic analysis

The genomes of the Brazilian strains of *P. citrulli* were compared with the genomes of the types strains of species of the genus *Paracidovorax* and with genomes of 22 strains of *P. citrulli*, including those representing groups I (M6) and II (AAC00-1) (Table S1), which were downloaded from the Genbank database (https://www.ncbi.nlm.nih.gov/genbank/). For general calculations of genome relatedness indices, the average nucleotide identity was calculated using Pyani v.0.2.11 (Pritchard *et al*., 2015), and DNA-DNA hybridization (*d*DDH) between genomes was calculated using the Genome-to-Genome Distance Calculator version 3.0 online service (2023), applying the recommended formula (formula 2) (Meier-Kolthoff et al., 2022). The similarity matrices obtained by average nucleotide identity and DNA-DNA hybridization were converted into a heatmap using the Morpheus platform (2023). The genomes of the strains under study were analyzed together with genomes of the type strain of the *Paracidovorax* species to obtain orthologous genes using the Roary software (Version 3.13.0) (Page et al., 2015), available on the Galaxy Europe server (2023). Gene alignment was carried out using MAFFT 7.487 (Katoh and Standley, 2013) Later, IQ-TREE (Trifinopoulos et al., 2016) and iTOL software (Letunic and Bork, 2021) were used to obtain a Maximum Likelihood (ML) phylogenomic tree.

### CRISPR-Cas, Presence of plasmids, and Type III and Type IV Secretion System Analyses

The genomes of Brazilian *P. citrilli* strains were also analyzed to find clustered regularly interspaced palindromic repeats (CRISPR) and associated proteins (*Cas*) using the online tool CRISPRone, in addition, the presence of plasmids in the genomes was analyzed using the MOB-Recon software (Robertson and Nash, 2018). The type III and IV secretion system effector proteins present in the genomes of Brazilian *P. citrilli* strains and those available in GenBank were predicted using the T3Sepp (Hui et al., 2020) and T4Sepre (Wang *et al*., 2014) software, respectively.

## Results

### Whole-genome DNA sequencing, assembly, and annotation

The analyzes performed with the 17 genomic sequences of the Brazilian strains of *P. citrulli* showed values for genome size, number of coding sequences, and N50 ranged from 4,714,945 bp (CCRMAc1.12) to 5,134,657 bp (IBSBF1627), 4,472 (CCRMAc1.12) to 5,022 (IBSBF1627), and 84,151 (IBSBF1627) to 170,687 (CCRMAc5.3), respectively (Table 1). GC content ranged from 68.9% (IBSBF1627) to 69% (CCRMAc1.12), and the RNA number varied between 49 (IBSBF1214) and 51 (CCRMAcR2 and IBSBF1627). Furthermore, the genomes exhibited different values in the number of contigs, which ranged from 65 (CCRMAc5.3) and 127 (IBSBF1627).

**Table 1-:** General characteristics and relevant information of sequenced strains of *Paracidovorax citrulli* from Brazil.

	Strains								
Parameters	CCRMAc12	CCRMAc1.12	CCRMAc1.43	CCRMAc1.45	CCRMAc1.73	CCRMAc1.78	CCRMAc1.83	CCRMAc5.1	CCRMAc5.3
Genome length (bp)	4,769,369	4,714,945	4,786,390	4,778,659	4,798,004	4,765,464	4,796,320	4,787,299	4,844,472
GC Content (%)	69	69	68.9	68.9	68.9	68.9	68.9	68.9	68.9
Number of coding sequences	4532	4472	4562	4572	4596	4542	4592	4571	4635
Largest contig	387,708	336,687	383,600	383,599	383,600	383,600	383,600	387,542	383,601
N50	148,160	133,581	146,522	170,686	133,580	131,905	161,592	161,592	170,687
Number of RNAs	50	50	50	50	50	50	50	50	50
Number of contigs	67	67	68	79	68	71	67	72	65
Genome Accession*	SAMN2481 2615	SAMN2481 2616	SAMN2481 2624	SAMN2481 2620	SAMN2481 2618	SAMN2481 2617	SAMN2481 2621	SAMN2481 2630	SAMN2481 2628
	**Strains**								
**Parameters**	**CCRMAc5.28**	**CCRMAc8**	**CCRMAc9**	**CCRMAcMP2**	**CCRMAcR2**	**IBSBF1214**	**IBSBF1521**	**IBSBF1627**	
Genome length (bp)	4,785,933	4,817,077	4,817,808	4,816,063	4,759,031	4,786,754	4,863,575	5,134,657	
GC Content (%)	68.9	68.9	68.9	69	69	68.9	68.9	68.9	
Number of coding sequences	4564	4596	4602	4602	4530	4576	4658	5022	
Largest contig	383,600	286,527	336,669	383,600	383,600	383,600	383,600	304,264	
N50	146,592	161,583	150,361	149,251	133,585	170,687	133,585	84,151	
Number of RNAs	50	50	50	50	51	49	50	51	
Number of contigs	69	69	70	67	67	70	73	127	
Genome Accession*	SAMN2481 2625	SAMN2481 2634	SAMN2481 2614	SAMN2481 2627	SAMN2481 2619	SAMN2481 2622	SAMN2481 2632	SAMN2481 2629	

*The Bioproject PRJNA796053

### Taxogenomic and phylogenomic analysis

**Figure 1:**
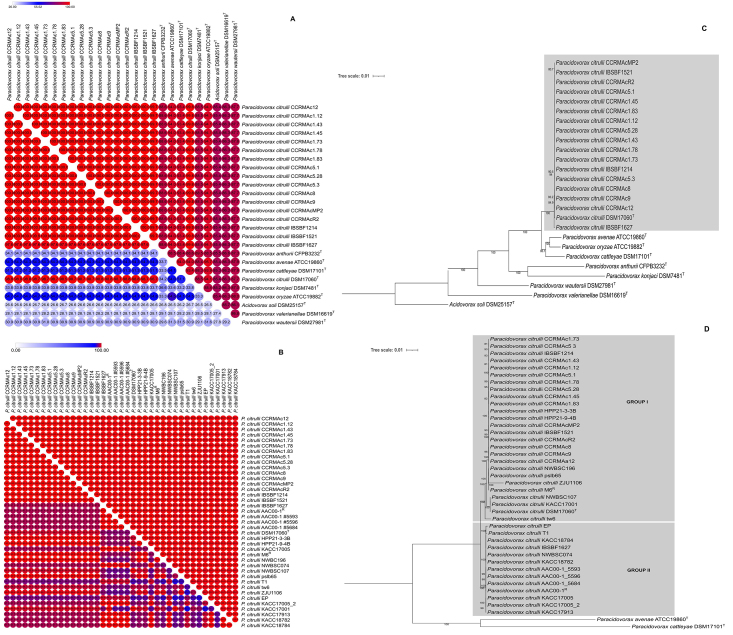
Maximum-likelihood phylogenetic tree based on core genes of sequenced strains of *P.citrulli* (CCRM) and species of genus *Paracidovorax* (A) and strains of *P. citrulli* (B). Numbers at nodes are bootstrap values (>50%) from 1000 repetitions. Bar represents the expected number of substitutions per site. Superscripts following strain names: T = type strain of a species; R = Strains Representatives of group I and group II.

The results of the ANI and *d*DDH analyzes demonstrated that values among the genomes of the Brazilian strains of *P. citrulli* ranged from 99.6% (IBSBF1627) to 99.9% (CCRMAc1.12) and 97.5% (IBSBF1627) to 100% (CCRMAc1.12), respectively (Figure 1A). ANI and *d*DDH values above 99.6% and 96.7%, respectively, were observed among the genomes of the strains sequenced in this study and the genome of the type strain of *P. citrulli* (DMS 17060^T^), showing that the analyzed strains belong to this species. Pan- genomic analysis of the genomes of *Paracidovorax* species revealed 87 core genes, 4651 shell genes, and 29251 cloud genes. The phylogenomic ML tree built with the core genome showed that all Brazilian strains were grouped into a single cluster along with DMS 17060^T^ (Figure 1C).

The ANI and *d*DDH values observed among the genomes of Brazilian strains and genomes of 22 strains of *P. citrulli* available in GenBank ranged from 99.3% (*P. citrulli* EP) to 99.9% (CCRMAc1.43) and 93.1% (*P. citrulli* EP) to 100% (CCRMAc5.1), respectively (Figure 1B). When we analyzed only the Brazilian strains, we can verify that the ANIm and *d*DDH values observed among the genomes of the Brazilian strains of *P. citrulli* varied between 99.6% (IBSBF1627) to 99.9% (CCRMAc1.12), and 97.5% (IBSBF1627) to 100% (CCRMAc1.12), respectively. When we compared the genomes of the Brazilian strains with the group I (M6) and II (AAC00-1) strains, ANIm and *d*DDH values among Brazilian strains and the M6 strain ranged from 99.6% (IBSBF1627) to 99.9% (CCRMAc12) and from 97.5% (IBSBF1627) to 99.9% (CCRMAc12), respectively. In comparison, the values among Brazilian strains and AAC00-1 strain ranged from 99.6% (CCRMAc12) to 99.9% (IBSBF1627) for ANIm and 97.4% (CCRMAc1.83) to 99.9% (IBSBF1627) for *d*DDH.

The pan-genomics conducted on the Brazilian *P. citrulli* strains, along with strains obtained from GenBank, identified 1399 core genes, 2936 shell genes, and 11795 cloud genes. The phylogenomic ML tree built with the core genes (1399) grouped 16 Brazilian strains of *P. citrulli* along with DMS 17060^T^, M6, and other eight strains (HPP21-3-3B, HPP21-9-4B, NWBSC196, pslb65, ZJU1106, NWBSC107, KACC17001, tw6), while only one Brazilian strain (IBSBF1627) was grouped along with AAC00-1 and others 12 strains obtained from GenBank (Figure 1D).

### CRISPR-Cas, Presence of plasmids, and Type III and Type IV Secretion System Analyses

**Figure 2:**
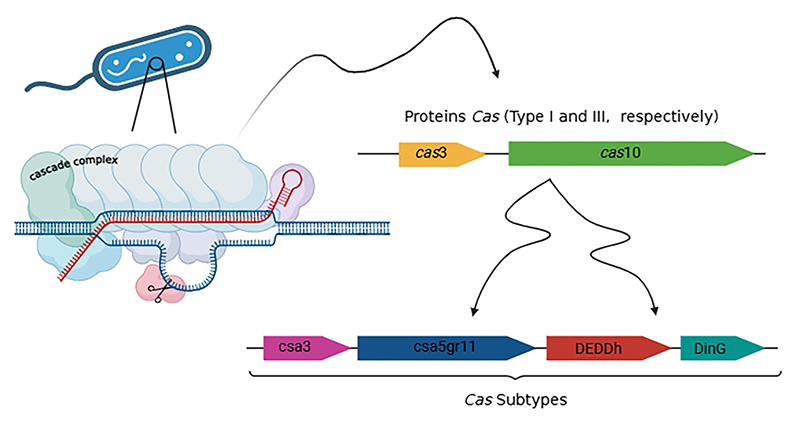
Representative diagram of *Cas* proteins found in all Brazilian genomes of *Paracidovorax citrulli* and its subtypes. Image created by BioRender.com.

Analysis of the CRISPR-Cas system revealed *Cas* proteins. All strains in the present study presented *Cas*3 and *Cas*10 proteins, which are included in class I. In addition to the proteins aforementioned, we also observed in all strains the presence of the *csa3*, *csa*5*gr*11, *deddh*, and *ding* genes (Figure 2).

**Figure 3:**
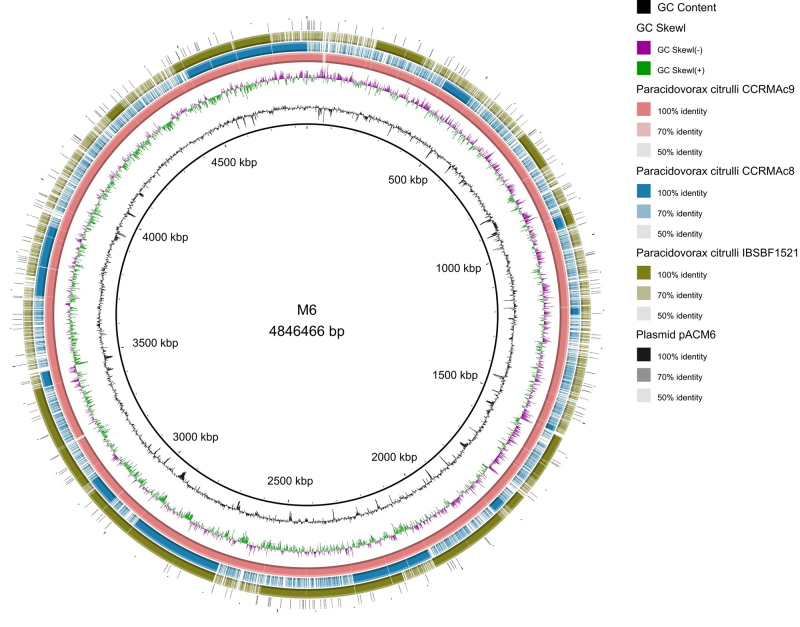
Circular visualization between the genomes of Brazilian strains of *Paracidovorax citrulli*. Graphs located in the inner center indicate GC content and GC asymmetry diagrams, in addition to the reference genome (M6). Colored rings indicate *P. citruli* strains CCRMAc9, CCRMAc8 and IBSBF1521, respectively.

The plasmids pACM6 and pAC53 were detected in the genomes of only three of the 17 strains sequenced in this study (Table 2). Plasmid pACM6 was identified in strains CCRMAc9 and IBSBF1521, while plasmid pAC53 was present in strains CCRMAc8, CCRMAc9 and IBSBF1521(strains belonging to group I). The predicted plasmids showed GC content ranging from 60.63 (CCRMAc8) to 61.25% (IBSBF1521). The visualization of pACM6 alongside the chromosomal genome revealed varying plasmid fragments across the strains examined in this study (Figure 3). 

**Table 2-:** Occurrence of plasmids in three strains of *Paracidovorax citrulli* group I.

Strain	Plasmid (code)*	Plamisd length (bp)	CG Content (%)	Description (NCBI)	Identity percentage (NCBI) (%)	Cod. Accession (NCBI)
CCRMAc8	AH419	49505	61,23	*P. citrulli* strain M6 plasmid pAC53	99,62	CP029374.1
				*P. citrulli* strain NWB SC196 plasmid pAC53	99,32	CP042322.1
CCRMAc9	AH419	48074	60,63	*P. citrulli* strain M6 plasmid pACM6	99,79	CP029374.1
				*P. citrulli* strain NWB SC196 plasmid pAC53	99,44	CP042322.1
IBSBF1521	AH419	49553	61,25	*P. citrulli* strain M6 plasmid pACM6	99,62	CP029374.1
				*P. citrulli* strain NWB SC196 plasmid pAC53	99,32	CP042322.1

*Code generated by MOB-Recon software.

The predicted number of effector proteins of the type III secretion system varied from 25 (CCRMAc1.43) to 34 (CCRMAcMP2 and IBSBF1214). In turn, the number of type IV secretion system proteins ranged from 60 (CCRMAc1.12) to 68 (IBSBF1627). The effector protein *XopE*/*AvrPphe*, which belongs to the type III secretion system effector family, was predicted in all Brazilian strains belonging to group I and was absent in the Brazilian strain belonging to group II (IBSBF1627). In addition, the description of the type III and type IV effector proteins predicted in the genomes of these strains is listed in Table S1. In turn, based on the result obtained from phylogenomic ML, we found that all Genbank strains belonging to group I present in their composition the effector protein *XopE*/*AvrPphe*, which belongs to the type III secretion system effector family; while in group II strains this protein is absent (Table S1).

## Discussion

Seventeen strains of *P. citrulli* obtained from melon (n = 16) and watermelon (n = 1) fruits with symptoms of bacterial fruit blotch in Brazil were selected to carry out a genomic characterization aiming to detect genomic differences among strains of the groups I and II to understand in more detail aspects of the pathogen-host interaction and help to improve the detection of the strains of these groups.

The ANI and *d*DDH values obtained for the 17 Brazilian strains, in comparison with the type strain of *P. citrulli* (DSM17060^T^), corroborate the parameters previously established for species determination. Thus, given that the criterion established for bacterial species determination is 70% for *d*DDH and 95% for ANI (Goris et al., 2007; Richter and Rosselló-Móra, 2009), these results confirm all strains as *P. citrulli*. In addition, the phylogenomic analysis performed here demonstrated that all Brazilian strains were closely related to DMS 17060^T^, as they were all grouped into a single clade with bootstrap 1000 (Figure 1A). Using rep-PCR, Melo et al. (2014) verified that the Brazilian strains were closely related to the type strain of *P. citrulli*, which was designated as group I, and similar results were also observed by Silva et al. (2016b). The congruence between whole-genome-based analyses (ANI, *d*DDH, and phylogenomics) and earlier molecular approaches strengthens the evidence that the Brazilian population of *P. citrulli* represents a homogeneous taxonomic group.

The values found in our study closely aligned with those observed for the genomes of the type strain (DSM 17060^T^) and reference strains of groups I (M6) and II (AAC00-1) of *P. citrulli* available in GenBank, which had genome sizes of 4,848,266 bp, 4,899,546 bp, and 5,352,772 bp, respectively, and a coding sequence number of 4,678, 4,644, and 5,174. Evolutionary evidence that separates strains of both groups lies in the presence of eight DNA fragments found in the genome of the strain AAC00-1 (group II reference strain), which are absent in the M6 genome (group I reference strain) (Eckshtain-Levi et al., 2016). In the present study, we found that the genome of strain IBSBF1627 is approximately 500 kb larger than those of the other Brazilian strains (Table 1). Furthermore, it was the only strain grouped along with AAC00-1 (group II). The ANI and *d*DDH analyses did not present borderline values that allowed the separation of *P. citrulli* groups. However, there was a trend regarding ANI and *d*DDH values; strains within the same group have higher ANI and *d*DDH values, while strains from different groups have lower ANI and *d*DDH values.

The phylogenetic ML tree constructed with the core genes (cMLSA) divided the strains of *P. citrulli* into two distinct groups, in which the majority of them, including 16 Brazilian strains, clustered together with the type strain DMS 17060^T^, as well as M6 and others strains (HPP21-3-3B, HPP21-9-4B, NWBSC196, NWBSC107, pslb65, ZJU1106, KACC17001, tw6), with a bootstrap of 1000% (Figure 1B), demonstrating that all of them belong to Group I sensu Walcott et al. (2000; 2004) and confirming the predominance of *P. citrulli* group I strains in Brazil, as previously described (Silva et al., 2016a, b). According to Zlatković et al. (2022), the strains ZJU1106 and pslb65 (also present in our study) clustered with the reference strain of group I (M6) and the type strain, thus corroborating the results of the present study. The strain IBSBF1627 was grouped along with AAC00-1 (group II), EP, T1, KACC18784, NWBSC074, KACC18782, AAC00-1 #5593, AAC00-1 #5596, AAC00-1 #5684, KACC17005, KACC17005_2 and KACC17913. Based on the analysis of the partial 23S rDNA sequence, strain IBSBF1627 was classified belonging to as group I (Silva *et al*., 2016b). However, in the analysis carried out, strains representative of the two groups were not used, resulting in a discrepancy in the results obtained. Through phylogenomic analysis based on core genes, our study demonstrated greater resolution and robustness compared to single-locus approaches, thus supporting the reclassification of isolate IBSBF1627 as belonging to group II. This result highlights the importance of using robust methods, based on complete genomes, for the correct determination of bacterial groups, since misclassifications resulting from partial gene analyses can lead to erroneous interpretations of the pathogen’s population structure.

Currently, several CRISPR-Cas mechanisms are found in prokaryotes, which are categorized into two large classes, six types and around 33 subtypes based on the nature of the effector complex and *Cas* enzymes involved (Furtado et al., 2021). Here, we found that all Brazilian strains of *P. citrulli* have *Cas*3 and *Cas*10 proteins, which are included in class I. The presence of these proteins in the *P. citrulli* genomes is essential for the bacterial response. In this case, the CRISPR-based bacterial response can be divided into three important phases: adaptation, expression and interference, therefore, the action of *Cas* proteins occurs in the first phase (adaptation), where the invading organism is detected and processed by a complete set of *Cas* proteins (Deveau et al., 2008, Makarova et al., 2020) (Figure 2).

The presence of plasmids pACM6 and pAC53 was only verified in the CCRMAc8, CCRMAc9, and IBSBF1521 strains, which show greater similarity to group I (reference M6 strain), whereas these plasmids were not found in the Brazilian strain of group II (IBSBF1627). Yang et al. (2019) observed the absence of plasmids in the AAC00-1 strain (group II), while in the M6 strain (group I) it was present. Furthermore, the authors identified the overlap of the pACM6 plasmid in eight strains group II through BlastN, indicating that these strains did not have the plasmid, and that in some group I lineages, plasmids are absent, which further reinforces the results obtained in the present study. The presence of plasmids offers several advantages in plant-pathogen interactions, considering that the plasmid is composed of several accretive genes, such as antibiotic resistance, heavy metal tolerance, toxin production, and ecological fitness of the pathogen to survive adverse conditions, in addition to virulence genes that contribute to bacterial pathogenicity (Smalla et al., 2015). In *P. citrulli*, certain plasmids, such as pAMC6, may contain in their composition genes responsible for plasmid replication, maintenance and transfer, such as the *stbB*, *repA*, *parA* and *parB, traX* and *mobC* genes, and all the genes necessary for the synthesis of a functional type IV secretion system (Yang *et al*., 2019). Plasmids also play an important role in bacterial evolution by transferring beneficial traits within and between bacterial species, positively contributing to host fitness (Rodríguez-Beltrán et al., 2021).

The presence of proteins essential for the type III secretion system was predicted in our study. The mechanisms by which plant pathogenic bacteria regulate the type III secretion system are crucial for the infection process (Tampakaki *et al*., 2010) and these mechanisms have been well reported in some plant bacteria. For example, *HrpG* and *HrpX* regulate the type III secretion system in bacteria of the genus *Xanthomonas* spp. (Xue et al., 2014) and *HrpB* in *Ralstonia solanacearum* (Jiménez-Guerrero et al., 2019). *hrpX* and *hrpB* encode AraC-type transcriptional activators that directly intercede in the expression of several *hrp*/*hrc* operations, in addition to many genes of the type III transmission system. Orthologous genes of the *hrpG* and *hrpX*/*hrpB* groups found in the *P. citrulli* Aac5 strain belonging to group II are critical for its pathogenicity, according to Zhang et al. (2018). The authors also report that *HrpG* triggers the expression of *hrpX*, which consequently regulates the expression of a T3E gene belonging to the *YopJ* family. Among the predicted effector proteins in our study, we found that all strains had a type IV pilin protein (Table S1). 

Among the predicted effector proteins in our study, we identified in all strains the presence of a type IV pilin protein (Table S1). Type IV pili are associated with the virulence of *P. citrulli*, since these bacterial surface structures contribute to adhesion to the host, colonization, aggregation, biofilm formation, and the uptake of genetic material (Craig et al., 2004; Nudleman and Kaiser, 2004). Previous studies demonstrated that suppression of the *pilA* gene resulted in the complete loss of motility in strain pslb65 (group I). However, in group II strains such as Aac5, mutation of *pilA* led only to partial loss of *twitching* motility (Yang et al., 2023), suggesting functional differences between groups. It is also important to emphasize that groups I and II differ in their T3SS effector repertoire (Eckshtain-Levi et al., 2014). In this study, we detected the effector protein of the *XopE*/*AvrPphe* family, which was present in all group I strains and absent in group II strains, both in Brazilian *P. citrulli* strains and in sequences available in GenBank. This finding reinforces the existence of specific variations between groups, which may be directly related to the adaptation and virulence of different lineages.

The results obtained from the characterization of the genomic diversity of the Brazilian *P. citrulli* population will contribute significantly to understanding the ecological, taxonomic, evolutionary, pathogenicity, and virulence aspects of the strains studied, which will be beneficial for the development of tools that will enable accurate diagnosis and the monitoring of emerging strains. Identifying strains carrying plasmids is of paramount importance, since plasmids can carry genes responsible for the bacterium’s adaptation and virulence, thus increasing its suitability in agricultural environments. Moreover, the identification of a group II strain underscores the relevance of ongoing surveillance in agricultural systems, as this group poses a threat to watermelons and other cucurbits, ultimately compromising crop performance and reducing productivity.

## Conclusion

We confirmed the predominance of two phylogenomic lineages, with predominance of *P. citrulli* group I strains in Brazil and verified that all strains had *Cas* genes in their composition. The study revealed for the first time the presence of plasmids pACM6 and pAc53 in Brazilian strains of *P. citrulli*, in which they varied between 49553 bp and 48074 bp. Furthermore, through the prediction of effector proteins we can also differentiate group I and II strains of Brazilian *P. citrulli*, verifying the presence of the *XopE*/*AvrPphe* protein. The present study will contribute to the understanding of the population diversity of Brazilian strains of *P. citrulli* and will assist in the development of techniques for managing bacterial fruit blotch, mainly in melon crops in Northeastern Brazil.

## Supplementary material

The following online material is available for this article: 

Table S1 - Predicted proteins for type III (T3S) and IV (T4S) secretion systems of Brazilian strains of *Paracidovorax citrulli* and strains available in GenBank.

## Data Availability 

The genomic sequence data generated in this study are available in GenBank, and the accession numbers are in Table 1.

## References

[B1] Assis SMP, Mariano RLR, Silva-Hanlind DMW, Duarte V (1999). Mancha aquosa do melão causada por Acidovorax avenae subsp.
citrulli no estado do Rio Grande do Norte. Fitopatol Bras.

[B2] Assunção EF, Conceição CS, Mariano RLR, Souza EB (2019). Situação atual da mancha aquosa, importante bacteriose em
meloeiro e melancieira. Anais Acad Pernamb Ciênc Agron.

[B3] Bankevich A, Nurk S, Antipov D, Gurevich AA, Dvorkin M, Kulikov AS., Lesin VM, Nikolenko SI, Pham S, Prjibelski AD (2012). SPAdes: A new genome assembly algorithm and its applications to
single-cell sequencing. J Comput Biol.

[B4] Bolger AM, Lohse M, Usadel B (2014). Trimmomatic: A flexible trimmer for Illumina sequence
data. Bioinformatics.

[B5] Carvalho FC, Santos LA, Dias RC, Mariano RL, Souza EB (2013). Selection of watermelon genotypes for resistance to bacterial
fruit blotch. Euphytica.

[B6] Craig L, Pique ME, Tainer JA (2004). Type IV pilus structure and bacterial
pathogenicity. Nat Rev Microbiol.

[B7] Deveau H, Barrangou R, Garneau JE, Labonté J, Fremaux C, Boyaval Patrick, Romero DA, Horvath P, Moineau S (2008). Phage response to CRISPR-encoded resistance in Streptococcus
thermophilus. J bacteriol.

[B8] Du J, Liu Y, Zhu H (2023). Genome-based analyses of the genus Acidovorax: proposal of the
two novel genera Paracidovorax gen. nov., Paenacidovorax gen. nov. and the
reclassification of Acidovorax antarcticus as Comamonas antarctica comb.
nov. and emended description of the genus Acidovorax. Arch Microbiol.

[B9] Eckshtain-Levi N, Munitz T, Zivanovic M, Traore SM, Sproer C, Zhao B, Welbaum G, Walcott R, Sikorski J, Burdman S (2014). Comparative analysis of Type III secreted effector genes reflects
divergence of Acidovorax citrulli strains into three distinct
lineages. Phytopathology.

[B10] Eckshtain-Levi N, Shkedy D, Gershovits M, Silva GM, Tamir-Ariel D, Walcott R, Pupko T, Burdman S (2016). Insights from the genome sequence of Acidovorax citrulli M6, a
group I strain of the causal agent of bacterial fruit blotch of
cucurbits. Front Microbiol.

[B11] Furtado LL, Rego-Machado CM, Marin-Ramirez G, Freitas NC, Molinari HBC, Santiago TR (2021). CRISPR/Cas: do sistema imune de procariotos ao controle de
doenças de plantas. Rev Anu de Patol Plantas.

[B12] Goris J, Konstantinidis KT, Klappenbach JA, Coenye T, Vandamme P, Tiedje JM (2007). DNA-DNA hybridization values and their relationship to
whole-genome sequence similarities. Int J Syst Evol Microbiol.

[B13] Gurevich A, Saveliev V, Vyahhi N, Tesler G (2013). QUAST: Quality assessment tool for genome
assemblies. Bioinformatics.

[B14] Halfeld-Vieira BA, Nechet KL (2007). Mancha-aquosa da melancia em Roraima. Fitopatol Bras.

[B15] Hui X, Chen Z, Lin M, Zhang J, Hu Y, Zeng Y, Cheng X, Ou-Yang L, Sun MA, White AP, Wang P (2020). T3SEpp: An integrated prediction pipeline for bacterial Type III
secreted effectors. mSystems.

[B16] Jiménez‐Guerrero I, Pérez‐Montaño F, Da Silva GM, Wagner N, Shkedy D, Zhao M, Burdman S (2019). Show me your secret (ed) weapons: A multifaceted approach reveals
novel Type III-secreted effectors of a plant pathogenic
bacterium. bioRxiv.

[B17] Katoh K, Standley DM (2013). MAFFT multiple sequence alignment software version 7:
Improvements in performance and usability. Mol Biol Evol.

[B18] Letunic I, Bork P (2021). Interactive Tree of Life (iTOL) v5: An online tool for
phylogenetic tree display and annotation. Nucleic Acids Res.

[B19] Macagnan D, Romeiro RS, Hl Mendonça, Barreto RW (2003). Mancha bacteriana da melancia: uma nova doença no estado de Minas
Gerais. Summa Phytopathol.

[B20] Makarova KS, Wolf YI, Iranzo J, Shmakov AS, Alkhnbashi OS, Brouns SJJ, Charpentier E, Cheng D, Haft DH, Horvath P (2020). Evolutionary classification of CRISPR-Cas systems: A burst of
class 2 and derived variants. Nat Rev Microbiol.

[B21] Meier-Kolthoff JP, Carbasse JS, Peinado-Olarte RL, Göker M (2022). TYGS and LPSN: A database tandem for fast and reliable
genome-based classification and nomenclature of prokaryotes. Nucleic Acids Res.

[B22] Melo AL, Tabaldi ND, Mehta A, Marques ASA (2014). Comparing Acidovorax citrulli strains from melon and watermelon:
Phenotypic characteristics, pathogenicity and genetic
diversity. Trop Plant Pathol.

[B23] Melo EA, Mariano RLR, Laranjeira D, Santos LA, Gusmão LO, Souza EB (2015). Efficacy of yeast in the biocontrol of bacterial fruit blotch in
melon plants. Trop Plant Pathol.

[B24] Nudleman E, Kaiser D (2004). Pulling together with Type IV pili. J Mol Microbiol Biotechnol.

[B25] Oliveira A, Santos MHM, Silveira EB, Gomes AMA, Mariano RLR (2006). Biocontrole da mancha aquosa do meloeiro pelo tratamento de
sementes com bactérias epifíticas e endofíticas. Hortic Bras.

[B26] Overbeek R, Olson R, Pusch GD, Olsen GJ, Davis JJ, Disz T, Edwards RA, Gerdes S, Parrello B, Shukla M (2014). The SEED and the rapid annotation of microbial genomes using
subsystems technology (RAST). Nucleic Acids Res.

[B27] Page AJ, Cummins CA, Hunt M, Wong VK, Reuter S, Holden MTG, Fookes M, Falush D, Keane JA, Parkhill J (2015). Roary: Rapid large-scale prokaryote pan genome
analysis. Bioinformatics.

[B28] Pritchard L, Glover RH, Humphris S, Elphinstone JG, Toth IK (2015). Genomics and taxonomy in diagnostics for food security:
Soft-rotting enterobacterial plant pathogens. Anal Methods.

[B29] Richter M, Rosselló-Móra R (2009). Shifting the genomic gold standard for the prokaryotic species
definition. Proc Natl Acad Sci U S A.

[B30] Robertson J, Nash JHE (2018). MOB-suite: Software tools for clustering, reconstruction and
typing of plasmids from draft assemblies. Microb Genom.

[B31] Rodríguez-Beltrán J, DelaFuente J, León-Sampedro R, MacLean RC, San Millan A (2021). Beyond horizontal gene transfer: The role of plasmids in
bacterial evolution. Nat Rev Microbiol.

[B32] Silva GM, Souza RM, Yan L, Sales R, Medeiros FHV, Walcott RR (2016). Strains of the group I lineage of Acidovorax citrulli, the causal
agent of bacterial fruit blotch of cucurbitaceous crops, are predominant in
Brazil. Phytopathology.

[B33] Silva KMM, Xavier AS, Gama MAS, Lima NB, Lyra MDCCP, Mariano RLR, Souza EB (2016). Polyphasic analysis of Acidovorax citrulli strains from
northeastern Brazil. Sci Agric.

[B34] Smalla K, Jechalke S, Top EM (2015). Plasmid detection, characterization, and ecology. Microbiol Spectr.

[B35] Tampakaki AP, Skandalis N, Gazi AD, Bastaki MN, Sarris PF, Charova SN, Kokkinidis M, Panopoulos NJ (2010). Playing the “harp”: Evolution of our understanding of hrp/hrc
genes. Annu Rev Phytopathol.

[B36] Tian Y, Zhao Y, Zhou J, Sun T, Luo X, Kurowski C, Walcott RR (2020). Prevalence of Acidovorax citrulli in commercial cucurbit seedlots
during 2010-2018 in China. Plant Dis.

[B37] Trifinopoulos J, Nguyen LT, Von Haeseler A, Minh BQ (2016). W-IQ-TREE: A fast online phylogenetic tool for maximum likelihood
analysis. Nucleic Acids Res.

[B38] Walcott RR, DB Langston, FH Sanders, Gitaitis RD (2000). Investigating intraspecific variation of Acidovorax avenae subsp.
citrulli using DNA fingerprinting and whole cell fatty acid
analysis. Phytopathol.

[B39] Walcott RR, Fessehaie A, Castro AC (2004). Differences in pathogenicity between two genetically distinct
groups of Acidovorax avenae subsp. citrulli on cucurbit
hosts. J Phytopathol.

[B40] Wang Y, Wei X, Bao H, Liu SL (2014). Prediction of bacterial Type IV secreted effectors by C-terminal
features. BMC Genomics.

[B41] Webb RE, Goth RW (1965). A seedborne bacterium isolated from watermelon. Plant Dis Rep.

[B42] Wechter WP, Levi A, Ling KS, Kousik C, Block CC (2011). Identification of resistance to Acidovorax avenae subsp. citrulli
among melon (Cucumis spp.) plant introductions. Hort Science.

[B43] Xue X, Zou L, Ma W, Liu Z, Chen G (2014). Identification of 17 HrpX-regulated proteins including two novel
Type III effectors, XOC_3956 and XOC_1550, in Xanthomonas oryzae pv.
oryzicola. PloS One.

[B44] Yan S, Yang Y, Wang T, Zhao T, Schaad NW (2013). Genetic diversity analysis of Acidovorax citrulli in
China. Eur J Plant Pathol.

[B45] Yang R, Garcia DS, Montaño PF, Silva MG, Zhao M, Guerrero JI, Burdman S (2019). Complete assembly of the genome of an Acidovorax citrulli strain
reveals a naturally occurring plasmid in this species. Front Microbiol.

[B46] Yang Y, Fei N, Ji N, Qiao P, Yang L, Liu D, Guan W, Zhao T (2023). pilA gene contributes to virulence, motility, biofilm formation,
and interspecific competition of bacteria in Acidovorax
citrulli. Microorganisms.

[B47] Yang Y, Qiao P, Wang T, Ji W, Fei N, Zhang L, Guan W, Zhao T (2022). Further characterization of host preference of Acidovorax
citrulli based on growth competition between group I and group II
strains. Horticulturae.

[B48] Zhang X, Zhao M, Yan J, Yang L, Yang Y, Guan W, Zhao T (2018). Involvement of hrpX and hrpG in the virulence of Acidovorax
citrulli strain Aac5, causal agent of bacterial fruit blotch in
cucurbits. Front Microbiol.

[B49] Zlatković N, Gasic K, Kuzmanovic N, Prokic A, Ivanovic M, Zovkovic S, Obradovic A (2022). Polyphasic characterization of Acidovorax citrulli strains
originating from serbia. Agronomy.

