# Supplementary material for: Phylogenomic analysis of Paracidovorax citrulli strains reveals the presence of two lineages in Brazil
Source: Genet Mol Biol. 2026 Feb 6;48(4):e20250046. doi: 10.1590/1678-4685-GMB-2025-0046 (PMC12961662; doi:10.1590/1678-4685-GMB-2025-0046)
Supplement: Table S1 - [file 1415-4757-GMB-48-4-e20250046-s1.pdf]

Genetics and Molecular Biology
https://doi.org/10.1590/1678-4685-GMB-2025-0046

Supplementary Material to “Phylogenomic analysis of *Paracidovorax citrulli* strains reveals the presence of two lineages in Brazil”

Table S1 - Predicted proteins for type III (T3S) and IV (T4S) secretion systems of Brazilian strains of *Paracidovorax citrulli* and strains available in GenBank.

*P_citrulli*_CCRMAc5.28

Prot	Pred	Description
fig|80869.157.peg.2307	T3S	major facilitator superfamily MFS_1 [*Paracidovorax citrulli* AAC00-1]
fig|80869.157.peg.3200	T3S	Ig domain protein, group 1 domain protein [*Paracidovorax citrulli* AAC00-1]
fig|80869.157.peg.4351	T3S	hypothetical protein [*Paracidovorax citrulli*]
fig|80869.157.peg.3449	T3S	acyl-CoA thioesterase [*Paracidovorax citrulli*]
fig|80869.157.peg.1571	T3S	STY0301 family protein [*Paracidovorax citrulli*]
fig|80869.157.peg.1975	T3S	ABC transporter ATP-binding protein [*Paracidovorax citrulli*]
fig|80869.157.peg.1	T3S	DUF72 domain-containing protein [*Paracidovorax citrulli*]
fig|80869.157.peg.1021	T3S	rhodanese-like domain-containing protein [*Paracidovorax citrulli*]
fig|80869.157.peg.1038	T3S	GNAT family N-acetyltransferase [*Paracidovorax citrulli*]
fig|80869.157.peg.1051	T3S	ABC transporter permease [*Paracidovorax citrulli*]
fig|80869.157.peg.1055	T3S	hypothetical protein [*Paracidovorax citrulli*]
fig|80869.157.peg.1056	T3S	Metallo-beta-lactamase superfamily protein [*Paracidovorax citrulli*]
fig|80869.157.peg.1068	T3S	Trehalase [*Paracidovorax citrulli*]
fig|80869.157.peg.1088	T3S	hypothetical protein [*Paracidovorax citrulli*]
fig|80869.157.peg.1116	T3S	ABC transporter permease [*Paracidovorax citrulli*]
fig|80869.157.peg.1171	T3S	hypothetical protein [*Paracidovorax citrulli*]
fig|80869.157.peg.1183	T3S	YitT family protein [*Paracidovorax citrulli*]
fig|80869.157.peg.1184	T3S	hypothetical protein [*Paracidovorax citrulli*]
fig|80869.157.peg.1199	T3S	3-methyl-2-oxobutanoate hydroxymethyltransferase [*Paracidovorax citrulli*]
fig|80869.157.peg.1221	T3S	signal recognition particle-docking protein FtsY [*Paracidovorax citrulli*]
fig|80869.157.peg.1231	T3S	MATE family efflux transporter [*Paracidovorax citrulli*]
fig|80869.157.peg.1299	T3S	type III secretion system chaperone [*Paracidovorax citrulli*]
fig|80869.157.peg.1300	T3S	hypothetical protein [*Paracidovorax citrulli*]
fig|80869.157.peg.1326	T3S	luciferase family protein [*Paracidovorax citrulli* AAC00-1]
fig|80869.157.peg.1340	T3S	hypothetical protein [*Paracidovorax citrulli*]
fig|80869.157.peg.1353	T3S	ABC transporter permease [*Paracidovorax citrulli*]
fig|80869.157.peg.2307	T4S	major facilitator superfamily MFS_1 [*Paracidovorax citrulli* AAC00-1]
fig|80869.157.peg.3200	T4S	Ig domain protein, group 1 domain protein [*Paracidovorax citrulli* AAC00-1]
fig|80869.157.peg.4351	T4S	hypothetical protein [*Paracidovorax citrulli*]
fig|80869.157.peg.3449	T4S	acyl-CoA thioesterase [*Paracidovorax citrulli*]

Prot	Pred	Description
fig|80869.157.peg.1571	T4S	STY0301 family protein [*Paracidovorax citrulli*]
fig|80869.157.peg.1975	T4S	ABC transporter ATP-binding protein [*Paracidovorax citrulli*]
fig|80869.157.peg.1678	T4S	Ku protein [*Paracidovorax citrulli*]
fig|80869.157.peg.1214	T4S	ferritin-like domain-containing protein [*Paracidovorax citrulli*]
fig|80869.157.peg.148	T4S	terminase small subunit [*Paracidovorax citrulli*]
fig|80869.157.peg.1877	T4S	molybdenum cofactor biosynthesis protein MoaE [*Paracidovorax citrulli*]
fig|80869.157.peg.459	T4S	hypothetical protein [*Paracidovorax citrulli*]
fig|80869.157.peg.196	T4S	isoaspartyl peptidase/L-asparaginase [*Paracidovorax citrulli*]
fig|80869.157.peg.4396	T4S	NADAR family protein [Paracandidimonas lactea]
fig|80869.157.peg.3488	T4S	RNA-binding protein [*Paracidovorax citrulli*]
fig|80869.157.peg.3973	T4S	DUF6479 family protein [*Paracidovorax citrulli*]
fig|80869.157.peg.3076	T4S	DUF1795 domain-containing protein [*Paracidovorax citrulli*]
fig|80869.157.peg.1710	T4S	amino-acid N-acetyltransferase [*Paracidovorax citrulli*]
fig|80869.157.peg.4244	T4S	DUF924 family protein [*Paracidovorax citrulli*]
fig|80869.157.peg.3876	T4S	50S ribosomal protein L11 [*Paracidovorax citrulli*]
fig|80869.157.peg.1681	T4S	hypothetical protein [*Paracidovorax citrulli*]
fig|80869.157.peg.2281	T4S	hypothetical protein C8E08_4485 [*Paracidovorax citrulli*]
fig|80869.157.peg.2513	T4S	LysR family transcriptional regulator [Paracidovorax]
fig|80869.157.peg.4124	T4S	SMC-Scp complex subunit ScpB [*Paracidovorax citrulli*]
fig|80869.157.peg.842	T4S	PP2C family serine/threonine-protein phosphatase [*Paracidovorax citrulli*]
fig|80869.157.peg.2184	T4S	hypothetical protein [*Paracidovorax citrulli*]
fig|80869.157.peg.3897	T4S	cryptochrome/photolyase family protein [*Paracidovorax citrulli*]
fig|80869.157.peg.1799	T4S	phosphopyruvate hydratase [*Paracidovorax citrulli*]
fig|80869.157.peg.1266	T4S	glutathione-regulated potassium-efflux system protein KefC [*Paracidovorax citrulli*]
fig|80869.157.peg.786	T4S	hypothetical protein [*Paracidovorax citrulli*]
fig|80869.157.peg.563	T4S	hypothetical protein [*Paracidovorax citrulli*]
fig|80869.157.peg.25	T4S	ATP-binding protein [*Paracidovorax citrulli*]
fig|80869.157.peg.3831	T4S	DUF3025 domain-containing protein [*Paracidovorax citrulli*]
fig|80869.157.peg.843	T4S	serine/threonine-protein kinase [*Paracidovorax citrulli*]
fig|80869.157.peg.4335	T4S	hypothetical protein [*Paracidovorax citrulli*]
fig|80869.157.peg.3944	T4S	inositol monophosphatase family protein [*Paracidovorax citrulli*]
fig|80869.157.peg.1103	T4S	N-acetylmuramoyl-L-alanine amidase [*Paracidovorax citrulli*]
fig|80869.157.peg.2950	T4S	AraC family transcriptional regulator [*Paracidovorax citrulli*]
fig|80869.157.peg.622	T4S	hypothetical protein [*Paracidovorax citrulli*]
fig|80869.157.peg.1369	T4S	Fic family protein [*Paracidovorax citrulli*]
fig|80869.157.peg.4317	T4S	4-hydroxy-3-methylbut-2-enyl diphosphate reductase [*Paracidovorax citrulli*]
fig|80869.157.peg.4197	T4S	SsrA-binding protein SmpB [*Paracidovorax citrulli*]
fig|80869.157.peg.2814	T4S	F0F1 ATP synthase subunit gamma [*Paracidovorax citrulli*]
fig|80869.157.peg.364	T4S	hypothetical protein [*Paracidovorax citrulli*]
fig|80869.157.peg.3066	T4S	transglycosylase SLT domain-containing protein [*Paracidovorax citrulli*]
fig|80869.157.peg.2971	T4S	type III secretion system outer membrane ring subunit SctC [*Paracidovorax citrulli*]
fig|80869.157.peg.129	T4S	hypothetical protein [*Paracidovorax citrulli*]
fig|80869.157.peg.247	T4S	XopE/AvrPphe family type III secretion system effector [*Paracidovorax citrulli*]
fig|80869.157.peg.1328	T4S	5'-nucleotidase [*Paracidovorax citrulli*]
fig|80869.157.peg.2915	T4S	30S ribosomal protein S4 [*Paracidovorax citrulli*]

Prot	Pred	Description
fig|80869.157.peg.2975	T4S	adenylate kinase [*Paracidovorax citrulli*]
fig|80869.157.peg.544	T4S	hypothetical protein [*Paracidovorax citrulli*]
fig|80869.157.peg.3371	T4S	hypothetical protein [*Paracidovorax citrulli*]
fig|80869.157.peg.812	T4S	type IV pilin protein [*Paracidovorax citrulli*]
fig|80869.157.peg.1215	T4S	BON domain-containing protein [*Paracidovorax citrulli*]
fig|80869.157.peg.1696	T4S	MetQ/NlpA family ABC transporter substrate-binding protein [*Paracidovorax citrulli*]
fig|80869.157.peg.4164	T4S	Bug family tripartite tricarboxylate transporter substrate binding protein [*Paracidovorax citrulli*]
fig|80869.157.peg.31	T4S	tripartite tricarboxylate transporter substrate binding protein [*Paracidovorax citrulli*]
fig|80869.157.peg.1702	T4S	sulfate ABC transporter substrate-binding protein [*Paracidovorax citrulli*]
fig|80869.157.peg.3707	T4S	hypothetical protein [*Paracidovorax citrulli*]
fig|80869.157.peg.4245	T4S	OmpA family protein [*Paracidovorax citrulli*]
fig|80869.157.peg.111	T4S	endolysin [*Paracidovorax citrulli*]
fig|80869.157.peg.654	T4S	sigma-54 dependent transcriptional regulator [*Paracidovorax citrulli*]
fig|80869.157.peg.1329	T4S	EF-hand domain-containing protein [*Paracidovorax citrulli*]

*P_citrulli_*CCRMAc1.12

Prot	Pred	Description
fig|80869.149.peg.1943	T3S	major facilitator superfamily MFS_1 [*Paracidovorax citrulli* AAC00-1]
fig|80869.149.peg.3169	T3S	Ig domain protein, group 1 domain protein [*Paracidovorax citrulli* AAC00-1]
fig|80869.149.peg.4275	T3S	hypothetical protein [*Paracidovorax citrulli*]
fig|80869.149.peg.3398	T3S	acyl-CoA thioesterase [*Paracidovorax citrulli*]
fig|80869.149.peg.1386	T3S	STY0301 family protein [*Paracidovorax citrulli*]
fig|80869.149.peg.491	T3S	XopE/AvrPphe family type III secretion system effector [*Paracidovorax citrulli*]
fig|80869.149.peg.1611	T3S	ABC transporter ATP-binding protein [*Paracidovorax citrulli*]
fig|80869.149.peg.1009	T3S	NADH-quinone oxidoreductase subunit NuoI [*Paracidovorax citrulli*]
fig|80869.149.peg.1017	T3S	ABC transporter transmembrane domain-containing protein [*Paracidovorax citrulli*]
fig|80869.149.peg.1035	T3S	helix-turn-helix domain-containing protein [*Paracidovorax citrulli*]
fig|80869.149.peg.1055	T3S	HDOD domain-containing protein [*Paracidovorax citrulli*]
fig|80869.149.peg.1059	T3S	hypothetical protein [*Paracidovorax citrulli*]
fig|80869.149.peg.106	T3S	TetR/AcrR family transcriptional regulator [*Paracidovorax citrulli*]
fig|80869.149.peg.1085	T3S	pyridoxal kinase PdxY [*Paracidovorax citrulli*]
fig|80869.149.peg.1091	T3S	DNA internalization-related competence protein ComEC/Rec2 [*Paracidovorax citrulli*]
fig|80869.149.peg.1099	T3S	VF530 family protein [*Paracidovorax citrulli*]
fig|80869.149.peg.1131	T3S	CaiB/BaiF CoA-transferase family protein [*Paracidovorax citrulli*]
fig|80869.149.peg.1148	T3S	alpha/beta hydrolase [*Paracidovorax citrulli*]
fig|80869.149.peg.1177	T3S	septal ring lytic transglycosylase RlpA family protein [*Paracidovorax citrulli*]
fig|80869.149.peg.1190	T3S	hypothetical protein [*Paracidovorax citrulli*]
fig|80869.149.peg.1197	T3S	TRAP transporter small permease [Paracidovorax]
fig|80869.149.peg.1250	T3S	rhodanese-like domain-containing protein [*Paracidovorax citrulli*]
fig|80869.149.peg.1267	T3S	GNAT family N-acetyltransferase [*Paracidovorax citrulli*]
fig|80869.149.peg.1280	T3S	ABC transporter permease [*Paracidovorax citrulli*]
fig|80869.149.peg.1284	T3S	hypothetical protein [*Paracidovorax citrulli*]

Prot	Pred	Description
fig|80869.149.peg.1285	T3S	Metallo-beta-lactamase superfamily protein [*Paracidovorax citrulli*]
fig|80869.149.peg.1297	T3S	Trehalase [*Paracidovorax citrulli*]
fig|80869.149.peg.1943	T4S	major facilitator superfamily MFS_1 [*Paracidovorax citrulli* AAC00-1]
fig|80869.149.peg.3169	T4S	Ig domain protein, group 1 domain protein [*Paracidovorax citrulli* AAC00-1]
fig|80869.149.peg.4275	T4S	hypothetical protein [*Paracidovorax citrulli*]
fig|80869.149.peg.3398	T4S	acyl-CoA thioesterase [*Paracidovorax citrulli*]
fig|80869.149.peg.1386	T4S	STY0301 family protein [*Paracidovorax citrulli*]
fig|80869.149.peg.491	T4S	avirulence protein [*Paracidovorax citrulli*]
fig|80869.149.peg.1611	T4S	ABC transporter ATP-binding protein [*Paracidovorax citrulli*]
fig|80869.149.peg.1492	T4S	Ku protein [*Paracidovorax citrulli*]
fig|80869.149.peg.2440	T4S	ferritin-like domain-containing protein [*Paracidovorax citrulli*]
fig|80869.149.peg.983	T4S	molybdenum cofactor biosynthesis protein MoaE [*Paracidovorax citrulli*]
fig|80869.149.peg.137	T4S	hypothetical protein [*Paracidovorax citrulli*]
fig|80869.149.peg.440	T4S	isoaspartyl peptidase/L-asparaginase [*Paracidovorax citrulli*]
fig|80869.149.peg.4318	T4S	NADAR family protein [Paracandidimonas lactea]
fig|80869.149.peg.3436	T4S	RNA-binding protein [*Paracidovorax citrulli*]
fig|80869.149.peg.40	T4S	DUF6479 family protein [*Paracidovorax citrulli*]
fig|80869.149.peg.3047	T4S	DUF1795 domain-containing protein [*Paracidovorax citrulli*]
fig|80869.149.peg.1525	T4S	amino-acid N-acetyltransferase [*Paracidovorax citrulli*]
fig|80869.149.peg.4148	T4S	DUF924 family protein [*Paracidovorax citrulli*]
fig|80869.149.peg.3829	T4S	50S ribosomal protein L11 [*Paracidovorax citrulli*]
fig|80869.149.peg.1495	T4S	hypothetical protein [*Paracidovorax citrulli*]
fig|80869.149.peg.1917	T4S	hypothetical protein C8E08_4485 [*Paracidovorax citrulli*]
fig|80869.149.peg.4027	T4S	SMC-Scp complex subunit ScpB [*Paracidovorax citrulli*]
fig|80869.149.peg.811	T4S	PP2C family serine/threonine-protein phosphatase [*Paracidovorax citrulli*]
fig|80869.149.peg.1821	T4S	hypothetical protein [*Paracidovorax citrulli*]
fig|80869.149.peg.3850	T4S	cryptochrome/photolyase family protein [*Paracidovorax citrulli*]
fig|80869.149.peg.1061	T4S	phosphopyruvate hydratase [*Paracidovorax citrulli*]
fig|80869.149.peg.2388	T4S	glutathione-regulated potassium-efflux system protein KefC [*Paracidovorax citrulli*]
fig|80869.149.peg.755	T4S	hypothetical protein [*Paracidovorax citrulli*]
fig|80869.149.peg.241	T4S	hypothetical protein [*Paracidovorax citrulli*]
fig|80869.149.peg.349	T4S	ATP-binding protein [*Paracidovorax citrulli*]
fig|80869.149.peg.3784	T4S	DUF3025 domain-containing protein [*Paracidovorax citrulli*]
fig|80869.149.peg.812	T4S	serine/threonine-protein kinase [*Paracidovorax citrulli*]
fig|80869.149.peg.4239	T4S	hypothetical protein [*Paracidovorax citrulli*]
fig|80869.149.peg.3897	T4S	inositol monophosphatase family protein [*Paracidovorax citrulli*]
fig|80869.149.peg.1332	T4S	N-acetylmuramoyl-L-alanine amidase [*Paracidovorax citrulli*]
fig|80869.149.peg.2734	T4S	AraC family transcriptional regulator [*Paracidovorax citrulli*]
fig|80869.149.peg.299	T4S	hypothetical protein [*Paracidovorax citrulli*]
fig|80869.149.peg.3318	T4S	Fic family protein [*Paracidovorax citrulli*]
fig|80869.149.peg.4221	T4S	4-hydroxy-3-methylbut-2-enyl diphosphate reductase [*Paracidovorax citrulli*]
fig|80869.149.peg.4101	T4S	SsrA-binding protein SmpB [*Paracidovorax citrulli*]
fig|80869.149.peg.2598	T4S	F0F1 ATP synthase subunit gamma [*Paracidovorax citrulli*]
fig|80869.149.peg.610	T4S	hypothetical protein [*Paracidovorax citrulli*]
fig|80869.149.peg.2949	T4S	transglycosylase SLT domain-containing protein [*Paracidovorax citrulli*]

Prot	Pred	Description
fig|80869.149.peg.2754	T4S	type III secretion system outer membrane ring subunit SctC [*Paracidovorax citrulli*]
fig|80869.149.peg.3012	T4S	5'-nucleotidase [*Paracidovorax citrulli*]
fig|80869.149.peg.2699	T4S	30S ribosomal protein S4 [*Paracidovorax citrulli*]
fig|80869.149.peg.2858	T4S	adenylate kinase [*Paracidovorax citrulli*]
fig|80869.149.peg.222	T4S	hypothetical protein [*Paracidovorax citrulli*]
fig|80869.149.peg.3249	T4S	hypothetical protein [*Paracidovorax citrulli*]
fig|80869.149.peg.781	T4S	type IV pilin protein [*Paracidovorax citrulli*]
fig|80869.149.peg.2439	T4S	BON domain-containing protein [*Paracidovorax citrulli*]
fig|80869.149.peg.1511	T4S	MetQ/NlpA family ABC transporter substrate-binding protein [*Paracidovorax citrulli*]
fig|80869.149.peg.4068	T4S	Bug family tripartite tricarboxylate transporter substrate binding protein [*Paracidovorax citrulli*]
fig|80869.149.peg.355	T4S	tripartite tricarboxylate transporter substrate binding protein [*Paracidovorax citrulli*]
fig|80869.149.peg.1517	T4S	sulfate ABC transporter substrate-binding protein [*Paracidovorax citrulli*]
fig|80869.149.peg.3658	T4S	hypothetical protein [*Paracidovorax citrulli*]
fig|80869.149.peg.4149	T4S	OmpA family protein [*Paracidovorax citrulli*]
fig|80869.149.peg.2166	T4S	HTH-type transcriptional regulator GltC [*Paracidovorax citrulli*]
fig|80869.149.peg.623	T4S	sigma-54 dependent transcriptional regulator [*Paracidovorax citrulli*]
fig|80869.149.peg.3013	T4S	EF-hand domain-containing protein [*Paracidovorax citrulli*]

*P_citrulli_*CCRMAc1.43

Prot	Pred	Description
fig|80869.150.peg.2346	T3S	major facilitator superfamily MFS_1 [*Paracidovorax citrulli* AAC00-1]
fig|80869.150.peg.3238	T3S	Ig domain protein, group 1 domain protein [*Paracidovorax citrulli* AAC00-1]
fig|80869.150.peg.4369	T3S	hypothetical protein [*Paracidovorax citrulli*]
fig|80869.150.peg.3489	T3S	acyl-CoA thioesterase [*Paracidovorax citrulli*]
fig|80869.150.peg.1614	T3S	STY0301 family protein [*Paracidovorax citrulli*]
fig|80869.150.peg.2016	T3S	ABC transporter ATP-binding protein [*Paracidovorax citrulli*]
fig|80869.150.peg.1	T3S	DUF72 domain-containing protein [*Paracidovorax citrulli*]
fig|80869.150.peg.1016	T3S	TRAP transporter small permease [Paracidovorax]
fig|80869.150.peg.1068	T3S	rhodanese-like domain-containing protein [*Paracidovorax citrulli*]
fig|80869.150.peg.1084	T3S	GNAT family N-acetyltransferase [*Paracidovorax citrulli*]
fig|80869.150.peg.1097	T3S	ABC transporter permease [*Paracidovorax citrulli*]
fig|80869.150.peg.1102	T3S	Metallo-beta-lactamase superfamily protein [*Paracidovorax citrulli*]
fig|80869.150.peg.1114	T3S	Trehalase [*Paracidovorax citrulli*]
fig|80869.150.peg.1134	T3S	hypothetical protein [*Paracidovorax citrulli*]
fig|80869.150.peg.1162	T3S	ABC transporter permease [*Paracidovorax citrulli*]
fig|80869.150.peg.1217	T3S	hypothetical protein [*Paracidovorax citrulli*]
fig|80869.150.peg.1229	T3S	YitT family protein [*Paracidovorax citrulli*]
fig|80869.150.peg.1230	T3S	hypothetical protein [*Paracidovorax citrulli*]
fig|80869.150.peg.1245	T3S	3-methyl-2-oxobutanoate hydroxymethyltransferase [*Paracidovorax citrulli*]
fig|80869.150.peg.1267	T3S	signal recognition particle-docking protein FtsY [*Paracidovorax citrulli*]
fig|80869.150.peg.1277	T3S	MATE family efflux transporter [*Paracidovorax citrulli*]
fig|80869.150.peg.1344	T3S	type III secretion system chaperone [*Paracidovorax citrulli*]
fig|80869.150.peg.1345	T3S	hypothetical protein [*Paracidovorax citrulli*]

Prot	Pred	Description
fig|80869.150.peg.1372	T3S	LLM class flavin-dependent oxidoreductase [*Paracidovorax citrulli*]
fig|80869.150.peg.1385	T3S	hypothetical protein [*Paracidovorax citrulli*]
fig|80869.150.peg.2346	T4S	major facilitator superfamily MFS_1 [*Paracidovorax citrulli* AAC00-1]
fig|80869.150.peg.3238	T4S	Ig domain protein, group 1 domain protein [*Paracidovorax citrulli* AAC00-1]
fig|80869.150.peg.4369	T4S	hypothetical protein [*Paracidovorax citrulli*]
fig|80869.150.peg.3489	T4S	acyl-CoA thioesterase [*Paracidovorax citrulli*]
fig|80869.150.peg.1614	T4S	STY0301 family protein [*Paracidovorax citrulli*]
fig|80869.150.peg.2016	T4S	ABC transporter ATP-binding protein [*Paracidovorax citrulli*]
fig|80869.150.peg.1720	T4S	Ku protein [*Paracidovorax citrulli*]
fig|80869.150.peg.1723	T4S	hypothetical protein [*Paracidovorax citrulli*]
fig|80869.150.peg.1260	T4S	ferritin-like domain-containing protein [*Paracidovorax citrulli*]
fig|80869.150.peg.148	T4S	terminase small subunit [*Paracidovorax citrulli*]
fig|80869.150.peg.1919	T4S	molybdenum cofactor biosynthesis protein MoaE [*Paracidovorax citrulli*]
fig|80869.150.peg.507	T4S	hypothetical protein [*Paracidovorax citrulli*]
fig|80869.150.peg.196	T4S	isoaspartyl peptidase/L-asparaginase [*Paracidovorax citrulli*]
fig|80869.150.peg.4413	T4S	NADAR family protein [Paracandidimonas lactea]
fig|80869.150.peg.3528	T4S	RNA-binding protein [*Paracidovorax citrulli*]
fig|80869.150.peg.411	T4S	hypothetical protein Aave_3072 [*Paracidovorax citrulli* AAC00-1]
fig|80869.150.peg.3114	T4S	DUF1795 domain-containing protein [*Paracidovorax citrulli*]
fig|80869.150.peg.1753	T4S	amino-acid N-acetyltransferase [*Paracidovorax citrulli*]
fig|80869.150.peg.4263	T4S	DUF924 family protein [*Paracidovorax citrulli*]
fig|80869.150.peg.3944	T4S	50S ribosomal protein L11 [*Paracidovorax citrulli*]
fig|80869.150.peg.2321	T4S	hypothetical protein C8E08_4485 [*Paracidovorax citrulli*]
fig|80869.150.peg.4142	T4S	SMC-Scp complex subunit ScpB [*Paracidovorax citrulli*]
fig|80869.150.peg.888	T4S	PP2C family serine/threonine-protein phosphatase [*Paracidovorax citrulli*]
fig|80869.150.peg.2225	T4S	hypothetical protein [*Paracidovorax citrulli*]
fig|80869.150.peg.3966	T4S	cryptochrome/photolyase family protein [*Paracidovorax citrulli*]
fig|80869.150.peg.1841	T4S	phosphopyruvate hydratase [*Paracidovorax citrulli*]
fig|80869.150.peg.1312	T4S	glutathione-regulated potassium-efflux system protein KefC [*Paracidovorax citrulli*]
fig|80869.150.peg.832	T4S	hypothetical protein [*Paracidovorax citrulli*]
fig|80869.150.peg.612	T4S	hypothetical protein [*Paracidovorax citrulli*]
fig|80869.150.peg.25	T4S	ATP-binding protein [*Paracidovorax citrulli*]
fig|80869.150.peg.3898	T4S	DUF3025 domain-containing protein [*Paracidovorax citrulli*]
fig|80869.150.peg.889	T4S	serine/threonine-protein kinase [*Paracidovorax citrulli*]
fig|80869.150.peg.4354	T4S	hypothetical protein [*Paracidovorax citrulli*]
fig|80869.150.peg.4013	T4S	inositol monophosphatase family protein [*Paracidovorax citrulli*]
fig|80869.150.peg.1149	T4S	N-acetylmuramoyl-L-alanine amidase [*Paracidovorax citrulli*]
fig|80869.150.peg.2989	T4S	AraC family transcriptional regulator [*Paracidovorax citrulli*]
fig|80869.150.peg.670	T4S	hypothetical protein [*Paracidovorax citrulli*]
fig|80869.150.peg.1414	T4S	Fic family protein [*Paracidovorax citrulli*]
fig|80869.150.peg.4555	T4S	type VI secretion system tip protein VgrG [*Paracidovorax citrulli*]
fig|80869.150.peg.4336	T4S	4-hydroxy-3-methylbut-2-enyl diphosphate reductase [*Paracidovorax citrulli*]
fig|80869.150.peg.4216	T4S	SsrA-binding protein SmpB [*Paracidovorax citrulli*]
fig|80869.150.peg.2854	T4S	F0F1 ATP synthase subunit gamma [*Paracidovorax citrulli*]
fig|80869.150.peg.365	T4S	hypothetical protein [*Paracidovorax citrulli*]

Prot	Pred	Description
fig|80869.150.peg.3104	T4S	transglycosylase SLT domain-containing protein [*Paracidovorax citrulli*]
fig|80869.150.peg.3009	T4S	type III secretion system outer membrane ring subunit SctC [*Paracidovorax citrulli*]
fig|80869.150.peg.129	T4S	hypothetical protein [*Paracidovorax citrulli*]
fig|80869.150.peg.246	T4S	XopE/AvrPphe family type III secretion system effector [*Paracidovorax citrulli*]
fig|80869.150.peg.1374	T4S	5'-nucleotidase [*Paracidovorax citrulli*]
fig|80869.150.peg.2954	T4S	30S ribosomal protein S4 [*Paracidovorax citrulli*]
fig|80869.150.peg.3013	T4S	adenylate kinase [*Paracidovorax citrulli*]
fig|80869.150.peg.593	T4S	hypothetical protein [*Paracidovorax citrulli*]
fig|80869.150.peg.3411	T4S	hypothetical protein [*Paracidovorax citrulli*]
fig|80869.150.peg.858	T4S	type IV pilin protein [*Paracidovorax citrulli*]
fig|80869.150.peg.1261	T4S	BON domain-containing protein [*Paracidovorax citrulli*]
fig|80869.150.peg.1739	T4S	MetQ/NlpA family ABC transporter substrate-binding protein [*Paracidovorax citrulli*]
fig|80869.150.peg.4183	T4S	Bug family tripartite tricarboxylate transporter substrate binding protein [*Paracidovorax citrulli*]
fig|80869.150.peg.31	T4S	tripartite tricarboxylate transporter substrate binding protein [*Paracidovorax citrulli*]
fig|80869.150.peg.1745	T4S	sulfate ABC transporter substrate-binding protein [*Paracidovorax citrulli*]
fig|80869.150.peg.3672	T4S	hypothetical protein [*Paracidovorax citrulli*]
fig|80869.150.peg.4264	T4S	OmpA family protein [*Paracidovorax citrulli*]
fig|80869.150.peg.111	T4S	endolysin [*Paracidovorax citrulli*]
fig|80869.150.peg.2555	T4S	HTH-type transcriptional regulator GltC [*Paracidovorax citrulli*]
fig|80869.150.peg.702	T4S	sigma-54 dependent transcriptional regulator [*Paracidovorax citrulli*]
fig|80869.150.peg.1375	T4S	EF-hand domain-containing protein [*Paracidovorax citrulli*]

*P_citrulli_*CCRMAc1.45

Prot	Pred	Description
fig|80869.151.peg.2949	T3S	Ig domain protein, group 1 domain protein [*Paracidovorax citrulli* AAC00-1]
fig|80869.151.peg.3592	T3S	major facilitator superfamily MFS_1 [*Paracidovorax citrulli* AAC00-1]
fig|80869.151.peg.4337	T3S	hypothetical protein [*Paracidovorax citrulli*]
fig|80869.151.peg.3199	T3S	acyl-CoA thioesterase [*Paracidovorax citrulli*]
fig|80869.151.peg.1808	T3S	STY0301 family protein [*Paracidovorax citrulli*]
fig|80869.151.peg.2215	T3S	ABC transporter ATP-binding protein [*Paracidovorax citrulli*]
fig|80869.151.peg.1	T3S	DUF72 domain-containing protein [*Paracidovorax citrulli*]
fig|80869.151.peg.1025	T3S	ribonucleoside-diphosphate reductase subunit alpha [*Paracidovorax citrulli*]
fig|80869.151.peg.1038	T3S	3-deoxy-7-phosphoheptulonate synthase [*Paracidovorax citrulli*]
fig|80869.151.peg.1085	T3S	GTP-binding protein [*Paracidovorax citrulli*]
fig|80869.151.peg.1096	T3S	chromate efflux transporter [*Paracidovorax citrulli*]
fig|80869.151.peg.1103	T3S	M48 family metallopeptidase [*Paracidovorax citrulli*]
fig|80869.151.peg.1119	T3S	DNA-3-methyladenine glycosylase I [*Paracidovorax citrulli*]
fig|80869.151.peg.1120	T3S	aminopeptidase [*Paracidovorax citrulli*]
fig|80869.151.peg.1151	T3S	DUF2169 domain-containing protein [*Paracidovorax citrulli*]
fig|80869.151.peg.1160	T3S	alpha/beta hydrolase [*Paracidovorax citrulli*]
fig|80869.151.peg.1189	T3S	septal ring lytic transglycosylase RlpA family protein [*Paracidovorax citrulli*]
fig|80869.151.peg.1202	T3S	hypothetical protein [*Paracidovorax citrulli*]

Prot	Pred	Description
fig|80869.151.peg.1209	T3S	TRAP transporter small permease [Paracidovorax]
fig|80869.151.peg.1261	T3S	rhodanese-like domain-containing protein [*Paracidovorax citrulli*]
fig|80869.151.peg.1278	T3S	GNAT family N-acetyltransferase [*Paracidovorax citrulli*]
fig|80869.151.peg.1291	T3S	ABC transporter permease [*Paracidovorax citrulli*]
fig|80869.151.peg.1296	T3S	Metallo-beta-lactamase superfamily protein [*Paracidovorax citrulli*]
fig|80869.151.peg.1308	T3S	Trehalase [*Paracidovorax citrulli*]
fig|80869.151.peg.1328	T3S	hypothetical protein [*Paracidovorax citrulli*]
fig|80869.151.peg.1356	T3S	ABC transporter permease [*Paracidovorax citrulli*]
fig|80869.151.peg.2949	T4S	Ig domain protein, group 1 domain protein [*Paracidovorax citrulli* AAC00-1]
fig|80869.151.peg.3592	T4S	major facilitator superfamily MFS_1 [*Paracidovorax citrulli* AAC00-1]
fig|80869.151.peg.4337	T4S	hypothetical protein [*Paracidovorax citrulli*]
fig|80869.151.peg.3199	T4S	acyl-CoA thioesterase [*Paracidovorax citrulli*]
fig|80869.151.peg.1808	T4S	STY0301 family protein [*Paracidovorax citrulli*]
fig|80869.151.peg.2215	T4S	ABC transporter ATP-binding protein [*Paracidovorax citrulli*]
fig|80869.151.peg.1916	T4S	Ku protein [*Paracidovorax citrulli*]
fig|80869.151.peg.1454	T4S	ferritin-like domain-containing protein [*Paracidovorax citrulli*]
fig|80869.151.peg.148	T4S	terminase small subunit [*Paracidovorax citrulli*]
fig|80869.151.peg.2117	T4S	molybdenum cofactor biosynthesis protein MoaE [*Paracidovorax citrulli*]
fig|80869.151.peg.458	T4S	hypothetical protein [*Paracidovorax citrulli*]
fig|80869.151.peg.197	T4S	isoaspartyl peptidase/L-asparaginase [*Paracidovorax citrulli*]
fig|80869.151.peg.4380	T4S	NADAR family protein [Paracandidimonas lactea]
fig|80869.151.peg.3238	T4S	RNA-binding protein [*Paracidovorax citrulli*]
fig|80869.151.peg.3908	T4S	hypothetical protein Aave_3072 [*Paracidovorax citrulli* AAC00-1]
fig|80869.151.peg.3420	T4S	DUF1795 domain-containing protein [*Paracidovorax citrulli*]
fig|80869.151.peg.1949	T4S	amino-acid N-acetyltransferase [*Paracidovorax citrulli*]
fig|80869.151.peg.4178	T4S	DUF924 family protein [*Paracidovorax citrulli*]
fig|80869.151.peg.3853	T4S	50S ribosomal protein L11 [*Paracidovorax citrulli*]
fig|80869.151.peg.1919	T4S	hypothetical protein [*Paracidovorax citrulli*]
fig|80869.151.peg.3567	T4S	hypothetical protein C8E08_4485 [*Paracidovorax citrulli*]
fig|80869.151.peg.4056	T4S	SMC-Scp complex subunit ScpB [*Paracidovorax citrulli*]
fig|80869.151.peg.840	T4S	PP2C family serine/threonine-protein phosphatase [*Paracidovorax citrulli*]
fig|80869.151.peg.4432	T4S	PAS domain S-box protein [*Paracidovorax citrulli*]
fig|80869.151.peg.2425	T4S	hypothetical protein [*Paracidovorax citrulli*]
fig|80869.151.peg.3874	T4S	cryptochrome/photolyase family protein [*Paracidovorax citrulli*]
fig|80869.151.peg.2039	T4S	phosphopyruvate hydratase [*Paracidovorax citrulli*]
fig|80869.151.peg.1506	T4S	glutathione-regulated potassium-efflux system protein KefC [*Paracidovorax citrulli*]
fig|80869.151.peg.784	T4S	hypothetical protein [*Paracidovorax citrulli*]
fig|80869.151.peg.561	T4S	hypothetical protein [*Paracidovorax citrulli*]
fig|80869.151.peg.25	T4S	ATP-binding protein [*Paracidovorax citrulli*]
fig|80869.151.peg.3807	T4S	DUF3025 domain-containing protein [*Paracidovorax citrulli*]
fig|80869.151.peg.841	T4S	serine/threonine-protein kinase [*Paracidovorax citrulli*]
fig|80869.151.peg.4321	T4S	hypothetical protein [*Paracidovorax citrulli*]
fig|80869.151.peg.4198	T4S	inositol monophosphatase family protein [*Paracidovorax citrulli*]
fig|80869.151.peg.1343	T4S	N-acetylmuramoyl-L-alanine amidase [*Paracidovorax citrulli*]
fig|80869.151.peg.2801	T4S	AraC family transcriptional regulator [*Paracidovorax citrulli*]
fig|80869.151.peg.620	T4S	hypothetical protein [*Paracidovorax citrulli*]

Prot	Pred	Description
fig|80869.151.peg.1609	T4S	Fic family protein [*Paracidovorax citrulli*]
fig|80869.151.peg.4303	T4S	4-hydroxy-3-methylbut-2-enyl diphosphate reductase [*Paracidovorax citrulli*]
fig|80869.151.peg.4131	T4S	SsrA-binding protein SmpB [*Paracidovorax citrulli*]
fig|80869.151.peg.2666	T4S	F0F1 ATP synthase subunit gamma [*Paracidovorax citrulli*]
fig|80869.151.peg.364	T4S	hypothetical protein [*Paracidovorax citrulli*]
fig|80869.151.peg.2916	T4S	transglycosylase SLT domain-containing protein [*Paracidovorax citrulli*]
fig|80869.151.peg.2821	T4S	type III secretion system outer membrane ring subunit SctC [*Paracidovorax citrulli*]
fig|80869.151.peg.129	T4S	hypothetical protein [*Paracidovorax citrulli*]
fig|80869.151.peg.247	T4S	XopE/AvrPphe family type III secretion system effector [*Paracidovorax citrulli*]
fig|80869.151.peg.1568	T4S	5'-nucleotidase [*Paracidovorax citrulli*]
fig|80869.151.peg.2766	T4S	30S ribosomal protein S4 [*Paracidovorax citrulli*]
fig|80869.151.peg.2825	T4S	adenylate kinase [*Paracidovorax citrulli*]
fig|80869.151.peg.542	T4S	hypothetical protein [*Paracidovorax citrulli*]
fig|80869.151.peg.3122	T4S	hypothetical protein [*Paracidovorax citrulli*]
fig|80869.151.peg.810	T4S	type IV pilin protein [*Paracidovorax citrulli*]
fig|80869.151.peg.3827	T4S	hypothetical protein [*Paracidovorax citrulli*]
fig|80869.151.peg.1455	T4S	BON domain-containing protein [*Paracidovorax citrulli*]
fig|80869.151.peg.1935	T4S	MetQ/NlpA family ABC transporter substrate-binding protein [*Paracidovorax citrulli*]
fig|80869.151.peg.4097	T4S	Bug family tripartite tricarboxylate transporter substrate binding protein [*Paracidovorax citrulli*]
fig|80869.151.peg.31	T4S	tripartite tricarboxylate transporter substrate binding protein [*Paracidovorax citrulli*]
fig|80869.151.peg.1941	T4S	sulfate ABC transporter substrate-binding protein [*Paracidovorax citrulli*]
fig|80869.151.peg.3681	T4S	hypothetical protein [*Paracidovorax citrulli*]
fig|80869.151.peg.4179	T4S	OmpA family protein [*Paracidovorax citrulli*]
fig|80869.151.peg.111	T4S	endolysin [*Paracidovorax citrulli*]
fig|80869.151.peg.2484	T4S	HTH-type transcriptional regulator GltC [*Paracidovorax citrulli*]
fig|80869.151.peg.653	T4S	sigma-54 dependent transcriptional regulator [*Paracidovorax citrulli*]
fig|80869.151.peg.1569	T4S	EF-hand domain-containing protein [*Paracidovorax citrulli*]

*P_citrulli*_CCRMAc1.73

Prot	Pred	Description
fig|80869.152.peg.1030	T3S	major facilitator superfamily MFS_1 [*Paracidovorax citrulli* AAC00-1]
fig|80869.152.peg.3191	T3S	Ig domain protein, group 1 domain protein [*Paracidovorax citrulli* AAC00-1]
fig|80869.152.peg.4393	T3S	hypothetical protein [*Paracidovorax citrulli*]
fig|80869.152.peg.3348	T3S	acyl-CoA thioesterase [*Paracidovorax citrulli*]
fig|80869.152.peg.1363	T3S	STY0301 family protein [*Paracidovorax citrulli*]
fig|80869.152.peg.1760	T3S	ABC transporter ATP-binding protein [*Paracidovorax citrulli*]
fig|80869.152.peg.1	T3S	DUF72 domain-containing protein [*Paracidovorax citrulli*]
fig|80869.152.peg.1002	T3S	SDR family oxidoreductase [*Paracidovorax citrulli*]
fig|80869.152.peg.1003	T3S	sugar lactone lactonase YvrE [*Paracidovorax citrulli*]
fig|80869.152.peg.1011	T3S	GAF domain-containing sensor histidine kinase [*Paracidovorax citrulli*]
fig|80869.152.peg.1018	T3S	major facilitator superfamily MFS_1 [*Paracidovorax citrulli* AAC00-1]
fig|80869.152.peg.1020	T3S	redox-sensitive transcriptional activator SoxR [*Paracidovorax citrulli*]

Prot	Pred	Description
fig|80869.152.peg.1024	T3S	DHHA1 domain-containing protein [*Paracidovorax citrulli*]
fig|80869.152.peg.1034	T3S	Murein DD-endopeptidase MepM [*Paracidovorax citrulli*]
fig|80869.152.peg.1037	T3S	alpha/beta hydrolase [*Paracidovorax citrulli*]
fig|80869.152.peg.1048	T3S	glutamine--tRNA ligase/YqeY domain fusion protein [*Paracidovorax citrulli*]
fig|80869.152.peg.1054	T3S	class III extradiol ring-cleavage dioxygenase [*Paracidovorax citrulli*]
fig|80869.152.peg.1199	T3S	hypothetical protein [*Paracidovorax citrulli*]
fig|80869.152.peg.1203	T3S	DUF1653 domain-containing protein [*Paracidovorax citrulli*]
fig|80869.152.peg.1218	T3S	isoleucine--tRNA ligase [*Paracidovorax citrulli*]
fig|80869.152.peg.1220	T3S	hypothetical protein, partial [*Paracidovorax citrulli*]
fig|80869.152.peg.1262	T3S	adenylate/guanylate cyclase domain-containing protein [*Paracidovorax citrulli*]
fig|80869.152.peg.1301	T3S	urease accessory UreF family protein [*Paracidovorax citrulli*]
fig|80869.152.peg.1302	T3S	urease accessory protein UreG [*Paracidovorax citrulli*]
fig|80869.152.peg.1340	T3S	cyclopropane-fatty-acyl-phospholipid synthase family protein [*Paracidovorax citrulli*]
fig|80869.152.peg.1356	T3S	KGG domain-containing protein [*Paracidovorax citrulli*]
fig|80869.152.peg.1399	T3S	PAS domain S-box protein [*Paracidovorax citrulli*]
fig|80869.152.peg.1030	T4S	major facilitator superfamily MFS_1 [*Paracidovorax citrulli* AAC00-1]
fig|80869.152.peg.3191	T4S	Ig domain protein, group 1 domain protein [*Paracidovorax citrulli* AAC00-1]
fig|80869.152.peg.4393	T4S	hypothetical protein [*Paracidovorax citrulli*]
fig|80869.152.peg.3348	T4S	acyl-CoA thioesterase [*Paracidovorax citrulli*]
fig|80869.152.peg.1363	T4S	STY0301 family protein [*Paracidovorax citrulli*]
fig|80869.152.peg.1760	T4S	ABC transporter ATP-binding protein [*Paracidovorax citrulli*]
fig|80869.152.peg.1469	T4S	Ku protein [*Paracidovorax citrulli*]
fig|80869.152.peg.2651	T4S	ferritin-like domain-containing protein [*Paracidovorax citrulli*]
fig|80869.152.peg.148	T4S	terminase small subunit [*Paracidovorax citrulli*]
fig|80869.152.peg.1669	T4S	molybdenum cofactor biosynthesis protein MoaE [*Paracidovorax citrulli*]
fig|80869.152.peg.509	T4S	hypothetical protein [*Paracidovorax citrulli*]
fig|80869.152.peg.196	T4S	isoaspartyl peptidase/L-asparaginase [*Paracidovorax citrulli*]
fig|80869.152.peg.4433	T4S	NADAR family protein [Paracandidimonas lactea]
fig|80869.152.peg.3386	T4S	RNA-binding protein [*Paracidovorax citrulli*]
fig|80869.152.peg.411	T4S	hypothetical protein Aave_3072 [*Paracidovorax citrulli* AAC00-1]
fig|80869.152.peg.3069	T4S	DUF1795 domain-containing protein [*Paracidovorax citrulli*]
fig|80869.152.peg.1502	T4S	amino-acid N-acetyltransferase [*Paracidovorax citrulli*]
fig|80869.152.peg.3508	T4S	DUF924 family protein [*Paracidovorax citrulli*]
fig|80869.152.peg.4519	T4S	50S ribosomal protein L11 [*Paracidovorax citrulli*]
fig|80869.152.peg.1472	T4S	hypothetical protein [*Paracidovorax citrulli*]
fig|80869.152.peg.1004	T4S	hypothetical protein C8E08_4485 [*Paracidovorax citrulli*]
fig|80869.152.peg.4165	T4S	SMC-Scp complex subunit ScpB [*Paracidovorax citrulli*]
fig|80869.152.peg.1347	T4S	PP2C family serine/threonine-protein phosphatase [*Paracidovorax citrulli*]
fig|80869.152.peg.1970	T4S	hypothetical protein [*Paracidovorax citrulli*]
fig|80869.152.peg.4238	T4S	cryptochrome/photolyase family protein [*Paracidovorax citrulli*]
fig|80869.152.peg.1591	T4S	phosphopyruvate hydratase [*Paracidovorax citrulli*]
fig|80869.152.peg.2599	T4S	glutathione-regulated potassium-efflux system protein KefC [*Paracidovorax citrulli*]
fig|80869.152.peg.1291	T4S	hypothetical protein [*Paracidovorax citrulli*]
fig|80869.152.peg.613	T4S	hypothetical protein [*Paracidovorax citrulli*]
fig|80869.152.peg.25	T4S	ATP-binding protein [*Paracidovorax citrulli*]

Prot	Pred	Description
fig|80869.152.peg.4056	T4S	DUF3025 domain-containing protein [*Paracidovorax citrulli*]
fig|80869.152.peg.1348	T4S	serine/threonine-protein kinase [*Paracidovorax citrulli*]
fig|80869.152.peg.4377	T4S	hypothetical protein [*Paracidovorax citrulli*]
fig|80869.152.peg.2453	T4S	inositol monophosphatase family protein [*Paracidovorax citrulli*]
fig|80869.152.peg.890	T4S	N-acetylmuramoyl-L-alanine amidase [*Paracidovorax citrulli*]
fig|80869.152.peg.2944	T4S	AraC family transcriptional regulator [*Paracidovorax citrulli*]
fig|80869.152.peg.672	T4S	hypothetical protein [*Paracidovorax citrulli*]
fig|80869.152.peg.3415	T4S	Fic family protein [*Paracidovorax citrulli*]
fig|80869.152.peg.4359	T4S	4-hydroxy-3-methylbut-2-enyl diphosphate reductase [*Paracidovorax citrulli*]
fig|80869.152.peg.4278	T4S	SsrA-binding protein SmpB [*Paracidovorax citrulli*]
fig|80869.152.peg.2808	T4S	F0F1 ATP synthase subunit gamma [*Paracidovorax citrulli*]
fig|80869.152.peg.365	T4S	hypothetical protein [*Paracidovorax citrulli*]
fig|80869.152.peg.3059	T4S	transglycosylase SLT domain-containing protein [*Paracidovorax citrulli*]
fig|80869.152.peg.2964	T4S	type III secretion system outer membrane ring subunit SctC [*Paracidovorax citrulli*]
fig|80869.152.peg.129	T4S	hypothetical protein [*Paracidovorax citrulli*]
fig|80869.152.peg.247	T4S	XopE/AvrPphe family type III secretion system effector [*Paracidovorax citrulli*]
fig|80869.152.peg.3976	T4S	5'-nucleotidase [*Paracidovorax citrulli*]
fig|80869.152.peg.1123	T4S	hypothetical protein [*Paracidovorax citrulli*]
fig|80869.152.peg.2909	T4S	30S ribosomal protein S4 [*Paracidovorax citrulli*]
fig|80869.152.peg.2968	T4S	adenylate kinase [*Paracidovorax citrulli*]
fig|80869.152.peg.594	T4S	hypothetical protein [*Paracidovorax citrulli*]
fig|80869.152.peg.3271	T4S	hypothetical protein [*Paracidovorax citrulli*]
fig|80869.152.peg.1317	T4S	type IV pilin protein [*Paracidovorax citrulli*]
fig|80869.152.peg.4076	T4S	hypothetical protein [*Paracidovorax citrulli*]
fig|80869.152.peg.2650	T4S	BON domain-containing protein [*Paracidovorax citrulli*]
fig|80869.152.peg.1488	T4S	MetQ/NlpA family ABC transporter substrate-binding protein [*Paracidovorax citrulli*]
fig|80869.152.peg.4206	T4S	Bug family tripartite tricarboxylate transporter substrate binding protein [*Paracidovorax citrulli*]
fig|80869.152.peg.31	T4S	tripartite tricarboxylate transporter substrate binding protein [*Paracidovorax citrulli*]
fig|80869.152.peg.1494	T4S	sulfate ABC transporter substrate-binding protein [*Paracidovorax citrulli*]
fig|80869.152.peg.3762	T4S	hypothetical protein [*Paracidovorax citrulli*]
fig|80869.152.peg.3509	T4S	OmpA family protein [*Paracidovorax citrulli*]
fig|80869.152.peg.111	T4S	endolysin [*Paracidovorax citrulli*]
fig|80869.152.peg.2272	T4S	HTH-type transcriptional regulator GltC [*Paracidovorax citrulli*]
fig|80869.152.peg.1159	T4S	sigma-54 dependent transcriptional regulator [*Paracidovorax citrulli*]
fig|80869.152.peg.3977	T4S	EF-hand domain-containing protein [*Paracidovorax citrulli*]

*P_citrulli*_CCRMAc1.78

Prot	Pred	Description
fig|80869.153.peg.1830	T3S	major facilitator superfamily MFS_1 [*Paracidovorax citrulli* AAC00-1]
fig|80869.153.peg.3069	T3S	Ig domain protein, group 1 domain protein [*Paracidovorax citrulli* AAC00-1]
fig|80869.153.peg.4348	T3S	hypothetical protein [*Paracidovorax citrulli*]
fig|80869.153.peg.2267	T3S	acyl-CoA thioesterase [*Paracidovorax citrulli*]
fig|80869.153.peg.1106	T3S	STY0301 family protein [*Paracidovorax citrulli*]

Prot	Pred	Description
fig|80869.153.peg.1330	T3S	ABC transporter ATP-binding protein [*Paracidovorax citrulli*]
fig|80869.153.peg.1	T3S	DUF72 domain-containing protein [*Paracidovorax citrulli*]
fig|80869.153.peg.1032	T3S	type III secretion system chaperone [*Paracidovorax citrulli*]
fig|80869.153.peg.1033	T3S	hypothetical protein [*Paracidovorax citrulli*]
fig|80869.153.peg.1059	T3S	LLM class flavin-dependent oxidoreductase [*Paracidovorax citrulli*]
fig|80869.153.peg.1072	T3S	hypothetical protein [*Paracidovorax citrulli*]
fig|80869.153.peg.1085	T3S	ABC transporter permease [*Paracidovorax citrulli*]
fig|80869.153.peg.1086	T3S	ABC transporter permease subunit [*Paracidovorax citrulli*]
fig|80869.153.peg.1099	T3S	KGG domain-containing protein [*Paracidovorax citrulli*]
fig|80869.153.peg.1142	T3S	PAS domain S-box protein [*Paracidovorax citrulli*]
fig|80869.153.peg.1175	T3S	EAL domain-containing protein [*Paracidovorax citrulli*]
fig|80869.153.peg.1257	T3S	XopAP family type III secretion system effector [*Paracidovorax citrulli*]
fig|80869.153.peg.1297	T3S	cellulase family glycosylhydrolase [*Paracidovorax citrulli*]
fig|80869.153.peg.1309	T3S	YdiU family protein [*Paracidovorax citrulli*]
fig|80869.153.peg.1387	T3S	BON domain-containing protein [*Paracidovorax citrulli*]
fig|80869.153.peg.1388	T3S	SulP family inorganic anion transporter [*Paracidovorax citrulli*]
fig|80869.153.peg.1396	T3S	FAD-dependent oxidoreductase [*Paracidovorax citrulli*]
fig|80869.153.peg.1398	T3S	tRNA (guanosine(46)-N7)-methyltransferase TrmB [*Paracidovorax citrulli*]
fig|80869.153.peg.1406	T3S	tryptophan 2,3-dioxygenase [*Paracidovorax citrulli*]
fig|80869.153.peg.1457	T3S	Phytochrome-like protein cph2 [*Paracidovorax citrulli*]
fig|80869.153.peg.1458	T3S	lysine--tRNA ligase [*Paracidovorax citrulli*]
fig|80869.153.peg.1465	T3S	LON peptidase substrate-binding domain-containing protein [*Paracidovorax citrulli*]
fig|80869.153.peg.1830	T4S	major facilitator superfamily MFS_1 [*Paracidovorax citrulli* AAC00-1]
fig|80869.153.peg.3069	T4S	Ig domain protein, group 1 domain protein [*Paracidovorax citrulli* AAC00-1]
fig|80869.153.peg.4348	T4S	hypothetical protein [*Paracidovorax citrulli*]
fig|80869.153.peg.2267	T4S	acyl-CoA thioesterase [*Paracidovorax citrulli*]
fig|80869.153.peg.1106	T4S	STY0301 family protein [*Paracidovorax citrulli*]
fig|80869.153.peg.1330	T4S	ABC transporter ATP-binding protein [*Paracidovorax citrulli*]
fig|80869.153.peg.1213	T4S	Ku protein [*Paracidovorax citrulli*]
fig|80869.153.peg.948	T4S	ferritin-like domain-containing protein [*Paracidovorax citrulli*]
fig|80869.153.peg.150	T4S	terminase small subunit [*Paracidovorax citrulli*]
fig|80869.153.peg.1505	T4S	molybdenum cofactor biosynthesis protein MoaE [*Paracidovorax citrulli*]
fig|80869.153.peg.459	T4S	hypothetical protein [*Paracidovorax citrulli*]
fig|80869.153.peg.198	T4S	isoaspartyl peptidase/L-asparaginase [*Paracidovorax citrulli*]
fig|80869.153.peg.4393	T4S	NADAR family protein [Paracandidimonas lactea]
fig|80869.153.peg.3689	T4S	RNA-binding protein [*Paracidovorax citrulli*]
fig|80869.153.peg.3985	T4S	hypothetical protein Aave_3072 [*Paracidovorax citrulli* AAC00-1]
fig|80869.153.peg.3587	T4S	DUF1795 domain-containing protein [*Paracidovorax citrulli*]
fig|80869.153.peg.1245	T4S	amino-acid N-acetyltransferase [*Paracidovorax citrulli*]
fig|80869.153.peg.3311	T4S	DUF924 family protein [*Paracidovorax citrulli*]
fig|80869.153.peg.3930	T4S	50S ribosomal protein L11 [*Paracidovorax citrulli*]
fig|80869.153.peg.1216	T4S	hypothetical protein [*Paracidovorax citrulli*]
fig|80869.153.peg.1805	T4S	hypothetical protein C8E08_4485 [*Paracidovorax citrulli*]
fig|80869.153.peg.4134	T4S	SMC-Scp complex subunit ScpB [*Paracidovorax citrulli*]
fig|80869.153.peg.2779	T4S	PP2C family serine/threonine-protein phosphatase [*Paracidovorax citrulli*]
fig|80869.153.peg.1707	T4S	hypothetical protein [*Paracidovorax citrulli*]

Prot	Pred	Description
fig|80869.153.peg.3951	T4S	cryptochrome/photolyase family protein [*Paracidovorax citrulli*]
fig|80869.153.peg.3027	T4S	phosphopyruvate hydratase [*Paracidovorax citrulli*]
fig|80869.153.peg.1000	T4S	glutathione-regulated potassium-efflux system protein KefC [*Paracidovorax citrulli*]
fig|80869.153.peg.3900	T4S	hypothetical protein [*Paracidovorax citrulli*]
fig|80869.153.peg.563	T4S	hypothetical protein [*Paracidovorax citrulli*]
fig|80869.153.peg.25	T4S	ATP-binding protein [*Paracidovorax citrulli*]
fig|80869.153.peg.3828	T4S	DUF3025 domain-containing protein [*Paracidovorax citrulli*]
fig|80869.153.peg.2780	T4S	serine/threonine-protein kinase [*Paracidovorax citrulli*]
fig|80869.153.peg.4332	T4S	hypothetical protein [*Paracidovorax citrulli*]
fig|80869.153.peg.4049	T4S	inositol monophosphatase family protein [*Paracidovorax citrulli*]
fig|80869.153.peg.838	T4S	N-acetylmuramoyl-L-alanine amidase [*Paracidovorax citrulli*]
fig|80869.153.peg.2725	T4S	AraC family transcriptional regulator [*Paracidovorax citrulli*]
fig|80869.153.peg.622	T4S	hypothetical protein [*Paracidovorax citrulli*]
fig|80869.153.peg.3134	T4S	Fic family protein [*Paracidovorax citrulli*]
fig|80869.153.peg.4314	T4S	4-hydroxy-3-methylbut-2-enyl diphosphate reductase [*Paracidovorax citrulli*]
fig|80869.153.peg.4208	T4S	SsrA-binding protein SmpB [*Paracidovorax citrulli*]
fig|80869.153.peg.2588	T4S	F0F1 ATP synthase subunit gamma [*Paracidovorax citrulli*]
fig|80869.153.peg.365	T4S	hypothetical protein [*Paracidovorax citrulli*]
fig|80869.153.peg.2943	T4S	transglycosylase SLT domain-containing protein [*Paracidovorax citrulli*]
fig|80869.153.peg.2746	T4S	type III secretion system outer membrane ring subunit SctC [*Paracidovorax citrulli*]
fig|80869.153.peg.131	T4S	hypothetical protein [*Paracidovorax citrulli*]
fig|80869.153.peg.248	T4S	XopE/AvrPphe family type III secretion system effector [*Paracidovorax citrulli*]
fig|80869.153.peg.1061	T4S	5'-nucleotidase [*Paracidovorax citrulli*]
fig|80869.153.peg.2690	T4S	30S ribosomal protein S4 [*Paracidovorax citrulli*]
fig|80869.153.peg.2851	T4S	adenylate kinase [*Paracidovorax citrulli*]
fig|80869.153.peg.544	T4S	hypothetical protein [*Paracidovorax citrulli*]
fig|80869.153.peg.3223	T4S	hypothetical protein [*Paracidovorax citrulli*]
fig|80869.153.peg.3926	T4S	type IV pilin protein [*Paracidovorax citrulli*]
fig|80869.153.peg.3848	T4S	hypothetical protein [*Paracidovorax citrulli*]
fig|80869.153.peg.949	T4S	BON domain-containing protein [*Paracidovorax citrulli*]
fig|80869.153.peg.1231	T4S	MetQ/NlpA family ABC transporter substrate-binding protein [*Paracidovorax citrulli*]
fig|80869.153.peg.4175	T4S	Bug family tripartite tricarboxylate transporter substrate binding protein [*Paracidovorax citrulli*]
fig|80869.153.peg.31	T4S	tripartite tricarboxylate transporter substrate binding protein [*Paracidovorax citrulli*]
fig|80869.153.peg.1237	T4S	sulfate ABC transporter substrate-binding protein [*Paracidovorax citrulli*]
fig|80869.153.peg.3756	T4S	hypothetical protein [*Paracidovorax citrulli*]
fig|80869.153.peg.3312	T4S	OmpA family protein [*Paracidovorax citrulli*]
fig|80869.153.peg.113	T4S	endolysin [*Paracidovorax citrulli*]
fig|80869.153.peg.2035	T4S	HTH-type transcriptional regulator GltC [*Paracidovorax citrulli*]
fig|80869.153.peg.2376	T4S	sigma-54 dependent transcriptional regulator [*Paracidovorax citrulli*]
fig|80869.153.peg.1062	T4S	EF-hand domain-containing protein [*Paracidovorax citrulli*]

*P_citrulli*_CCRMAc1.83

Prot	Pred	Description
fig|80869.154.peg.2504	T3S	major facilitator superfamily MFS_1 [*Paracidovorax citrulli* AAC00-1]
fig|80869.154.peg.3256	T3S	Ig domain protein, group 1 domain protein [*Paracidovorax citrulli* AAC00-1]
fig|80869.154.peg.4417	T3S	hypothetical protein [*Paracidovorax citrulli*]
fig|80869.154.peg.3504	T3S	acyl-CoA thioesterase [*Paracidovorax citrulli*]
fig|80869.154.peg.1603	T3S	STY0301 family protein [*Paracidovorax citrulli*]
fig|80869.154.peg.247	T3S	XopE/AvrPphe family type III secretion system effector [*Paracidovorax citrulli*]
fig|80869.154.peg.2172	T3S	ABC transporter ATP-binding protein [*Paracidovorax citrulli*]
fig|80869.154.peg.1	T3S	DUF72 domain-containing protein [*Paracidovorax citrulli*]
fig|80869.154.peg.1006	T3S	Holliday junction resolvase RuvX [*Paracidovorax citrulli*]
fig|80869.154.peg.1015	T3S	bifunctional hydroxymethylpyrimidine kinase/phosphomethylpyrimidine kinase [*Paracidovorax citrulli*]
fig|80869.154.peg.1049	T3S	ribonucleoside-diphosphate reductase subunit alpha [*Paracidovorax citrulli*]
fig|80869.154.peg.1062	T3S	3-deoxy-7-phosphoheptulonate synthase [*Paracidovorax citrulli*]
fig|80869.154.peg.1119	T3S	chromate efflux transporter [*Paracidovorax citrulli*]
fig|80869.154.peg.1126	T3S	M48 family metallopeptidase [*Paracidovorax citrulli*]
fig|80869.154.peg.1142	T3S	DNA-3-methyladenine glycosylase I [*Paracidovorax citrulli*]
fig|80869.154.peg.1143	T3S	aminopeptidase [*Paracidovorax citrulli*]
fig|80869.154.peg.1176	T3S	DUF2169 domain-containing protein [*Paracidovorax citrulli*]
fig|80869.154.peg.1204	T3S	hypothetical protein [*Paracidovorax citrulli*]
fig|80869.154.peg.1216	T3S	YitT family protein [*Paracidovorax citrulli*]
fig|80869.154.peg.1217	T3S	hypothetical protein [*Paracidovorax citrulli*]
fig|80869.154.peg.1232	T3S	3-methyl-2-oxobutanoate hydroxymethyltransferase [*Paracidovorax citrulli*]
fig|80869.154.peg.1254	T3S	signal recognition particle-docking protein FtsY [*Paracidovorax citrulli*]
fig|80869.154.peg.1264	T3S	MATE family efflux transporter [*Paracidovorax citrulli*]
fig|80869.154.peg.1332	T3S	type III secretion system chaperone [*Paracidovorax citrulli*]
fig|80869.154.peg.1333	T3S	hypothetical protein [*Paracidovorax citrulli*]
fig|80869.154.peg.1360	T3S	LLM class flavin-dependent oxidoreductase [*Paracidovorax citrulli*]
fig|80869.154.peg.1373	T3S	hypothetical protein [*Paracidovorax citrulli*]
fig|80869.154.peg.1386	T3S	ABC transporter permease [*Paracidovorax citrulli*]
fig|80869.154.peg.2504	T4S	major facilitator superfamily MFS_1 [*Paracidovorax citrulli* AAC00-1]
fig|80869.154.peg.3256	T4S	Ig domain protein, group 1 domain protein [*Paracidovorax citrulli* AAC00-1]
fig|80869.154.peg.4417	T4S	hypothetical protein [*Paracidovorax citrulli*]
fig|80869.154.peg.3504	T4S	acyl-CoA thioesterase [*Paracidovorax citrulli*]
fig|80869.154.peg.1603	T4S	STY0301 family protein [*Paracidovorax citrulli*]
fig|80869.154.peg.247	T4S	XopE/AvrPphe family type III secretion system effector [*Paracidovorax citrulli*]
fig|80869.154.peg.2172	T4S	ABC transporter ATP-binding protein [*Paracidovorax citrulli*]
fig|80869.154.peg.1710	T4S	Ku protein [*Paracidovorax citrulli*]
fig|80869.154.peg.1247	T4S	ferritin-like domain-containing protein [*Paracidovorax citrulli*]
fig|80869.154.peg.148	T4S	terminase small subunit [*Paracidovorax citrulli*]
fig|80869.154.peg.2074	T4S	molybdenum cofactor biosynthesis protein MoaE [*Paracidovorax citrulli*]
fig|80869.154.peg.462	T4S	hypothetical protein [*Paracidovorax citrulli*]
fig|80869.154.peg.196	T4S	isoaspartyl peptidase/L-asparaginase [*Paracidovorax citrulli*]
fig|80869.154.peg.4460	T4S	NADAR family protein [Paracandidimonas lactea]
fig|80869.154.peg.3543	T4S	RNA-binding protein [*Paracidovorax citrulli*]

Prot	Pred	Description
fig|80869.154.peg.3997	T4S	DUF6479 family protein [*Paracidovorax citrulli*]
fig|80869.154.peg.2938	T4S	DUF1795 domain-containing protein [*Paracidovorax citrulli*]
fig|80869.154.peg.1742	T4S	amino-acid N-acetyltransferase [*Paracidovorax citrulli*]
fig|80869.154.peg.4310	T4S	DUF924 family protein [*Paracidovorax citrulli*]
fig|80869.154.peg.3942	T4S	50S ribosomal protein L11 [*Paracidovorax citrulli*]
fig|80869.154.peg.1713	T4S	hypothetical protein [*Paracidovorax citrulli*]
fig|80869.154.peg.2478	T4S	hypothetical protein C8E08_4485 [*Paracidovorax citrulli*]
fig|80869.154.peg.4189	T4S	SMC-Scp complex subunit ScpB [*Paracidovorax citrulli*]
fig|80869.154.peg.1845	T4S	PP2C family serine/threonine-protein phosphatase [*Paracidovorax citrulli*]
fig|80869.154.peg.2382	T4S	hypothetical protein [*Paracidovorax citrulli*]
fig|80869.154.peg.3963	T4S	cryptochrome/photolyase family protein [*Paracidovorax citrulli*]
fig|80869.154.peg.1996	T4S	phosphopyruvate hydratase [*Paracidovorax citrulli*]
fig|80869.154.peg.1299	T4S	glutathione-regulated potassium-efflux system protein KefC [*Paracidovorax citrulli*]
fig|80869.154.peg.1789	T4S	hypothetical protein [*Paracidovorax citrulli*]
fig|80869.154.peg.566	T4S	hypothetical protein [*Paracidovorax citrulli*]
fig|80869.154.peg.25	T4S	ATP-binding protein [*Paracidovorax citrulli*]
fig|80869.154.peg.3897	T4S	DUF3025 domain-containing protein [*Paracidovorax citrulli*]
fig|80869.154.peg.1846	T4S	serine/threonine-protein kinase [*Paracidovorax citrulli*]
fig|80869.154.peg.4401	T4S	hypothetical protein [*Paracidovorax citrulli*]
fig|80869.154.peg.4060	T4S	inositol monophosphatase family protein [*Paracidovorax citrulli*]
fig|80869.154.peg.895	T4S	N-acetylmuramoyl-L-alanine amidase [*Paracidovorax citrulli*]
fig|80869.154.peg.2913	T4S	AraC family transcriptional regulator [*Paracidovorax citrulli*]
fig|80869.154.peg.625	T4S	hypothetical protein [*Paracidovorax citrulli*]
fig|80869.154.peg.1402	T4S	Fic family protein [*Paracidovorax citrulli*]
fig|80869.154.peg.4383	T4S	4-hydroxy-3-methylbut-2-enyl diphosphate reductase [*Paracidovorax citrulli*]
fig|80869.154.peg.4263	T4S	SsrA-binding protein SmpB [*Paracidovorax citrulli*]
fig|80869.154.peg.760	T4S	hypothetical protein [*Paracidovorax citrulli*]
fig|80869.154.peg.2777	T4S	F0F1 ATP synthase subunit gamma [*Paracidovorax citrulli*]
fig|80869.154.peg.369	T4S	hypothetical protein [*Paracidovorax citrulli*]
fig|80869.154.peg.3124	T4S	transglycosylase SLT domain-containing protein [*Paracidovorax citrulli*]
fig|80869.154.peg.2933	T4S	type III secretion system outer membrane ring subunit SctC [*Paracidovorax citrulli*]
fig|80869.154.peg.129	T4S	hypothetical protein [*Paracidovorax citrulli*]
fig|80869.154.peg.1362	T4S	5'-nucleotidase [*Paracidovorax citrulli*]
fig|80869.154.peg.2878	T4S	30S ribosomal protein S4 [*Paracidovorax citrulli*]
fig|80869.154.peg.3032	T4S	adenylate kinase [*Paracidovorax citrulli*]
fig|80869.154.peg.547	T4S	hypothetical protein [*Paracidovorax citrulli*]
fig|80869.154.peg.3426	T4S	hypothetical protein [*Paracidovorax citrulli*]
fig|80869.154.peg.1815	T4S	type IV pilin protein [*Paracidovorax citrulli*]
fig|80869.154.peg.1248	T4S	BON domain-containing protein [*Paracidovorax citrulli*]
fig|80869.154.peg.1728	T4S	MetQ/NlpA family ABC transporter substrate-binding protein [*Paracidovorax citrulli*]
fig|80869.154.peg.4230	T4S	Bug family tripartite tricarboxylate transporter substrate binding protein [*Paracidovorax citrulli*]
fig|80869.154.peg.31	T4S	tripartite tricarboxylate transporter substrate binding protein [*Paracidovorax citrulli*]
fig|80869.154.peg.1734	T4S	sulfate ABC transporter substrate-binding protein [*Paracidovorax citrulli*]
fig|80869.154.peg.3825	T4S	hypothetical protein [*Paracidovorax citrulli*]

Prot	Pred	Description
fig|80869.154.peg.4311	T4S	OmpA family protein [*Paracidovorax citrulli*]
fig|80869.154.peg.111	T4S	endolysin [*Paracidovorax citrulli*]
fig|80869.154.peg.2593	T4S	HTH-type transcriptional regulator GltC [*Paracidovorax citrulli*]
fig|80869.154.peg.3133	T4S	sigma-54 dependent transcriptional regulator [*Paracidovorax citrulli*]
fig|80869.154.peg.1363	T4S	EF-hand domain-containing protein [*Paracidovorax citrulli*]

*P_citrulli_*CCRMAc5.1

Prot	Pred	Description
fig|80869.155.peg.2542	T3S	major facilitator superfamily MFS_1 [*Paracidovorax citrulli* AAC00-1]
fig|80869.155.peg.3176	T3S	Ig domain protein, group 1 domain protein [*Paracidovorax citrulli* AAC00-1]
fig|80869.155.peg.4328	T3S	hypothetical protein [*Paracidovorax citrulli*]
fig|80869.155.peg.3334	T3S	acyl-CoA thioesterase [*Paracidovorax citrulli*]
fig|80869.155.peg.2316	T3S	STY0301 family protein [*Paracidovorax citrulli*]
fig|80869.155.peg.2070	T3S	ABC transporter ATP-binding protein [*Paracidovorax citrulli*]
fig|80869.155.peg.1	T3S	peptidoglycan-binding domain-containing protein, partial [*Paracidovorax citrulli*]
fig|80869.155.peg.1016	T3S	alpha/beta hydrolase [*Paracidovorax citrulli*]
fig|80869.155.peg.1045	T3S	septal ring lytic transglycosylase RlpA family protein [*Paracidovorax citrulli*]
fig|80869.155.peg.1058	T3S	hypothetical protein [*Paracidovorax citrulli*]
fig|80869.155.peg.1065	T3S	TRAP transporter small permease [Paracidovorax]
fig|80869.155.peg.1117	T3S	rhodanese-like domain-containing protein [*Paracidovorax citrulli*]
fig|80869.155.peg.113	T3S	ribonucleoside-diphosphate reductase subunit alpha [*Paracidovorax citrulli*]
fig|80869.155.peg.1134	T3S	GNAT family N-acetyltransferase [*Paracidovorax citrulli*]
fig|80869.155.peg.1147	T3S	ABC transporter permease [*Paracidovorax citrulli*]
fig|80869.155.peg.1151	T3S	hypothetical protein [*Paracidovorax citrulli*]
fig|80869.155.peg.1152	T3S	Metallo-beta-lactamase superfamily protein [*Paracidovorax citrulli*]
fig|80869.155.peg.1164	T3S	Trehalase [*Paracidovorax citrulli*]
fig|80869.155.peg.1184	T3S	hypothetical protein [*Paracidovorax citrulli*]
fig|80869.155.peg.1212	T3S	ABC transporter permease [*Paracidovorax citrulli*]
fig|80869.155.peg.125	T3S	3-deoxy-7-phosphoheptulonate synthase [*Paracidovorax citrulli*]
fig|80869.155.peg.1267	T3S	hypothetical protein [*Paracidovorax citrulli*]
fig|80869.155.peg.1279	T3S	YitT family protein [*Paracidovorax citrulli*]
fig|80869.155.peg.1280	T3S	hypothetical protein [*Paracidovorax citrulli*]
fig|80869.155.peg.1295	T3S	3-methyl-2-oxobutanoate hydroxymethyltransferase [*Paracidovorax citrulli*]
fig|80869.155.peg.1316	T3S	signal recognition particle-docking protein FtsY [*Paracidovorax citrulli*]
fig|80869.155.peg.1326	T3S	MATE family efflux transporter [*Paracidovorax citrulli*]
fig|80869.155.peg.1395	T3S	type III secretion system chaperone [*Paracidovorax citrulli*]
fig|80869.155.peg.2542	T4S	major facilitator superfamily MFS_1 [*Paracidovorax citrulli* AAC00-1]
fig|80869.155.peg.3176	T4S	Ig domain protein, group 1 domain protein [*Paracidovorax citrulli* AAC00-1]
fig|80869.155.peg.4328	T4S	hypothetical protein [*Paracidovorax citrulli*]
fig|80869.155.peg.3334	T4S	acyl-CoA thioesterase [*Paracidovorax citrulli*]
fig|80869.155.peg.2316	T4S	STY0301 family protein [*Paracidovorax citrulli*]
fig|80869.155.peg.2070	T4S	ABC transporter ATP-binding protein [*Paracidovorax citrulli*]
fig|80869.155.peg.2423	T4S	Ku protein [*Paracidovorax citrulli*]

Prot	Pred	Description
fig|80869.155.peg.1309	T4S	ferritin-like domain-containing protein [*Paracidovorax citrulli*]
fig|80869.155.peg.507	T4S	terminase small subunit [*Paracidovorax citrulli*]
fig|80869.155.peg.1979	T4S	molybdenum cofactor biosynthesis protein MoaE [*Paracidovorax citrulli*]
fig|80869.155.peg.820	T4S	hypothetical protein [*Paracidovorax citrulli*]
fig|80869.155.peg.556	T4S	isoaspartyl peptidase/L-asparaginase [*Paracidovorax citrulli*]
fig|80869.155.peg.4373	T4S	NADAR family protein [Paracandidimonas lactea]
fig|80869.155.peg.3373	T4S	RNA-binding protein [*Paracidovorax citrulli*]
fig|80869.155.peg.3888	T4S	hypothetical protein Aave_3072 [*Paracidovorax citrulli* AAC00-1]
fig|80869.155.peg.3054	T4S	DUF1795 domain-containing protein [*Paracidovorax citrulli*]
fig|80869.155.peg.4400	T4S	amino-acid N-acetyltransferase [*Paracidovorax citrulli*]
fig|80869.155.peg.4199	T4S	DUF924 family protein [*Paracidovorax citrulli*]
fig|80869.155.peg.3782	T4S	50S ribosomal protein L11 [*Paracidovorax citrulli*]
fig|80869.155.peg.2426	T4S	hypothetical protein [*Paracidovorax citrulli*]
fig|80869.155.peg.2516	T4S	hypothetical protein C8E08_4485 [*Paracidovorax citrulli*]
fig|80869.155.peg.4078	T4S	SMC-Scp complex subunit ScpB [*Paracidovorax citrulli*]
fig|80869.155.peg.2981	T4S	PP2C family serine/threonine-protein phosphatase [*Paracidovorax citrulli*]
fig|80869.155.peg.2280	T4S	hypothetical protein [*Paracidovorax citrulli*]
fig|80869.155.peg.3803	T4S	cryptochrome/photolyase family protein [*Paracidovorax citrulli*]
fig|80869.155.peg.1901	T4S	phosphopyruvate hydratase [*Paracidovorax citrulli*]
fig|80869.155.peg.1362	T4S	glutathione-regulated potassium-efflux system protein KefC [*Paracidovorax citrulli*]
fig|80869.155.peg.1793	T4S	hypothetical protein [*Paracidovorax citrulli*]
fig|80869.155.peg.925	T4S	hypothetical protein [*Paracidovorax citrulli*]
fig|80869.155.peg.384	T4S	ATP-binding protein [*Paracidovorax citrulli*]
fig|80869.155.peg.3736	T4S	DUF3025 domain-containing protein [*Paracidovorax citrulli*]
fig|80869.155.peg.2982	T4S	serine/threonine-protein kinase [*Paracidovorax citrulli*]
fig|80869.155.peg.4290	T4S	hypothetical protein [*Paracidovorax citrulli*]
fig|80869.155.peg.3950	T4S	inositol monophosphatase family protein [*Paracidovorax citrulli*]
fig|80869.155.peg.1199	T4S	N-acetylmuramoyl-L-alanine amidase [*Paracidovorax citrulli*]
fig|80869.155.peg.335	T4S	AraC family transcriptional regulator [*Paracidovorax citrulli*]
fig|80869.155.peg.983	T4S	hypothetical protein [*Paracidovorax citrulli*]
fig|80869.155.peg.1466	T4S	Fic family protein [*Paracidovorax citrulli*]
fig|80869.155.peg.4272	T4S	4-hydroxy-3-methylbut-2-enyl diphosphate reductase [*Paracidovorax citrulli*]
fig|80869.155.peg.4152	T4S	SsrA-binding protein SmpB [*Paracidovorax citrulli*]
fig|80869.155.peg.2811	T4S	F0F1 ATP synthase subunit gamma [*Paracidovorax citrulli*]
fig|80869.155.peg.725	T4S	hypothetical protein [*Paracidovorax citrulli*]
fig|80869.155.peg.2943	T4S	transglycosylase SLT domain-containing protein [*Paracidovorax citrulli*]
fig|80869.155.peg.355	T4S	type III secretion system outer membrane ring subunit SctC [*Paracidovorax citrulli*]
fig|80869.155.peg.488	T4S	hypothetical protein [*Paracidovorax citrulli*]
fig|80869.155.peg.607	T4S	XopE/AvrPphe family type III secretion system effector [*Paracidovorax citrulli*]
fig|80869.155.peg.1425	T4S	5'-nucleotidase [*Paracidovorax citrulli*]
fig|80869.155.peg.300	T4S	30S ribosomal protein S4 [*Paracidovorax citrulli*]
fig|80869.155.peg.2850	T4S	adenylate kinase [*Paracidovorax citrulli*]
fig|80869.155.peg.906	T4S	hypothetical protein [*Paracidovorax citrulli*]
fig|80869.155.peg.3258	T4S	hypothetical protein [*Paracidovorax citrulli*]
fig|80869.155.peg.1819	T4S	type IV pilin protein [*Paracidovorax citrulli*]

Prot	Pred	Description
fig|80869.155.peg.1310	T4S	BON domain-containing protein [*Paracidovorax citrulli*]
fig|80869.155.peg.2443	T4S	MetQ/NlpA family ABC transporter substrate-binding protein [*Paracidovorax citrulli*]
fig|80869.155.peg.4119	T4S	Bug family tripartite tricarboxylate transporter substrate binding protein [*Paracidovorax citrulli*]
fig|80869.155.peg.390	T4S	tripartite tricarboxylate transporter substrate binding protein [*Paracidovorax citrulli*]
fig|80869.155.peg.4392	T4S	sulfate ABC transporter substrate-binding protein [*Paracidovorax citrulli*]
fig|80869.155.peg.3664	T4S	hypothetical protein [*Paracidovorax citrulli*]
fig|80869.155.peg.4200	T4S	OmpA family protein [*Paracidovorax citrulli*]
fig|80869.155.peg.470	T4S	endolysin [*Paracidovorax citrulli*]
fig|80869.155.peg.2629	T4S	HTH-type transcriptional regulator GltC [*Paracidovorax citrulli*]
fig|80869.155.peg.1662	T4S	sigma-54 dependent transcriptional regulator [*Paracidovorax citrulli*]
fig|80869.155.peg.1426	T4S	EF-hand domain-containing protein [*Paracidovorax citrulli*]

*P_citrulli_*CCRMAc5.3

Prot	Pred	Description
fig|80869.156.peg.2595	T3S	major facilitator superfamily MFS_1 [*Paracidovorax citrulli* AAC00-1]
fig|80869.156.peg.3255	T3S	Ig domain protein, group 1 domain protein [*Paracidovorax citrulli* AAC00-1]
fig|80869.156.peg.4432	T3S	hypothetical protein [*Paracidovorax citrulli*]
fig|80869.156.peg.3504	T3S	acyl-CoA thioesterase [*Paracidovorax citrulli*]
fig|80869.156.peg.1860	T3S	STY0301 family protein [*Paracidovorax citrulli*]
fig|80869.156.peg.2263	T3S	ABC transporter ATP-binding protein [*Paracidovorax citrulli*]
fig|80869.156.peg.1	T3S	DUF72 domain-containing protein [*Paracidovorax citrulli*]
fig|80869.156.peg.1000	T3S	dipeptide ABC transporter ATP-binding protein [*Paracidovorax citrulli*]
fig|80869.156.peg.1033	T3S	Holliday junction resolvase RuvX [*Paracidovorax citrulli*]
fig|80869.156.peg.1042	T3S	bifunctional hydroxymethylpyrimidine kinase/phosphomethylpyrimidine kinase [*Paracidovorax citrulli*]
fig|80869.156.peg.1076	T3S	ribonucleoside-diphosphate reductase subunit alpha [*Paracidovorax citrulli*]
fig|80869.156.peg.1090	T3S	3-deoxy-7-phosphoheptulonate synthase [*Paracidovorax citrulli*]
fig|80869.156.peg.1136	T3S	GTP-binding protein [*Paracidovorax citrulli*]
fig|80869.156.peg.1147	T3S	chromate efflux transporter [*Paracidovorax citrulli*]
fig|80869.156.peg.1154	T3S	M48 family metallopeptidase [*Paracidovorax citrulli*]
fig|80869.156.peg.1169	T3S	DNA-3-methyladenine glycosylase I [*Paracidovorax citrulli*]
fig|80869.156.peg.1170	T3S	putative zinc protease protein [*Paracidovorax citrulli* AAC00-1]
fig|80869.156.peg.1201	T3S	DUF2169 domain-containing protein [*Paracidovorax citrulli*]
fig|80869.156.peg.1210	T3S	alpha/beta hydrolase [*Paracidovorax citrulli*]
fig|80869.156.peg.1239	T3S	septal ring lytic transglycosylase RlpA family protein [*Paracidovorax citrulli*]
fig|80869.156.peg.1252	T3S	hypothetical protein [*Paracidovorax citrulli*]
fig|80869.156.peg.1259	T3S	TRAP transporter small permease [Paracidovorax]
fig|80869.156.peg.1311	T3S	rhodanese-like domain-containing protein [*Paracidovorax citrulli*]
fig|80869.156.peg.1328	T3S	GNAT family N-acetyltransferase [*Paracidovorax citrulli*]
fig|80869.156.peg.1341	T3S	ABC transporter permease [*Paracidovorax citrulli*]
fig|80869.156.peg.1345	T3S	hypothetical protein [*Paracidovorax citrulli*]
fig|80869.156.peg.1346	T3S	Metallo-beta-lactamase superfamily protein [*Paracidovorax citrulli*]
fig|80869.156.peg.2595	T4S	major facilitator superfamily MFS_1 [*Paracidovorax citrulli* AAC00-1]
fig|80869.156.peg.3255	T4S	Ig domain protein, group 1 domain protein [*Paracidovorax citrulli* AAC00-1]

Prot	Pred	Description
fig|80869.156.peg.4432	T4S	hypothetical protein [*Paracidovorax citrulli*]
fig|80869.156.peg.3504	T4S	acyl-CoA thioesterase [*Paracidovorax citrulli*]
fig|80869.156.peg.1860	T4S	STY0301 family protein [*Paracidovorax citrulli*]
fig|80869.156.peg.2263	T4S	ABC transporter ATP-binding protein [*Paracidovorax citrulli*]
fig|80869.156.peg.1966	T4S	Ku protein [*Paracidovorax citrulli*]
fig|80869.156.peg.1504	T4S	ferritin-like domain-containing protein [*Paracidovorax citrulli*]
fig|80869.156.peg.148	T4S	terminase small subunit [*Paracidovorax citrulli*]
fig|80869.156.peg.2165	T4S	molybdenum cofactor biosynthesis protein MoaE [*Paracidovorax citrulli*]
fig|80869.156.peg.509	T4S	hypothetical protein [*Paracidovorax citrulli*]
fig|80869.156.peg.196	T4S	isoaspartyl peptidase/L-asparaginase [*Paracidovorax citrulli*]
fig|80869.156.peg.4474	T4S	NADAR family protein [Paracandidimonas lactea]
fig|80869.156.peg.3543	T4S	RNA-binding protein [*Paracidovorax citrulli*]
fig|80869.156.peg.413	T4S	hypothetical protein Aave_3072 [*Paracidovorax citrulli* AAC00-1]
fig|80869.156.peg.3132	T4S	DUF1795 domain-containing protein [*Paracidovorax citrulli*]
fig|80869.156.peg.1999	T4S	amino-acid N-acetyltransferase [*Paracidovorax citrulli*]
fig|80869.156.peg.4325	T4S	DUF924 family protein [*Paracidovorax citrulli*]
fig|80869.156.peg.4007	T4S	50S ribosomal protein L11 [*Paracidovorax citrulli*]
fig|80869.156.peg.1969	T4S	hypothetical protein [*Paracidovorax citrulli*]
fig|80869.156.peg.2569	T4S	hypothetical protein C8E08_4485 [*Paracidovorax citrulli*]
fig|80869.156.peg.4204	T4S	SMC-Scp complex subunit ScpB [*Paracidovorax citrulli*]
fig|80869.156.peg.892	T4S	PP2C family serine/threonine-protein phosphatase [*Paracidovorax citrulli*]
fig|80869.156.peg.2473	T4S	hypothetical protein [*Paracidovorax citrulli*]
fig|80869.156.peg.4028	T4S	cryptochrome/photolyase family protein [*Paracidovorax citrulli*]
fig|80869.156.peg.2087	T4S	phosphopyruvate hydratase [*Paracidovorax citrulli*]
fig|80869.156.peg.1556	T4S	glutathione-regulated potassium-efflux system protein KefC [*Paracidovorax citrulli*]
fig|80869.156.peg.836	T4S	hypothetical protein [*Paracidovorax citrulli*]
fig|80869.156.peg.613	T4S	hypothetical protein [*Paracidovorax citrulli*]
fig|80869.156.peg.25	T4S	ATP-binding protein [*Paracidovorax citrulli*]
fig|80869.156.peg.3895	T4S	DUF3025 domain-containing protein [*Paracidovorax citrulli*]
fig|80869.156.peg.893	T4S	serine/threonine-protein kinase [*Paracidovorax citrulli*]
fig|80869.156.peg.4417	T4S	hypothetical protein [*Paracidovorax citrulli*]
fig|80869.156.peg.4075	T4S	inositol monophosphatase family protein [*Paracidovorax citrulli*]
fig|80869.156.peg.1393	T4S	N-acetylmuramoyl-L-alanine amidase [*Paracidovorax citrulli*]
fig|80869.156.peg.3005	T4S	AraC family transcriptional regulator [*Paracidovorax citrulli*]
fig|80869.156.peg.673	T4S	hypothetical protein [*Paracidovorax citrulli*]
fig|80869.156.peg.1658	T4S	Fic family protein [*Paracidovorax citrulli*]
fig|80869.156.peg.4399	T4S	4-hydroxy-3-methylbut-2-enyl diphosphate reductase [*Paracidovorax citrulli*]
fig|80869.156.peg.4278	T4S	SsrA-binding protein SmpB [*Paracidovorax citrulli*]
fig|80869.156.peg.2868	T4S	F0F1 ATP synthase subunit gamma [*Paracidovorax citrulli*]
fig|80869.156.peg.367	T4S	hypothetical protein [*Paracidovorax citrulli*]
fig|80869.156.peg.3122	T4S	transglycosylase SLT domain-containing protein [*Paracidovorax citrulli*]
fig|80869.156.peg.3026	T4S	type III secretion system outer membrane ring subunit SctC [*Paracidovorax citrulli*]
fig|80869.156.peg.129	T4S	hypothetical protein [*Paracidovorax citrulli*]
fig|80869.156.peg.248	T4S	XopE/AvrPphe family type III secretion system effector [*Paracidovorax citrulli*]
fig|80869.156.peg.1618	T4S	5'-nucleotidase [*Paracidovorax citrulli*]

Prot	Pred	Description
fig|80869.156.peg.3974	T4S	hypothetical protein [*Paracidovorax citrulli*]
fig|80869.156.peg.2970	T4S	30S ribosomal protein S4 [*Paracidovorax citrulli*]
fig|80869.156.peg.3030	T4S	adenylate kinase [*Paracidovorax citrulli*]
fig|80869.156.peg.594	T4S	hypothetical protein [*Paracidovorax citrulli*]
fig|80869.156.peg.3426	T4S	hypothetical protein [*Paracidovorax citrulli*]
fig|80869.156.peg.862	T4S	type IV pilin protein [*Paracidovorax citrulli*]
fig|80869.156.peg.1505	T4S	BON domain-containing protein [*Paracidovorax citrulli*]
fig|80869.156.peg.1985	T4S	MetQ/NlpA family ABC transporter substrate-binding protein [*Paracidovorax citrulli*]
fig|80869.156.peg.4245	T4S	Bug family tripartite tricarboxylate transporter substrate binding protein [*Paracidovorax citrulli*]
fig|80869.156.peg.31	T4S	tripartite tricarboxylate transporter substrate binding protein [*Paracidovorax citrulli*]
fig|80869.156.peg.1991	T4S	sulfate ABC transporter substrate-binding protein [*Paracidovorax citrulli*]
fig|80869.156.peg.3822	T4S	hypothetical protein [*Paracidovorax citrulli*]
fig|80869.156.peg.4326	T4S	OmpA family protein [*Paracidovorax citrulli*]
fig|80869.156.peg.111	T4S	endolysin [*Paracidovorax citrulli*]
fig|80869.156.peg.2684	T4S	HTH-type transcriptional regulator GltC [*Paracidovorax citrulli*]
fig|80869.156.peg.705	T4S	sigma-54 dependent transcriptional regulator [*Paracidovorax citrulli*]
fig|80869.156.peg.1619	T4S	EF-hand domain-containing protein [*Paracidovorax citrulli*]

*P_citrulli*_CCRMAc8

Prot	Pred	Description
fig|80869.158.peg.2434	T3S	major facilitator superfamily MFS_1 [*Paracidovorax citrulli* AAC00-1]
fig|80869.158.peg.3209	T3S	Ig domain protein, group 1 domain protein [*Paracidovorax citrulli* AAC00-1]
fig|80869.158.peg.4389	T3S	hypothetical protein [*Paracidovorax citrulli*]
fig|80869.158.peg.3367	T3S	acyl-CoA thioesterase [*Paracidovorax citrulli*]
fig|80869.158.peg.2757	T3S	STY0301 family protein [*Paracidovorax citrulli*]
fig|80869.158.peg.1936	T3S	ABC transporter ATP-binding protein [*Paracidovorax citrulli*]
fig|80869.158.peg.1008	T3S	CaiB/BaiF CoA-transferase family protein [*Paracidovorax citrulli*]
fig|80869.158.peg.1026	T3S	alpha/beta hydrolase [*Paracidovorax citrulli*]
fig|80869.158.peg.1056	T3S	septal ring lytic transglycosylase RlpA family protein [*Paracidovorax citrulli*]
fig|80869.158.peg.1069	T3S	hypothetical protein [*Paracidovorax citrulli*]
fig|80869.158.peg.1076	T3S	TRAP transporter small permease [Paracidovorax]
fig|80869.158.peg.1128	T3S	rhodanese-like domain-containing protein [*Paracidovorax citrulli*]
fig|80869.158.peg.1145	T3S	GNAT family N-acetyltransferase [*Paracidovorax citrulli*]
fig|80869.158.peg.1158	T3S	ABC transporter permease [*Paracidovorax citrulli*]
fig|80869.158.peg.1163	T3S	Metallo-beta-lactamase superfamily protein [*Paracidovorax citrulli*]
fig|80869.158.peg.1175	T3S	Trehalase [*Paracidovorax citrulli*]
fig|80869.158.peg.1195	T3S	hypothetical protein [*Paracidovorax citrulli*]
fig|80869.158.peg.12	T3S	ATP-binding protein [*Paracidovorax citrulli*]
fig|80869.158.peg.1223	T3S	ABC transporter permease [*Paracidovorax citrulli*]
fig|80869.158.peg.1278	T3S	hypothetical protein [*Paracidovorax citrulli*]
fig|80869.158.peg.1290	T3S	YitT family protein [*Paracidovorax citrulli*]
fig|80869.158.peg.1291	T3S	hypothetical protein [*Paracidovorax citrulli*]
fig|80869.158.peg.1306	T3S	3-methyl-2-oxobutanoate hydroxymethyltransferase [*Paracidovorax citrulli*]

Prot	Pred	Description
fig|80869.158.peg.1327	T3S	signal recognition particle-docking protein FtsY [*Paracidovorax citrulli*]
fig|80869.158.peg.1337	T3S	MATE family efflux transporter [*Paracidovorax citrulli*]
fig|80869.158.peg.1404	T3S	type III secretion system chaperone [*Paracidovorax citrulli*]
fig|80869.158.peg.1405	T3S	hypothetical protein [*Paracidovorax citrulli*]
fig|80869.158.peg.1431	T3S	LLM class flavin-dependent oxidoreductase [*Paracidovorax citrulli*]
fig|80869.158.peg.2434	T4S	major facilitator superfamily MFS_1 [*Paracidovorax citrulli* AAC00-1]
fig|80869.158.peg.3209	T4S	Ig domain protein, group 1 domain protein [*Paracidovorax citrulli* AAC00-1]
fig|80869.158.peg.4389	T4S	hypothetical protein [*Paracidovorax citrulli*]
fig|80869.158.peg.3367	T4S	acyl-CoA thioesterase [*Paracidovorax citrulli*]
fig|80869.158.peg.2757	T4S	STY0301 family protein [*Paracidovorax citrulli*]
fig|80869.158.peg.1936	T4S	ABC transporter ATP-binding protein [*Paracidovorax citrulli*]
fig|80869.158.peg.3817	T4S	Ku protein [*Paracidovorax citrulli*]
fig|80869.158.peg.1320	T4S	ferritin-like domain-containing protein [*Paracidovorax citrulli*]
fig|80869.158.peg.2155	T4S	terminase small subunit [*Paracidovorax citrulli*]
fig|80869.158.peg.859	T4S	molybdenum cofactor biosynthesis protein MoaE [*Paracidovorax citrulli*]
fig|80869.158.peg.86	T4S	hypothetical protein [*Paracidovorax citrulli*]
fig|80869.158.peg.1684	T4S	isoaspartyl peptidase/L-asparaginase [*Paracidovorax citrulli*]
fig|80869.158.peg.3406	T4S	RNA-binding protein [*Paracidovorax citrulli*]
fig|80869.158.peg.3915	T4S	hypothetical protein Aave_3072 [*Paracidovorax citrulli* AAC00-1]
fig|80869.158.peg.3087	T4S	DUF1795 domain-containing protein [*Paracidovorax citrulli*]
fig|80869.158.peg.3850	T4S	amino-acid N-acetyltransferase [*Paracidovorax citrulli*]
fig|80869.158.peg.4282	T4S	DUF924 family protein [*Paracidovorax citrulli*]
fig|80869.158.peg.1613	T4S	hypothetical protein [*Paracidovorax citrulli*]
fig|80869.158.peg.3860	T4S	50S ribosomal protein L11 [*Paracidovorax citrulli*]
fig|80869.158.peg.3820	T4S	hypothetical protein [*Paracidovorax citrulli*]
fig|80869.158.peg.2409	T4S	hypothetical protein C8E08_4485 [*Paracidovorax citrulli*]
fig|80869.158.peg.4196	T4S	SMC-Scp complex subunit ScpB [*Paracidovorax citrulli*]
fig|80869.158.peg.468	T4S	PP2C family serine/threonine-protein phosphatase [*Paracidovorax citrulli*]
fig|80869.158.peg.2313	T4S	hypothetical protein [*Paracidovorax citrulli*]
fig|80869.158.peg.3881	T4S	cryptochrome/photolyase family protein [*Paracidovorax citrulli*]
fig|80869.158.peg.937	T4S	phosphopyruvate hydratase [*Paracidovorax citrulli*]
fig|80869.158.peg.1372	T4S	glutathione-regulated potassium-efflux system protein KefC [*Paracidovorax citrulli*]
fig|80869.158.peg.411	T4S	hypothetical protein [*Paracidovorax citrulli*]
fig|80869.158.peg.190	T4S	hypothetical protein [*Paracidovorax citrulli*]
fig|80869.158.peg.2035	T4S	ATP-binding protein [*Paracidovorax citrulli*]
fig|80869.158.peg.4108	T4S	DUF3025 domain-containing protein [*Paracidovorax citrulli*]
fig|80869.158.peg.469	T4S	serine/threonine-protein kinase [*Paracidovorax citrulli*]
fig|80869.158.peg.4373	T4S	hypothetical protein [*Paracidovorax citrulli*]
fig|80869.158.peg.3977	T4S	inositol monophosphatase family protein [*Paracidovorax citrulli*]
fig|80869.158.peg.1210	T4S	N-acetylmuramoyl-L-alanine amidase [*Paracidovorax citrulli*]
fig|80869.158.peg.2957	T4S	AraC family transcriptional regulator [*Paracidovorax citrulli*]
fig|80869.158.peg.247	T4S	hypothetical protein [*Paracidovorax citrulli*]
fig|80869.158.peg.1470	T4S	Fic family protein [*Paracidovorax citrulli*]
fig|80869.158.peg.4355	T4S	4-hydroxy-3-methylbut-2-enyl diphosphate reductase [*Paracidovorax citrulli*]
fig|80869.158.peg.4158	T4S	SsrA-binding protein SmpB [*Paracidovorax citrulli*]

Prot	Pred	Description
fig|80869.158.peg.2706	T4S	F0F1 ATP synthase subunit gamma [*Paracidovorax citrulli*]
fig|80869.158.peg.1852	T4S	hypothetical protein [*Paracidovorax citrulli*]
fig|80869.158.peg.3072	T4S	transglycosylase SLT domain-containing protein [*Paracidovorax citrulli*]
fig|80869.158.peg.2977	T4S	type III secretion system outer membrane ring subunit SctC [*Paracidovorax citrulli*]
fig|80869.158.peg.2136	T4S	hypothetical protein [*Paracidovorax citrulli*]
fig|80869.158.peg.1734	T4S	XopE/AvrPphe family type III secretion system effector [*Paracidovorax citrulli*]
fig|80869.158.peg.3289	T4S	hypothetical protein [*Paracidovorax citrulli*]
fig|80869.158.peg.1433	T4S	5'-nucleotidase [*Paracidovorax citrulli*]
fig|80869.158.peg.2922	T4S	30S ribosomal protein S4 [*Paracidovorax citrulli*]
fig|80869.158.peg.2981	T4S	adenylate kinase [*Paracidovorax citrulli*]
fig|80869.158.peg.171	T4S	hypothetical protein [*Paracidovorax citrulli*]
fig|80869.158.peg.437	T4S	type IV pilin protein [*Paracidovorax citrulli*]
fig|80869.158.peg.4128	T4S	hypothetical protein [*Paracidovorax citrulli*]
fig|80869.158.peg.1321	T4S	BON domain-containing protein [*Paracidovorax citrulli*]
fig|80869.158.peg.3836	T4S	MetQ/NlpA family ABC transporter substrate-binding protein [*Paracidovorax citrulli*]
fig|80869.158.peg.4239	T4S	Bug family tripartite tricarboxylate transporter substrate binding protein [*Paracidovorax citrulli*]
fig|80869.158.peg.2041	T4S	tripartite tricarboxylate transporter substrate binding protein [*Paracidovorax citrulli*]
fig|80869.158.peg.3842	T4S	sulfate ABC transporter substrate-binding protein [*Paracidovorax citrulli*]
fig|80869.158.peg.3695	T4S	hypothetical protein [*Paracidovorax citrulli*]
fig|80869.158.peg.4283	T4S	OmpA family protein [*Paracidovorax citrulli*]
fig|80869.158.peg.2118	T4S	endolysin [*Paracidovorax citrulli*]
fig|80869.158.peg.2524	T4S	HTH-type transcriptional regulator GltC [*Paracidovorax citrulli*]
fig|80869.158.peg.279	T4S	sigma-54 dependent transcriptional regulator [*Paracidovorax citrulli*]
fig|80869.158.peg.1434	T4S	EF-hand domain-containing protein [*Paracidovorax citrulli*]

*P_citrulli_*CCRMAc9

Prot	Pred	Description
fig|80869.159.peg.2465	T3S	major facilitator superfamily MFS_1 [*Paracidovorax citrulli* AAC00-1]
fig|80869.159.peg.3239	T3S	Ig domain protein, group 1 domain protein [*Paracidovorax citrulli* AAC00-1]
fig|80869.159.peg.4395	T3S	hypothetical protein [*Paracidovorax citrulli*]
fig|80869.159.peg.3397	T3S	acyl-CoA thioesterase [*Paracidovorax citrulli*]
fig|80869.159.peg.2789	T3S	STY0301 family protein [*Paracidovorax citrulli*]
fig|80869.159.peg.1982	T3S	ABC transporter ATP-binding protein [*Paracidovorax citrulli*]
fig|80869.159.peg.1006	T3S	pyridoxal kinase PdxY [*Paracidovorax citrulli*]
fig|80869.159.peg.1012	T3S	DNA internalization-related competence protein ComEC/Rec2 [*Paracidovorax citrulli*]
fig|80869.159.peg.1020	T3S	VF530 family protein [*Paracidovorax citrulli*]
fig|80869.159.peg.104	T3S	TetR/AcrR family transcriptional regulator [*Paracidovorax citrulli*]
fig|80869.159.peg.1053	T3S	CaiB/BaiF CoA-transferase family protein [*Paracidovorax citrulli*]
fig|80869.159.peg.1071	T3S	alpha/beta hydrolase [*Paracidovorax citrulli*]
fig|80869.159.peg.1100	T3S	septal ring lytic transglycosylase RlpA family protein [*Paracidovorax citrulli*]
fig|80869.159.peg.1113	T3S	hypothetical protein [*Paracidovorax citrulli*]

Prot	Pred	Description
fig|80869.159.peg.1120	T3S	TRAP transporter small permease [Paracidovorax]
fig|80869.159.peg.1172	T3S	rhodanese-like domain-containing protein [*Paracidovorax citrulli*]
fig|80869.159.peg.1189	T3S	GNAT family N-acetyltransferase [*Paracidovorax citrulli*]
fig|80869.159.peg.1202	T3S	ABC transporter permease [*Paracidovorax citrulli*]
fig|80869.159.peg.1206	T3S	hypothetical protein [*Paracidovorax citrulli*]
fig|80869.159.peg.1207	T3S	Metallo-beta-lactamase superfamily protein [*Paracidovorax citrulli*]
fig|80869.159.peg.1219	T3S	Trehalase [*Paracidovorax citrulli*]
fig|80869.159.peg.1239	T3S	hypothetical protein [*Paracidovorax citrulli*]
fig|80869.159.peg.1267	T3S	ABC transporter permease [*Paracidovorax citrulli*]
fig|80869.159.peg.128	T3S	2OG-Fe dioxygenase family protein [*Paracidovorax citrulli*]
fig|80869.159.peg.1318	T3S	hypothetical protein [*Paracidovorax citrulli*]
fig|80869.159.peg.1330	T3S	YitT family protein [*Paracidovorax citrulli*]
fig|80869.159.peg.1331	T3S	hypothetical protein [*Paracidovorax citrulli*]
fig|80869.159.peg.1346	T3S	3-methyl-2-oxobutanoate hydroxymethyltransferase [*Paracidovorax citrulli*]
fig|80869.159.peg.1367	T3S	signal recognition particle-docking protein FtsY [*Paracidovorax citrulli*]
fig|80869.159.peg.2465	T4S	major facilitator superfamily MFS_1 [*Paracidovorax citrulli* AAC00-1]
fig|80869.159.peg.3239	T4S	Ig domain protein, group 1 domain protein [*Paracidovorax citrulli* AAC00-1]
fig|80869.159.peg.4395	T4S	hypothetical protein [*Paracidovorax citrulli*]
fig|80869.159.peg.3397	T4S	acyl-CoA thioesterase [*Paracidovorax citrulli*]
fig|80869.159.peg.2789	T4S	STY0301 family protein [*Paracidovorax citrulli*]
fig|80869.159.peg.1982	T4S	ABC transporter ATP-binding protein [*Paracidovorax citrulli*]
fig|80869.159.peg.1360	T4S	ferritin-like domain-containing protein [*Paracidovorax citrulli*]
fig|80869.159.peg.2345	T4S	terminase small subunit [*Paracidovorax citrulli*]
fig|80869.159.peg.905	T4S	molybdenum cofactor biosynthesis protein MoaE [*Paracidovorax citrulli*]
fig|80869.159.peg.136	T4S	hypothetical protein [*Paracidovorax citrulli*]
fig|80869.159.peg.1727	T4S	isoaspartyl peptidase/L-asparaginase [*Paracidovorax citrulli*]
fig|80869.159.peg.3436	T4S	RNA-binding protein [*Paracidovorax citrulli*]
fig|80869.159.peg.39	T4S	hypothetical protein Aave_3072 [*Paracidovorax citrulli* AAC00-1]
fig|80869.159.peg.3117	T4S	DUF1795 domain-containing protein [*Paracidovorax citrulli*]
fig|80869.159.peg.3942	T4S	amino-acid N-acetyltransferase [*Paracidovorax citrulli*]
fig|80869.159.peg.4287	T4S	DUF924 family protein [*Paracidovorax citrulli*]
fig|80869.159.peg.1656	T4S	hypothetical protein [*Paracidovorax citrulli*]
fig|80869.159.peg.3952	T4S	50S ribosomal protein L11 [*Paracidovorax citrulli*]
fig|80869.159.peg.3912	T4S	hypothetical protein [*Paracidovorax citrulli*]
fig|80869.159.peg.2440	T4S	hypothetical protein C8E08_4485 [*Paracidovorax citrulli*]
fig|80869.159.peg.4200	T4S	SMC-Scp complex subunit ScpB [*Paracidovorax citrulli*]
fig|80869.159.peg.518	T4S	PP2C family serine/threonine-protein phosphatase [*Paracidovorax citrulli*]
fig|80869.159.peg.2194	T4S	hypothetical protein [*Paracidovorax citrulli*]
fig|80869.159.peg.3973	T4S	cryptochrome/photolyase family protein [*Paracidovorax citrulli*]
fig|80869.159.peg.982	T4S	phosphopyruvate hydratase [*Paracidovorax citrulli*]
fig|80869.159.peg.1412	T4S	glutathione-regulated potassium-efflux system protein KefC [*Paracidovorax citrulli*]
fig|80869.159.peg.461	T4S	hypothetical protein [*Paracidovorax citrulli*]
fig|80869.159.peg.240	T4S	hypothetical protein [*Paracidovorax citrulli*]
fig|80869.159.peg.2225	T4S	ATP-binding protein [*Paracidovorax citrulli*]
fig|80869.159.peg.3861	T4S	DUF3025 domain-containing protein [*Paracidovorax citrulli*]

Prot	Pred	Description
fig|80869.159.peg.519	T4S	serine/threonine-protein kinase [*Paracidovorax citrulli*]
fig|80869.159.peg.4378	T4S	hypothetical protein [*Paracidovorax citrulli*]
fig|80869.159.peg.4021	T4S	inositol monophosphatase family protein [*Paracidovorax citrulli*]
fig|80869.159.peg.1254	T4S	N-acetylmuramoyl-L-alanine amidase [*Paracidovorax citrulli*]
fig|80869.159.peg.2986	T4S	AraC family transcriptional regulator [*Paracidovorax citrulli*]
fig|80869.159.peg.298	T4S	hypothetical protein [*Paracidovorax citrulli*]
fig|80869.159.peg.3600	T4S	type VI secretion system tip protein VgrG [*Paracidovorax citrulli*]
fig|80869.159.peg.4482	T4S	type VI secretion system tip protein VgrG [*Paracidovorax citrulli*]
fig|80869.159.peg.1513	T4S	Fic family protein [*Paracidovorax citrulli*]
fig|80869.159.peg.4360	T4S	4-hydroxy-3-methylbut-2-enyl diphosphate reductase [*Paracidovorax citrulli*]
fig|80869.159.peg.4162	T4S	SsrA-binding protein SmpB [*Paracidovorax citrulli*]
fig|80869.159.peg.2738	T4S	F0F1 ATP synthase subunit gamma [*Paracidovorax citrulli*]
fig|80869.159.peg.1896	T4S	hypothetical protein [*Paracidovorax citrulli*]
fig|80869.159.peg.3102	T4S	transglycosylase SLT domain-containing protein [*Paracidovorax citrulli*]
fig|80869.159.peg.3007	T4S	type III secretion system outer membrane ring subunit SctC [*Paracidovorax citrulli*]
fig|80869.159.peg.2326	T4S	hypothetical protein [*Paracidovorax citrulli*]
fig|80869.159.peg.1778	T4S	XopE/AvrPphe family type III secretion system effector [*Paracidovorax citrulli*]
fig|80869.159.peg.3320	T4S	hypothetical protein [*Paracidovorax citrulli*]
fig|80869.159.peg.1475	T4S	5'-nucleotidase [*Paracidovorax citrulli*]
fig|80869.159.peg.2951	T4S	30S ribosomal protein S4 [*Paracidovorax citrulli*]
fig|80869.159.peg.3011	T4S	adenylate kinase [*Paracidovorax citrulli*]
fig|80869.159.peg.221	T4S	hypothetical protein [*Paracidovorax citrulli*]
fig|80869.159.peg.488	T4S	type IV pilin protein [*Paracidovorax citrulli*]
fig|80869.159.peg.1361	T4S	BON domain-containing protein [*Paracidovorax citrulli*]
fig|80869.159.peg.3928	T4S	MetQ/NlpA family ABC transporter substrate-binding protein [*Paracidovorax citrulli*]
fig|80869.159.peg.4243	T4S	Bug family tripartite tricarboxylate transporter substrate binding protein
fig|80869.159.peg.2231	T4S	tripartite tricarboxylate transporter substrate binding protein [*Paracidovorax citrulli*]
fig|80869.159.peg.3934	T4S	sulfate ABC transporter substrate-binding protein [*Paracidovorax citrulli*]
fig|80869.159.peg.3909	T4S	Ku protein [*Paracidovorax citrulli*]
fig|80869.159.peg.3788	T4S	hypothetical protein [*Paracidovorax citrulli*]
fig|80869.159.peg.4288	T4S	OmpA family protein [*Paracidovorax citrulli*]
fig|80869.159.peg.2308	T4S	endolysin [*Paracidovorax citrulli*]
fig|80869.159.peg.2555	T4S	HTH-type transcriptional regulator GltC [*Paracidovorax citrulli*]
fig|80869.159.peg.329	T4S	sigma-54 dependent transcriptional regulator [*Paracidovorax citrulli*]
fig|80869.159.peg.1476	T4S	EF-hand domain-containing protein [*Paracidovorax citrulli*]

*P_citrulli*_CCRMAc12

Prot	Pred	Description
fig|80869.148.peg.2339	T3S	major facilitator superfamily MFS_1 [*Paracidovorax citrulli* AAC00-1]
fig|80869.148.peg.3139	T3S	Ig domain protein, group 1 domain protein [*Paracidovorax citrulli* AAC00-1]
fig|80869.148.peg.4359	T3S	hypothetical protein [*Paracidovorax citrulli*]
fig|80869.148.peg.3381	T3S	acyl-CoA thioesterase [*Paracidovorax citrulli*]
fig|80869.148.peg.2809	T3S	STY0301 family protein [*Paracidovorax citrulli*]

Prot	Pred	Description
fig|80869.148.peg.1843	T3S	ABC transporter ATP-binding protein [*Paracidovorax citrulli*]
fig|80869.148.peg.1	T3S	peptidoglycan-binding domain-containing protein, partial [*Paracidovorax citrulli*]
fig|80869.148.peg.1001	T3S	hypothetical protein [*Paracidovorax citrulli*]
fig|80869.148.peg.1021	T3S	NADH-quinone oxidoreductase subunit NuoI [*Paracidovorax citrulli*]
fig|80869.148.peg.1029	T3S	ABC transporter transmembrane domain-containing protein [*Paracidovorax citrulli*]
fig|80869.148.peg.1047	T3S	helix-turn-helix domain-containing protein [*Paracidovorax citrulli*]
fig|80869.148.peg.1056	T3S	N-acetylmuramoyl-L-alanine amidase [*Paracidovorax citrulli*]
fig|80869.148.peg.1067	T3S	HDOD domain-containing protein [*Paracidovorax citrulli*]
fig|80869.148.peg.1071	T3S	hypothetical protein [*Paracidovorax citrulli*]
fig|80869.148.peg.1097	T3S	pyridoxal kinase PdxY [*Paracidovorax citrulli*]
fig|80869.148.peg.1103	T3S	DNA internalization-related competence protein ComEC/Rec2 [*Paracidovorax citrulli*]
fig|80869.148.peg.1111	T3S	VF530 family protein [*Paracidovorax citrulli*]
fig|80869.148.peg.113	T3S	ribonucleoside-diphosphate reductase subunit alpha [*Paracidovorax citrulli*]
fig|80869.148.peg.1143	T3S	CaiB/BaiF CoA-transferase family protein [*Paracidovorax citrulli*]
fig|80869.148.peg.1179	T3S	hypothetical protein [*Paracidovorax citrulli*]
fig|80869.148.peg.1191	T3S	YitT family protein [*Paracidovorax citrulli*]
fig|80869.148.peg.1192	T3S	outer membrane protein assembly factor BamE [*Paracidovorax citrulli*]
fig|80869.148.peg.1207	T3S	3-methyl-2-oxobutanoate hydroxymethyltransferase [*Paracidovorax citrulli*]
fig|80869.148.peg.1228	T3S	signal recognition particle-docking protein FtsY [*Paracidovorax citrulli*]
fig|80869.148.peg.1238	T3S	MATE family efflux transporter [*Paracidovorax citrulli*]
fig|80869.148.peg.125	T3S	3-deoxy-7-phosphoheptulonate synthase [*Paracidovorax citrulli*]
fig|80869.148.peg.1306	T3S	type III secretion system chaperone [*Paracidovorax citrulli*]
fig|80869.148.peg.1307	T3S	hypothetical protein [*Paracidovorax citrulli*]
fig|80869.148.peg.1335	T3S	LLM class flavin-dependent oxidoreductase [*Paracidovorax citrulli*]
fig|80869.148.peg.1349	T3S	hypothetical protein [*Paracidovorax citrulli*]
fig|80869.148.peg.1362	T3S	ABC transporter permease [*Paracidovorax citrulli*]
fig|80869.148.peg.1363	T3S	ABC transporter permease subunit [*Paracidovorax citrulli*]
fig|80869.148.peg.2339	T4S	major facilitator superfamily MFS_1 [*Paracidovorax citrulli* AAC00-1]
fig|80869.148.peg.3139	T4S	Ig domain protein, group 1 domain protein [*Paracidovorax citrulli* AAC00-1]
fig|80869.148.peg.4359	T4S	hypothetical protein [*Paracidovorax citrulli*]
fig|80869.148.peg.3381	T4S	acyl-CoA thioesterase [*Paracidovorax citrulli*]
fig|80869.148.peg.2809	T4S	STY0301 family protein [*Paracidovorax citrulli*]
fig|80869.148.peg.1843	T4S	ABC transporter ATP-binding protein [*Paracidovorax citrulli*]
fig|80869.148.peg.3884	T4S	Ku protein [*Paracidovorax citrulli*]
fig|80869.148.peg.1221	T4S	ferritin-like domain-containing protein [*Paracidovorax citrulli*]
fig|80869.148.peg.2061	T4S	terminase small subunit [*Paracidovorax citrulli*]
fig|80869.148.peg.995	T4S	molybdenum cofactor biosynthesis protein MoaE [*Paracidovorax citrulli*]
fig|80869.148.peg.443	T4S	hypothetical protein [*Paracidovorax citrulli*]
fig|80869.148.peg.1589	T4S	isoaspartyl peptidase/L-asparaginase [*Paracidovorax citrulli*]
fig|80869.148.peg.3420	T4S	RNA-binding protein [*Paracidovorax citrulli*]
fig|80869.148.peg.3981	T4S	hypothetical protein Aave_3072 [*Paracidovorax citrulli* AAC00-1]
fig|80869.148.peg.3017	T4S	DUF1795 domain-containing protein [*Paracidovorax citrulli*]
fig|80869.148.peg.3916	T4S	amino-acid N-acetyltransferase [*Paracidovorax citrulli*]
fig|80869.148.peg.4252	T4S	DUF924 family protein [*Paracidovorax citrulli*]

Prot	Pred	Description
fig|80869.148.peg.1517	T4S	hypothetical protein [*Paracidovorax citrulli*]
fig|80869.148.peg.3926	T4S	50S ribosomal protein L11 [*Paracidovorax citrulli*]
fig|80869.148.peg.3887	T4S	hypothetical protein [*Paracidovorax citrulli*]
fig|80869.148.peg.2314	T4S	hypothetical protein C8E08_4485 [*Paracidovorax citrulli*]
fig|80869.148.peg.4165	T4S	SMC-Scp complex subunit ScpB [*Paracidovorax citrulli*]
fig|80869.148.peg.822	T4S	PP2C family serine/threonine-protein phosphatase [*Paracidovorax citrulli*]
fig|80869.148.peg.2218	T4S	hypothetical protein [*Paracidovorax citrulli*]
fig|80869.148.peg.3947	T4S	cryptochrome/photolyase family protein [*Paracidovorax citrulli*]
fig|80869.148.peg.1073	T4S	phosphopyruvate hydratase [*Paracidovorax citrulli*]
fig|80869.148.peg.1273	T4S	glutathione-regulated potassium-efflux system protein KefC [*Paracidovorax citrulli*]
fig|80869.148.peg.766	T4S	hypothetical protein [*Paracidovorax citrulli*]
fig|80869.148.peg.546	T4S	hypothetical protein [*Paracidovorax citrulli*]
fig|80869.148.peg.1941	T4S	ATP-binding protein [*Paracidovorax citrulli*]
fig|80869.148.peg.3835	T4S	DUF3025 domain-containing protein [*Paracidovorax citrulli*]
fig|80869.148.peg.823	T4S	serine/threonine-protein kinase [*Paracidovorax citrulli*]
fig|80869.148.peg.4343	T4S	hypothetical protein [*Paracidovorax citrulli*]
fig|80869.148.peg.4044	T4S	inositol monophosphatase family protein [*Paracidovorax citrulli*]
fig|80869.148.peg.2499	T4S	N-acetylmuramoyl-L-alanine amidase [*Paracidovorax citrulli*]
fig|80869.148.peg.334	T4S	AraC family transcriptional regulator [*Paracidovorax citrulli*]
fig|80869.148.peg.604	T4S	hypothetical protein [*Paracidovorax citrulli*]
fig|80869.148.peg.1375	T4S	Fic family protein [*Paracidovorax citrulli*]
fig|80869.148.peg.4325	T4S	4-hydroxy-3-methylbut-2-enyl diphosphate reductase [*Paracidovorax citrulli*]
fig|80869.148.peg.4127	T4S	SsrA-binding protein SmpB [*Paracidovorax citrulli*]
fig|80869.148.peg.2758	T4S	F0F1 ATP synthase subunit gamma [*Paracidovorax citrulli*]
fig|80869.148.peg.1757	T4S	hypothetical protein [*Paracidovorax citrulli*]
fig|80869.148.peg.3002	T4S	transglycosylase SLT domain-containing protein [*Paracidovorax citrulli*]
fig|80869.148.peg.354	T4S	type III secretion system outer membrane ring subunit SctC [*Paracidovorax citrulli*]
fig|80869.148.peg.2042	T4S	hypothetical protein [*Paracidovorax citrulli*]
fig|80869.148.peg.1640	T4S	XopE/AvrPphe family type III secretion system effector [*Paracidovorax citrulli*]
fig|80869.148.peg.3305	T4S	hypothetical protein [*Paracidovorax citrulli*]
fig|80869.148.peg.1337	T4S	5'-nucleotidase [*Paracidovorax citrulli*]
fig|80869.148.peg.299	T4S	30S ribosomal protein S4 [*Paracidovorax citrulli*]
fig|80869.148.peg.2911	T4S	adenylate kinase [*Paracidovorax citrulli*]
fig|80869.148.peg.527	T4S	hypothetical protein [*Paracidovorax citrulli*]
fig|80869.148.peg.792	T4S	type IV pilin protein [*Paracidovorax citrulli*]
fig|80869.148.peg.1222	T4S	BON domain-containing protein [*Paracidovorax citrulli*]
fig|80869.148.peg.3902	T4S	MetQ/NlpA family ABC transporter substrate-binding protein [*Paracidovorax citrulli*]
fig|80869.148.peg.4208	T4S	Bug family tripartite tricarboxylate transporter substrate binding protein [*Paracidovorax citrulli*]
fig|80869.148.peg.1947	T4S	tripartite tricarboxylate transporter substrate binding protein [*Paracidovorax citrulli*]
fig|80869.148.peg.3908	T4S	sulfate ABC transporter substrate-binding protein [*Paracidovorax citrulli*]
fig|80869.148.peg.3709	T4S	hypothetical protein [*Paracidovorax citrulli*]
fig|80869.148.peg.4253	T4S	OmpA family protein [*Paracidovorax citrulli*]
fig|80869.148.peg.2024	T4S	endolysin [*Paracidovorax citrulli*]
fig|80869.148.peg.2576	T4S	HTH-type transcriptional regulator GltC [*Paracidovorax citrulli*]

Prot	Pred	Description
fig|80869.148.peg.635	T4S	sigma-54 dependent transcriptional regulator [*Paracidovorax citrulli*]
fig|80869.148.peg.1338	T4S	EF-hand domain-containing protein [*Paracidovorax citrulli*]

*P_citrulli*_CCRMAcMP2

Prot	Pred	Description
fig|80869.161.peg.1474	T3S	major facilitator superfamily MFS_1 [*Paracidovorax citrulli* AAC00-1]
fig|80869.161.peg.3385	T3S	Ig domain protein, group 1 domain protein [*Paracidovorax citrulli* AAC00-1]
fig|80869.161.peg.4419	T3S	hypothetical protein [*Paracidovorax citrulli*]
fig|80869.161.peg.3547	T3S	acyl-CoA thioesterase [*Paracidovorax citrulli*]
fig|80869.161.peg.1612	T3S	STY0301 family protein [*Paracidovorax citrulli*]
fig|80869.161.peg.2004	T3S	ABC transporter ATP-binding protein [*Paracidovorax citrulli*]
fig|80869.161.peg.1	T3S	DUF72 domain-containing protein [*Paracidovorax citrulli*]
fig|80869.161.peg.1040	T3S	rhodanese-like domain-containing protein [*Paracidovorax citrulli*]
fig|80869.161.peg.1057	T3S	GNAT family N-acetyltransferase [*Paracidovorax citrulli*]
fig|80869.161.peg.1070	T3S	ABC transporter permease [*Paracidovorax citrulli*]
fig|80869.161.peg.1074	T3S	hypothetical protein [*Paracidovorax citrulli*]
fig|80869.161.peg.1075	T3S	Metallo-beta-lactamase superfamily protein [*Paracidovorax citrulli*]
fig|80869.161.peg.1087	T3S	Trehalase [*Paracidovorax citrulli*]
fig|80869.161.peg.1107	T3S	hypothetical protein [*Paracidovorax citrulli*]
fig|80869.161.peg.1135	T3S	ABC transporter permease [*Paracidovorax citrulli*]
fig|80869.161.peg.1190	T3S	hypothetical protein [*Paracidovorax citrulli*]
fig|80869.161.peg.1202	T3S	YitT family protein [*Paracidovorax citrulli*]
fig|80869.161.peg.1203	T3S	hypothetical protein [*Paracidovorax citrulli*]
fig|80869.161.peg.1218	T3S	3-methyl-2-oxobutanoate hydroxymethyltransferase [*Paracidovorax citrulli*]
fig|80869.161.peg.1240	T3S	signal recognition particle-docking protein FtsY [*Paracidovorax citrulli*]
fig|80869.161.peg.1250	T3S	MATE family efflux transporter [*Paracidovorax citrulli*]
fig|80869.161.peg.1317	T3S	type III secretion system chaperone [*Paracidovorax citrulli*]
fig|80869.161.peg.1318	T3S	hypothetical protein [*Paracidovorax citrulli*]
fig|80869.161.peg.1330	T3S	hypothetical protein [*Paracidovorax citrulli*]
fig|80869.161.peg.1344	T3S	LLM class flavin-dependent oxidoreductase [*Paracidovorax citrulli*]
fig|80869.161.peg.1357	T3S	hypothetical protein [*Paracidovorax citrulli*]
fig|80869.161.peg.1370	T3S	ABC transporter permease [*Paracidovorax citrulli*]
fig|80869.161.peg.1371	T3S	ABC transporter permease subunit [*Paracidovorax citrulli*]
fig|80869.161.peg.1379	T3S	NAD-dependent succinate-semialdehyde dehydrogenase [*Paracidovorax citrulli*]
fig|80869.161.peg.1394	T3S	GntR family transcriptional regulator [*Paracidovorax citrulli*]
fig|80869.161.peg.1409	T3S	cytochrome o ubiquinol oxidase subunit IV [*Paracidovorax citrulli*]
fig|80869.161.peg.1443	T3S	multiple monosaccharide ABC transporter permease [*Paracidovorax citrulli*]
fig|80869.161.peg.1447	T3S	aldehyde dehydrogenase family protein [*Paracidovorax citrulli*]
fig|80869.161.peg.1448	T3S	sugar lactone lactonase YvrE [*Paracidovorax citrulli*]
fig|80869.161.peg.1474	T4S	major facilitator superfamily MFS_1 [*Paracidovorax citrulli* AAC00-1]
fig|80869.161.peg.3385	T4S	Ig domain protein, group 1 domain protein [*Paracidovorax citrulli* AAC00-1]
fig|80869.161.peg.4419	T4S	hypothetical protein [*Paracidovorax citrulli*]
fig|80869.161.peg.3547	T4S	acyl-CoA thioesterase [*Paracidovorax citrulli*]
fig|80869.161.peg.1612	T4S	STY0301 family protein [*Paracidovorax citrulli*]

Prot	Pred	Description
fig|80869.161.peg.2004	T4S	ABC transporter ATP-binding protein [*Paracidovorax citrulli*]
fig|80869.161.peg.1719	T4S	Ku protein [*Paracidovorax citrulli*]
fig|80869.161.peg.1722	T4S	hypothetical protein [*Paracidovorax citrulli*]
fig|80869.161.peg.1233	T4S	ferritin-like domain-containing protein [*Paracidovorax citrulli*]
fig|80869.161.peg.147	T4S	terminase small subunit [*Paracidovorax citrulli*]
fig|80869.161.peg.774	T4S	molybdenum cofactor biosynthesis protein MoaE [*Paracidovorax citrulli*]
fig|80869.161.peg.506	T4S	hypothetical protein [*Paracidovorax citrulli*]
fig|80869.161.peg.195	T4S	isoaspartyl peptidase/L-asparaginase [*Paracidovorax citrulli*]
fig|80869.161.peg.3586	T4S	RNA-binding protein [*Paracidovorax citrulli*]
fig|80869.161.peg.410	T4S	hypothetical protein Aave_3072 [*Paracidovorax citrulli* AAC00-1]
fig|80869.161.peg.3165	T4S	DUF1795 domain-containing protein [*Paracidovorax citrulli*]
fig|80869.161.peg.1752	T4S	amino-acid N-acetyltransferase [*Paracidovorax citrulli*]
fig|80869.161.peg.4313	T4S	DUF924 family protein [*Paracidovorax citrulli*]
fig|80869.161.peg.3113	T4S	hypothetical protein [*Paracidovorax citrulli*]
fig|80869.161.peg.4035	T4S	50S ribosomal protein L11 [*Paracidovorax citrulli*]
fig|80869.161.peg.1449	T4S	hypothetical protein C8E08_4485 [*Paracidovorax citrulli*]
fig|80869.161.peg.2405	T4S	LysR family transcriptional regulator [Paracidovorax]
fig|80869.161.peg.4227	T4S	SMC-Scp complex subunit ScpB [*Paracidovorax citrulli*]
fig|80869.161.peg.2982	T4S	PP2C family serine/threonine-protein phosphatase [*Paracidovorax citrulli*]
fig|80869.161.peg.2213	T4S	hypothetical protein [*Paracidovorax citrulli*]
fig|80869.161.peg.4056	T4S	cryptochrome/photolyase family protein [*Paracidovorax citrulli*]
fig|80869.161.peg.852	T4S	phosphopyruvate hydratase [*Paracidovorax citrulli*]
fig|80869.161.peg.1285	T4S	glutathione-regulated potassium-efflux system protein KefC [*Paracidovorax citrulli*]
fig|80869.161.peg.1899	T4S	hypothetical protein [*Paracidovorax citrulli*]
fig|80869.161.peg.610	T4S	hypothetical protein [*Paracidovorax citrulli*]
fig|80869.161.peg.25	T4S	ATP-binding protein [*Paracidovorax citrulli*]
fig|80869.161.peg.3989	T4S	DUF3025 domain-containing protein [*Paracidovorax citrulli*]
fig|80869.161.peg.2983	T4S	serine/threonine-protein kinase [*Paracidovorax citrulli*]
fig|80869.161.peg.4404	T4S	hypothetical protein [*Paracidovorax citrulli*]
fig|80869.161.peg.4103	T4S	inositol monophosphatase family protein [*Paracidovorax citrulli*]
fig|80869.161.peg.1122	T4S	N-acetylmuramoyl-L-alanine amidase [*Paracidovorax citrulli*]
fig|80869.161.peg.2721	T4S	AraC family transcriptional regulator [*Paracidovorax citrulli*]
fig|80869.161.peg.668	T4S	hypothetical protein [*Paracidovorax citrulli*]
fig|80869.161.peg.2860	T4S	Fic family protein [*Paracidovorax citrulli*]
fig|80869.161.peg.4386	T4S	4-hydroxy-3-methylbut-2-enyl diphosphate reductase [*Paracidovorax citrulli*]
fig|80869.161.peg.4187	T4S	SsrA-binding protein SmpB [*Paracidovorax citrulli*]
fig|80869.161.peg.2586	T4S	F0F1 ATP synthase subunit gamma [*Paracidovorax citrulli*]
fig|80869.161.peg.363	T4S	hypothetical protein [*Paracidovorax citrulli*]
fig|80869.161.peg.3352	T4S	transglycosylase SLT domain-containing protein [*Paracidovorax citrulli*]
fig|80869.161.peg.2741	T4S	type III secretion system outer membrane ring subunit SctC [*Paracidovorax citrulli*]
fig|80869.161.peg.128	T4S	hypothetical protein [*Paracidovorax citrulli*]
fig|80869.161.peg.246	T4S	XopE/AvrPphe family type III secretion system effector [*Paracidovorax citrulli*]
fig|80869.161.peg.1346	T4S	5'-nucleotidase [*Paracidovorax citrulli*]
fig|80869.161.peg.1565	T4S	hypothetical protein [*Paracidovorax citrulli*]
fig|80869.161.peg.2686	T4S	30S ribosomal protein S4 [*Paracidovorax citrulli*]

Prot	Pred	Description
fig|80869.161.peg.3259	T4S	adenylate kinase [*Paracidovorax citrulli*]
fig|80869.161.peg.591	T4S	hypothetical protein [*Paracidovorax citrulli*]
fig|80869.161.peg.3468	T4S	hypothetical protein [*Paracidovorax citrulli*]
fig|80869.161.peg.1925	T4S	type IV pilin protein [*Paracidovorax citrulli*]
fig|80869.161.peg.1234	T4S	BON domain-containing protein [*Paracidovorax citrulli*]
fig|80869.161.peg.1738	T4S	MetQ/NlpA family ABC transporter substrate-binding protein [*Paracidovorax citrulli*]
fig|80869.161.peg.4269	T4S	Bug family tripartite tricarboxylate transporter substrate binding protein [*Paracidovorax citrulli*]
fig|80869.161.peg.31	T4S	tripartite tricarboxylate transporter substrate binding protein [*Paracidovorax citrulli*]
fig|80869.161.peg.1744	T4S	sulfate ABC transporter substrate-binding protein [*Paracidovorax citrulli*]
fig|80869.161.peg.3859	T4S	hypothetical protein [*Paracidovorax citrulli*]
fig|80869.161.peg.4314	T4S	OmpA family protein [*Paracidovorax citrulli*]
fig|80869.161.peg.110	T4S	endolysin [*Paracidovorax citrulli*]
fig|80869.161.peg.1765	T4S	sigma-54 dependent transcriptional regulator [*Paracidovorax citrulli*]
fig|80869.161.peg.1347	T4S	EF-hand domain-containing protein [*Paracidovorax citrulli*]

*P_citrulli_*CCRMAcR2

Prot	Pred	Description
fig|80869.162.peg.2061	T3S	major facilitator superfamily MFS_1 [*Paracidovorax citrulli* AAC00-1]
fig|80869.162.peg.3252	T3S	Ig domain protein, group 1 domain protein [*Paracidovorax citrulli* AAC00-1]
fig|80869.162.peg.4363	T3S	hypothetical protein [*Paracidovorax citrulli*]
fig|80869.162.peg.3412	T3S	acyl-CoA thioesterase [*Paracidovorax citrulli*]
fig|80869.162.peg.860	T3S	STY0301 family protein [*Paracidovorax citrulli*]
fig|80869.162.peg.1249	T3S	ABC transporter ATP-binding protein [*Paracidovorax citrulli*]
fig|80869.162.peg.1	T3S	DUF72 domain-containing protein [*Paracidovorax citrulli*]
fig|80869.162.peg.1053	T3S	hypothetical protein [*Paracidovorax citrulli*]
fig|80869.162.peg.1057	T3S	DUF1653 domain-containing protein [*Paracidovorax citrulli*]
fig|80869.162.peg.1072	T3S	isoleucine--tRNA ligase [*Paracidovorax citrulli*]
fig|80869.162.peg.1117	T3S	adenylate/guanylate cyclase domain-containing protein [*Paracidovorax citrulli*]
fig|80869.162.peg.1156	T3S	urease accessory UreF family protein [*Paracidovorax citrulli*]
fig|80869.162.peg.1157	T3S	urease accessory protein UreG [*Paracidovorax citrulli*]
fig|80869.162.peg.1178	T3S	XopAP family type III secretion system effector [*Paracidovorax citrulli*]
fig|80869.162.peg.1216	T3S	cellulase family glycosylhydrolase [*Paracidovorax citrulli*]
fig|80869.162.peg.1228	T3S	YdiU family protein [*Paracidovorax citrulli*]
fig|80869.162.peg.1306	T3S	BON domain-containing protein [*Paracidovorax citrulli*]
fig|80869.162.peg.1307	T3S	SulP family inorganic anion transporter [*Paracidovorax citrulli*]
fig|80869.162.peg.1315	T3S	FAD-dependent oxidoreductase [*Paracidovorax citrulli*]
fig|80869.162.peg.1317	T3S	tRNA (guanosine(46)-N7)-methyltransferase TrmB [*Paracidovorax citrulli*]
fig|80869.162.peg.1392	T3S	ABC transporter permease [*Paracidovorax citrulli*]
fig|80869.162.peg.1462	T3S	hypothetical protein [*Paracidovorax citrulli*]
fig|80869.162.peg.1464	T3S	trifunctional transcriptional regulator/proline dehydrogenase/L-glutamate gamma-semialdehyde dehydrogenase [*Paracidovorax citrulli*]
fig|80869.162.peg.1490	T3S	hypothetical protein [*Paracidovorax citrulli*]
fig|80869.162.peg.150	T3S	hypothetical protein [*Paracidovorax citrulli*]
fig|80869.162.peg.1505	T3S	CysB family HTH-type transcriptional regulator [Comamonadaceae]
fig|80869.162.peg.1519	T3S	2OG-Fe dioxygenase family protein [*Paracidovorax citrulli*]

Prot	Pred	Description
fig|80869.162.peg.1544	T3S	TetR/AcrR family transcriptional regulator [*Paracidovorax citrulli*]
fig|80869.162.peg.2061	T4S	major facilitator superfamily MFS_1 [*Paracidovorax citrulli* AAC00-1]
fig|80869.162.peg.3252	T4S	Ig domain protein, group 1 domain protein [*Paracidovorax citrulli* AAC00-1]
fig|80869.162.peg.4363	T4S	hypothetical protein [*Paracidovorax citrulli*]
fig|80869.162.peg.3412	T4S	acyl-CoA thioesterase [*Paracidovorax citrulli*]
fig|80869.162.peg.860	T4S	STY0301 family protein [*Paracidovorax citrulli*]
fig|80869.162.peg.1249	T4S	ABC transporter ATP-binding protein [*Paracidovorax citrulli*]
fig|80869.162.peg.967	T4S	Ku protein [*Paracidovorax citrulli*]
fig|80869.162.peg.970	T4S	hypothetical protein [*Paracidovorax citrulli*]
fig|80869.162.peg.701	T4S	ferritin-like domain-containing protein [*Paracidovorax citrulli*]
fig|80869.162.peg.147	T4S	terminase small subunit [*Paracidovorax citrulli*]
fig|80869.162.peg.474	T4S	molybdenum cofactor biosynthesis protein MoaE [*Paracidovorax citrulli*]
fig|80869.162.peg.1511	T4S	hypothetical protein [*Paracidovorax citrulli*]
fig|80869.162.peg.195	T4S	isoaspartyl peptidase/L-asparaginase [*Paracidovorax citrulli*]
fig|80869.162.peg.3451	T4S	RNA-binding protein [*Paracidovorax citrulli*]
fig|80869.162.peg.1610	T4S	hypothetical protein Aave_3072 [*Paracidovorax citrulli* AAC00-1]
fig|80869.162.peg.3032	T4S	DUF1795 domain-containing protein [*Paracidovorax citrulli*]
fig|80869.162.peg.246	T4S	XopE/AvrPphe family type III secretion system effector [*Paracidovorax citrulli*]
fig|80869.162.peg.1000	T4S	amino-acid N-acetyltransferase [*Paracidovorax citrulli*]
fig|80869.162.peg.3505	T4S	DUF924 family protein [*Paracidovorax citrulli*]
fig|80869.162.peg.2980	T4S	hypothetical protein [*Paracidovorax citrulli*]
fig|80869.162.peg.4065	T4S	50S ribosomal protein L11 [*Paracidovorax citrulli*]
fig|80869.162.peg.2036	T4S	hypothetical protein C8E08_4485 [*Paracidovorax citrulli*]
fig|80869.162.peg.2274	T4S	LysR family transcriptional regulator [*Paracidovorax citrulli*]
fig|80869.162.peg.4212	T4S	SMC-Scp complex subunit ScpB [*Paracidovorax citrulli*]
fig|80869.162.peg.2850	T4S	PP2C family serine/threonine-protein phosphatase [*Paracidovorax citrulli*]
fig|80869.162.peg.1785	T4S	hypothetical protein [*Paracidovorax citrulli*]
fig|80869.162.peg.4086	T4S	cryptochrome/photolyase family protein [*Paracidovorax citrulli*]
fig|80869.162.peg.552	T4S	phosphopyruvate hydratase [*Paracidovorax citrulli*]
fig|80869.162.peg.753	T4S	glutathione-regulated potassium-efflux system protein KefC [*Paracidovorax citrulli*]
fig|80869.162.peg.1146	T4S	hypothetical protein [*Paracidovorax citrulli*]
fig|80869.162.peg.1402	T4S	hypothetical protein [*Paracidovorax citrulli*]
fig|80869.162.peg.25	T4S	ATP-binding protein [*Paracidovorax citrulli*]
fig|80869.162.peg.4021	T4S	DUF3025 domain-containing protein [*Paracidovorax citrulli*]
fig|80869.162.peg.2851	T4S	serine/threonine-protein kinase [*Paracidovorax citrulli*]
fig|80869.162.peg.4348	T4S	hypothetical protein [*Paracidovorax citrulli*]
fig|80869.162.peg.4133	T4S	inositol monophosphatase family protein [*Paracidovorax citrulli*]
fig|80869.162.peg.1921	T4S	N-acetylmuramoyl-L-alanine amidase [*Paracidovorax citrulli*]
fig|80869.162.peg.2590	T4S	AraC family transcriptional regulator [*Paracidovorax citrulli*]
fig|80869.162.peg.1460	T4S	hypothetical protein [*Paracidovorax citrulli*]
fig|80869.162.peg.2728	T4S	Fic family protein [*Paracidovorax citrulli*]
fig|80869.162.peg.4330	T4S	4-hydroxy-3-methylbut-2-enyl diphosphate reductase [*Paracidovorax citrulli*]
fig|80869.162.peg.4172	T4S	SsrA-binding protein SmpB [*Paracidovorax citrulli*]
fig|80869.162.peg.2454	T4S	F0F1 ATP synthase subunit gamma [*Paracidovorax citrulli*]
fig|80869.162.peg.364	T4S	hypothetical protein [*Paracidovorax citrulli*]
fig|80869.162.peg.3219	T4S	transglycosylase SLT domain-containing protein [*Paracidovorax citrulli*]

Prot	Pred	Description
fig|80869.162.peg.2610	T4S	type III secretion system outer membrane ring subunit SctC [*Paracidovorax citrulli*]
fig|80869.162.peg.129	T4S	hypothetical protein [*Paracidovorax citrulli*]
fig|80869.162.peg.815	T4S	5'-nucleotidase [*Paracidovorax citrulli*]
fig|80869.162.peg.2555	T4S	30S ribosomal protein S4 [*Paracidovorax citrulli*]
fig|80869.162.peg.3126	T4S	adenylate kinase [*Paracidovorax citrulli*]
fig|80869.162.peg.1383	T4S	hypothetical protein [*Paracidovorax citrulli*]
fig|80869.162.peg.3333	T4S	hypothetical protein [*Paracidovorax citrulli*]
fig|80869.162.peg.1172	T4S	type IV pilin protein [*Paracidovorax citrulli*]
fig|80869.162.peg.702	T4S	BON domain-containing protein [*Paracidovorax citrulli*]
fig|80869.162.peg.986	T4S	MetQ/NlpA family ABC transporter substrate-binding protein [*Paracidovorax citrulli*]
fig|80869.162.peg.4253	T4S	Bug family tripartite tricarboxylate transporter substrate binding protein [*Paracidovorax citrulli*]
fig|80869.162.peg.31	T4S	tripartite tricarboxylate transporter substrate binding protein [*Paracidovorax citrulli*]
fig|80869.162.peg.992	T4S	sulfate ABC transporter substrate-binding protein [*Paracidovorax citrulli*]
fig|80869.162.peg.3891	T4S	hypothetical protein [*Paracidovorax citrulli*]
fig|80869.162.peg.3506	T4S	OmpA family protein [*Paracidovorax citrulli*]
fig|80869.162.peg.111	T4S	endolysin [*Paracidovorax citrulli*]
fig|80869.162.peg.1014	T4S	sigma-54 dependent transcriptional regulator [*Paracidovorax citrulli*]
fig|80869.162.peg.816	T4S	EF-hand domain-containing protein [*Paracidovorax citrulli*]

*P_citrulli*_IBSBF1214

Prot	Pred	Description
fig|80869.163.peg.2544	T3S	major facilitator superfamily MFS_1 [*Paracidovorax citrulli* AAC00-1]
fig|80869.163.peg.3198	T3S	Ig domain protein, group 1 domain protein [*Paracidovorax citrulli* AAC00-1]
fig|80869.163.peg.4360	T3S	hypothetical protein [*Paracidovorax citrulli*]
fig|80869.163.peg.3449	T3S	acyl-CoA thioesterase [*Paracidovorax citrulli*]
fig|80869.163.peg.1811	T3S	STY0301 family protein [*Paracidovorax citrulli*]
fig|80869.163.peg.2213	T3S	ABC transporter ATP-binding protein [*Paracidovorax citrulli*]
fig|80869.163.peg.1	T3S	DUF72 domain-containing protein [*Paracidovorax citrulli*]
fig|80869.163.peg.1027	T3S	ribonucleoside-diphosphate reductase subunit alpha [*Paracidovorax citrulli*]
fig|80869.163.peg.1040	T3S	3-deoxy-7-phosphoheptulonate synthase [*Paracidovorax citrulli*]
fig|80869.163.peg.1097	T3S	chromate efflux transporter [*Paracidovorax citrulli*]
fig|80869.163.peg.1104	T3S	M48 family metallopeptidase [*Paracidovorax citrulli*]
fig|80869.163.peg.1120	T3S	DNA-3-methyladenine glycosylase I [*Paracidovorax citrulli*]
fig|80869.163.peg.1121	T3S	putative zinc protease protein [*Paracidovorax citrulli* AAC00-1]
fig|80869.163.peg.1153	T3S	DUF2169 domain-containing protein [*Paracidovorax citrulli*]
fig|80869.163.peg.1162	T3S	alpha/beta hydrolase [*Paracidovorax citrulli*]
fig|80869.163.peg.1191	T3S	septal ring lytic transglycosylase RlpA family protein [*Paracidovorax citrulli*]
fig|80869.163.peg.1204	T3S	hypothetical protein [*Paracidovorax citrulli*]
fig|80869.163.peg.1211	T3S	TRAP transporter small permease [Paracidovorax]
fig|80869.163.peg.1263	T3S	rhodanese-like domain-containing protein [*Paracidovorax citrulli*]
fig|80869.163.peg.1280	T3S	GNAT family N-acetyltransferase [*Paracidovorax citrulli*]
fig|80869.163.peg.1293	T3S	ABC transporter permease [*Paracidovorax citrulli*]
fig|80869.163.peg.1298	T3S	Metallo-beta-lactamase superfamily protein [*Paracidovorax citrulli*]

Prot	Pred	Description
fig|80869.163.peg.1310	T3S	Trehalase [*Paracidovorax citrulli*]
fig|80869.163.peg.1330	T3S	hypothetical protein [*Paracidovorax citrulli*]
fig|80869.163.peg.1358	T3S	ABC transporter permease [*Paracidovorax citrulli*]
fig|80869.163.peg.1413	T3S	hypothetical protein [*Paracidovorax citrulli*]
fig|80869.163.peg.1425	T3S	YitT family protein [*Paracidovorax citrulli*]
fig|80869.163.peg.1426	T3S	hypothetical protein [*Paracidovorax citrulli*]
fig|80869.163.peg.1441	T3S	3-methyl-2-oxobutanoate hydroxymethyltransferase [*Paracidovorax citrulli*]
fig|80869.163.peg.1463	T3S	signal recognition particle-docking protein FtsY [*Paracidovorax citrulli*]
fig|80869.163.peg.1473	T3S	MATE family efflux transporter [*Paracidovorax citrulli*]
fig|80869.163.peg.152	T3S	hypothetical protein [*Paracidovorax citrulli*]
fig|80869.163.peg.1541	T3S	type III secretion system chaperone [*Paracidovorax citrulli*]
fig|80869.163.peg.1542	T3S	hypothetical protein [*Paracidovorax citrulli*]
fig|80869.163.peg.2544	T4S	major facilitator superfamily MFS_1 [*Paracidovorax citrulli* AAC00-1]
fig|80869.163.peg.3198	T4S	Ig domain protein, group 1 domain protein [*Paracidovorax citrulli* AAC00-1]
fig|80869.163.peg.4360	T4S	hypothetical protein [*Paracidovorax citrulli*]
fig|80869.163.peg.3449	T4S	acyl-CoA thioesterase [*Paracidovorax citrulli*]
fig|80869.163.peg.1811	T4S	STY0301 family protein [*Paracidovorax citrulli*]
fig|80869.163.peg.2213	T4S	ABC transporter ATP-binding protein [*Paracidovorax citrulli*]
fig|80869.163.peg.1917	T4S	Ku protein [*Paracidovorax citrulli*]
fig|80869.163.peg.1456	T4S	ferritin-like domain-containing protein [*Paracidovorax citrulli*]
fig|80869.163.peg.149	T4S	terminase small subunit [*Paracidovorax citrulli*]
fig|80869.163.peg.2116	T4S	molybdenum cofactor biosynthesis protein MoaE [*Paracidovorax citrulli*]
fig|80869.163.peg.461	T4S	hypothetical protein [*Paracidovorax citrulli*]
fig|80869.163.peg.197	T4S	isoaspartyl peptidase/L-asparaginase [*Paracidovorax citrulli*]
fig|80869.163.peg.4403	T4S	NADAR family protein [Paracandidimonas lactea]
fig|80869.163.peg.3488	T4S	RNA-binding protein [*Paracidovorax citrulli*]
fig|80869.163.peg.3986	T4S	hypothetical protein Aave_3072 [*Paracidovorax citrulli* AAC00-1]
fig|80869.163.peg.3076	T4S	DUF1795 domain-containing protein [*Paracidovorax citrulli*]
fig|80869.163.peg.1950	T4S	amino-acid N-acetyltransferase [*Paracidovorax citrulli*]
fig|80869.163.peg.3542	T4S	DUF924 family protein [*Paracidovorax citrulli*]
fig|80869.163.peg.4500	T4S	50S ribosomal protein L11 [*Paracidovorax citrulli*]
fig|80869.163.peg.1920	T4S	hypothetical protein [*Paracidovorax citrulli*]
fig|80869.163.peg.2519	T4S	hypothetical protein C8E08_4485 [*Paracidovorax citrulli*]
fig|80869.163.peg.4132	T4S	SMC-Scp complex subunit ScpB [*Paracidovorax citrulli*]
fig|80869.163.peg.843	T4S	PP2C family serine/threonine-protein phosphatase [*Paracidovorax citrulli*]
fig|80869.163.peg.2423	T4S	hypothetical protein [*Paracidovorax citrulli*]
fig|80869.163.peg.4205	T4S	cryptochrome/photolyase family protein [*Paracidovorax citrulli*]
fig|80869.163.peg.2038	T4S	phosphopyruvate hydratase [*Paracidovorax citrulli*]
fig|80869.163.peg.1508	T4S	glutathione-regulated potassium-efflux system protein KefC [*Paracidovorax citrulli*]
fig|80869.163.peg.787	T4S	hypothetical protein [*Paracidovorax citrulli*]
fig|80869.163.peg.565	T4S	hypothetical protein [*Paracidovorax citrulli*]
fig|80869.163.peg.25	T4S	ATP-binding protein [*Paracidovorax citrulli*]
fig|80869.163.peg.3932	T4S	DUF3025 domain-containing protein [*Paracidovorax citrulli*]
fig|80869.163.peg.844	T4S	serine/threonine-protein kinase [*Paracidovorax citrulli*]
fig|80869.163.peg.4344	T4S	hypothetical protein [*Paracidovorax citrulli*]

Prot	Pred	Description
fig|80869.163.peg.4048	T4S	inositol monophosphatase family protein [*Paracidovorax citrulli*]
fig|80869.163.peg.1345	T4S	N-acetylmuramoyl-L-alanine amidase [*Paracidovorax citrulli*]
fig|80869.163.peg.2952	T4S	AraC family transcriptional regulator [*Paracidovorax citrulli*]
fig|80869.163.peg.624	T4S	hypothetical protein [*Paracidovorax citrulli*]
fig|80869.163.peg.1610	T4S	Fic family protein [*Paracidovorax citrulli*]
fig|80869.163.peg.4326	T4S	4-hydroxy-3-methylbut-2-enyl diphosphate reductase [*Paracidovorax citrulli*]
fig|80869.163.peg.4245	T4S	SsrA-binding protein SmpB [*Paracidovorax citrulli*]
fig|80869.163.peg.2816	T4S	F0F1 ATP synthase subunit gamma [*Paracidovorax citrulli*]
fig|80869.163.peg.367	T4S	hypothetical protein [*Paracidovorax citrulli*]
fig|80869.163.peg.3067	T4S	transglycosylase SLT domain-containing protein [*Paracidovorax citrulli*]
fig|80869.163.peg.2972	T4S	type III secretion system outer membrane ring subunit SctC [*Paracidovorax citrulli*]
fig|80869.163.peg.130	T4S	hypothetical protein [*Paracidovorax citrulli*]
fig|80869.163.peg.247	T4S	XopE/AvrPphe family type III secretion system effector [*Paracidovorax citrulli*]
fig|80869.163.peg.1570	T4S	5'-nucleotidase [*Paracidovorax citrulli*]
fig|80869.163.peg.2917	T4S	30S ribosomal protein S4 [*Paracidovorax citrulli*]
fig|80869.163.peg.2976	T4S	adenylate kinase [*Paracidovorax citrulli*]
fig|80869.163.peg.546	T4S	hypothetical protein [*Paracidovorax citrulli*]
fig|80869.163.peg.3372	T4S	hypothetical protein [*Paracidovorax citrulli*]
fig|80869.163.peg.813	T4S	type IV pilin protein [*Paracidovorax citrulli*]
fig|80869.163.peg.1457	T4S	BON domain-containing protein [*Paracidovorax citrulli*]
fig|80869.163.peg.1936	T4S	MetQ/NlpA family ABC transporter substrate-binding protein [*Paracidovorax citrulli*]
fig|80869.163.peg.4173	T4S	Bug family tripartite tricarboxylate transporter substrate binding protein [*Paracidovorax citrulli*]
fig|80869.163.peg.31	T4S	tripartite tricarboxylate transporter substrate binding protein [*Paracidovorax citrulli*]
fig|80869.163.peg.1942	T4S	sulfate ABC transporter substrate-binding protein [*Paracidovorax citrulli*]
fig|80869.163.peg.3859	T4S	hypothetical protein [*Paracidovorax citrulli*]
fig|80869.163.peg.3543	T4S	OmpA family protein [*Paracidovorax citrulli*]
fig|80869.163.peg.112	T4S	endolysin [*Paracidovorax citrulli*]
fig|80869.163.peg.2633	T4S	HTH-type transcriptional regulator GltC [*Paracidovorax citrulli*]
fig|80869.163.peg.656	T4S	sigma-54 dependent transcriptional regulator [*Paracidovorax citrulli*]
fig|80869.163.peg.1571	T4S	EF-hand domain-containing protein [*Paracidovorax citrulli*]

*P_citrulli*_IBSBF1521

Prot	Pred	Description
fig|80869.165.peg.1454	T3S	major facilitator superfamily MFS_1 [*Paracidovorax citrulli* AAC00-1]
fig|80869.165.peg.3217	T3S	Ig domain protein, group 1 domain protein [*Paracidovorax citrulli* AAC00-1]
fig|80869.165.peg.4462	T3S	hypothetical protein [*Paracidovorax citrulli*]
fig|80869.165.peg.3372	T3S	acyl-CoA thioesterase [*Paracidovorax citrulli*]
fig|80869.165.peg.1593	T3S	STY0301 family protein [*Paracidovorax citrulli*]
fig|80869.165.peg.1982	T3S	ABC transporter ATP-binding protein [*Paracidovorax citrulli*]
fig|80869.165.peg.1	T3S	DUF72 domain-containing protein [*Paracidovorax citrulli*]
fig|80869.165.peg.1018	T3S	rhodanese-like domain-containing protein [*Paracidovorax citrulli*]
fig|80869.165.peg.1048	T3S	ABC transporter permease [*Paracidovorax citrulli*]
fig|80869.165.peg.1052	T3S	hypothetical protein [*Paracidovorax citrulli*]

Prot	Pred	Description
fig|80869.165.peg.1053	T3S	Metallo-beta-lactamase superfamily protein [*Paracidovorax citrulli*]
fig|80869.165.peg.1065	T3S	Trehalase [*Paracidovorax citrulli*]
fig|80869.165.peg.1085	T3S	hypothetical protein [*Paracidovorax citrulli*]
fig|80869.165.peg.1113	T3S	ABC transporter permease [*Paracidovorax citrulli*]
fig|80869.165.peg.1164	T3S	hypothetical protein [*Paracidovorax citrulli*]
fig|80869.165.peg.1176	T3S	YitT family protein [*Paracidovorax citrulli*]
fig|80869.165.peg.1177	T3S	hypothetical protein [*Paracidovorax citrulli*]
fig|80869.165.peg.1192	T3S	3-methyl-2-oxobutanoate hydroxymethyltransferase [*Paracidovorax citrulli*]
fig|80869.165.peg.1214	T3S	signal recognition particle-docking protein FtsY [*Paracidovorax citrulli*]
fig|80869.165.peg.1224	T3S	MATE family efflux transporter [*Paracidovorax citrulli*]
fig|80869.165.peg.1292	T3S	type III secretion system chaperone [*Paracidovorax citrulli*]
fig|80869.165.peg.1293	T3S	hypothetical protein [*Paracidovorax citrulli*]
fig|80869.165.peg.1320	T3S	LLM class flavin-dependent oxidoreductase [*Paracidovorax citrulli*]
fig|80869.165.peg.1333	T3S	hypothetical protein [*Paracidovorax citrulli*]
fig|80869.165.peg.1347	T3S	ABC transporter permease [*Paracidovorax citrulli*]
fig|80869.165.peg.1348	T3S	ABC transporter permease subunit [*Paracidovorax citrulli*]
fig|80869.165.peg.1356	T3S	NAD-dependent succinate-semialdehyde dehydrogenase [*Paracidovorax citrulli*]
fig|80869.165.peg.1372	T3S	GntR family transcriptional regulator [*Paracidovorax citrulli*]
fig|80869.165.peg.1387	T3S	cytochrome o ubiquinol oxidase subunit IV [*Paracidovorax citrulli*]
fig|80869.165.peg.1421	T3S	multiple monosaccharide ABC transporter permease [*Paracidovorax citrulli*]
fig|80869.165.peg.1454	T4S	major facilitator superfamily MFS_1 [*Paracidovorax citrulli* AAC00-1]
fig|80869.165.peg.3217	T4S	Ig domain protein, group 1 domain protein [*Paracidovorax citrulli* AAC00-1]
fig|80869.165.peg.4462	T4S	hypothetical protein [*Paracidovorax citrulli*]
fig|80869.165.peg.3372	T4S	acyl-CoA thioesterase [*Paracidovorax citrulli*]
fig|80869.165.peg.1593	T4S	STY0301 family protein [*Paracidovorax citrulli*]
fig|80869.165.peg.1982	T4S	ABC transporter ATP-binding protein [*Paracidovorax citrulli*]
fig|80869.165.peg.1702	T4S	Ku protein [*Paracidovorax citrulli*]
fig|80869.165.peg.1207	T4S	ferritin-like domain-containing protein [*Paracidovorax citrulli*]
fig|80869.165.peg.147	T4S	terminase small subunit [*Paracidovorax citrulli*]
fig|80869.165.peg.751	T4S	molybdenum cofactor biosynthesis protein MoaE [*Paracidovorax citrulli*]
fig|80869.165.peg.458	T4S	hypothetical protein [*Paracidovorax citrulli*]
fig|80869.165.peg.196	T4S	isoaspartyl peptidase/L-asparaginase [*Paracidovorax citrulli*]
fig|80869.165.peg.3411	T4S	RNA-binding protein [*Paracidovorax citrulli*]
fig|80869.165.peg.3926	T4S	hypothetical protein Aave_3072 [*Paracidovorax citrulli* AAC00-1]
fig|80869.165.peg.2999	T4S	DUF1795 domain-containing protein [*Paracidovorax citrulli*]
fig|80869.165.peg.247	T4S	XopE/AvrPphe family type III secretion system effector [*Paracidovorax citrulli*]
fig|80869.165.peg.1736	T4S	amino-acid N-acetyltransferase [*Paracidovorax citrulli*]
fig|80869.165.peg.4301	T4S	DUF924 family protein [*Paracidovorax citrulli*]
fig|80869.165.peg.2946	T4S	hypothetical protein [*Paracidovorax citrulli*]
fig|80869.165.peg.3871	T4S	50S ribosomal protein L11 [*Paracidovorax citrulli*]
fig|80869.165.peg.1705	T4S	hypothetical protein [*Paracidovorax citrulli*]
fig|80869.165.peg.1429	T4S	hypothetical protein C8E08_4485 [*Paracidovorax citrulli*]
fig|80869.165.peg.2350	T4S	LysR family transcriptional regulator [Paracidovorax]
fig|80869.165.peg.4215	T4S	SMC-Scp complex subunit ScpB [*Paracidovorax citrulli*]
fig|80869.165.peg.4166	T4S	PP2C family serine/threonine-protein phosphatase [*Paracidovorax citrulli*]

Prot	Pred	Description
fig|80869.165.peg.4500	T4S	hypothetical protein [*Paracidovorax citrulli*]
fig|80869.165.peg.3892	T4S	cryptochrome/photolyase family protein [*Paracidovorax citrulli*]
fig|80869.165.peg.829	T4S	phosphopyruvate hydratase [*Paracidovorax citrulli*]
fig|80869.165.peg.1259	T4S	glutathione-regulated potassium-efflux system protein KefC [*Paracidovorax citrulli*]
fig|80869.165.peg.1878	T4S	hypothetical protein [*Paracidovorax citrulli*]
fig|80869.165.peg.563	T4S	hypothetical protein [*Paracidovorax citrulli*]
fig|80869.165.peg.25	T4S	ATP-binding protein [*Paracidovorax citrulli*]
fig|80869.165.peg.3826	T4S	DUF3025 domain-containing protein [*Paracidovorax citrulli*]
fig|80869.165.peg.4165	T4S	serine/threonine-protein kinase [*Paracidovorax citrulli*]
fig|80869.165.peg.4424	T4S	hypothetical protein [*Paracidovorax citrulli*]
fig|80869.165.peg.3988	T4S	inositol monophosphatase family protein [*Paracidovorax citrulli*]
fig|80869.165.peg.1100	T4S	N-acetylmuramoyl-L-alanine amidase [*Paracidovorax citrulli*]
fig|80869.165.peg.2669	T4S	AraC family transcriptional regulator [*Paracidovorax citrulli*]
fig|80869.165.peg.622	T4S	hypothetical protein [*Paracidovorax citrulli*]
fig|80869.165.peg.2808	T4S	Fic family protein [*Paracidovorax citrulli*]
fig|80869.165.peg.4406	T4S	4-hydroxy-3-methylbut-2-enyl diphosphate reductase [*Paracidovorax citrulli*]
fig|80869.165.peg.4130	T4S	SsrA-binding protein SmpB [*Paracidovorax citrulli*]
fig|80869.165.peg.2534	T4S	F0F1 ATP synthase subunit gamma [*Paracidovorax citrulli*]
fig|80869.165.peg.365	T4S	hypothetical protein [*Paracidovorax citrulli*]
fig|80869.165.peg.3184	T4S	transglycosylase SLT domain-containing protein [*Paracidovorax citrulli*]
fig|80869.165.peg.2690	T4S	type III secretion system outer membrane ring subunit SctC [*Paracidovorax citrulli*]
fig|80869.165.peg.128	T4S	hypothetical protein [*Paracidovorax citrulli*]
fig|80869.165.peg.1322	T4S	5'-nucleotidase [*Paracidovorax citrulli*]
fig|80869.165.peg.1546	T4S	hypothetical protein [*Paracidovorax citrulli*]
fig|80869.165.peg.2634	T4S	30S ribosomal protein S4 [*Paracidovorax citrulli*]
fig|80869.165.peg.3093	T4S	adenylate kinase [*Paracidovorax citrulli*]
fig|80869.165.peg.544	T4S	hypothetical protein [*Paracidovorax citrulli*]
fig|80869.165.peg.3296	T4S	hypothetical protein [*Paracidovorax citrulli*]
fig|80869.165.peg.1904	T4S	type IV pilin protein [*Paracidovorax citrulli*]
fig|80869.165.peg.3846	T4S	hypothetical protein [*Paracidovorax citrulli*]
fig|80869.165.peg.1208	T4S	BON domain-containing protein [*Paracidovorax citrulli*]
fig|80869.165.peg.1721	T4S	MetQ/NlpA family ABC transporter substrate-binding protein [*Paracidovorax citrulli*]
fig|80869.165.peg.4257	T4S	Bug family tripartite tricarboxylate transporter substrate binding protein [*Paracidovorax citrulli*]
fig|80869.165.peg.31	T4S	tripartite tricarboxylate transporter substrate binding protein [*Paracidovorax citrulli*]
fig|80869.165.peg.1728	T4S	sulfate ABC transporter substrate-binding protein [*Paracidovorax citrulli*]
fig|80869.165.peg.3754	T4S	hypothetical protein [*Paracidovorax citrulli*]
fig|80869.165.peg.4302	T4S	OmpA family protein [*Paracidovorax citrulli*]
fig|80869.165.peg.110	T4S	endolysin [*Paracidovorax citrulli*]
fig|80869.165.peg.1750	T4S	sigma-54 dependent transcriptional regulator [*Paracidovorax citrulli*]
fig|80869.165.peg.1323	T4S	EF-hand domain-containing protein [*Paracidovorax citrulli*]

*P_citrulli*_IBSBF1627

Prot	Pred	Description
fig|80869.166.peg.1314	T3S	Major Facilitator Superfamily MFS_1 [*Paracidovorax citrulli* AAC00-1]
fig|80869.166.peg.1893	T3S	Ig domain protein, group 1 domain protein [*Paracidovorax citrulli* AAC00-1]
fig|80869.166.peg.3487	T3S	hypothetical protein [*Paracidovorax citrulli*]
fig|80869.166.peg.4437	T3S	DUF3577 domain-containing protein [*Paracidovorax citrulli*]
fig|80869.166.peg.3592	T3S	acyl-CoA thioesterase [*Paracidovorax citrulli*]
fig|80869.166.peg.4622	T3S	STY0301 family protein [*Paracidovorax citrulli*]
fig|80869.166.peg.3464	T3S	hypothetical protein [*Paracidovorax citrulli*]
fig|80869.166.peg.3179	T3S	ABC transporter ATP-binding protein [*Paracidovorax citrulli*]
fig|80869.166.peg.1015	T3S	Holliday junction resolvase RuvX [*Paracidovorax citrulli*]
fig|80869.166.peg.1024	T3S	bifunctional hydroxymethylpyrimidine kinase/phosphomethylpyrimidine kinase [*Paracidovorax citrulli*]
fig|80869.166.peg.104	T3S	helix-turn-helix domain-containing protein [*Paracidovorax citrulli*]
fig|80869.166.peg.1058	T3S	ribonucleoside-diphosphate reductase subunit alpha [*Paracidovorax citrulli*]
fig|80869.166.peg.1071	T3S	3-deoxy-7-phosphoheptulonate synthase [*Paracidovorax citrulli*]
fig|80869.166.peg.1115	T3S	CysB family HTH-type transcriptional regulator [Comamonadaceae]
fig|80869.166.peg.1126	T3S	2OG-Fe dioxygenase family protein [*Paracidovorax citrulli*]
fig|80869.166.peg.1150	T3S	TetR/AcrR family transcriptional regulator [*Paracidovorax citrulli*]
fig|80869.166.peg.1169	T3S	RDD domain containing protein [*Paracidovorax citrulli* AAC00-1]
fig|80869.166.peg.1183	T3S	YbaN family protein [*Paracidovorax citrulli*]
fig|80869.166.peg.1193	T3S	ATP-binding protein [*Paracidovorax citrulli*]
fig|80869.166.peg.1200	T3S	hypothetical protein CQB05_09585 [*Paracidovorax citrulli*]
fig|80869.166.peg.1217	T3S	NAD-dependent succinate-semialdehyde dehydrogenase [*Paracidovorax citrulli*]
fig|80869.166.peg.122	T3S	ABC transporter transmembrane domain-containing protein [*Paracidovorax citrulli*]
fig|80869.166.peg.1232	T3S	transcriptional regulator, GntR family [*Paracidovorax citrulli* AAC00-1]
fig|80869.166.peg.1247	T3S	cytochrome o ubiquinol oxidase subunit IV [*Paracidovorax citrulli*]
fig|80869.166.peg.1280	T3S	multiple monosaccharide ABC transporter permease [*Paracidovorax citrulli*]
fig|80869.166.peg.1286	T3S	SDR family oxidoreductase [*Paracidovorax citrulli*]
fig|80869.166.peg.1287	T3S	sugar lactone lactonase YvrE [*Paracidovorax citrulli*]
fig|80869.166.peg.1295	T3S	GAF domain-containing sensor histidine kinase [*Paracidovorax citrulli*]
fig|80869.166.peg.130	T3S	NADH-quinone oxidoreductase subunit NuoI [Comamonadaceae]
fig|80869.166.peg.1302	T3S	major facilitator superfamily MFS_1 [*Paracidovorax citrulli* AAC00-1]
fig|80869.166.peg.1304	T3S	redox-sensitive transcriptional activator SoxR [*Paracidovorax citrulli*]
fig|80869.166.peg.1308	T3S	DHHA1 domain-containing protein [*Paracidovorax citrulli*]
fig|80869.166.peg.1314	T4S	major facilitator superfamily MFS_1 [*Paracidovorax citrulli* AAC00-1]
fig|80869.166.peg.1893	T4S	Ig domain protein, group 1 domain protein [*Paracidovorax citrulli* AAC00-1]
fig|80869.166.peg.3487	T4S	hypothetical protein [*Paracidovorax citrulli*]
fig|80869.166.peg.4437	T4S	DUF3577 domain-containing protein [*Paracidovorax citrulli*]
fig|80869.166.peg.3592	T4S	acyl-CoA thioesterase [*Paracidovorax citrulli*]
fig|80869.166.peg.4622	T4S	STY0301 family protein [*Paracidovorax citrulli*]
fig|80869.166.peg.3464	T4S	hypothetical protein [*Paracidovorax citrulli*]
fig|80869.166.peg.3179	T4S	ABC transporter ATP-binding protein [*Paracidovorax citrulli*]
fig|80869.166.peg.4197	T4S	Ku protein [*Paracidovorax citrulli*]
fig|80869.166.peg.3209	T4S	terminase small subunit [*Paracidovorax citrulli*]
fig|80869.166.peg.156	T4S	molybdenum cofactor biosynthesis protein MoaE [*Paracidovorax citrulli*]

Prot	Pred	Description
fig|80869.166.peg.636	T4S	ferritin-like domain-containing protein [*Paracidovorax citrulli*]
fig|80869.166.peg.4126	T4S	isoaspartyl peptidase/L-asparaginase [*Paracidovorax citrulli*]
fig|80869.166.peg.5021	T4S	hypothetical protein CQB05_06935 [*Paracidovorax citrulli*]
fig|80869.166.peg.3449	T4S	RNA-binding protein [*Paracidovorax citrulli*]
fig|80869.166.peg.4503	T4S	hypothetical protein [*Paracidovorax citrulli*]
fig|80869.166.peg.1815	T4S	hypothetical protein Aave_3072 [*Paracidovorax citrulli* AAC00-1]
fig|80869.166.peg.2016	T4S	DUF1795 domain-containing protein [*Paracidovorax citrulli*]
fig|80869.166.peg.4333	T4S	type III secretion system outer membrane ring subunit SctC [*Paracidovorax citrulli*]
fig|80869.166.peg.4592	T4S	amino-acid N-acetyltransferase [*Paracidovorax citrulli*]
fig|80869.166.peg.3324	T4S	DUF924 family protein [*Paracidovorax citrulli*]
fig|80869.166.peg.1559	T4S	hypothetical protein [*Paracidovorax citrulli*]
fig|80869.166.peg.3825	T4S	50S ribosomal protein L11 [*Paracidovorax citrulli*]
fig|80869.166.peg.4200	T4S	hypothetical protein [*Paracidovorax citrulli*]
fig|80869.166.peg.1288	T4S	hypothetical protein C8E08_4485 [*Paracidovorax citrulli*]
fig|80869.166.peg.1396	T4S	PP2C family serine/threonine-protein phosphatase [*Paracidovorax citrulli*]
fig|80869.166.peg.689	T4S	glutathione-regulated potassium-efflux system protein KefC [*Paracidovorax citrulli*]
fig|80869.166.peg.4533	T4S	hypothetical protein [*Paracidovorax citrulli*]
fig|80869.166.peg.3844	T4S	cryptochrome/photolyase family protein [*Paracidovorax citrulli*]
fig|80869.166.peg.77	T4S	phosphopyruvate hydratase [*Paracidovorax citrulli*]
fig|80869.166.peg.1454	T4S	hypothetical protein [*Paracidovorax citrulli*]
fig|80869.166.peg.3819	T4S	hypothetical protein [*Paracidovorax citrulli*]
fig|80869.166.peg.2706	T4S	ATP-binding protein [*Paracidovorax citrulli*]
fig|80869.166.peg.2250	T4S	DUF3025 domain-containing protein [*Paracidovorax citrulli*]
fig|80869.166.peg.1395	T4S	serine/threonine-protein kinase [*Paracidovorax citrulli*]
fig|80869.166.peg.3681	T4S	inositol monophosphatase family protein [*Paracidovorax citrulli*]
fig|80869.166.peg.929	T4S	N-acetylmuramoyl-L-alanine amidase [*Paracidovorax citrulli*]
fig|80869.166.peg.2337	T4S	AraC family transcriptional regulator [*Paracidovorax citrulli*]
fig|80869.166.peg.3918	T4S	SMC-Scp complex subunit ScpB [*Paracidovorax citrulli*]
fig|80869.166.peg.2581	T4S	hypothetical protein [*Paracidovorax citrulli*]
fig|80869.166.peg.309	T4S	Fic family protein [*Paracidovorax citrulli*]
fig|80869.166.peg.4912	T4S	recombinase family protein [Pseudomonadota]
fig|80869.166.peg.4416	T4S	4-hydroxy-3-methylbut-2-enyl diphosphate reductase [*Paracidovorax citrulli*]
fig|80869.166.peg.3877	T4S	SsrA-binding protein SmpB [*Paracidovorax citrulli*]
fig|80869.166.peg.3128	T4S	hypothetical protein [*Paracidovorax citrulli*]
fig|80869.166.peg.3407	T4S	F0F1 ATP synthase subunit gamma [*Paracidovorax citrulli*]
fig|80869.166.peg.3959	T4S	hypothetical protein [*Paracidovorax citrulli*]
fig|80869.166.peg.2442	T4S	hypothetical protein [*Paracidovorax citrulli*]
fig|80869.166.peg.3374	T4S	transglycosylase SLT domain-containing protein [*Paracidovorax citrulli*]
fig|80869.166.peg.793	T4S	hypothetical protein [*Paracidovorax citrulli*]
fig|80869.166.peg.3227	T4S	hypothetical protein [*Paracidovorax citrulli*]
fig|80869.166.peg.752	T4S	5'-nucleotidase [*Paracidovorax citrulli*]
fig|80869.166.peg.2372	T4S	30S ribosomal protein S4 [*Paracidovorax citrulli*]
fig|80869.166.peg.4056	T4S	adenylate kinase [*Paracidovorax citrulli*]
fig|80869.166.peg.3800	T4S	hypothetical protein [*Paracidovorax citrulli*]
fig|80869.166.peg.1427	T4S	type IV pilin protein [*Paracidovorax citrulli*]

Prot	Pred	Description
fig|80869.166.peg.2230	T4S	hypothetical protein [*Paracidovorax citrulli*]
fig|80869.166.peg.637	T4S	BON domain-containing protein [*Paracidovorax citrulli*]
fig|80869.166.peg.4789	T4S	MetQ/NlpA family ABC transporter substrate-binding protein [*Paracidovorax citrulli*]
fig|80869.166.peg.3630	T4S	Bug family tripartite tricarboxylate transporter substrate binding protein [*Paracidovorax citrulli*]
fig|80869.166.peg.2910	T4S	hypothetical protein [*Paracidovorax citrulli*]
fig|80869.166.peg.4584	T4S	sulfate ABC transporter substrate-binding protein [*Paracidovorax citrulli*]
fig|80869.166.peg.1605	T4S	hypothetical protein [*Paracidovorax citrulli*]
fig|80869.166.peg.3323	T4S	OmpA family protein [*Paracidovorax citrulli*]
fig|80869.166.peg.3245	T4S	endolysin [*Paracidovorax citrulli*]
fig|80869.166.peg.2170	T4S	DNA-binding transcriptional LysR family regulator [*Paracidovorax citrulli*]
fig|80869.166.peg.4242	T4S	sigma-54 dependent transcriptional regulator [*Paracidovorax citrulli*]
fig|80869.166.peg.753	T4S	EF-hand domain-containing protein [*Paracidovorax citrulli*]

*P_citrulli_*NWBS074

Prot	Pred	Description
fig|80869.179.peg.1065	T3S	hypothetical protein [*Paracidovorax citrulli*]
fig|80869.179.peg.1177	T3S	major facilitator superfamily MFS_1 [*Paracidovorax citrulli* AAC00-1]
fig|80869.179.peg.3286	T3S	Ig domain protein, group 1 domain protein [*Paracidovorax citrulli* AAC00-1]
fig|80869.179.peg.3767	T3S	DUF3577 domain-containing protein [*Paracidovorax citrulli*]
fig|80869.179.peg.983	T3S	acyl-CoA thioesterase [*Paracidovorax citrulli*]
fig|80869.179.peg.1264	T3S	STY0301 family protein [*Paracidovorax citrulli*]
fig|80869.179.peg.1855	T3S	ABC transporter ATP-binding protein [*Paracidovorax citrulli*]
fig|80869.179.peg.1378	T3S	Ku protein [*Paracidovorax citrulli*]
fig|80869.179.peg.2533	T3S	terminase small subunit [*Paracidovorax citrulli*]
fig|80869.179.peg.1001	T3S	carboxyl transferase domain-containing protein [*Paracidovorax citrulli*]
fig|80869.179.peg.1009	T3S	excinuclease ABC subunit UvrA [*Paracidovorax citrulli*]
fig|80869.179.peg.1014	T3S	DUF2169 domain-containing protein [*Paracidovorax citrulli*]
fig|80869.179.peg.1035	T3S	hypothetical protein Aave_2148 [*Paracidovorax citrulli* AAC00-1]
fig|80869.179.peg.1080	T3S	NAD-dependent succinate-semialdehyde dehydrogenase [*Paracidovorax citrulli*]
fig|80869.179.peg.1095	T3S	transcriptional regulator, GntR family [*Paracidovorax citrulli* AAC00-1]
fig|80869.179.peg.110	T3S	ABC transporter permease [*Paracidovorax citrulli*]
fig|80869.179.peg.111	T3S	ABC transporter permease [*Paracidovorax citrulli*]
fig|80869.179.peg.1110	T3S	cytochrome o ubiquinol oxidase subunit IV [*Paracidovorax citrulli*]
fig|80869.179.peg.1144	T3S	multiple monosaccharide ABC transporter permease [*Paracidovorax citrulli*]
fig|80869.179.peg.1150	T3S	SDR family NAD(P)-dependent oxidoreductase [*Paracidovorax citrulli*]
fig|80869.179.peg.1151	T3S	SMP-30/gluconolactonase/LRE family protein [*Paracidovorax citrulli*]
fig|80869.179.peg.1158	T3S	GAF domain-containing sensor histidine kinase [*Paracidovorax citrulli*]
fig|80869.179.peg.1165	T3S	MFS transporter [*Paracidovorax citrulli*]
fig|80869.179.peg.1167	T3S	redox-sensitive transcriptional activator SoxR [*Paracidovorax citrulli*]
fig|80869.179.peg.1171	T3S	DHH family phosphoesterase [*Paracidovorax citrulli*]
fig|80869.179.peg.1181	T3S	Murein DD-endopeptidase MepM [*Paracidovorax citrulli*]
fig|80869.179.peg.1184	T3S	esterase/lipase/thioesterase family protein [*Paracidovorax citrulli* AAC00-1]
fig|80869.179.peg.1196	T3S	glutamine--tRNA ligase/YqeY domain fusion protein [*Paracidovorax citrulli*]
fig|80869.179.peg.1201	T3S	class III extradiol ring-cleavage dioxygenase [*Paracidovorax citrulli*]

Prot	Pred	Description
fig|80869.179.peg.1240	T3S	hypothetical protein [*Paracidovorax citrulli*]
fig|80869.179.peg.125	T3S	hypothetical protein [*Paracidovorax citrulli*]
fig|80869.179.peg.1257	T3S	KGG domain-containing protein [*Paracidovorax citrulli*]
fig|80869.179.peg.1304	T3S	PAS domain-containing sensor histidine kinase [*Paracidovorax citrulli*]
fig|80869.179.peg.1338	T3S	EAL domain-containing protein [*Paracidovorax citrulli*]
fig|80869.179.peg.142	T3S	luciferase family protein [*Paracidovorax citrulli* AAC00-1]
fig|80869.179.peg.1462	T3S	TonB-dependent receptor [*Paracidovorax citrulli*]
fig|80869.179.peg.1473	T3S	D-serine/D-alanine/glycine transporter [*Paracidovorax citrulli*]
fig|80869.179.peg.1474	T3S	hypothetical protein [*Paracidovorax citrulli*]
fig|80869.179.peg.1487	T3S	Polyphosphate kinase [*Paracidovorax citrulli* AAC00-1]
fig|80869.179.peg.1492	T3S	phosphate ABC transporter permease PstC [*Paracidovorax citrulli*]
fig|80869.179.peg.1521	T3S	gephyrin-like molybdotransferase Glp [*Paracidovorax citrulli*]
fig|80869.179.peg.1524	T3S	GTP cyclohydrolase subunit MoaA [*Paracidovorax citrulli* AAC00-1]
fig|80869.179.peg.1535	T3S	translesion DNA synthesis-associated protein ImuA [*Paracidovorax citrulli*]
fig|80869.179.peg.1539	T3S	pseudouridine synthase [*Paracidovorax citrulli*]
fig|80869.179.peg.1560	T3S	hypothetical protein CQB05_01060 [*Paracidovorax citrulli*]
fig|80869.179.peg.1570	T3S	DEAD/DEAH box helicase [*Paracidovorax citrulli*]
fig|80869.179.peg.1612	T3S	hypothetical protein [*Paracidovorax citrulli*]
fig|80869.179.peg.168	T3S	hypothetical protein [*Paracidovorax citrulli*]
fig|80869.179.peg.1687	T3S	pyridoxamine 5'-phosphate oxidase [*Paracidovorax citrulli*]
fig|80869.179.peg.169	T3S	type III secretion system chaperone [*Paracidovorax citrulli*]
fig|80869.179.peg.1698	T3S	transcriptional repressor [*Paracidovorax citrulli*]
fig|80869.179.peg.1700	T3S	TetR/AcrR family transcriptional regulator [*Paracidovorax citrulli*]
fig|80869.179.peg.1719	T3S	EVE domain-containing protein [*Paracidovorax citrulli*]
fig|80869.179.peg.1730	T3S	XopAP family type III secretion system effector [*Paracidovorax citrulli*]
fig|80869.179.peg.1823	T3S	glycoside hydrolase family 5 protein [*Paracidovorax citrulli*]
fig|80869.179.peg.1835	T3S	protein of unknown function UPF0061 [*Paracidovorax citrulli* AAC00-1]
fig|80869.179.peg.1914	T3S	BON domain-containing protein [*Paracidovorax citrulli*]
fig|80869.179.peg.1915	T3S	SulP family inorganic anion transporter [*Paracidovorax citrulli*]
fig|80869.179.peg.1923	T3S	NAD(P)/FAD-dependent oxidoreductase [*Paracidovorax citrulli*]
fig|80869.179.peg.1925	T3S	tRNA (guanosine(46)-N7)-methyltransferase TrmB [*Paracidovorax citrulli*]
fig|80869.179.peg.1985	T3S	hypothetical protein [*Paracidovorax citrulli*]
fig|80869.179.peg.1992	T3S	ATP-binding protein [*Paracidovorax citrulli*]
fig|80869.179.peg.2002	T3S	YbaN family protein [*Paracidovorax citrulli*]
fig|80869.179.peg.2016	T3S	RDD domain containing protein [*Paracidovorax citrulli* AAC00-1]
fig|80869.179.peg.2035	T3S	TetR/AcrR family transcriptional regulator [*Paracidovorax citrulli*]
fig|80869.179.peg.2058	T3S	2OG-Fe dioxygenase family protein [*Paracidovorax citrulli*]
fig|80869.179.peg.2069	T3S	CysB family HTH-type transcriptional regulator [Comamonadaceae]
fig|80869.179.peg.2124	T3S	DUF4230 domain-containing protein [*Paracidovorax citrulli*]
fig|80869.179.peg.2158	T3S	ABC transporter permease [*Paracidovorax citrulli*]
fig|80869.179.peg.2228	T3S	hypothetical protein [*Paracidovorax citrulli*]
fig|80869.179.peg.2230	T3S	L-proline dehydrogenase /delta-1-pyrroline-5-carboxylate dehydrogenase [*Paracidovorax citrulli*]
fig|80869.179.peg.2274	T3S	AlpA family transcriptional regulator [*Paracidovorax citrulli*]
fig|80869.179.peg.2291	T3S	hypothetical protein [*Paracidovorax citrulli*]
fig|80869.179.peg.2299	T3S	thioredoxin family protein [*Paracidovorax citrulli*]
fig|80869.179.peg.2301	T3S	cytochrome c553-like protein [*Paracidovorax citrulli* AAC00-1]

Prot	Pred	Description
fig|80869.179.peg.2317	T3S	ThiF family adenylyltransferase [*Paracidovorax citrulli*]
fig|80869.179.peg.2321	T3S	lipid A export permease/ATP-binding protein MsbA [*Paracidovorax citrulli*]
fig|80869.179.peg.2339	T3S	DUF72 domain-containing protein [*Paracidovorax citrulli*]
fig|80869.179.peg.2342	T3S	DUF1624 domain-containing protein [*Paracidovorax citrulli*]
fig|80869.179.peg.2343	T3S	glutamate--tRNA ligase [*Paracidovorax citrulli*]
fig|80869.179.peg.236	T3S	MATE family efflux transporter [*Paracidovorax citrulli*]
fig|80869.179.peg.2376	T3S	UDP-3-O-(3-hydroxymyristoyl)glucosamine N-acyltransferase [*Paracidovorax citrulli*]
fig|80869.179.peg.2391	T3S	MlaD family protein [*Paracidovorax citrulli*]
fig|80869.179.peg.2395	T3S	YitT family protein [*Paracidovorax citrulli*]
fig|80869.179.peg.2406	T3S	P1 family peptidase [*Paracidovorax citrulli*]
fig|80869.179.peg.2411	T3S	ATP synthase F1 subunit epsilon [*Paracidovorax citrulli*]
fig|80869.179.peg.2425	T3S	structural protein P5 [*Paracidovorax citrulli*]
fig|80869.179.peg.2428	T3S	hypothetical protein [*Paracidovorax citrulli*]
fig|80869.179.peg.247	T3S	signal recognition particle-docking protein FtsY [*Paracidovorax citrulli*]
fig|80869.179.peg.2536	T3S	hypothetical protein [*Paracidovorax citrulli*]
fig|80869.179.peg.2607	T3S	structural protein P5 [*Paracidovorax citrulli*]
fig|80869.179.peg.2637	T3S	hypothetical protein Aave_1606 [*Paracidovorax citrulli* AAC00-1]
fig|80869.179.peg.2647	T3S	polyhydroxyalkanoate synthesis repressor PhaR [*Paracidovorax citrulli*]
fig|80869.179.peg.2649	T3S	type 1 glutamine amidotransferase domain-containing protein [*Paracidovorax citrulli*]
fig|80869.179.peg.268	T3S	3-methyl-2-oxobutanoate hydroxymethyltransferase [*Paracidovorax citrulli*]
fig|80869.179.peg.2697	T3S	SDR family NAD(P)-dependent oxidoreductase [*Paracidovorax citrulli*]
fig|80869.179.peg.2727	T3S	cation-translocating P-type ATPase [*Paracidovorax citrulli*]
fig|80869.179.peg.2735	T3S	malonyl-CoA synthase [*Paracidovorax citrulli*]
fig|80869.179.peg.2754	T3S	glycine betaine/L-proline ABC transporter permease ProW [*Paracidovorax citrulli*]
fig|80869.179.peg.2796	T3S	farnesyl-diphosphate farnesyltransferase [*Paracidovorax citrulli* AAC00-1]
fig|80869.179.peg.2799	T3S	DUF2069 domain-containing protein [*Paracidovorax citrulli*]
fig|80869.179.peg.2824	T3S	GTPase HflX [*Paracidovorax citrulli*]
fig|80869.179.peg.283	T3S	hypothetical protein [*Paracidovorax citrulli*]
fig|80869.179.peg.2839	T3S	protein-L-isoaspartate(D-aspartate) O-methyltransferase [*Paracidovorax citrulli*]
fig|80869.179.peg.284	T3S	YitT family protein [*Paracidovorax citrulli*]
fig|80869.179.peg.2868	T3S	CaiB/BaiF CoA-transferase family protein [*Paracidovorax citrulli*]
fig|80869.179.peg.2901	T3S	VF530 family DNA-binding protein [*Paracidovorax citrulli*]
fig|80869.179.peg.2909	T3S	DNA internalization-related competence protein ComEC/Rec2 [*Paracidovorax citrulli*]
fig|80869.179.peg.2915	T3S	pyridoxal kinase PdxY [*Paracidovorax citrulli*]
fig|80869.179.peg.2941	T3S	hypothetical protein [*Paracidovorax citrulli*]
fig|80869.179.peg.2945	T3S	diguanylate phosphodiesterase [*Paracidovorax citrulli* AAC00-1]
fig|80869.179.peg.296	T3S	hypothetical protein [*Paracidovorax citrulli*]
fig|80869.179.peg.2966	T3S	helix-turn-helix domain-containing protein [*Paracidovorax citrulli*]
fig|80869.179.peg.2984	T3S	ABC transporter transmembrane domain-containing protein [*Paracidovorax citrulli*]
fig|80869.179.peg.2992	T3S	NADH-quinone oxidoreductase subunit NuoI [*Paracidovorax citrulli*]
fig|80869.179.peg.3012	T3S	hypothetical protein [*Paracidovorax citrulli*]
fig|80869.179.peg.3024	T3S	molybdopterin converting factor subunit 1 [*Paracidovorax citrulli*]
fig|80869.179.peg.3039	T3S	nucleotide exchange factor GrpE [*Paracidovorax citrulli*]
fig|80869.179.peg.3057	T3S	LON peptidase substrate-binding domain-containing protein [*Paracidovorax citrulli*]

Prot	Pred	Description
fig|80869.179.peg.3064	T3S	lysine--tRNA ligase [*Paracidovorax citrulli*]
fig|80869.179.peg.3065	T3S	Phytochrome-like protein cph2 [*Paracidovorax citrulli*]
fig|80869.179.peg.3117	T3S	tryptophan 2,3-dioxygenase [*Paracidovorax citrulli*]
fig|80869.179.peg.3126	T3S	neutral zinc metallopeptidase [*Paracidovorax citrulli*]
fig|80869.179.peg.3164	T3S	glycerate kinase [*Paracidovorax citrulli*]
fig|80869.179.peg.3180	T3S	NCS1 family nucleobase:cation symporter-1 [*Paracidovorax citrulli*]
fig|80869.179.peg.3182	T3S	GntR family transcriptional regulator [*Paracidovorax citrulli*]
fig|80869.179.peg.3201	T3S	DMT family transporter [*Paracidovorax citrulli*]
fig|80869.179.peg.3245	T3S	PelD GGDEF domain-containing protein [*Paracidovorax citrulli*]
fig|80869.179.peg.3282	T3S	protein of unknown function DUF1415 [*Paracidovorax citrulli* AAC00-1]
fig|80869.179.peg.3292	T3S	penicillin-binding protein 1A [*Paracidovorax citrulli*]
fig|80869.179.peg.33	T3S	TRAP transporter small permease [*Paracidovorax citrulli*]
fig|80869.179.peg.3316	T3S	peptidoglycan-binding domain-containing protein [*Paracidovorax citrulli*]
fig|80869.179.peg.3319	T3S	NCS1 family nucleobase:cation symporter-1 [*Paracidovorax citrulli*]
fig|80869.179.peg.3351	T3S	ABC transporter ATP-binding protein [*Paracidovorax citrulli*]
fig|80869.179.peg.3384	T3S	Holliday junction resolvase RuvX [*Paracidovorax citrulli*]
fig|80869.179.peg.3393	T3S	bifunctional hydroxymethylpyrimidine kinase/phosphomethylpyrimidine kinase [*Paracidovorax citrulli*]
fig|80869.179.peg.3427	T3S	ribonucleoside-diphosphate reductase subunit alpha [*Paracidovorax citrulli*]
fig|80869.179.peg.3439	T3S	3-deoxy-7-phosphoheptulonate synthase [*Paracidovorax citrulli*]
fig|80869.179.peg.3485	T3S	GTP-binding protein [*Paracidovorax citrulli*]
fig|80869.179.peg.3497	T3S	chromate efflux transporter [*Paracidovorax citrulli*]
fig|80869.179.peg.3504	T3S	M48 family metallopeptidase [*Paracidovorax citrulli*]
fig|80869.179.peg.3519	T3S	DNA-3-methyladenine glycosylase I [*Paracidovorax citrulli*]
fig|80869.179.peg.3520	T3S	putative zinc protease protein [*Paracidovorax citrulli* AAC00-1]
fig|80869.179.peg.3597	T3S	FMN-dependent NADH-azoreductase [*Paracidovorax citrulli*]
fig|80869.179.peg.3643	T3S	NarK family nitrate/nitrite MFS transporter [*Paracidovorax citrulli*]
fig|80869.179.peg.3645	T3S	transcriptional regulator, AraC family [*Paracidovorax citrulli* AAC00-1]
fig|80869.179.peg.3658	T3S	FAD-linked oxidase C-terminal domain-containing protein [*Paracidovorax citrulli*]
fig|80869.179.peg.3703	T3S	pyrroline-5-carboxylate reductase [*Paracidovorax citrulli*]
fig|80869.179.peg.3705	T3S	glycosyltransferase involved in cell wall biosynthesis [*Paracidovorax citrulli*]
fig|80869.179.peg.3719	T3S	uracil-DNA glycosylase [*Paracidovorax citrulli*]
fig|80869.179.peg.3757	T3S	HNH endonuclease [*Paracidovorax citrulli*]
fig|80869.179.peg.3777	T3S	hypothetical protein [Pseudomonadota]
fig|80869.179.peg.3843	T3S	hypothetical protein [*Paracidovorax citrulli*]
fig|80869.179.peg.3849	T3S	hypothetical protein [*Paracidovorax citrulli*]
fig|80869.179.peg.3860	T3S	hypothetical protein [*Paracidovorax citrulli*]
fig|80869.179.peg.3861	T3S	hypothetical protein [*Paracidovorax citrulli*]
fig|80869.179.peg.3865	T3S	hypothetical protein Aave_0457 [*Paracidovorax citrulli* AAC00-1]
fig|80869.179.peg.3893	T3S	DUF4139 domain-containing protein [*Paracidovorax citrulli*]
fig|80869.179.peg.3900	T3S	hypothetical protein [*Paracidovorax citrulli*]
fig|80869.179.peg.3905	T3S	TspO/MBR family protein [*Paracidovorax citrulli*]
fig|80869.179.peg.1065	T4S	hypothetical protein [*Paracidovorax citrulli*]
fig|80869.179.peg.1177	T4S	major facilitator superfamily MFS_1 [*Paracidovorax citrulli* AAC00-1]
fig|80869.179.peg.3286	T4S	Ig domain protein, group 1 domain protein [*Paracidovorax citrulli* AAC00-1]
fig|80869.179.peg.3767	T4S	DUF3577 domain-containing protein [*Paracidovorax citrulli*]

Prot	Pred	Description
fig|80869.179.peg.983	T4S	acyl-CoA thioesterase [*Paracidovorax citrulli*]
fig|80869.179.peg.1264	T4S	STY0301 family protein [*Paracidovorax citrulli*]
fig|80869.179.peg.1855	T4S	ABC transporter ATP-binding protein [*Paracidovorax citrulli*]
fig|80869.179.peg.1378	T4S	Ku protein [*Paracidovorax citrulli*]
fig|80869.179.peg.3018	T4S	molybdenum cofactor biosynthesis protein MoaE [*Paracidovorax citrulli*]
fig|80869.179.peg.254	T4S	bacterioferritin [*Paracidovorax citrulli*]
fig|80869.179.peg.2582	T4S	isoaspartyl peptidase/L-asparaginase family protein [*Paracidovorax citrulli*]
fig|80869.179.peg.356	T4S	abortive infection system antitoxin AbiGi family protein [*Paracidovorax citrulli*]
fig|80869.179.peg.1027	T4S	RNA-binding protein [*Paracidovorax citrulli*]
fig|80869.179.peg.1561	T4S	hypothetical protein [*Paracidovorax citrulli*]
fig|80869.179.peg.1969	T4S	hypothetical protein Aave_3072 [*Paracidovorax citrulli* AAC00-1]
fig|80869.179.peg.1725	T4S	DUF1795 domain-containing protein [*Paracidovorax citrulli*]
fig|80869.179.peg.3846	T4S	type III secretion system outer membrane ring subunit SctC [*Paracidovorax citrulli*]
fig|80869.179.peg.1042	T4S	hypothetical protein [*Paracidovorax citrulli*]
fig|80869.179.peg.1414	T4S	amino-acid N-acetyltransferase [Paracidovorax]
fig|80869.179.peg.4577	T4S	DUF924 family protein [*Paracidovorax citrulli*]
fig|80869.179.peg.424	T4S	hypothetical protein [*Paracidovorax citrulli*]
fig|80869.179.peg.4639	T4S	50S ribosomal protein L11 [*Paracidovorax citrulli*]
fig|80869.179.peg.1381	T4S	hypothetical protein [*Paracidovorax citrulli*]
fig|80869.179.peg.1152	T4S	conserved hypothetical protein [*Paracidovorax citrulli* AAC00-1]
fig|80869.179.peg.577	T4S	PP2C family serine/threonine-protein phosphatase [*Paracidovorax citrulli*]
fig|80869.179.peg.201	T4S	glutathione-regulated potassium-efflux system protein KefC [*Paracidovorax citrulli*]
fig|80869.179.peg.4261	T4S	hypothetical protein [*Paracidovorax citrulli*]
fig|80869.179.peg.4658	T4S	cryptochrome/photolyase family protein [*Paracidovorax citrulli*]
fig|80869.179.peg.2939	T4S	phosphopyruvate hydratase [*Paracidovorax citrulli*]
fig|80869.179.peg.634	T4S	hypothetical protein [*Paracidovorax citrulli*]
fig|80869.179.peg.2168	T4S	hypothetical protein [*Paracidovorax citrulli*]
fig|80869.179.peg.2363	T4S	AAA family ATPase [*Paracidovorax citrulli*]
fig|80869.179.peg.4871	T4S	DUF3025 domain-containing protein [*Paracidovorax citrulli*]
fig|80869.179.peg.576	T4S	serine/threonine-protein kinase [*Paracidovorax citrulli*]
fig|80869.179.peg.950	T4S	inositol monophosphatase family protein [*Paracidovorax citrulli*]
fig|80869.179.peg.4946	T4S	N-acetylmuramoyl-L-alanine amidase [*Paracidovorax citrulli*]
fig|80869.179.peg.3713	T4S	AraC family transcriptional regulator [*Paracidovorax citrulli*]
fig|80869.179.peg.2836	T4S	SMC-Scp complex subunit ScpB [*Paracidovorax citrulli*]
fig|80869.179.peg.2226	T4S	hypothetical protein [*Paracidovorax citrulli*]
fig|80869.179.peg.4541	T4S	Fic family protein [*Paracidovorax citrulli*]
fig|80869.179.peg.349	T4S	4-hydroxy-3-methylbut-2-enyl diphosphate reductase [*Paracidovorax citrulli*]
fig|80869.179.peg.2286	T4S	SsrA-binding protein SmpB [*Paracidovorax citrulli*]
fig|80869.179.peg.1806	T4S	hypothetical protein [*Paracidovorax citrulli*]
fig|80869.179.peg.3955	T4S	F0F1 ATP synthase subunit gamma [*Paracidovorax citrulli*]
fig|80869.179.peg.798	T4S	hypothetical protein [*Paracidovorax citrulli*]
fig|80869.179.peg.2809	T4S	hypothetical protein [*Paracidovorax citrulli*]
fig|80869.179.peg.1515	T4S	transglycosylase SLT domain-containing protein [*Paracidovorax citrulli*]
fig|80869.179.peg.5083	T4S	hypothetical protein [*Paracidovorax citrulli*]
fig|80869.179.peg.2514	T4S	hypothetical protein [*Paracidovorax citrulli*]

Prot	Pred	Description
fig|80869.179.peg.140	T4S	5'-nucleotidase [*Paracidovorax citrulli*]
fig|80869.179.peg.3678	T4S	30S ribosomal protein S4 [*Paracidovorax citrulli*]
fig|80869.179.peg.1424	T4S	adenylate kinase [*Paracidovorax citrulli*]
fig|80869.179.peg.2283	T4S	DUF4124 domain-containing protein [*Paracidovorax citrulli*]
fig|80869.179.peg.2149	T4S	hypothetical protein [*Paracidovorax citrulli*]
fig|80869.179.peg.607	T4S	type IV pilin protein [*Paracidovorax citrulli*]
fig|80869.179.peg.4851	T4S	hypothetical protein [*Paracidovorax citrulli*]
fig|80869.179.peg.253	T4S	BON domain-containing protein [*Paracidovorax citrulli*]
fig|80869.179.peg.1400	T4S	MetQ/NlpA family ABC transporter substrate-binding protein [*Paracidovorax citrulli*]
fig|80869.179.peg.4710	T4S	Bug family tripartite tricarboxylate transporter substrate binding protein [*Paracidovorax citrulli*]
fig|80869.179.peg.1595	T4S	hypothetical protein [*Paracidovorax citrulli*]
fig|80869.179.peg.1406	T4S	sulfate ABC transporter substrate-binding protein [*Paracidovorax citrulli*]
fig|80869.179.peg.374	T4S	hypothetical protein [*Paracidovorax citrulli*]
fig|80869.179.peg.4578	T4S	OmpA family protein [*Paracidovorax citrulli*]
fig|80869.179.peg.2496	T4S	glycoside hydrolase family protein [*Paracidovorax citrulli*]
fig|80869.179.peg.4029	T4S	DNA-binding transcriptional LysR family regulator [*Paracidovorax citrulli*]
fig|80869.179.peg.767	T4S	sigma-54 dependent transcriptional regulator [*Paracidovorax citrulli*]
fig|80869.179.peg.139	T4S	EF-hand domain-containing protein [*Paracidovorax citrulli*]

*P_citrulli_*NWBS107

Prot	Pred	Description
fig|80869.180.peg.2566	T3S	major facilitator superfamily MFS_1 [*Paracidovorax citrulli* AAC00-1]
fig|80869.180.peg.2677	T3S	hypothetical protein [*Paracidovorax citrulli*]
fig|80869.180.peg.681	T3S	Ig domain protein, group 1 domain protein [*Paracidovorax citrulli* AAC00-1]
fig|80869.180.peg.2762	T3S	acyl-CoA thioesterase [*Paracidovorax citrulli*]
fig|80869.180.peg.2171	T3S	STY0301 family protein [*Paracidovorax citrulli*]
fig|80869.180.peg.1276	T3S	XopE/AvrPphe family type III secretion system effector [*Paracidovorax citrulli*]
fig|80869.180.peg.2019	T3S	ABC transporter ATP-binding protein [*Paracidovorax citrulli*]
fig|80869.180.peg.2280	T3S	Ku protein [*Paracidovorax citrulli*]
fig|80869.180.peg.2284	T3S	hypothetical protein [*Paracidovorax citrulli*]
fig|80869.180.peg.1005	T3S	helix-turn-helix domain-containing protein [*Paracidovorax citrulli*]
fig|80869.180.peg.1014	T3S	N-acetylmuramoyl-L-alanine amidase [*Paracidovorax citrulli*]
fig|80869.180.peg.1026	T3S	HDOD domain-containing protein [*Paracidovorax citrulli*]
fig|80869.180.peg.1030	T3S	hypothetical protein [*Paracidovorax citrulli*]
fig|80869.180.peg.1056	T3S	pyridoxal kinase PdxY [*Paracidovorax citrulli*]
fig|80869.180.peg.1062	T3S	DNA internalization-related competence protein ComEC/Rec2 [*Paracidovorax citrulli*]
fig|80869.180.peg.1070	T3S	VF530 family DNA-binding protein [*Paracidovorax citrulli*]
fig|80869.180.peg.1103	T3S	CaiB/BaiF CoA-transferase family protein [*Paracidovorax citrulli*]
fig|80869.180.peg.1114	T3S	ketopantoate reductase [*Paracidovorax citrulli*]
fig|80869.180.peg.1129	T3S	protein-L-isoaspartate(D-aspartate) O-methyltransferase [*Paracidovorax citrulli*]
fig|80869.180.peg.114	T3S	amino acid ABC transporter ATP-binding protein [*Paracidovorax citrulli*]
fig|80869.180.peg.1144	T3S	GTPase HflX [*Paracidovorax citrulli*]
fig|80869.180.peg.1169	T3S	DUF2069 domain-containing protein [*Paracidovorax citrulli*]

Prot	Pred	Description
fig|80869.180.peg.1172	T3S	farnesyl-diphosphate farnesyltransferase [*Paracidovorax citrulli* AAC00-1]
fig|80869.180.peg.1213	T3S	glycine betaine/L-proline ABC transporter permease ProW [*Paracidovorax citrulli*]
fig|80869.180.peg.122	T3S	MULTISPECIES: ABC transporter ATP-binding protein [Paracidovorax]
fig|80869.180.peg.1231	T3S	malonyl-CoA synthase [*Paracidovorax citrulli*]
fig|80869.180.peg.1236	T3S	cation-translocating P-type ATPase [*Paracidovorax citrulli*]
fig|80869.180.peg.1266	T3S	SDR family NAD(P)-dependent oxidoreductase [*Paracidovorax citrulli*]
fig|80869.180.peg.1312	T3S	type 1 glutamine amidotransferase domain-containing protein [*Paracidovorax citrulli*]
fig|80869.180.peg.1314	T3S	polyhydroxyalkanoate synthesis repressor PhaR [*Paracidovorax citrulli*]
fig|80869.180.peg.1316	T3S	MHS family citrate/tricarballylate:H+ symporter-like MFS transporter [*Paracidovorax citrulli*]
fig|80869.180.peg.137	T3S	DHA2 family efflux MFS transporter permease subunit [*Paracidovorax citrulli*]
fig|80869.180.peg.138	T3S	efflux RND transporter periplasmic adaptor subunit [*Paracidovorax citrulli*]
fig|80869.180.peg.1380	T3S	hypothetical protein [*Paracidovorax citrulli*]
fig|80869.180.peg.1407	T3S	PLxRFG domain-containing protein [*Paracidovorax citrulli*]
fig|80869.180.peg.1456	T3S	ATP synthase F1 subunit epsilon [*Paracidovorax citrulli*]
fig|80869.180.peg.1461	T3S	P1 family peptidase [*Paracidovorax citrulli*]
fig|80869.180.peg.1471	T3S	YitT family protein [*Paracidovorax citrulli*]
fig|80869.180.peg.1475	T3S	MlaD family protein [*Paracidovorax citrulli*]
fig|80869.180.peg.1490	T3S	UDP-3-O-(3-hydroxymyristoyl)glucosamine N-acyltransferase [*Paracidovorax citrulli*]
fig|80869.180.peg.1523	T3S	glutamate--tRNA ligase [*Paracidovorax citrulli*]
fig|80869.180.peg.1524	T3S	DUF1624 domain-containing protein [*Paracidovorax citrulli*]
fig|80869.180.peg.1527	T3S	DUF72 domain-containing protein [*Paracidovorax citrulli*]
fig|80869.180.peg.1545	T3S	lipid A export permease/ATP-binding protein MsbA [*Paracidovorax citrulli*]
fig|80869.180.peg.1549	T3S	ThiF family adenylyltransferase [*Paracidovorax citrulli*]
fig|80869.180.peg.1565	T3S	cytochrome c553-like protein [*Paracidovorax citrulli* AAC00-1]
fig|80869.180.peg.1567	T3S	thioredoxin family protein [*Paracidovorax citrulli*]
fig|80869.180.peg.1593	T3S	AlpA family transcriptional regulator [*Paracidovorax citrulli*]
fig|80869.180.peg.1636	T3S	Bifunctional protein PutA [*Paracidovorax citrulli*]
fig|80869.180.peg.1638	T3S	hypothetical protein [*Paracidovorax citrulli*]
fig|80869.180.peg.1711	T3S	ABC transporter permease [*Paracidovorax citrulli*]
fig|80869.180.peg.176	T3S	potassium-transporting ATPase subunit KdpB [*Paracidovorax citrulli*]
fig|80869.180.peg.1804	T3S	MULTISPECIES: CysB family HTH-type transcriptional regulator [Comamonadaceae]
fig|80869.180.peg.1817	T3S	2OG-Fe dioxygenase family protein [*Paracidovorax citrulli*]
fig|80869.180.peg.1841	T3S	TetR/AcrR family transcriptional regulator [*Paracidovorax citrulli*]
fig|80869.180.peg.1860	T3S	RDD domain containing protein [*Paracidovorax citrulli* AAC00-1]
fig|80869.180.peg.1874	T3S	YbaN family protein [*Paracidovorax citrulli*]
fig|80869.180.peg.1884	T3S	ATP-binding protein [*Paracidovorax citrulli*]
fig|80869.180.peg.1891	T3S	hypothetical protein [*Paracidovorax citrulli*]
fig|80869.180.peg.1950	T3S	tRNA (guanosine(46)-N7)-methyltransferase TrmB [*Paracidovorax citrulli*]
fig|80869.180.peg.1952	T3S	NAD(P)/FAD-dependent oxidoreductase [*Paracidovorax citrulli*]
fig|80869.180.peg.1960	T3S	SulP family inorganic anion transporter [*Paracidovorax citrulli*]
fig|80869.180.peg.1961	T3S	BON domain-containing protein [*Paracidovorax citrulli*]
fig|80869.180.peg.199	T3S	NEL-type E3 ubiquitin ligase domain-containing protein [*Paracidovorax citrulli*]
fig|80869.180.peg.2077	T3S	conserved hypothetical protein [*Paracidovorax citrulli* AAC00-1]
fig|80869.180.peg.208	T3S	N-acetylneuraminate epimerase [*Paracidovorax citrulli*]

Prot	Pred	Description
fig|80869.180.peg.2082	T3S	hypothetical protein [*Paracidovorax citrulli*]
fig|80869.180.peg.2085	T3S	structural protein P5 [*Paracidovorax citrulli*]
fig|80869.180.peg.2098	T3S	Uncharacterized conserved protein YdiU, UPF0061 family [*Paracidovorax citrulli*]
fig|80869.180.peg.2111	T3S	glycoside hydrolase family 5 protein [*Paracidovorax citrulli*]
fig|80869.180.peg.2163	T3S	KGG domain-containing protein [*Paracidovorax citrulli*]
fig|80869.180.peg.2208	T3S	PAS domain S-box protein [*Paracidovorax citrulli*]
fig|80869.180.peg.2239	T3S	EAL domain-containing protein [*Paracidovorax citrulli*]
fig|80869.180.peg.2315	T3S	FAD-binding oxidoreductase [*Paracidovorax citrulli*]
fig|80869.180.peg.2318	T3S	ATP-dependent RNA helicase HrpA [*Paracidovorax citrulli*]
fig|80869.180.peg.2360	T3S	TonB-dependent receptor [*Paracidovorax citrulli*]
fig|80869.180.peg.2371	T3S	D-serine/D-alanine/glycine transporter [*Paracidovorax citrulli*]
fig|80869.180.peg.2372	T3S	hypothetical protein [*Paracidovorax citrulli*]
fig|80869.180.peg.2386	T3S	Polyphosphate kinase [*Paracidovorax citrulli* AAC00-1]
fig|80869.180.peg.2391	T3S	phosphate ABC transporter permease PstC [*Paracidovorax citrulli*]
fig|80869.180.peg.242	T3S	TspO/MBR family protein [*Paracidovorax citrulli*]
fig|80869.180.peg.2421	T3S	gephyrin-like molybdotransferase Glp [*Paracidovorax citrulli*]
fig|80869.180.peg.2424	T3S	GTP cyclohydrolase subunit MoaA [*Paracidovorax citrulli* AAC00-1]
fig|80869.180.peg.2435	T3S	translesion DNA synthesis-associated protein ImuA [*Paracidovorax citrulli*]
fig|80869.180.peg.2439	T3S	pseudouridine synthase [*Paracidovorax citrulli*]
fig|80869.180.peg.2469	T3S	pyridoxamine 5'-phosphate oxidase [*Paracidovorax citrulli*]
fig|80869.180.peg.247	T3S	hypothetical protein [*Paracidovorax citrulli*]
fig|80869.180.peg.2480	T3S	transcriptional repressor [*Paracidovorax citrulli*]
fig|80869.180.peg.2482	T3S	TetR/AcrR family transcriptional regulator [*Paracidovorax citrulli*]
fig|80869.180.peg.254	T3S	DUF4139 domain-containing protein [*Paracidovorax citrulli*]
fig|80869.180.peg.2542	T3S	class III extradiol ring-cleavage dioxygenase [*Paracidovorax citrulli*]
fig|80869.180.peg.2547	T3S	glutamine--tRNA ligase/YqeY domain fusion protein [*Paracidovorax citrulli*]
fig|80869.180.peg.2559	T3S	alpha/beta hydrolase [*Paracidovorax citrulli*]
fig|80869.180.peg.2562	T3S	Murein DD-endopeptidase MepM [*Paracidovorax citrulli*]
fig|80869.180.peg.2572	T3S	DHH family phosphoesterase [*Paracidovorax citrulli*]
fig|80869.180.peg.2576	T3S	redox-sensitive transcriptional activator SoxR [*Paracidovorax citrulli*]
fig|80869.180.peg.2578	T3S	MFS transporter [*Paracidovorax citrulli*]
fig|80869.180.peg.2585	T3S	GAF domain-containing sensor histidine kinase [*Paracidovorax citrulli*]
fig|80869.180.peg.2592	T3S	SMP-30/gluconolactonase/LRE family protein [*Paracidovorax citrulli*]
fig|80869.180.peg.2593	T3S	SDR family NAD(P)-dependent oxidoreductase [*Paracidovorax citrulli*]
fig|80869.180.peg.2599	T3S	multiple monosaccharide ABC transporter permease [*Paracidovorax citrulli*]
fig|80869.180.peg.2633	T3S	cytochrome o ubiquinol oxidase subunit IV [*Paracidovorax citrulli*]
fig|80869.180.peg.2648	T3S	transcriptional regulator, GntR family [*Paracidovorax citrulli*]
fig|80869.180.peg.2662	T3S	NAD-dependent succinate-semialdehyde dehydrogenase [*Paracidovorax citrulli*]
fig|80869.180.peg.2679	T3S	hypothetical protein [*Paracidovorax citrulli*]
fig|80869.180.peg.2710	T3S	hypothetical protein FRC90_13055 [*Paracidovorax citrulli*]
fig|80869.180.peg.2732	T3S	MFS transporter [*Paracidovorax citrulli*]
fig|80869.180.peg.2743	T3S	carboxyl transferase domain-containing protein [*Paracidovorax citrulli*]
fig|80869.180.peg.2566	T4S	major facilitator superfamily MFS_1 [*Paracidovorax citrulli* AAC00-1]
fig|80869.180.peg.2677	T4S	hypothetical protein [*Paracidovorax citrulli*]
fig|80869.180.peg.681	T4S	Ig domain protein, group 1 domain protein [*Paracidovorax citrulli* AAC00-1]

Prot	Pred	Description
fig|80869.180.peg.2762	T4S	acyl-CoA thioesterase [*Paracidovorax citrulli*]
fig|80869.180.peg.2171	T4S	STY0301 family protein [*Paracidovorax citrulli*]
fig|80869.180.peg.1276	T4S	XopE/AvrPphe family type III secretion system effector [*Paracidovorax citrulli*]
fig|80869.180.peg.2019	T4S	ABC transporter ATP-binding protein [*Paracidovorax citrulli*]
fig|80869.180.peg.2280	T4S	Ku protein [*Paracidovorax citrulli*]
fig|80869.180.peg.2284	T4S	hypothetical protein [*Paracidovorax citrulli*]
fig|80869.180.peg.3461	T4S	bacterioferritin [*Paracidovorax citrulli*]
fig|80869.180.peg.1383	T4S	terminase small subunit [*Paracidovorax citrulli*]
fig|80869.180.peg.955	T4S	molybdenum cofactor biosynthesis protein MoaE [*Paracidovorax citrulli*]
fig|80869.180.peg.1810	T4S	hypothetical protein [*Paracidovorax citrulli*]
fig|80869.180.peg.1326	T4S	isoaspartyl peptidase/L-asparaginase family protein [*Paracidovorax citrulli*]
fig|80869.180.peg.2053	T4S	DUF2800 domain-containing protein [*Paracidovorax citrulli*]
fig|80869.180.peg.2718	T4S	RNA-binding protein [*Paracidovorax citrulli*]
fig|80869.180.peg.1906	T4S	hypothetical protein Aave_3072 [*Paracidovorax citrulli* AAC00-1]
fig|80869.180.peg.2508	T4S	DUF1795 domain-containing protein [*Paracidovorax citrulli*]
fig|80869.180.peg.2314	T4S	amino-acid N-acetyltransferase [Paracidovorax]
fig|80869.180.peg.4238	T4S	DUF924 family protein [*Paracidovorax citrulli*]
fig|80869.180.peg.4177	T4S	50S ribosomal protein L11 [*Paracidovorax citrulli*]
fig|80869.180.peg.2591	T4S	conserved hypothetical protein [*Paracidovorax citrulli* AAC00-1]
fig|80869.180.peg.3160	T4S	PP2C family serine/threonine-protein phosphatase [*Paracidovorax citrulli*]
fig|80869.180.peg.4552	T4S	hypothetical protein [*Paracidovorax citrulli*]
fig|80869.180.peg.4156	T4S	cryptochrome/photolyase family protein [*Paracidovorax citrulli*]
fig|80869.180.peg.1032	T4S	phosphopyruvate hydratase [*Paracidovorax citrulli*]
fig|80869.180.peg.3513	T4S	glutathione-regulated potassium-efflux system protein KefC [*Paracidovorax citrulli*]
fig|80869.180.peg.3104	T4S	hypothetical protein [*Paracidovorax citrulli*]
fig|80869.180.peg.1701	T4S	hypothetical protein [*Paracidovorax citrulli*]
fig|80869.180.peg.1503	T4S	AAA family ATPase [*Paracidovorax citrulli*]
fig|80869.180.peg.3943	T4S	DUF3025 domain-containing protein [*Paracidovorax citrulli*]
fig|80869.180.peg.3161	T4S	serine/threonine-protein kinase [*Paracidovorax citrulli*]
fig|80869.180.peg.3386	T4S	hypothetical protein [*Paracidovorax citrulli*]
fig|80869.180.peg.2794	T4S	inositol monophosphatase family protein [*Paracidovorax citrulli*]
fig|80869.180.peg.3869	T4S	N-acetylmuramoyl-L-alanine amidase [*Paracidovorax citrulli*]
fig|80869.180.peg.323	T4S	AraC family transcriptional regulator [*Paracidovorax citrulli*]
fig|80869.180.peg.1132	T4S	SMC-Scp complex subunit ScpB [*Paracidovorax citrulli*]
fig|80869.180.peg.1640	T4S	hypothetical protein [*Paracidovorax citrulli*]
fig|80869.180.peg.4273	T4S	Fic family protein [*Paracidovorax citrulli*]
fig|80869.180.peg.3368	T4S	4-hydroxy-3-methylbut-2-enyl diphosphate reductase [*Paracidovorax citrulli*]
fig|80869.180.peg.1580	T4S	SsrA-binding protein SmpB [*Paracidovorax citrulli*]
fig|80869.180.peg.3737	T4S	hypothetical protein [*Paracidovorax citrulli*]
fig|80869.180.peg.191	T4S	F0F1 ATP synthase subunit gamma [*Paracidovorax citrulli*]
fig|80869.180.peg.2942	T4S	hypothetical protein [*Paracidovorax citrulli*]
fig|80869.180.peg.2052	T4S	DUF2815 family protein [*Paracidovorax citrulli*]
fig|80869.180.peg.1159	T4S	hypothetical protein [*Paracidovorax citrulli*]
fig|80869.180.peg.2415	T4S	transglycosylase SLT domain-containing protein [*Paracidovorax citrulli*]
fig|80869.180.peg.303	T4S	type III secretion system outer membrane ring subunit SctC [*Paracidovorax citrulli*]

Prot	Pred	Description
fig|80869.180.peg.1401	T4S	hypothetical protein [*Paracidovorax citrulli*]
fig|80869.180.peg.3576	T4S	5'-nucleotidase [*Paracidovorax citrulli*]
fig|80869.180.peg.359	T4S	30S ribosomal protein S4 [*Paracidovorax citrulli*]
fig|80869.180.peg.2323	T4S	adenylate kinase [*Paracidovorax citrulli*]
fig|80869.180.peg.1720	T4S	hypothetical protein [*Paracidovorax citrulli*]
fig|80869.180.peg.3130	T4S	type IV pilin protein [*Paracidovorax citrulli*]
fig|80869.180.peg.3962	T4S	hypothetical protein [*Paracidovorax citrulli*]
fig|80869.180.peg.3462	T4S	BON domain-containing protein [*Paracidovorax citrulli*]
fig|80869.180.peg.2300	T4S	MetQ/NlpA family ABC transporter substrate-binding protein [*Paracidovorax citrulli*]
fig|80869.180.peg.4106	T4S	Bug family tripartite tricarboxylate transporter substrate binding protein [*Paracidovorax citrulli*]
fig|80869.180.peg.1497	T4S	tripartite tricarboxylate transporter substrate binding protein [*Paracidovorax citrulli*]
fig|80869.180.peg.2306	T4S	sulfate ABC transporter substrate-binding protein [*Paracidovorax citrulli*]
fig|80869.180.peg.3344	T4S	hypothetical protein [*Paracidovorax citrulli*]
fig|80869.180.peg.4237	T4S	OmpA family protein [*Paracidovorax citrulli*]
fig|80869.180.peg.1415	T4S	glycoside hydrolase family protein [*Paracidovorax citrulli*]
fig|80869.180.peg.118	T4S	HTH-type transcriptional regulator GltC [*Paracidovorax citrulli*]
fig|80869.180.peg.2974	T4S	sigma-54 dependent transcriptional regulator [*Paracidovorax citrulli*]
fig|80869.180.peg.3577	T4S	EF-hand domain-containing protein [*Paracidovorax citrulli*]

*P_citrulli*_KACC17005

Prot	Pred	Description
fig|80869.178.peg.3404	T3S	Ig domain protein, group 1 domain protein [*Paracidovorax citrulli* AAC00-1]
fig|80869.178.peg.3886	T3S	DUF3577 domain-containing protein [*Paracidovorax citrulli*]
fig|80869.178.peg.690	T3S	major facilitator superfamily MFS_1 [*Paracidovorax citrulli* AAC00-1]
fig|80869.178.peg.805	T3S	hypothetical protein [*Paracidovorax citrulli*]
fig|80869.178.peg.888	T3S	acyl-CoA thioesterase [*Paracidovorax citrulli*]
fig|80869.178.peg.530	T3S	STY0301 family protein [*Paracidovorax citrulli*]
fig|80869.178.peg.1952	T3S	ABC transporter ATP-binding protein [*Paracidovorax citrulli*]
fig|80869.178.peg.416	T3S	Ku protein [*Paracidovorax citrulli*]
fig|80869.178.peg.1000	T3S	AI-2E family transporter [*Paracidovorax citrulli*]
fig|80869.178.peg.1002	T3S	phosphoenolpyruvate carboxylase [*Paracidovorax citrulli*]
fig|80869.178.peg.1005	T3S	uroporphyrinogen-III C-methyltransferase [*Paracidovorax citrulli*]
fig|80869.178.peg.1007	T3S	ABC-F family ATP-binding cassette domain-containing protein [*Paracidovorax citrulli*]
fig|80869.178.peg.1020	T3S	hypothetical protein [*Paracidovorax citrulli*]
fig|80869.178.peg.1029	T3S	CDP-6-deoxy-delta-3,4-glucoseen reductase [*Paracidovorax citrulli*]
fig|80869.178.peg.104	T3S	pyridoxamine 5'-phosphate oxidase [*Paracidovorax citrulli*]
fig|80869.178.peg.1059	T3S	argininosuccinate lyase [*Paracidovorax citrulli*]
fig|80869.178.peg.1153	T3S	hypothetical protein [*Paracidovorax citrulli*]
fig|80869.178.peg.1157	T3S	protein of unknown function DUF1653 [*Paracidovorax citrulli* AAC00-1]
fig|80869.178.peg.1172	T3S	isoleucine--tRNA ligase [*Paracidovorax citrulli*]
fig|80869.178.peg.1222	T3S	PilZ domain-containing protein [*Paracidovorax citrulli*]
fig|80869.178.peg.1261	T3S	urease accessory protein [*Paracidovorax citrulli*]
fig|80869.178.peg.1262	T3S	urease accessory protein UreG [*Paracidovorax citrulli*]

Prot	Pred	Description
fig|80869.178.peg.1300	T3S	cyclopropane-fatty-acyl-phospholipid synthase family protein [*Paracidovorax citrulli*]
fig|80869.178.peg.1370	T3S	cardiolipin synthase [*Paracidovorax citrulli*]
fig|80869.178.peg.1385	T3S	GGDEF domain-containing protein [*Paracidovorax citrulli*]
fig|80869.178.peg.1394	T3S	glycerophosphodiester phosphodiesterase [*Paracidovorax citrulli*]
fig|80869.178.peg.1470	T3S	hypothetical protein [*Paracidovorax citrulli*]
fig|80869.178.peg.1471	T3S	4'-phosphopantetheinyl transferase [*Paracidovorax citrulli* AAC00-1]
fig|80869.178.peg.1476	T3S	GNAT family N-acetyltransferase [*Paracidovorax citrulli*]
fig|80869.178.peg.1584	T3S	hypothetical protein [*Paracidovorax citrulli*]
fig|80869.178.peg.1596	T3S	YitT family protein [*Paracidovorax citrulli*]
fig|80869.178.peg.1597	T3S	hypothetical protein [*Paracidovorax citrulli*]
fig|80869.178.peg.1609	T3S	hypothetical protein [*Paracidovorax citrulli*]
fig|80869.178.peg.1613	T3S	3-methyl-2-oxobutanoate hydroxymethyltransferase [*Paracidovorax citrulli*]
fig|80869.178.peg.1634	T3S	signal recognition particle-docking protein FtsY [*Paracidovorax citrulli*]
fig|80869.178.peg.1645	T3S	MATE family efflux transporter [*Paracidovorax citrulli*]
fig|80869.178.peg.1714	T3S	type III secretion system chaperone [*Paracidovorax citrulli*]
fig|80869.178.peg.1715	T3S	hypothetical protein [*Paracidovorax citrulli*]
fig|80869.178.peg.1742	T3S	luciferase family protein [*Paracidovorax citrulli* AAC00-1]
fig|80869.178.peg.1759	T3S	hypothetical protein [*Paracidovorax citrulli*]
fig|80869.178.peg.176	T3S	hypothetical protein [*Paracidovorax citrulli*]
fig|80869.178.peg.1773	T3S	ABC transporter permease [*Paracidovorax citrulli*]
fig|80869.178.peg.1774	T3S	ABC transporter permease [*Paracidovorax citrulli*]
fig|80869.178.peg.1786	T3S	(2Fe-2S)-binding protein [*Paracidovorax citrulli*]
fig|80869.178.peg.1787	T3S	molybdenum cofactor cytidylyltransferase [*Paracidovorax citrulli*]
fig|80869.178.peg.1793	T3S	hypothetical protein [*Paracidovorax citrulli*]
fig|80869.178.peg.1794	T3S	NUDIX domain-containing protein [*Paracidovorax citrulli*]
fig|80869.178.peg.1804	T3S	alpha/beta hydrolase [*Paracidovorax citrulli*]
fig|80869.178.peg.1830	T3S	septal ring lytic transglycosylase RlpA family protein [*Paracidovorax citrulli*]
fig|80869.178.peg.1843	T3S	hypothetical protein [*Paracidovorax citrulli*]
fig|80869.178.peg.1850	T3S	TRAP transporter small permease [*Paracidovorax citrulli*]
fig|80869.178.peg.1918	T3S	glycoside hydrolase family 5 protein [*Paracidovorax citrulli*]
fig|80869.178.peg.1930	T3S	protein of unknown function UPF0061 [*Paracidovorax citrulli* AAC00-1]
fig|80869.178.peg.2011	T3S	BON domain-containing protein [*Paracidovorax citrulli*]
fig|80869.178.peg.2012	T3S	SulP family inorganic anion transporter [*Paracidovorax citrulli*]
fig|80869.178.peg.2019	T3S	NAD(P)/FAD-dependent oxidoreductase [*Paracidovorax citrulli*]
fig|80869.178.peg.2021	T3S	tRNA (guanosine(46)-N7)-methyltransferase TrmB [*Paracidovorax citrulli*]
fig|80869.178.peg.2082	T3S	hypothetical protein [*Paracidovorax citrulli*]
fig|80869.178.peg.2089	T3S	ATP-binding protein [*Paracidovorax citrulli*]
fig|80869.178.peg.2099	T3S	YbaN family protein [*Paracidovorax citrulli*]
fig|80869.178.peg.2114	T3S	RDD domain containing protein [*Paracidovorax citrulli* AAC00-1]
fig|80869.178.peg.2159	T3S	2OG-Fe dioxygenase family protein [*Paracidovorax citrulli*]
fig|80869.178.peg.2172	T3S	CysB family HTH-type transcriptional regulator [Comamonadaceae]
fig|80869.178.peg.218	T3S	DEAD/DEAH box helicase [*Paracidovorax citrulli*]
fig|80869.178.peg.2228	T3S	DUF4230 domain-containing protein [*Paracidovorax citrulli*]
fig|80869.178.peg.2264	T3S	ABC transporter permease [*Paracidovorax citrulli*]
fig|80869.178.peg.230	T3S	hypothetical protein CQB05_01060 [*Paracidovorax citrulli*]
fig|80869.178.peg.2334	T3S	hypothetical protein [*Paracidovorax citrulli*]

Prot	Pred	Description
fig|80869.178.peg.2336	T3S	L-proline dehydrogenase /delta-1-pyrroline-5-carboxylate dehydrogenase [*Paracidovorax citrulli*]
fig|80869.178.peg.2379	T3S	AlpA family transcriptional regulator [*Paracidovorax citrulli*]
fig|80869.178.peg.2405	T3S	thioredoxin family protein [*Paracidovorax citrulli*]
fig|80869.178.peg.2407	T3S	cytochrome c553-like protein [*Paracidovorax citrulli* AAC00-1]
fig|80869.178.peg.2423	T3S	ThiF family adenylyltransferase [*Paracidovorax citrulli*]
fig|80869.178.peg.2446	T3S	DUF72 domain-containing protein [*Paracidovorax citrulli*]
fig|80869.178.peg.2449	T3S	DUF1624 domain-containing protein [*Paracidovorax citrulli*]
fig|80869.178.peg.2450	T3S	glutamate--tRNA ligase [*Paracidovorax citrulli*]
fig|80869.178.peg.2483	T3S	UDP-3-O-(3-hydroxymyristoyl)glucosamine N-acyltransferase [*Paracidovorax citrulli*]
fig|80869.178.peg.2498	T3S	MlaD family protein [*Paracidovorax citrulli*]
fig|80869.178.peg.2502	T3S	YitT family protein [*Paracidovorax citrulli*]
fig|80869.178.peg.2513	T3S	P1 family peptidase [*Paracidovorax citrulli*]
fig|80869.178.peg.2518	T3S	ATP synthase F1 subunit epsilon [*Paracidovorax citrulli*]
fig|80869.178.peg.253	T3S	pseudouridine synthase [*Paracidovorax citrulli*]
fig|80869.178.peg.2533	T3S	structural protein P5 [*Paracidovorax citrulli*]
fig|80869.178.peg.2535	T3S	hypothetical protein [*Paracidovorax citrulli*]
fig|80869.178.peg.257	T3S	translesion DNA synthesis-associated protein ImuA [*Paracidovorax citrulli*]
fig|80869.178.peg.2647	T3S	hypothetical protein [*Paracidovorax citrulli*]
fig|80869.178.peg.2660	T3S	hypothetical protein Aave_1685 [*Paracidovorax citrulli* AAC00-1]
fig|80869.178.peg.268	T3S	GTP cyclohydrolase subunit MoaA [*Paracidovorax citrulli* AAC00-1]
fig|80869.178.peg.271	T3S	gephyrin-like molybdotransferase Glp [*Paracidovorax citrulli*]
fig|80869.178.peg.2718	T3S	hypothetical protein [*Paracidovorax citrulli*]
fig|80869.178.peg.2748	T3S	hypothetical protein Aave_1606 [*Paracidovorax citrulli* AAC00-1]
fig|80869.178.peg.2758	T3S	polyhydroxyalkanoate synthesis repressor PhaR [*Paracidovorax citrulli*]
fig|80869.178.peg.2760	T3S	type 1 glutamine amidotransferase domain-containing protein [*Paracidovorax citrulli*]
fig|80869.178.peg.2808	T3S	SDR family NAD(P)-dependent oxidoreductase [*Paracidovorax citrulli*]
fig|80869.178.peg.2838	T3S	cation-translocating P-type ATPase [*Paracidovorax citrulli*]
fig|80869.178.peg.2846	T3S	malonyl-CoA synthase [*Paracidovorax citrulli*]
fig|80869.178.peg.2865	T3S	glycine betaine/L-proline ABC transporter permease ProW [*Paracidovorax citrulli*]
fig|80869.178.peg.2908	T3S	farnesyl-diphosphate farnesyltransferase [*Paracidovorax citrulli* AAC00-1]
fig|80869.178.peg.2911	T3S	DUF2069 domain-containing protein [*Paracidovorax citrulli*]
fig|80869.178.peg.2937	T3S	GTPase HflX [*Paracidovorax citrulli*]
fig|80869.178.peg.2953	T3S	protein-L-isoaspartate(D-aspartate) O-methyltransferase [*Paracidovorax citrulli*]
fig|80869.178.peg.2980	T3S	CaiB/BaiF CoA-transferase family protein [*Paracidovorax citrulli*]
fig|80869.178.peg.3012	T3S	VF530 family DNA-binding protein [*Paracidovorax citrulli*]
fig|80869.178.peg.3020	T3S	DNA internalization-related competence protein ComEC/Rec2 [*Paracidovorax citrulli*]
fig|80869.178.peg.3026	T3S	pyridoxal kinase PdxY [*Paracidovorax citrulli*]
fig|80869.178.peg.303	T3S	phosphate ABC transporter permease PstC [*Paracidovorax citrulli*]
fig|80869.178.peg.3052	T3S	hypothetical protein [*Paracidovorax citrulli*]
fig|80869.178.peg.3056	T3S	diguanylate phosphodiesterase [*Paracidovorax citrulli* AAC00-1]
fig|80869.178.peg.3078	T3S	helix-turn-helix domain-containing protein [*Paracidovorax citrulli*]
fig|80869.178.peg.309	T3S	Polyphosphate kinase [*Paracidovorax citrulli* AAC00-1]
fig|80869.178.peg.3096	T3S	ABC transporter transmembrane domain-containing protein [*Paracidovorax citrulli*]

Prot	Pred	Description
fig|80869.178.peg.3105	T3S	MULTISPECIES: NADH-quinone oxidoreductase subunit NuoI [Comamonadaceae]
fig|80869.178.peg.3126	T3S	hypothetical protein [*Paracidovorax citrulli*]
fig|80869.178.peg.3138	T3S	molybdopterin converting factor subunit 1 [*Paracidovorax citrulli*]
fig|80869.178.peg.3166	T3S	phosphoribosylanthranilate isomerase [*Paracidovorax citrulli* AAC00-1]
fig|80869.178.peg.3171	T3S	LON peptidase substrate-binding domain-containing protein [*Paracidovorax citrulli*]
fig|80869.178.peg.3179	T3S	Phytochrome-like protein cph2 [*Paracidovorax citrulli*]
fig|80869.178.peg.322	T3S	hypothetical protein [*Paracidovorax citrulli*]
fig|80869.178.peg.323	T3S	D-serine/D-alanine/glycine transporter [*Paracidovorax citrulli*]
fig|80869.178.peg.3233	T3S	tryptophan 2,3-dioxygenase [*Paracidovorax citrulli*]
fig|80869.178.peg.3243	T3S	neutral zinc metallopeptidase [*Paracidovorax citrulli*]
fig|80869.178.peg.3282	T3S	glycerate kinase [*Paracidovorax citrulli*]
fig|80869.178.peg.3298	T3S	NCS1 family nucleobase:cation symporter-1 [*Paracidovorax citrulli*]
fig|80869.178.peg.3300	T3S	GntR family transcriptional regulator [*Paracidovorax citrulli*]
fig|80869.178.peg.3319	T3S	DMT family transporter [*Paracidovorax citrulli*]
fig|80869.178.peg.334	T3S	TonB-dependent receptor [*Paracidovorax citrulli*]
fig|80869.178.peg.3400	T3S	protein of unknown function DUF1415 [*Paracidovorax citrulli* AAC00-1]
fig|80869.178.peg.3410	T3S	penicillin-binding protein 1A [*Paracidovorax citrulli*]
fig|80869.178.peg.3435	T3S	peptidoglycan-binding domain-containing protein [*Paracidovorax citrulli*]
fig|80869.178.peg.3438	T3S	NCS1 family nucleobase:cation symporter-1 [*Paracidovorax citrulli*]
fig|80869.178.peg.3471	T3S	ABC transporter ATP-binding protein [*Paracidovorax citrulli*]
fig|80869.178.peg.3503	T3S	Holliday junction resolvase RuvX [*Paracidovorax citrulli*]
fig|80869.178.peg.3512	T3S	bifunctional hydroxymethylpyrimidine kinase/phosphomethylpyrimidine kinase [*Paracidovorax citrulli*]
fig|80869.178.peg.3546	T3S	ribonucleoside-diphosphate reductase subunit alpha [*Paracidovorax citrulli*]
fig|80869.178.peg.3559	T3S	3-deoxy-7-phosphoheptulonate synthase [*Paracidovorax citrulli*]
fig|80869.178.peg.3607	T3S	GTP-binding protein [*Paracidovorax citrulli*]
fig|80869.178.peg.3618	T3S	chromate efflux transporter [*Paracidovorax citrulli*]
fig|80869.178.peg.3625	T3S	M48 family metallopeptidase [*Paracidovorax citrulli*]
fig|80869.178.peg.3640	T3S	DNA-3-methyladenine glycosylase I [*Paracidovorax citrulli*]
fig|80869.178.peg.3720	T3S	FMN-dependent NADH-azoreductase [*Paracidovorax citrulli*]
fig|80869.178.peg.3766	T3S	NarK family nitrate/nitrite MFS transporter [*Paracidovorax citrulli*]
fig|80869.178.peg.3768	T3S	transcriptional regulator, AraC family [*Paracidovorax citrulli* AAC00-1]
fig|80869.178.peg.3781	T3S	FAD-linked oxidase C-terminal domain-containing protein [*Paracidovorax citrulli*]
fig|80869.178.peg.3828	T3S	glycosyltransferase involved in cell wall biosynthesis [*Paracidovorax citrulli*]
fig|80869.178.peg.3841	T3S	uracil-DNA glycosylase [*Paracidovorax citrulli*]
fig|80869.178.peg.3876	T3S	HNH endonuclease [*Paracidovorax citrulli*]
fig|80869.178.peg.3896	T3S	MULTISPECIES: hypothetical protein [Pseudomonadota]
fig|80869.178.peg.3963	T3S	hypothetical protein [*Paracidovorax citrulli*]
fig|80869.178.peg.3969	T3S	hypothetical protein [*Paracidovorax citrulli*]
fig|80869.178.peg.3980	T3S	hypothetical protein [*Paracidovorax citrulli*]
fig|80869.178.peg.3981	T3S	hypothetical protein [*Paracidovorax citrulli*]
fig|80869.178.peg.3985	T3S	hypothetical protein Aave_0457 [*Paracidovorax citrulli* AAC00-1]
fig|80869.178.peg.4021	T3S	hypothetical protein [*Paracidovorax citrulli*]
fig|80869.178.peg.4026	T3S	TspO/MBR family protein [*Paracidovorax citrulli*]
fig|80869.178.peg.4061	T3S	kelch repeat-containing protein [*Paracidovorax citrulli*]
fig|80869.178.peg.4069	T3S	TPM domain-containing protein [*Paracidovorax citrulli*]

Prot	Pred	Description
fig|80869.178.peg.4092	T3S	potassium-transporting ATPase subunit KdpB [*Paracidovorax citrulli*]
fig|80869.178.peg.4128	T3S	efflux RND transporter periplasmic adaptor subunit [*Paracidovorax citrulli*]
fig|80869.178.peg.4129	T3S	DHA2 family efflux MFS transporter permease subunit [*Paracidovorax citrulli*]
fig|80869.178.peg.3404	T4S	Ig domain protein, group 1 domain protein [*Paracidovorax citrulli* AAC00-1]
fig|80869.178.peg.3886	T4S	DUF3577 domain-containing protein [*Paracidovorax citrulli*]
fig|80869.178.peg.690	T4S	major facilitator superfamily MFS_1 [*Paracidovorax citrulli* AAC00-1]
fig|80869.178.peg.805	T4S	hypothetical protein [*Paracidovorax citrulli*]
fig|80869.178.peg.888	T4S	acyl-CoA thioesterase [*Paracidovorax citrulli*]
fig|80869.178.peg.530	T4S	STY0301 family protein [*Paracidovorax citrulli*]
fig|80869.178.peg.1952	T4S	ABC transporter ATP-binding protein [*Paracidovorax citrulli*]
fig|80869.178.peg.416	T4S	Ku protein [*Paracidovorax citrulli*]
fig|80869.178.peg.2644	T4S	terminase small subunit [*Paracidovorax citrulli*]
fig|80869.178.peg.3132	T4S	molybdenum cofactor biosynthesis protein MoaE [*Paracidovorax citrulli*]
fig|80869.178.peg.1627	T4S	bacterioferritin [*Paracidovorax citrulli*]
fig|80869.178.peg.2693	T4S	isoaspartyl peptidase/L-asparaginase family protein [*Paracidovorax citrulli*]
fig|80869.178.peg.2951	T4S	RluA family pseudouridine synthase [*Paracidovorax citrulli*]
fig|80869.178.peg.1435	T4S	cytochrome bc complex cytochrome b subunit [*Paracidovorax citrulli*]
fig|80869.178.peg.1525	T4S	abortive infection system antitoxin AbiGi family protein [*Paracidovorax citrulli*]
fig|80869.178.peg.844	T4S	RNA-binding protein [*Paracidovorax citrulli*]
fig|80869.178.peg.229	T4S	hypothetical protein [*Paracidovorax citrulli*]
fig|80869.178.peg.2066	T4S	hypothetical protein Aave_3072 [*Paracidovorax citrulli* AAC00-1]
fig|80869.178.peg.65	T4S	DUF1795 domain-containing protein [*Paracidovorax citrulli*]
fig|80869.178.peg.3966	T4S	type III secretion system outer membrane ring subunit SctC [*Paracidovorax citrulli*]
fig|80869.178.peg.463	T4S	succinyl-diaminopimelate desuccinylase [*Paracidovorax citrulli*]
fig|80869.178.peg.829	T4S	hypothetical protein [*Paracidovorax citrulli*]
fig|80869.178.peg.381	T4S	MULTISPECIES: amino-acid N-acetyltransferase [Paracidovorax]
fig|80869.178.peg.4713	T4S	DUF924 family protein [*Paracidovorax citrulli*]
fig|80869.178.peg.1461	T4S	hypothetical protein [*Paracidovorax citrulli*]
fig|80869.178.peg.4776	T4S	50S ribosomal protein L11 [*Paracidovorax citrulli*]
fig|80869.178.peg.2600	T4S	Gp49 family protein [*Paracidovorax citrulli*]
fig|80869.178.peg.715	T4S	conserved hypothetical protein [*Paracidovorax citrulli* AAC00-1]
fig|80869.178.peg.4714	T4S	OmpA family protein [*Paracidovorax citrulli*]
fig|80869.178.peg.1308	T4S	PP2C family serine/threonine-protein phosphatase [*Paracidovorax citrulli*]
fig|80869.178.peg.2613	T4S	PLxRFG domain-containing protein [*Paracidovorax citrulli*]
fig|80869.178.peg.4389	T4S	hypothetical protein [*Paracidovorax citrulli*]
fig|80869.178.peg.4795	T4S	cryptochrome/photolyase family protein [*Paracidovorax citrulli*]
fig|80869.178.peg.3050	T4S	phosphopyruvate hydratase [*Paracidovorax citrulli*]
fig|80869.178.peg.1251	T4S	hypothetical protein [*Paracidovorax citrulli*]
fig|80869.178.peg.2274	T4S	hypothetical protein [*Paracidovorax citrulli*]
fig|80869.178.peg.2470	T4S	AAA family ATPase [*Paracidovorax citrulli*]
fig|80869.178.peg.1309	T4S	serine/threonine-protein kinase [*Paracidovorax citrulli*]
fig|80869.178.peg.923	T4S	inositol monophosphatase family protein [*Paracidovorax citrulli*]
fig|80869.178.peg.5086	T4S	N-acetylmuramoyl-L-alanine amidase [*Paracidovorax citrulli*]
fig|80869.178.peg.3836	T4S	AraC family transcriptional regulator [*Paracidovorax citrulli*]
fig|80869.178.peg.2949	T4S	SMC-Scp complex subunit ScpB [*Paracidovorax citrulli*]

Prot	Pred	Description
fig|80869.178.peg.2332	T4S	hypothetical protein [*Paracidovorax citrulli*]
fig|80869.178.peg.4676	T4S	Fic family protein [*Paracidovorax citrulli*]
fig|80869.178.peg.1532	T4S	4-hydroxy-3-methylbut-2-enyl diphosphate reductase [*Paracidovorax citrulli*]
fig|80869.178.peg.2391	T4S	SsrA-binding protein SmpB [*Paracidovorax citrulli*]
fig|80869.178.peg.1901	T4S	hypothetical protein [*Paracidovorax citrulli*]
fig|80869.178.peg.4077	T4S	F0F1 ATP synthase subunit gamma [*Paracidovorax citrulli*]
fig|80869.178.peg.1079	T4S	hypothetical protein [*Paracidovorax citrulli*]
fig|80869.178.peg.2922	T4S	hypothetical protein [*Paracidovorax citrulli*]
fig|80869.178.peg.277	T4S	transglycosylase SLT domain-containing protein [*Paracidovorax citrulli*]
fig|80869.178.peg.5222	T4S	hypothetical protein [*Paracidovorax citrulli*]
fig|80869.178.peg.3252	T4S	penicillin acylase family protein [*Paracidovorax citrulli*]
fig|80869.178.peg.2625	T4S	hypothetical protein [*Paracidovorax citrulli*]
fig|80869.178.peg.1744	T4S	5'-nucleotidase [*Paracidovorax citrulli*]
fig|80869.178.peg.599	T4S	hypothetical protein [*Paracidovorax citrulli*]
fig|80869.178.peg.3802	T4S	30S ribosomal protein S4 [*Paracidovorax citrulli*]
fig|80869.178.peg.372	T4S	adenylate kinase [*Paracidovorax citrulli*]
fig|80869.178.peg.2255	T4S	hypothetical protein [*Paracidovorax citrulli*]
fig|80869.178.peg.1277	T4S	type IV pilin protein [*Paracidovorax citrulli*]
fig|80869.178.peg.4992	T4S	hypothetical protein [*Paracidovorax citrulli*]
fig|80869.178.peg.1628	T4S	BON domain-containing protein [*Paracidovorax citrulli*]
fig|80869.178.peg.395	T4S	MetQ/NlpA family ABC transporter substrate-binding protein [*Paracidovorax citrulli*]
fig|80869.178.peg.4847	T4S	Bug family tripartite tricarboxylate transporter substrate binding protein [*Paracidovorax citrulli*]
fig|80869.178.peg.194	T4S	hypothetical protein [*Paracidovorax citrulli*]
fig|80869.178.peg.389	T4S	sulfate ABC transporter substrate-binding protein [*Paracidovorax citrulli*]
fig|80869.178.peg.1507	T4S	hypothetical protein [*Paracidovorax citrulli*]
fig|80869.178.peg.2604	T4S	glycoside hydrolase family protein [*Paracidovorax citrulli*]
fig|80869.178.peg.4150	T4S	DNA-binding transcriptional LysR family regulator [*Paracidovorax citrulli*]
fig|80869.178.peg.1114	T4S	sigma-54 dependent transcriptional regulator [*Paracidovorax citrulli*]
fig|80869.178.peg.1745	T4S	EF-hand domain-containing protein [*Paracidovorax citrulli*]

*P_citrulli_*KACC18782

Prot	Pred	Description
fig|80869.199.peg.1054	T3S	hypothetical protein [*Paracidovorax citrulli*]
fig|80869.199.peg.1166	T3S	major facilitator superfamily MFS_1 [*Paracidovorax citrulli* AAC00-1]
fig|80869.199.peg.3275	T3S	Ig domain protein, group 1 domain protein [*Paracidovorax citrulli* AAC00-1]
fig|80869.199.peg.3755	T3S	MULTISPECIES: DUF3577 domain-containing protein [Pseudomonadota]
fig|80869.199.peg.973	T3S	acyl-CoA thioesterase [*Paracidovorax citrulli*]
fig|80869.199.peg.1253	T3S	STY0301 family protein [*Paracidovorax citrulli*]
fig|80869.199.peg.1843	T3S	ABC transporter ATP-binding protein [*Paracidovorax citrulli*]
fig|80869.199.peg.1366	T3S	Ku protein [*Paracidovorax citrulli*]
fig|80869.199.peg.2523	T3S	terminase small subunit [*Paracidovorax citrulli*]
fig|80869.199.peg.1004	T3S	DUF2169 domain-containing protein [*Paracidovorax citrulli*]
fig|80869.199.peg.102	T3S	ABC transporter permease [*Paracidovorax citrulli*]
fig|80869.199.peg.103	T3S	ABC transporter permease [*Paracidovorax citrulli*]

Prot	Pred	Description
fig|80869.199.peg.1069	T3S	NAD-dependent succinate-semialdehyde dehydrogenase [*Paracidovorax citrulli*]
fig|80869.199.peg.1084	T3S	transcriptional regulator, GntR family [*Paracidovorax citrulli* AAC00-1]
fig|80869.199.peg.1099	T3S	cytochrome o ubiquinol oxidase subunit IV [*Paracidovorax citrulli*]
fig|80869.199.peg.1133	T3S	multiple monosaccharide ABC transporter permease [*Paracidovorax citrulli*]
fig|80869.199.peg.1139	T3S	SDR family NAD(P)-dependent oxidoreductase [*Paracidovorax citrulli*]
fig|80869.199.peg.1140	T3S	SMP-30/gluconolactonase/LRE family protein [*Paracidovorax citrulli*]
fig|80869.199.peg.1147	T3S	GAF domain-containing sensor histidine kinase [*Paracidovorax citrulli*]
fig|80869.199.peg.1154	T3S	MFS transporter [*Paracidovorax citrulli*]
fig|80869.199.peg.1156	T3S	redox-sensitive transcriptional activator SoxR [*Paracidovorax citrulli*]
fig|80869.199.peg.1160	T3S	DHH family phosphoesterase [*Paracidovorax citrulli*]
fig|80869.199.peg.117	T3S	hypothetical protein [*Paracidovorax citrulli*]
fig|80869.199.peg.1170	T3S	Murein DD-endopeptidase MepM [*Paracidovorax citrulli*]
fig|80869.199.peg.1173	T3S	esterase/lipase/thioesterase family protein [*Paracidovorax citrulli* AAC00-1]
fig|80869.199.peg.1185	T3S	glutamine--tRNA ligase/YqeY domain fusion protein [*Paracidovorax citrulli*]
fig|80869.199.peg.1190	T3S	DODA-type extradiol aromatic ring-opening family dioxygenase [*Paracidovorax citrulli*]
fig|80869.199.peg.1229	T3S	hypothetical protein [*Paracidovorax citrulli*]
fig|80869.199.peg.1246	T3S	KGG domain-containing protein [*Paracidovorax citrulli*]
fig|80869.199.peg.1293	T3S	PAS domain-containing sensor histidine kinase [*Paracidovorax citrulli*]
fig|80869.199.peg.1327	T3S	EAL domain-containing protein [*Paracidovorax citrulli*]
fig|80869.199.peg.134	T3S	luciferase family protein [*Paracidovorax citrulli* AAC00-1]
fig|80869.199.peg.1450	T3S	TonB-dependent receptor family protein [*Paracidovorax citrulli*]
fig|80869.199.peg.1461	T3S	D-serine/D-alanine/glycine transporter [*Paracidovorax citrulli*]
fig|80869.199.peg.1462	T3S	hypothetical protein [*Paracidovorax citrulli*]
fig|80869.199.peg.1475	T3S	Polyphosphate kinase [*Paracidovorax citrulli* AAC00-1]
fig|80869.199.peg.1480	T3S	phosphate ABC transporter permease PstC [*Paracidovorax citrulli*]
fig|80869.199.peg.1509	T3S	gephyrin-like molybdotransferase Glp [*Paracidovorax citrulli*]
fig|80869.199.peg.1512	T3S	GTP cyclohydrolase subunit MoaA [*Paracidovorax citrulli* AAC00-1]
fig|80869.199.peg.1523	T3S	translesion DNA synthesis-associated protein ImuA [*Paracidovorax citrulli*]
fig|80869.199.peg.1527	T3S	pseudouridine synthase [*Paracidovorax citrulli*]
fig|80869.199.peg.1548	T3S	hypothetical protein CQB05_01060 [*Paracidovorax citrulli*]
fig|80869.199.peg.1558	T3S	DEAD/DEAH box helicase [*Paracidovorax citrulli*]
fig|80869.199.peg.160	T3S	hypothetical protein [*Paracidovorax citrulli*]
fig|80869.199.peg.1600	T3S	hypothetical protein [*Paracidovorax citrulli*]
fig|80869.199.peg.161	T3S	type III secretion system chaperone [*Paracidovorax citrulli*]
fig|80869.199.peg.1675	T3S	pyridoxamine 5'-phosphate oxidase [*Paracidovorax citrulli*]
fig|80869.199.peg.1686	T3S	transcriptional repressor [*Paracidovorax citrulli*]
fig|80869.199.peg.1688	T3S	TetR/AcrR family transcriptional regulator [*Paracidovorax citrulli*]
fig|80869.199.peg.1707	T3S	EVE domain-containing protein [*Paracidovorax citrulli*]
fig|80869.199.peg.1718	T3S	XopAP family type III secretion system effector [*Paracidovorax citrulli*]
fig|80869.199.peg.1811	T3S	glycoside hydrolase family 5 protein [*Paracidovorax citrulli*]
fig|80869.199.peg.1823	T3S	protein of unknown function UPF0061 [*Paracidovorax citrulli* AAC00-1]
fig|80869.199.peg.1903	T3S	BON domain-containing protein [*Paracidovorax citrulli*]
fig|80869.199.peg.1904	T3S	SulP family inorganic anion transporter [*Paracidovorax citrulli*]
fig|80869.199.peg.1912	T3S	NAD(P)/FAD-dependent oxidoreductase [*Paracidovorax citrulli*]
fig|80869.199.peg.1914	T3S	tRNA (guanosine(46)-N7)-methyltransferase TrmB [*Paracidovorax citrulli*]

Prot	Pred	Description
fig|80869.199.peg.1974	T3S	hypothetical protein [*Paracidovorax citrulli*]
fig|80869.199.peg.1981	T3S	ATP-binding protein [*Paracidovorax citrulli*]
fig|80869.199.peg.1991	T3S	YbaN family protein [*Paracidovorax citrulli*]
fig|80869.199.peg.2005	T3S	RDD domain containing protein [*Paracidovorax citrulli* AAC00-1]
fig|80869.199.peg.2024	T3S	TetR/AcrR family transcriptional regulator [*Paracidovorax citrulli*]
fig|80869.199.peg.2047	T3S	2OG-Fe dioxygenase family protein [*Paracidovorax citrulli*]
fig|80869.199.peg.2058	T3S	MULTISPECIES: CysB family HTH-type transcriptional regulator [Comamonadaceae]
fig|80869.199.peg.2113	T3S	DUF4230 domain-containing protein [*Paracidovorax citrulli*]
fig|80869.199.peg.2147	T3S	ABC transporter permease [*Paracidovorax citrulli*]
fig|80869.199.peg.2217	T3S	hypothetical protein [*Paracidovorax citrulli*]
fig|80869.199.peg.2219	T3S	L-proline dehydrogenase /delta-1-pyrroline-5-carboxylate dehydrogenase [*Paracidovorax citrulli*]
fig|80869.199.peg.2264	T3S	helix-turn-helix transcriptional regulator [*Paracidovorax citrulli*]
fig|80869.199.peg.2289	T3S	thioredoxin family protein [*Paracidovorax citrulli*]
fig|80869.199.peg.229	T3S	MATE family efflux transporter [*Paracidovorax citrulli*]
fig|80869.199.peg.2291	T3S	cytochrome c553-like protein [*Paracidovorax citrulli* AAC00-1]
fig|80869.199.peg.2307	T3S	ThiF family adenylyltransferase [*Paracidovorax citrulli*]
fig|80869.199.peg.2311	T3S	lipid A export permease/ATP-binding protein MsbA [*Paracidovorax citrulli*]
fig|80869.199.peg.2329	T3S	DUF72 domain-containing protein [*Paracidovorax citrulli*]
fig|80869.199.peg.2332	T3S	DUF1624 domain-containing protein [*Paracidovorax citrulli*]
fig|80869.199.peg.2333	T3S	glutamate--tRNA ligase [*Paracidovorax citrulli*]
fig|80869.199.peg.2366	T3S	UDP-3-O-(3-hydroxymyristoyl)glucosamine N-acyltransferase [*Paracidovorax citrulli*]
fig|80869.199.peg.2381	T3S	MlaD family protein [*Paracidovorax citrulli*]
fig|80869.199.peg.2385	T3S	YitT family protein [*Paracidovorax citrulli*]
fig|80869.199.peg.2396	T3S	P1 family peptidase [*Paracidovorax citrulli*]
fig|80869.199.peg.240	T3S	signal recognition particle-docking protein FtsY [*Paracidovorax citrulli*]
fig|80869.199.peg.2401	T3S	ATP synthase F1 subunit epsilon [*Paracidovorax citrulli*]
fig|80869.199.peg.2415	T3S	structural protein P5 [*Paracidovorax citrulli*]
fig|80869.199.peg.2418	T3S	hypothetical protein [*Paracidovorax citrulli*]
fig|80869.199.peg.25	T3S	TRAP transporter small permease [*Paracidovorax citrulli*]
fig|80869.199.peg.2526	T3S	hypothetical protein [*Paracidovorax citrulli*]
fig|80869.199.peg.2597	T3S	hypothetical protein [*Paracidovorax citrulli*]
fig|80869.199.peg.261	T3S	3-methyl-2-oxobutanoate hydroxymethyltransferase [*Paracidovorax citrulli*]
fig|80869.199.peg.2627	T3S	hypothetical protein Aave_1606 [*Paracidovorax citrulli* AAC00-1]
fig|80869.199.peg.2637	T3S	polyhydroxyalkanoate synthesis repressor PhaR [*Paracidovorax citrulli*]
fig|80869.199.peg.2639	T3S	type 1 glutamine amidotransferase domain-containing protein [*Paracidovorax citrulli*]
fig|80869.199.peg.2686	T3S	SDR family NAD(P)-dependent oxidoreductase [*Paracidovorax citrulli*]
fig|80869.199.peg.2716	T3S	cation-translocating P-type ATPase [*Paracidovorax citrulli*]
fig|80869.199.peg.2724	T3S	malonate--CoA ligase [*Paracidovorax citrulli*]
fig|80869.199.peg.2743	T3S	glycine betaine/L-proline ABC transporter permease ProW [*Paracidovorax citrulli*]
fig|80869.199.peg.276	T3S	hypothetical protein [*Paracidovorax citrulli*]
fig|80869.199.peg.277	T3S	YitT family protein [*Paracidovorax citrulli*]
fig|80869.199.peg.2785	T3S	farnesyl-diphosphate farnesyltransferase [*Paracidovorax citrulli* AAC00-1]
fig|80869.199.peg.2788	T3S	DUF2069 domain-containing protein [*Paracidovorax citrulli*]
fig|80869.199.peg.2813	T3S	GTPase HflX [*Paracidovorax citrulli*]

Prot	Pred	Description
fig|80869.199.peg.2828	T3S	protein-L-isoaspartate(D-aspartate) O-methyltransferase [*Paracidovorax citrulli*]
fig|80869.199.peg.2858	T3S	CaiB/BaiF CoA transferase family protein [*Paracidovorax citrulli*]
fig|80869.199.peg.289	T3S	hypothetical protein [*Paracidovorax citrulli*]
fig|80869.199.peg.2891	T3S	uncharacterized protein DUF2132 [*Paracidovorax citrulli*]
fig|80869.199.peg.2899	T3S	DNA internalization-related competence protein ComEC/Rec2 [*Paracidovorax citrulli*]
fig|80869.199.peg.2905	T3S	pyridoxal kinase PdxY [*Paracidovorax citrulli*]
fig|80869.199.peg.2931	T3S	hypothetical protein [*Paracidovorax citrulli*]
fig|80869.199.peg.2935	T3S	diguanylate phosphodiesterase [*Paracidovorax citrulli* AAC00-1]
fig|80869.199.peg.2956	T3S	winged helix-turn-helix transcriptional regulator [*Paracidovorax citrulli*]
fig|80869.199.peg.2974	T3S	ABC transporter transmembrane domain-containing protein [*Paracidovorax citrulli*]
fig|80869.199.peg.2982	T3S	MULTISPECIES: NADH-quinone oxidoreductase subunit NuoI [Comamonadaceae]
fig|80869.199.peg.3001	T3S	hypothetical protein [*Paracidovorax citrulli*]
fig|80869.199.peg.3013	T3S	molybdopterin converting factor subunit 1 [*Paracidovorax citrulli*]
fig|80869.199.peg.3028	T3S	nucleotide exchange factor GrpE [*Paracidovorax citrulli*]
fig|80869.199.peg.3046	T3S	LON peptidase substrate-binding domain-containing protein [*Paracidovorax citrulli*]
fig|80869.199.peg.3053	T3S	lysine--tRNA ligase [*Paracidovorax citrulli*]
fig|80869.199.peg.3054	T3S	Phytochrome-like protein cph2 [*Paracidovorax citrulli*]
fig|80869.199.peg.3106	T3S	tryptophan 2,3-dioxygenase [*Paracidovorax citrulli*]
fig|80869.199.peg.3115	T3S	neutral zinc metallopeptidase [*Paracidovorax citrulli*]
fig|80869.199.peg.3153	T3S	glycerate kinase [*Paracidovorax citrulli*]
fig|80869.199.peg.3169	T3S	NCS1 family nucleobase:cation symporter-1 [*Paracidovorax citrulli*]
fig|80869.199.peg.3171	T3S	GntR family transcriptional regulator [*Paracidovorax citrulli*]
fig|80869.199.peg.3190	T3S	DMT family transporter [*Paracidovorax citrulli*]
fig|80869.199.peg.32	T3S	hypothetical protein [*Paracidovorax citrulli*]
fig|80869.199.peg.3234	T3S	PelD GGDEF domain-containing protein [*Paracidovorax citrulli*]
fig|80869.199.peg.3271	T3S	protein of unknown function DUF1415 [*Paracidovorax citrulli* AAC00-1]
fig|80869.199.peg.3281	T3S	penicillin-binding protein 1A [*Paracidovorax citrulli*]
fig|80869.199.peg.3305	T3S	peptidoglycan-binding domain-containing protein [*Paracidovorax citrulli*]
fig|80869.199.peg.3308	T3S	NCS1 family nucleobase:cation symporter-1 [*Paracidovorax citrulli*]
fig|80869.199.peg.3340	T3S	ABC transporter ATP-binding protein [*Paracidovorax citrulli*]
fig|80869.199.peg.3373	T3S	Holliday junction resolvase RuvX [*Paracidovorax citrulli*]
fig|80869.199.peg.3382	T3S	hydroxymethylpyrimidine/phosphomethylpyrimidine kinase [*Paracidovorax citrulli*]
fig|80869.199.peg.3416	T3S	ribonucleoside-diphosphate reductase subunit alpha [*Paracidovorax citrulli*]
fig|80869.199.peg.3429	T3S	3-deoxy-7-phosphoheptulonate synthase [*Paracidovorax citrulli*]
fig|80869.199.peg.3475	T3S	CobW family GTP-binding protein [*Paracidovorax citrulli*]
fig|80869.199.peg.3486	T3S	chromate efflux transporter [*Paracidovorax citrulli*]
fig|80869.199.peg.3493	T3S	M48 family metallopeptidase [*Paracidovorax citrulli*]
fig|80869.199.peg.3508	T3S	DNA-3-methyladenine glycosylase I [*Paracidovorax citrulli*]
fig|80869.199.peg.3509	T3S	putative zinc protease protein [*Paracidovorax citrulli* AAC00-1]
fig|80869.199.peg.3586	T3S	FMN-dependent NADH-azoreductase [*Paracidovorax citrulli*]
fig|80869.199.peg.3631	T3S	NarK family nitrate/nitrite MFS transporter [*Paracidovorax citrulli*]
fig|80869.199.peg.3633	T3S	transcriptional regulator, AraC family [*Paracidovorax citrulli* AAC00-1]
fig|80869.199.peg.3646	T3S	FAD-linked oxidase C-terminal domain-containing protein [*Paracidovorax citrulli*]
fig|80869.199.peg.3691	T3S	pyrroline-5-carboxylate reductase [*Paracidovorax citrulli*]

Prot	Pred	Description
fig|80869.199.peg.3693	T3S	glycosyltransferase involved in cell wall biosynthesis [*Paracidovorax citrulli*]
fig|80869.199.peg.3707	T3S	uracil-DNA glycosylase [*Paracidovorax citrulli*]
fig|80869.199.peg.3745	T3S	HNH endonuclease [*Paracidovorax citrulli*]
fig|80869.199.peg.3765	T3S	MULTISPECIES: hypothetical protein [Pseudomonadota]
fig|80869.199.peg.3831	T3S	hypothetical protein [*Paracidovorax citrulli*]
fig|80869.199.peg.3837	T3S	hypothetical protein [*Paracidovorax citrulli*]
fig|80869.199.peg.3848	T3S	hypothetical protein [*Paracidovorax citrulli*]
fig|80869.199.peg.3849	T3S	hypothetical protein [*Paracidovorax citrulli*]
fig|80869.199.peg.3853	T3S	hypothetical protein Aave_0457 [*Paracidovorax citrulli* AAC00-1]
fig|80869.199.peg.3880	T3S	DUF4139 domain-containing protein [*Paracidovorax citrulli*]
fig|80869.199.peg.3887	T3S	hypothetical protein [*Paracidovorax citrulli*]
fig|80869.199.peg.3892	T3S	TspO/MBR family protein [*Paracidovorax citrulli*]
fig|80869.199.peg.3927	T3S	Kelch repeat-containing protein [*Paracidovorax citrulli*]
fig|80869.199.peg.3935	T3S	TPM domain-containing protein [*Paracidovorax citrulli*]
fig|80869.199.peg.3958	T3S	potassium-transporting ATPase subunit KdpB [*Paracidovorax citrulli*]
fig|80869.199.peg.1054	T4S	hypothetical protein [*Paracidovorax citrulli*]
fig|80869.199.peg.1166	T4S	major facilitator superfamily MFS_1 [*Paracidovorax citrulli* AAC00-1]
fig|80869.199.peg.3275	T4S	Ig domain protein, group 1 domain protein [*Paracidovorax citrulli* AAC00-1]
fig|80869.199.peg.3755	T4S	MULTISPECIES: DUF3577 domain-containing protein [Pseudomonadota]
fig|80869.199.peg.973	T4S	acyl-CoA thioesterase [*Paracidovorax citrulli*]
fig|80869.199.peg.1253	T4S	STY0301 family protein [*Paracidovorax citrulli*]
fig|80869.199.peg.1843	T4S	ABC transporter ATP-binding protein [*Paracidovorax citrulli*]
fig|80869.199.peg.1366	T4S	Ku protein [*Paracidovorax citrulli*]
fig|80869.199.peg.2523	T4S	terminase small subunit [*Paracidovorax citrulli*]
fig|80869.199.peg.3007	T4S	molybdenum cofactor biosynthesis protein MoaE [*Paracidovorax citrulli*]
fig|80869.199.peg.247	T4S	bacterioferritin [*Paracidovorax citrulli*]
fig|80869.199.peg.2572	T4S	isoaspartyl peptidase/L-asparaginase family protein [*Paracidovorax citrulli*]
fig|80869.199.peg.349	T4S	abortive infection system antitoxin AbiGi family protein [*Paracidovorax citrulli*]
fig|80869.199.peg.1017	T4S	RNA recognition motif domain-containing protein [*Paracidovorax citrulli*]
fig|80869.199.peg.1549	T4S	hypothetical protein [*Paracidovorax citrulli*]
fig|80869.199.peg.1958	T4S	hypothetical protein Aave_3072 [*Paracidovorax citrulli* AAC00-1]
fig|80869.199.peg.1713	T4S	DUF1795 domain-containing protein [*Paracidovorax citrulli*]
fig|80869.199.peg.3834	T4S	type III secretion system outer membrane ring subunit SctC [*Paracidovorax citrulli*]
fig|80869.199.peg.1032	T4S	hypothetical protein [*Paracidovorax citrulli*]
fig|80869.199.peg.1402	T4S	MULTISPECIES: amino-acid N-acetyltransferase [Paracidovorax]
fig|80869.199.peg.4567	T4S	DUF924 family protein [*Paracidovorax citrulli*]
fig|80869.199.peg.417	T4S	hypothetical protein [*Paracidovorax citrulli*]
fig|80869.199.peg.4630	T4S	50S ribosomal protein L11 [*Paracidovorax citrulli*]
fig|80869.199.peg.1369	T4S	hypothetical protein [*Paracidovorax citrulli*]
fig|80869.199.peg.1141	T4S	conserved hypothetical protein [*Paracidovorax citrulli* AAC00-1]
fig|80869.199.peg.568	T4S	PP2C family protein-serine/threonine phosphatase [*Paracidovorax citrulli*]
fig|80869.199.peg.194	T4S	glutathione-regulated potassium-efflux system protein KefC [*Paracidovorax citrulli*]
fig|80869.199.peg.4251	T4S	hypothetical protein [*Paracidovorax citrulli*]
fig|80869.199.peg.4649	T4S	cryptochrome/photolyase family protein [*Paracidovorax citrulli*]
fig|80869.199.peg.2929	T4S	phosphopyruvate hydratase [*Paracidovorax citrulli*]

Prot	Pred	Description
fig|80869.199.peg.625	T4S	hypothetical protein [*Paracidovorax citrulli*]
fig|80869.199.peg.2157	T4S	hypothetical protein [*Paracidovorax citrulli*]
fig|80869.199.peg.2353	T4S	AAA family ATPase [*Paracidovorax citrulli*]
fig|80869.199.peg.4861	T4S	DUF3025 domain-containing protein [*Paracidovorax citrulli*]
fig|80869.199.peg.567	T4S	serine/threonine protein kinase [*Paracidovorax citrulli*]
fig|80869.199.peg.941	T4S	inositol monophosphatase family protein [*Paracidovorax citrulli*]
fig|80869.199.peg.4936	T4S	N-acetylmuramoyl-L-alanine amidase [*Paracidovorax citrulli*]
fig|80869.199.peg.3701	T4S	AraC family transcriptional regulator [*Paracidovorax citrulli*]
fig|80869.199.peg.2825	T4S	AraC family transcriptional regulator [*Paracidovorax citrulli*]
fig|80869.199.peg.2215	T4S	hypothetical protein [*Paracidovorax citrulli*]
fig|80869.199.peg.4531	T4S	Fic family protein [*Paracidovorax citrulli*]
fig|80869.199.peg.342	T4S	4-hydroxy-3-methylbut-2-enyl diphosphate reductase [*Paracidovorax citrulli*]
fig|80869.199.peg.2276	T4S	SsrA-binding protein SmpB [*Paracidovorax citrulli*]
fig|80869.199.peg.1794	T4S	hypothetical protein [*Paracidovorax citrulli*]
fig|80869.199.peg.3943	T4S	F0F1 ATP synthase subunit gamma [*Paracidovorax citrulli*]
fig|80869.199.peg.789	T4S	hypothetical protein [*Paracidovorax citrulli*]
fig|80869.199.peg.2798	T4S	hypothetical protein [*Paracidovorax citrulli*]
fig|80869.199.peg.1503	T4S	transglycosylase SLT domain-containing protein [*Paracidovorax citrulli*]
fig|80869.199.peg.5073	T4S	hypothetical protein [*Paracidovorax citrulli*]
fig|80869.199.peg.2504	T4S	hypothetical protein [*Paracidovorax citrulli*]
fig|80869.199.peg.132	T4S	5'-nucleotidase [*Paracidovorax citrulli*]
fig|80869.199.peg.3666	T4S	30S ribosomal protein S4 [*Paracidovorax citrulli*]
fig|80869.199.peg.1412	T4S	adenylate kinase [*Paracidovorax citrulli*]
fig|80869.199.peg.2273	T4S	DUF4124 domain-containing protein [*Paracidovorax citrulli*]
fig|80869.199.peg.2138	T4S	hypothetical protein [*Paracidovorax citrulli*]
fig|80869.199.peg.598	T4S	type IV pilin protein [*Paracidovorax citrulli*]
fig|80869.199.peg.4841	T4S	hypothetical protein [*Paracidovorax citrulli*]
fig|80869.199.peg.246	T4S	BON domain-containing protein [*Paracidovorax citrulli*]
fig|80869.199.peg.1388	T4S	MetQ/NlpA family ABC transporter substrate-binding protein [*Paracidovorax citrulli*]
fig|80869.199.peg.4701	T4S	Bug family tripartite tricarboxylate transporter substrate binding protein [*Paracidovorax citrulli*]
fig|80869.199.peg.1583	T4S	hypothetical protein [*Paracidovorax citrulli*]
fig|80869.199.peg.1394	T4S	sulfate ABC transporter substrate-binding protein [*Paracidovorax citrulli*]
fig|80869.199.peg.367	T4S	hypothetical protein [*Paracidovorax citrulli*]
fig|80869.199.peg.4568	T4S	OmpA family protein [*Paracidovorax citrulli*]
fig|80869.199.peg.2486	T4S	glycoside hydrolase family protein [*Paracidovorax citrulli*]
fig|80869.199.peg.4017	T4S	DNA-binding transcriptional LysR family regulator [*Paracidovorax citrulli*]
fig|80869.199.peg.758	T4S	sigma-54-dependent transcriptional regulator [*Paracidovorax citrulli*]
fig|80869.199.peg.131	T4S	EF-hand domain-containing protein [*Paracidovorax citrulli*]

*P_citrulli_*KACC18784

Prot	Pred	Description
fig|80869.198.peg.1035	T3S	hypothetical protein [*Paracidovorax citrulli*]
fig|80869.198.peg.1146	T3S	major facilitator superfamily MFS_1 [*Paracidovorax citrulli* AAC00-1]
fig|80869.198.peg.3326	T3S	Ig domain protein, group 1 domain protein [*Paracidovorax citrulli* AAC00-1]

Prot	Pred	Description
fig|80869.198.peg.3805	T3S	MULTISPECIES: DUF3577 domain-containing protein [Pseudomonadota]
fig|80869.198.peg.952	T3S	acyl-CoA thioesterase [*Paracidovorax citrulli*]
fig|80869.198.peg.1308	T3S	STY0301 family protein [*Paracidovorax citrulli*]
fig|80869.198.peg.1893	T3S	ABC transporter ATP-binding protein [*Paracidovorax citrulli*]
fig|80869.198.peg.1422	T3S	Ku protein [*Paracidovorax citrulli*]
fig|80869.198.peg.2578	T3S	terminase small subunit [*Paracidovorax citrulli*]
fig|80869.198.peg.1005	T3S	hypothetical protein Aave_2148 [*Paracidovorax citrulli* AAC00-1]
fig|80869.198.peg.101	T3S	ABC transporter permease [*Paracidovorax citrulli*]
fig|80869.198.peg.102	T3S	ABC transporter permease [*Paracidovorax citrulli*]
fig|80869.198.peg.1033	T3S	hypothetical protein [*Paracidovorax citrulli*]
fig|80869.198.peg.1050	T3S	NAD-dependent succinate-semialdehyde dehydrogenase [*Paracidovorax citrulli*]
fig|80869.198.peg.1064	T3S	transcriptional regulator, GntR family [*Paracidovorax citrulli* AAC00-1]
fig|80869.198.peg.1113	T3S	multiple monosaccharide ABC transporter permease [*Paracidovorax citrulli*]
fig|80869.198.peg.1119	T3S	SDR family NAD(P)-dependent oxidoreductase [*Paracidovorax citrulli*]
fig|80869.198.peg.1120	T3S	SMP-30/gluconolactonase/LRE family protein [*Paracidovorax citrulli*]
fig|80869.198.peg.1127	T3S	GAF domain-containing sensor histidine kinase [*Paracidovorax citrulli*]
fig|80869.198.peg.1134	T3S	MFS transporter [*Paracidovorax citrulli*]
fig|80869.198.peg.1140	T3S	DHH family phosphoesterase [*Paracidovorax citrulli*]
fig|80869.198.peg.1150	T3S	Murein DD-endopeptidase MepM [*Paracidovorax citrulli*]
fig|80869.198.peg.1153	T3S	esterase/lipase/thioesterase family protein [*Paracidovorax citrulli* AAC00-1]
fig|80869.198.peg.116	T3S	hypothetical protein [*Paracidovorax citrulli*]
fig|80869.198.peg.1165	T3S	glutamine--tRNA ligase/YqeY domain fusion protein [*Paracidovorax citrulli*]
fig|80869.198.peg.1170	T3S	DODA-type extradiol aromatic ring-opening family dioxygenase [*Paracidovorax citrulli*]
fig|80869.198.peg.1279	T3S	hypothetical protein [*Paracidovorax citrulli*]
fig|80869.198.peg.1300	T3S	KGG domain-containing protein [*Paracidovorax citrulli*]
fig|80869.198.peg.132	T3S	luciferase family protein [*Paracidovorax citrulli* AAC00-1]
fig|80869.198.peg.1349	T3S	PAS domain-containing sensor histidine kinase [*Paracidovorax citrulli*]
fig|80869.198.peg.1383	T3S	EAL domain-containing protein [*Paracidovorax citrulli*]
fig|80869.198.peg.1504	T3S	TonB-dependent receptor family protein [*Paracidovorax citrulli*]
fig|80869.198.peg.1514	T3S	D-serine/D-alanine/glycine transporter [*Paracidovorax citrulli*]
fig|80869.198.peg.1515	T3S	hypothetical protein [*Paracidovorax citrulli*]
fig|80869.198.peg.1528	T3S	Polyphosphate kinase [*Paracidovorax citrulli* AAC00-1]
fig|80869.198.peg.1533	T3S	phosphate ABC transporter permease PstC [*Paracidovorax citrulli*]
fig|80869.198.peg.1562	T3S	gephyrin-like molybdotransferase Glp [*Paracidovorax citrulli*]
fig|80869.198.peg.1565	T3S	GTP cyclohydrolase subunit MoaA [*Paracidovorax citrulli* AAC00-1]
fig|80869.198.peg.1576	T3S	translesion DNA synthesis-associated protein ImuA [*Paracidovorax citrulli*]
fig|80869.198.peg.1580	T3S	pseudouridine synthase [*Paracidovorax citrulli*]
fig|80869.198.peg.159	T3S	hypothetical protein [*Paracidovorax citrulli*]
fig|80869.198.peg.160	T3S	type III secretion system chaperone [*Paracidovorax citrulli*]
fig|80869.198.peg.1601	T3S	hypothetical protein CQB05_01060 [*Paracidovorax citrulli*]
fig|80869.198.peg.1611	T3S	DEAD/DEAH box helicase [*Paracidovorax citrulli*]
fig|80869.198.peg.1653	T3S	hypothetical protein [*Paracidovorax citrulli*]
fig|80869.198.peg.1725	T3S	pyridoxamine 5'-phosphate oxidase [*Paracidovorax citrulli*]
fig|80869.198.peg.1736	T3S	transcriptional repressor [*Paracidovorax citrulli*]
fig|80869.198.peg.1738	T3S	TetR/AcrR family transcriptional regulator [*Paracidovorax citrulli*]

Prot	Pred	Description
fig|80869.198.peg.1757	T3S	EVE domain-containing protein [*Paracidovorax citrulli*]
fig|80869.198.peg.1768	T3S	XopAP family type III secretion system effector [*Paracidovorax citrulli*]
fig|80869.198.peg.1861	T3S	glycoside hydrolase family 5 protein [*Paracidovorax citrulli*]
fig|80869.198.peg.1873	T3S	protein of unknown function UPF0061 [*Paracidovorax citrulli* AAC00-1]
fig|80869.198.peg.1952	T3S	BON domain-containing protein [*Paracidovorax citrulli*]
fig|80869.198.peg.1953	T3S	SulP family inorganic anion transporter [*Paracidovorax citrulli*]
fig|80869.198.peg.1961	T3S	NAD(P)/FAD-dependent oxidoreductase [*Paracidovorax citrulli*]
fig|80869.198.peg.1963	T3S	tRNA (guanosine(46)-N7)-methyltransferase TrmB [*Paracidovorax citrulli*]
fig|80869.198.peg.2023	T3S	hypothetical protein [*Paracidovorax citrulli*]
fig|80869.198.peg.2030	T3S	ATP-binding protein [*Paracidovorax citrulli*]
fig|80869.198.peg.2040	T3S	YbaN family protein [*Paracidovorax citrulli*]
fig|80869.198.peg.2054	T3S	RDD domain containing protein [*Paracidovorax citrulli* AAC00-1]
fig|80869.198.peg.2074	T3S	TetR/AcrR family transcriptional regulator [*Paracidovorax citrulli*]
fig|80869.198.peg.2097	T3S	2OG-Fe dioxygenase family protein [*Paracidovorax citrulli*]
fig|80869.198.peg.2108	T3S	MULTISPECIES: CysB family HTH-type transcriptional regulator [Comamonadaceae]
fig|80869.198.peg.2165	T3S	DUF4230 domain-containing protein [*Paracidovorax citrulli*]
fig|80869.198.peg.2200	T3S	ABC transporter permease [*Paracidovorax citrulli*]
fig|80869.198.peg.227	T3S	MATE family efflux transporter [*Paracidovorax citrulli*]
fig|80869.198.peg.2272	T3S	hypothetical protein [*Paracidovorax citrulli*]
fig|80869.198.peg.2274	T3S	L-proline dehydrogenase /delta-1-pyrroline-5-carboxylate dehydrogenase [*Paracidovorax citrulli*]
fig|80869.198.peg.2319	T3S	helix-turn-helix transcriptional regulator [*Paracidovorax citrulli*]
fig|80869.198.peg.2336	T3S	hypothetical protein [*Paracidovorax citrulli*]
fig|80869.198.peg.2344	T3S	thioredoxin family protein [*Paracidovorax citrulli*]
fig|80869.198.peg.2346	T3S	cytochrome c553-like protein [*Paracidovorax citrulli* AAC00-1]
fig|80869.198.peg.2362	T3S	ThiF family adenylyltransferase [*Paracidovorax citrulli*]
fig|80869.198.peg.2366	T3S	lipid A export permease/ATP-binding protein MsbA [*Paracidovorax citrulli*]
fig|80869.198.peg.238	T3S	signal recognition particle-docking protein FtsY [*Paracidovorax citrulli*]
fig|80869.198.peg.2384	T3S	DUF72 domain-containing protein [*Paracidovorax citrulli*]
fig|80869.198.peg.2387	T3S	DUF1624 domain-containing protein [*Paracidovorax citrulli*]
fig|80869.198.peg.2388	T3S	glutamate--tRNA ligase [*Paracidovorax citrulli*]
fig|80869.198.peg.24	T3S	TRAP transporter small permease [*Paracidovorax citrulli*]
fig|80869.198.peg.2421	T3S	UDP-3-O-(3-hydroxymyristoyl)glucosamine N-acyltransferase [*Paracidovorax citrulli*]
fig|80869.198.peg.2436	T3S	MlaD family protein [*Paracidovorax citrulli*]
fig|80869.198.peg.2440	T3S	YitT family protein [*Paracidovorax citrulli*]
fig|80869.198.peg.2451	T3S	P1 family peptidase [*Paracidovorax citrulli*]
fig|80869.198.peg.2456	T3S	ATP synthase F1 subunit epsilon [*Paracidovorax citrulli*]
fig|80869.198.peg.2470	T3S	structural protein P5 [*Paracidovorax citrulli*]
fig|80869.198.peg.2473	T3S	hypothetical protein [*Paracidovorax citrulli*]
fig|80869.198.peg.2581	T3S	hypothetical protein [*Paracidovorax citrulli*]
fig|80869.198.peg.259	T3S	3-methyl-2-oxobutanoate hydroxymethyltransferase [*Paracidovorax citrulli*]
fig|80869.198.peg.2594	T3S	hypothetical protein Aave_1685 [*Paracidovorax citrulli* AAC00-1]
fig|80869.198.peg.2652	T3S	hypothetical protein [*Paracidovorax citrulli*]
fig|80869.198.peg.2682	T3S	hypothetical protein Aave_1606 [*Paracidovorax citrulli* AAC00-1]
fig|80869.198.peg.2692	T3S	polyhydroxyalkanoate synthesis repressor PhaR [*Paracidovorax citrulli*]
fig|80869.198.peg.2694	T3S	type 1 glutamine amidotransferase domain-containing protein [*Paracidovorax citrulli*]

Prot	Pred	Description
fig|80869.198.peg.274	T3S	hypothetical protein [*Paracidovorax citrulli*]
fig|80869.198.peg.2741	T3S	SDR family NAD(P)-dependent oxidoreductase [*Paracidovorax citrulli*]
fig|80869.198.peg.275	T3S	YitT family protein [*Paracidovorax citrulli*]
fig|80869.198.peg.2771	T3S	cation-translocating P-type ATPase [*Paracidovorax citrulli*]
fig|80869.198.peg.2778	T3S	malonate--CoA ligase [*Paracidovorax citrulli*]
fig|80869.198.peg.2798	T3S	glycine betaine/L-proline ABC transporter permease ProW [*Paracidovorax citrulli*]
fig|80869.198.peg.2840	T3S	farnesyl-diphosphate farnesyltransferase [*Paracidovorax citrulli* AAC00-1]
fig|80869.198.peg.2843	T3S	DUF2069 domain-containing protein [*Paracidovorax citrulli*]
fig|80869.198.peg.2868	T3S	GTPase HflX [*Paracidovorax citrulli*]
fig|80869.198.peg.287	T3S	hypothetical protein [*Paracidovorax citrulli*]
fig|80869.198.peg.2883	T3S	protein-L-isoaspartate(D-aspartate) O-methyltransferase [*Paracidovorax citrulli*]
fig|80869.198.peg.2911	T3S	CaiB/BaiF CoA transferase family protein [*Paracidovorax citrulli*]
fig|80869.198.peg.2944	T3S	uncharacterized protein DUF2132 [*Paracidovorax citrulli*]
fig|80869.198.peg.2952	T3S	DNA internalization-related competence protein ComEC/Rec2 [*Paracidovorax citrulli*]
fig|80869.198.peg.2958	T3S	pyridoxal kinase PdxY [*Paracidovorax citrulli*]
fig|80869.198.peg.2984	T3S	hypothetical protein [*Paracidovorax citrulli*]
fig|80869.198.peg.2988	T3S	diguanylate phosphodiesterase [*Paracidovorax citrulli* AAC00-1]
fig|80869.198.peg.3009	T3S	winged helix-turn-helix transcriptional regulator [*Paracidovorax citrulli*]
fig|80869.198.peg.3027	T3S	ABC transporter transmembrane domain-containing protein [*Paracidovorax citrulli*]
fig|80869.198.peg.3035	T3S	MULTISPECIES: NADH-quinone oxidoreductase subunit NuoI [Comamonadaceae]
fig|80869.198.peg.3054	T3S	hypothetical protein [*Paracidovorax citrulli*]
fig|80869.198.peg.3066	T3S	molybdopterin converting factor subunit 1 [*Paracidovorax citrulli*]
fig|80869.198.peg.3081	T3S	nucleotide exchange factor GrpE [*Paracidovorax citrulli*]
fig|80869.198.peg.3099	T3S	LON peptidase substrate-binding domain-containing protein [*Paracidovorax citrulli*]
fig|80869.198.peg.31	T3S	hypothetical protein [*Paracidovorax citrulli*]
fig|80869.198.peg.3106	T3S	lysine--tRNA ligase [*Paracidovorax citrulli*]
fig|80869.198.peg.3107	T3S	Phytochrome-like protein cph2 [*Paracidovorax citrulli*]
fig|80869.198.peg.3158	T3S	tryptophan 2,3-dioxygenase [*Paracidovorax citrulli*]
fig|80869.198.peg.3167	T3S	neutral zinc metallopeptidase [*Paracidovorax citrulli*]
fig|80869.198.peg.3205	T3S	glycerate kinase [*Paracidovorax citrulli*]
fig|80869.198.peg.3221	T3S	NCS1 family nucleobase:cation symporter-1 [*Paracidovorax citrulli*]
fig|80869.198.peg.3223	T3S	GntR family transcriptional regulator [*Paracidovorax citrulli*]
fig|80869.198.peg.3242	T3S	DMT family transporter [*Paracidovorax citrulli*]
fig|80869.198.peg.3285	T3S	PelD GGDEF domain-containing protein [*Paracidovorax citrulli*]
fig|80869.198.peg.3322	T3S	protein of unknown function DUF1415 [*Paracidovorax citrulli* AAC00-1]
fig|80869.198.peg.3332	T3S	penicillin-binding protein 1A [*Paracidovorax citrulli*]
fig|80869.198.peg.3354	T3S	hypothetical protein Aave_0973 [*Paracidovorax citrulli* AAC00-1]
fig|80869.198.peg.3356	T3S	peptidoglycan-binding domain-containing protein [*Paracidovorax citrulli*]
fig|80869.198.peg.3359	T3S	NCS1 family nucleobase:cation symporter-1 [*Paracidovorax citrulli*]
fig|80869.198.peg.3392	T3S	ABC transporter ATP-binding protein [*Paracidovorax citrulli*]
fig|80869.198.peg.3424	T3S	Holliday junction resolvase RuvX [*Paracidovorax citrulli*]
fig|80869.198.peg.3433	T3S	hydroxymethylpyrimidine/phosphomethylpyrimidine kinase [*Paracidovorax citrulli*]
fig|80869.198.peg.3467	T3S	ribonucleoside-diphosphate reductase subunit alpha [*Paracidovorax citrulli*]
fig|80869.198.peg.3479	T3S	3-deoxy-7-phosphoheptulonate synthase [*Paracidovorax citrulli*]

Prot	Pred	Description
fig|80869.198.peg.3525	T3S	CobW family GTP-binding protein [*Paracidovorax citrulli*]
fig|80869.198.peg.3536	T3S	chromate efflux transporter [*Paracidovorax citrulli*]
fig|80869.198.peg.3543	T3S	M48 family metallopeptidase [*Paracidovorax citrulli*]
fig|80869.198.peg.3558	T3S	DNA-3-methyladenine glycosylase I [*Paracidovorax citrulli*]
fig|80869.198.peg.3559	T3S	putative zinc protease protein [*Paracidovorax citrulli* AAC00-1]
fig|80869.198.peg.3638	T3S	FMN-dependent NADH-azoreductase [*Paracidovorax citrulli*]
fig|80869.198.peg.3684	T3S	NarK family nitrate/nitrite MFS transporter [*Paracidovorax citrulli*]
fig|80869.198.peg.3686	T3S	transcriptional regulator, AraC family [*Paracidovorax citrulli* AAC00-1]
fig|80869.198.peg.3699	T3S	FAD-linked oxidase C-terminal domain-containing protein [*Paracidovorax citrulli*]
fig|80869.198.peg.3744	T3S	pyrroline-5-carboxylate reductase [*Paracidovorax citrulli*]
fig|80869.198.peg.3745	T3S	glycosyltransferase family 39 protein [*Paracidovorax citrulli*]
fig|80869.198.peg.3746	T3S	glycosyltransferase involved in cell wall biosynthesis [*Paracidovorax citrulli*]
fig|80869.198.peg.3760	T3S	uracil-DNA glycosylase [*Paracidovorax citrulli*]
fig|80869.198.peg.3795	T3S	HNH endonuclease [*Paracidovorax citrulli*]
fig|80869.198.peg.3815	T3S	MULTISPECIES: hypothetical protein [Pseudomonadota]
fig|80869.198.peg.3865	T3S	hypothetical protein [*Paracidovorax citrulli*]
fig|80869.198.peg.3871	T3S	hypothetical protein [*Paracidovorax citrulli*]
fig|80869.198.peg.3882	T3S	hypothetical protein [*Paracidovorax citrulli*]
fig|80869.198.peg.3883	T3S	hypothetical protein [*Paracidovorax citrulli*]
fig|80869.198.peg.3887	T3S	hypothetical protein Aave_0457 [*Paracidovorax citrulli* AAC00-1]
fig|80869.198.peg.3915	T3S	DUF4139 domain-containing protein [*Paracidovorax citrulli*]
fig|80869.198.peg.3922	T3S	hypothetical protein [*Paracidovorax citrulli*]
fig|80869.198.peg.3927	T3S	TspO/MBR family protein [*Paracidovorax citrulli*]
fig|80869.198.peg.394	T3S	GNAT family N-acetyltransferase [*Paracidovorax citrulli*]
fig|80869.198.peg.1035	T4S	hypothetical protein [*Paracidovorax citrulli*]
fig|80869.198.peg.1146	T4S	major facilitator superfamily MFS_1 [*Paracidovorax citrulli* AAC00-1]
fig|80869.198.peg.3326	T4S	Ig domain protein, group 1 domain protein [*Paracidovorax citrulli* AAC00-1]
fig|80869.198.peg.3805	T4S	MULTISPECIES: DUF3577 domain-containing protein [Pseudomonadota]
fig|80869.198.peg.952	T4S	acyl-CoA thioesterase [*Paracidovorax citrulli*]
fig|80869.198.peg.1308	T4S	STY0301 family protein [*Paracidovorax citrulli*]
fig|80869.198.peg.1893	T4S	ABC transporter ATP-binding protein [*Paracidovorax citrulli*]
fig|80869.198.peg.1422	T4S	Ku protein [*Paracidovorax citrulli*]
fig|80869.198.peg.2578	T4S	terminase small subunit [*Paracidovorax citrulli*]
fig|80869.198.peg.3060	T4S	molybdenum cofactor biosynthesis protein MoaE [*Paracidovorax citrulli*]
fig|80869.198.peg.245	T4S	bacterioferritin [*Paracidovorax citrulli*]
fig|80869.198.peg.2627	T4S	isoaspartyl peptidase/L-asparaginase family protein [*Paracidovorax citrulli*]
fig|80869.198.peg.4770	T4S	hypothetical protein [*Paracidovorax citrulli*]
fig|80869.198.peg.346	T4S	abortive infection system antitoxin AbiGi family protein [*Paracidovorax citrulli*]
fig|80869.198.peg.997	T4S	RNA recognition motif domain-containing protein [*Paracidovorax citrulli*]
fig|80869.198.peg.1602	T4S	hypothetical protein [*Paracidovorax citrulli*]
fig|80869.198.peg.2007	T4S	hypothetical protein Aave_3072 [*Paracidovorax citrulli* AAC00-1]
fig|80869.198.peg.1763	T4S	DUF1795 domain-containing protein [*Paracidovorax citrulli*]
fig|80869.198.peg.3868	T4S	type III secretion system outer membrane ring subunit SctC [*Paracidovorax citrulli*]
fig|80869.198.peg.1012	T4S	hypothetical protein [*Paracidovorax citrulli*]
fig|80869.198.peg.1457	T4S	MULTISPECIES: amino-acid N-acetyltransferase [Paracidovorax]

Prot	Pred	Description
fig|80869.198.peg.4602	T4S	DUF924 family protein [*Paracidovorax citrulli*]
fig|80869.198.peg.409	T4S	hypothetical protein [*Paracidovorax citrulli*]
fig|80869.198.peg.4664	T4S	50S ribosomal protein L11 [*Paracidovorax citrulli*]
fig|80869.198.peg.1425	T4S	hypothetical protein [*Paracidovorax citrulli*]
fig|80869.198.peg.1121	T4S	conserved hypothetical protein [*Paracidovorax citrulli* AAC00-1]
fig|80869.198.peg.545	T4S	PP2C family protein-serine/threonine phosphatase [*Paracidovorax citrulli*]
fig|80869.198.peg.192	T4S	glutathione-regulated potassium-efflux system protein KefC [*Paracidovorax citrulli*]
fig|80869.198.peg.4285	T4S	hypothetical protein [*Paracidovorax citrulli*]
fig|80869.198.peg.4683	T4S	cryptochrome/photolyase family protein [*Paracidovorax citrulli*]
fig|80869.198.peg.2982	T4S	phosphopyruvate hydratase [*Paracidovorax citrulli*]
fig|80869.198.peg.601	T4S	hypothetical protein [*Paracidovorax citrulli*]
fig|80869.198.peg.2210	T4S	hypothetical protein [*Paracidovorax citrulli*]
fig|80869.198.peg.2408	T4S	AAA family ATPase [*Paracidovorax citrulli*]
fig|80869.198.peg.544	T4S	serine/threonine protein kinase [*Paracidovorax citrulli*]
fig|80869.198.peg.919	T4S	inositol monophosphatase family protein [*Paracidovorax citrulli*]
fig|80869.198.peg.4968	T4S	N-acetylmuramoyl-L-alanine amidase [*Paracidovorax citrulli*]
fig|80869.198.peg.3754	T4S	AraC family transcriptional regulator [*Paracidovorax citrulli*]
fig|80869.198.peg.2880	T4S	SMC-Scp complex subunit ScpB [*Paracidovorax citrulli*]
fig|80869.198.peg.2270	T4S	hypothetical protein [*Paracidovorax citrulli*]
fig|80869.198.peg.4566	T4S	Fic family protein [*Paracidovorax citrulli*]
fig|80869.198.peg.339	T4S	4-hydroxy-3-methylbut-2-enyl diphosphate reductase [*Paracidovorax citrulli*]
fig|80869.198.peg.2331	T4S	SsrA-binding protein SmpB [*Paracidovorax citrulli*]
fig|80869.198.peg.1844	T4S	hypothetical protein [*Paracidovorax citrulli*]
fig|80869.198.peg.3979	T4S	F0F1 ATP synthase subunit gamma [*Paracidovorax citrulli*]
fig|80869.198.peg.766	T4S	hypothetical protein [*Paracidovorax citrulli*]
fig|80869.198.peg.2853	T4S	hypothetical protein [*Paracidovorax citrulli*]
fig|80869.198.peg.1556	T4S	transglycosylase SLT domain-containing protein [*Paracidovorax citrulli*]
fig|80869.198.peg.5103	T4S	hypothetical protein [*Paracidovorax citrulli*]
fig|80869.198.peg.2559	T4S	hypothetical protein [*Paracidovorax citrulli*]
fig|80869.198.peg.130	T4S	5'-nucleotidase [*Paracidovorax citrulli*]
fig|80869.198.peg.1237	T4S	hypothetical protein [*Paracidovorax citrulli*]
fig|80869.198.peg.3719	T4S	30S ribosomal protein S4 [*Paracidovorax citrulli*]
fig|80869.198.peg.1466	T4S	adenylate kinase [*Paracidovorax citrulli*]
fig|80869.198.peg.2191	T4S	hypothetical protein [*Paracidovorax citrulli*]
fig|80869.198.peg.575	T4S	type IV pilin protein [*Paracidovorax citrulli*]
fig|80869.198.peg.4876	T4S	hypothetical protein [*Paracidovorax citrulli*]
fig|80869.198.peg.244	T4S	BON domain-containing protein [*Paracidovorax citrulli*]
fig|80869.198.peg.1443	T4S	MetQ/NlpA family ABC transporter substrate-binding protein [*Paracidovorax citrulli*]
fig|80869.198.peg.4735	T4S	Bug family tripartite tricarboxylate transporter substrate binding protein [*Paracidovorax citrulli*]
fig|80869.198.peg.1636	T4S	hypothetical protein [*Paracidovorax citrulli*]
fig|80869.198.peg.1449	T4S	sulfate ABC transporter substrate-binding protein [*Paracidovorax citrulli*]
fig|80869.198.peg.364	T4S	hypothetical protein [*Paracidovorax citrulli*]
fig|80869.198.peg.4603	T4S	OmpA family protein [*Paracidovorax citrulli*]
fig|80869.198.peg.2541	T4S	glycoside hydrolase family protein [*Paracidovorax citrulli*]
fig|80869.198.peg.4053	T4S	DNA-binding transcriptional LysR family regulator [*Paracidovorax citrulli*]

Prot	Pred	Description
fig|80869.198.peg.734	T4S	sigma-54-dependent transcriptional regulator [*Paracidovorax citrulli*]
fig|80869.198.peg.129	T4S	EF-hand domain-containing protein [*Paracidovorax citrulli*]

*P_citrulli_*AAC00-1

Prot	Pred	Description
fig|80869.170.peg.1062	T3S	Ig domain protein, group 1 domain protein [*Paracidovorax citrulli* AAC00-1]
fig|80869.170.peg.2342	T3S	hypothetical protein [*Paracidovorax citrulli*]
fig|80869.170.peg.2454	T3S	major facilitator superfamily MFS_1 [*Paracidovorax citrulli* AAC00-1]
fig|80869.170.peg.584	T3S	MULTISPECIES: DUF3577 domain-containing protein [Pseudomonadota]
fig|80869.170.peg.2260	T3S	acyl-CoA thioesterase [*Paracidovorax citrulli*]
fig|80869.170.peg.2609	T3S	STY0301 family protein [*Paracidovorax citrulli*]
fig|80869.170.peg.3212	T3S	ABC transporter ATP-binding protein [*Paracidovorax citrulli*]
fig|80869.170.peg.2723	T3S	Ku protein [*Paracidovorax citrulli*]
fig|80869.170.peg.1815	T3S	terminase small subunit [*Paracidovorax citrulli*]
fig|80869.170.peg.1029	T3S	NCS1 family nucleobase:cation symporter-1 [*Paracidovorax citrulli*]
fig|80869.170.peg.1032	T3S	peptidoglycan-binding domain-containing protein [*Paracidovorax citrulli*]
fig|80869.170.peg.1056	T3S	penicillin-binding protein 1A [*Paracidovorax citrulli*]
fig|80869.170.peg.1066	T3S	protein of unknown function DUF1415 [*Paracidovorax citrulli* AAC00-1]
fig|80869.170.peg.110	T3S	hotdog fold thioesterase [*Paracidovorax citrulli*]
fig|80869.170.peg.1103	T3S	PelD GGDEF domain-containing protein [*Paracidovorax citrulli*]
fig|80869.170.peg.114	T3S	hypothetical protein [*Paracidovorax citrulli*]
fig|80869.170.peg.1147	T3S	DMT family transporter [*Paracidovorax citrulli*]
fig|80869.170.peg.1166	T3S	GntR family transcriptional regulator [*Paracidovorax citrulli*]
fig|80869.170.peg.1168	T3S	NCS1 family nucleobase:cation symporter-1 [*Paracidovorax citrulli*]
fig|80869.170.peg.1184	T3S	glycerate kinase [*Paracidovorax citrulli*]
fig|80869.170.peg.122	T3S	MBL fold metallo-hydrolase [*Paracidovorax citrulli*]
fig|80869.170.peg.1222	T3S	neutral zinc metallopeptidase [*Paracidovorax citrulli*]
fig|80869.170.peg.1231	T3S	tryptophan 2,3-dioxygenase [*Paracidovorax citrulli*]
fig|80869.170.peg.126	T3S	5-demethoxyubiquinol-8 5-hydroxylase UbiM [*Paracidovorax citrulli*]
fig|80869.170.peg.1283	T3S	Phytochrome-like protein cph2 [*Paracidovorax citrulli*]
fig|80869.170.peg.1284	T3S	lysine--tRNA ligase [*Paracidovorax citrulli*]
fig|80869.170.peg.1291	T3S	LON peptidase substrate-binding domain-containing protein [*Paracidovorax citrulli*]
fig|80869.170.peg.1309	T3S	nucleotide exchange factor GrpE [*Paracidovorax citrulli*]
fig|80869.170.peg.1324	T3S	molybdopterin converting factor subunit 1 [*Paracidovorax citrulli*]
fig|80869.170.peg.1337	T3S	hypothetical protein [*Paracidovorax citrulli*]
fig|80869.170.peg.1357	T3S	MULTISPECIES: NADH-quinone oxidoreductase subunit NuoI [Comamonadaceae]
fig|80869.170.peg.1365	T3S	ABC transporter transmembrane domain-containing protein [*Paracidovorax citrulli*]
fig|80869.170.peg.1383	T3S	winged helix-turn-helix transcriptional regulator [*Paracidovorax citrulli*]
fig|80869.170.peg.1404	T3S	diguanylate phosphodiesterase [*Paracidovorax citrulli* AAC00-1]
fig|80869.170.peg.1408	T3S	hypothetical protein [*Paracidovorax citrulli*]
fig|80869.170.peg.1434	T3S	pyridoxal kinase PdxY [*Paracidovorax citrulli*]
fig|80869.170.peg.1440	T3S	DNA internalization-related competence protein ComEC/Rec2 [*Paracidovorax citrulli*]
fig|80869.170.peg.1448	T3S	uncharacterized protein DUF2132 [*Paracidovorax citrulli*]

Prot	Pred	Description
fig|80869.170.peg.1481	T3S	CaiB/BaiF CoA transferase family protein [*Paracidovorax citrulli*]
fig|80869.170.peg.1509	T3S	protein-L-isoaspartate(D-aspartate) O-methyltransferase [*Paracidovorax citrulli*]
fig|80869.170.peg.1524	T3S	GTPase HflX [*Paracidovorax citrulli*]
fig|80869.170.peg.1549	T3S	DUF2069 domain-containing protein [*Paracidovorax citrulli*]
fig|80869.170.peg.1552	T3S	farnesyl-diphosphate farnesyltransferase [*Paracidovorax citrulli* AAC00-1]
fig|80869.170.peg.1593	T3S	glycine betaine/L-proline ABC transporter permease ProW [*Paracidovorax citrulli*]
fig|80869.170.peg.1613	T3S	malonate--CoA ligase [*Paracidovorax citrulli*]
fig|80869.170.peg.1620	T3S	cation-translocating P-type ATPase [*Paracidovorax citrulli*]
fig|80869.170.peg.1650	T3S	SDR family NAD(P)-dependent oxidoreductase [*Paracidovorax citrulli*]
fig|80869.170.peg.1699	T3S	type 1 glutamine amidotransferase domain-containing protein [*Paracidovorax citrulli*]
fig|80869.170.peg.1701	T3S	polyhydroxyalkanoate synthesis repressor PhaR [*Paracidovorax citrulli*]
fig|80869.170.peg.1711	T3S	hypothetical protein Aave_1606 [*Paracidovorax citrulli* AAC00-1]
fig|80869.170.peg.1741	T3S	hypothetical protein [*Paracidovorax citrulli*]
fig|80869.170.peg.1756	T3S	MHS family citrate/tricarballylate:H+ symporter-like MFS transporter [*Paracidovorax citrulli*]
fig|80869.170.peg.1799	T3S	hypothetical protein Aave_1685 [*Paracidovorax citrulli* AAC00-1]
fig|80869.170.peg.180	T3S	aromatic ring-hydroxylating dioxygenase subunit alpha [*Paracidovorax citrulli*]
fig|80869.170.peg.1812	T3S	hypothetical protein [*Paracidovorax citrulli*]
fig|80869.170.peg.1919	T3S	hypothetical protein [*Paracidovorax citrulli*]
fig|80869.170.peg.192	T3S	Cupin 2, conserved barrel domain protein [*Paracidovorax citrulli* AAC00-1]
fig|80869.170.peg.1922	T3S	structural protein P5 [*Paracidovorax citrulli*]
fig|80869.170.peg.1937	T3S	ATP synthase F1 subunit epsilon [*Paracidovorax citrulli*]
fig|80869.170.peg.1942	T3S	P1 family peptidase [*Paracidovorax citrulli*]
fig|80869.170.peg.1954	T3S	YitT family protein [*Paracidovorax citrulli*]
fig|80869.170.peg.1958	T3S	MlaD family protein [*Paracidovorax citrulli*]
fig|80869.170.peg.1973	T3S	UDP-3-O-(3-hydroxymyristoyl)glucosamine N-acyltransferase [*Paracidovorax citrulli*]
fig|80869.170.peg.2006	T3S	glutamate--tRNA ligase [*Paracidovorax citrulli*]
fig|80869.170.peg.2007	T3S	DUF1624 domain-containing protein [*Paracidovorax citrulli*]
fig|80869.170.peg.2010	T3S	DUF72 domain-containing protein [*Paracidovorax citrulli*]
fig|80869.170.peg.2093	T3S	argininosuccinate lyase [*Paracidovorax citrulli*]
fig|80869.170.peg.2124	T3S	CDP-6-deoxy-delta-3,4-glucoseen reductase [*Paracidovorax citrulli*]
fig|80869.170.peg.213	T3S	transcriptional regulator CynR [*Paracidovorax citrulli*]
fig|80869.170.peg.2133	T3S	hypothetical protein [*Paracidovorax citrulli*]
fig|80869.170.peg.2146	T3S	ABC-F family ATP-binding cassette domain-containing protein [*Paracidovorax citrulli*]
fig|80869.170.peg.2148	T3S	uroporphyrinogen-III C-methyltransferase [*Paracidovorax citrulli*]
fig|80869.170.peg.2150	T3S	hydroxymethylbilane synthase [*Paracidovorax citrulli*]
fig|80869.170.peg.2151	T3S	phosphoenolpyruvate carboxylase [*Paracidovorax citrulli*]
fig|80869.170.peg.2152	T3S	YccS family putative transporter [*Paracidovorax citrulli*]
fig|80869.170.peg.2154	T3S	AI-2E family transporter [*Paracidovorax citrulli*]
fig|80869.170.peg.2160	T3S	SfnB family sulfur acquisition oxidoreductase [*Paracidovorax citrulli*]
fig|80869.170.peg.2161	T3S	SfnB family sulfur acquisition oxidoreductase [*Paracidovorax citrulli*]
fig|80869.170.peg.2166	T3S	exodeoxyribonuclease VII small subunit [*Paracidovorax citrulli*]
fig|80869.170.peg.2177	T3S	alpha-1,4-glucan--maltose-1-phosphate maltosyltransferase [*Paracidovorax citrulli*]
fig|80869.170.peg.2180	T3S	BCCT family transporter [*Paracidovorax citrulli*]

Prot	Pred	Description
fig|80869.170.peg.2185	T3S	TOBE domain-containing protein [*Paracidovorax citrulli*]
fig|80869.170.peg.2196	T3S	efflux transporter outer membrane subunit [*Paracidovorax citrulli*]
fig|80869.170.peg.2239	T3S	succinate-semialdehyde dehydrogenase/glutarate-semialdehyde dehydrogenase [*Paracidovorax citrulli*]
fig|80869.170.peg.2262	T3S	uroporphyrinogen-III C-methyltransferase [*Paracidovorax citrulli*]
fig|80869.170.peg.2279	T3S	carboxyl transferase domain-containing protein [*Paracidovorax citrulli*]
fig|80869.170.peg.2287	T3S	excinuclease ABC subunit UvrA [*Paracidovorax citrulli*]
fig|80869.170.peg.2292	T3S	DUF2169 domain-containing protein [*Paracidovorax citrulli*]
fig|80869.170.peg.2313	T3S	hypothetical protein Aave_2148 [*Paracidovorax citrulli* AAC00-1]
fig|80869.170.peg.2340	T3S	hypothetical protein [*Paracidovorax citrulli*]
fig|80869.170.peg.2357	T3S	NAD-dependent succinate-semialdehyde dehydrogenase [*Paracidovorax citrulli*]
fig|80869.170.peg.2372	T3S	transcriptional regulator, GntR family [*Paracidovorax citrulli* AAC00-1]
fig|80869.170.peg.2387	T3S	cytochrome o ubiquinol oxidase subunit IV [*Paracidovorax citrulli*]
fig|80869.170.peg.2420	T3S	multiple monosaccharide ABC transporter permease [*Paracidovorax citrulli*]
fig|80869.170.peg.2426	T3S	SDR family NAD(P)-dependent oxidoreductase [*Paracidovorax citrulli*]
fig|80869.170.peg.2427	T3S	SMP-30/gluconolactonase/LRE family protein [*Paracidovorax citrulli*]
fig|80869.170.peg.2435	T3S	GAF domain-containing sensor histidine kinase [*Paracidovorax citrulli*]
fig|80869.170.peg.2442	T3S	MFS transporter [*Paracidovorax citrulli*]
fig|80869.170.peg.2444	T3S	redox-sensitive transcriptional activator SoxR [*Paracidovorax citrulli*]
fig|80869.170.peg.2448	T3S	DHH family phosphoesterase [*Paracidovorax citrulli*]
fig|80869.170.peg.245	T3S	GNAT family N-acetyltransferase [Paracidovorax avenae]
fig|80869.170.peg.2458	T3S	Murein DD-endopeptidase MepM [*Paracidovorax citrulli*]
fig|80869.170.peg.2461	T3S	esterase/lipase/thioesterase family protein [*Paracidovorax citrulli* AAC00-1]
fig|80869.170.peg.2473	T3S	glutamine--tRNA ligase/YqeY domain fusion protein [*Paracidovorax citrulli*]
fig|80869.170.peg.2478	T3S	DODA-type extradiol aromatic ring-opening family dioxygenase [*Paracidovorax citrulli*]
fig|80869.170.peg.2585	T3S	hypothetical protein [*Paracidovorax citrulli*]
fig|80869.170.peg.2602	T3S	KGG domain-containing protein [*Paracidovorax citrulli*]
fig|80869.170.peg.2649	T3S	PAS domain-containing sensor histidine kinase [*Paracidovorax citrulli*]
fig|80869.170.peg.2683	T3S	EAL domain-containing protein [*Paracidovorax citrulli*]
fig|80869.170.peg.2764	T3S	ATP-dependent RNA helicase HrpA [*Paracidovorax citrulli*]
fig|80869.170.peg.2807	T3S	TonB-dependent receptor family protein [*Paracidovorax citrulli*]
fig|80869.170.peg.2818	T3S	D-serine/D-alanine/glycine transporter [*Paracidovorax citrulli*]
fig|80869.170.peg.2819	T3S	hypothetical protein [*Paracidovorax citrulli*]
fig|80869.170.peg.2832	T3S	Polyphosphate kinase [*Paracidovorax citrulli* AAC00-1]
fig|80869.170.peg.2837	T3S	phosphate ABC transporter permease PstC [*Paracidovorax citrulli*]
fig|80869.170.peg.2866	T3S	gephyrin-like molybdotransferase Glp [*Paracidovorax citrulli*]
fig|80869.170.peg.2869	T3S	GTP cyclohydrolase subunit MoaA [*Paracidovorax citrulli* AAC00-1]
fig|80869.170.peg.2880	T3S	translesion DNA synthesis-associated protein ImuA [*Paracidovorax citrulli*]
fig|80869.170.peg.2884	T3S	pseudouridine synthase [*Paracidovorax citrulli*]
fig|80869.170.peg.2905	T3S	hypothetical protein CQB05_01060 [*Paracidovorax citrulli*]
fig|80869.170.peg.2915	T3S	DEAD/DEAH box helicase [*Paracidovorax citrulli*]
fig|80869.170.peg.2957	T3S	hypothetical protein [*Paracidovorax citrulli*]
fig|80869.170.peg.298	T3S	AmpG family muropeptide MFS transporter [*Paracidovorax citrulli*]
fig|80869.170.peg.3	T3S	DNA topoisomerase (ATP-hydrolyzing) subunit B [*Paracidovorax citrulli*]
fig|80869.170.peg.301	T3S	DME family drug/metabolite transporter [*Paracidovorax citrulli*]
fig|80869.170.peg.303	T3S	SLC13 family permease [*Paracidovorax citrulli*]

Prot	Pred	Description
fig|80869.170.peg.3030	T3S	pyridoxamine 5'-phosphate oxidase [*Paracidovorax citrulli*]
fig|80869.170.peg.3041	T3S	transcriptional repressor [*Paracidovorax citrulli*]
fig|80869.170.peg.3043	T3S	TetR/AcrR family transcriptional regulator [*Paracidovorax citrulli*]
fig|80869.170.peg.3062	T3S	EVE domain-containing protein [*Paracidovorax citrulli*]
fig|80869.170.peg.3073	T3S	XopAP family type III secretion system effector [*Paracidovorax citrulli*]
fig|80869.170.peg.3166	T3S	glycoside hydrolase family 5 protein [*Paracidovorax citrulli*]
fig|80869.170.peg.3192	T3S	protein of unknown function UPF0061 [*Paracidovorax citrulli* AAC00-1]
fig|80869.170.peg.322	T3S	amino acid ABC transporter ATP-binding protein [*Paracidovorax citrulli*]
fig|80869.170.peg.3271	T3S	BON domain-containing protein [*Paracidovorax citrulli*]
fig|80869.170.peg.3272	T3S	SulP family inorganic anion transporter [*Paracidovorax citrulli*]
fig|80869.170.peg.3280	T3S	NAD(P)/FAD-dependent oxidoreductase [*Paracidovorax citrulli*]
fig|80869.170.peg.3282	T3S	tRNA (guanosine(46)-N7)-methyltransferase TrmB [*Paracidovorax citrulli*]
fig|80869.170.peg.3342	T3S	hypothetical protein [*Paracidovorax citrulli*]
fig|80869.170.peg.3349	T3S	ATP-binding protein [*Paracidovorax citrulli*]
fig|80869.170.peg.3359	T3S	YbaN family protein [*Paracidovorax citrulli*]
fig|80869.170.peg.3373	T3S	RDD domain containing protein [*Paracidovorax citrulli* AAC00-1]
fig|80869.170.peg.3392	T3S	TetR/AcrR family transcriptional regulator [*Paracidovorax citrulli*]
fig|80869.170.peg.3415	T3S	2OG-Fe dioxygenase family protein [*Paracidovorax citrulli*]
fig|80869.170.peg.3426	T3S	MULTISPECIES: CysB family HTH-type transcriptional regulator [Comamonadaceae]
fig|80869.170.peg.346	T3S	DHA2 family efflux MFS transporter permease subunit [*Paracidovorax citrulli*]
fig|80869.170.peg.3484	T3S	DUF4230 domain-containing protein [*Paracidovorax citrulli*]
fig|80869.170.peg.3519	T3S	ABC transporter permease [*Paracidovorax citrulli*]
fig|80869.170.peg.3589	T3S	hypothetical protein [*Paracidovorax citrulli*]
fig|80869.170.peg.3591	T3S	L-proline dehydrogenase /delta-1-pyrroline-5-carboxylate dehydrogenase [*Paracidovorax citrulli*]
fig|80869.170.peg.3635	T3S	helix-turn-helix transcriptional regulator [*Paracidovorax citrulli*]
fig|80869.170.peg.3660	T3S	thioredoxin family protein [*Paracidovorax citrulli*]
fig|80869.170.peg.3662	T3S	cytochrome c553-like protein [*Paracidovorax citrulli* AAC00-1]
fig|80869.170.peg.3678	T3S	ThiF family adenylyltransferase [*Paracidovorax citrulli*]
fig|80869.170.peg.3682	T3S	lipid A export permease/ATP-binding protein MsbA [*Paracidovorax citrulli*]
fig|80869.170.peg.3706	T3S	hypothetical protein [*Paracidovorax citrulli*]
fig|80869.170.peg.3710	T3S	protein of unknown function DUF1653 [*Paracidovorax citrulli* AAC00-1]
fig|80869.170.peg.3725	T3S	isoleucine--tRNA ligase [*Paracidovorax citrulli*]
fig|80869.170.peg.3727	T3S	hypothetical protein Aave_3452 [*Paracidovorax citrulli* AAC00-1]
fig|80869.170.peg.3771	T3S	PilZ domain-containing protein [*Paracidovorax citrulli*]
fig|80869.170.peg.1062	T4S	Ig domain protein, group 1 domain protein [*Paracidovorax citrulli* AAC00-1]
fig|80869.170.peg.2342	T4S	hypothetical protein [*Paracidovorax citrulli*]
fig|80869.170.peg.2454	T4S	major facilitator superfamily MFS_1 [*Paracidovorax citrulli* AAC00-1]
fig|80869.170.peg.584	T4S	MULTISPECIES: DUF3577 domain-containing protein [Pseudomonadota]
fig|80869.170.peg.2260	T4S	acyl-CoA thioesterase [*Paracidovorax citrulli*]
fig|80869.170.peg.2609	T4S	STY0301 family protein [*Paracidovorax citrulli*]
fig|80869.170.peg.3212	T4S	ABC transporter ATP-binding protein [*Paracidovorax citrulli*]
fig|80869.170.peg.2723	T4S	Ku protein [*Paracidovorax citrulli*]
fig|80869.170.peg.1815	T4S	terminase small subunit [*Paracidovorax citrulli*]
fig|80869.170.peg.1331	T4S	molybdenum cofactor biosynthesis protein MoaE [*Paracidovorax citrulli*]
fig|80869.170.peg.4172	T4S	bacterioferritin [*Paracidovorax citrulli*]

Prot	Pred	Description
fig|80869.170.peg.1766	T4S	isoaspartyl peptidase/L-asparaginase family protein [*Paracidovorax citrulli*]
fig|80869.170.peg.4071	T4S	abortive infection system antitoxin AbiGi family protein [*Paracidovorax citrulli*]
fig|80869.170.peg.2305	T4S	RNA recognition motif domain-containing protein [*Paracidovorax citrulli*]
fig|80869.170.peg.2906	T4S	hypothetical protein [*Paracidovorax citrulli*]
fig|80869.170.peg.3326	T4S	hypothetical protein Aave_3072 [*Paracidovorax citrulli* AAC00-1]
fig|80869.170.peg.3068	T4S	DUF1795 domain-containing protein [*Paracidovorax citrulli*]
fig|80869.170.peg.506	T4S	type III secretion system outer membrane ring subunit SctC [*Paracidovorax citrulli*]
fig|80869.170.peg.2320	T4S	hypothetical protein [*Paracidovorax citrulli*]
fig|80869.170.peg.2760	T4S	MULTISPECIES: amino-acid N-acetyltransferase [Paracidovorax]
fig|80869.170.peg.4953	T4S	DUF924 family protein [*Paracidovorax citrulli*]
fig|80869.170.peg.4007	T4S	hypothetical protein [*Paracidovorax citrulli*]
fig|80869.170.peg.4891	T4S	50S ribosomal protein L11 [*Paracidovorax citrulli*]
fig|80869.170.peg.2726	T4S	hypothetical protein [*Paracidovorax citrulli*]
fig|80869.170.peg.2428	T4S	conserved hypothetical protein [*Paracidovorax citrulli* AAC00-1]
fig|80869.170.peg.3857	T4S	PP2C family protein-serine/threonine phosphatase [*Paracidovorax citrulli*]
fig|80869.170.peg.4225	T4S	glutathione-regulated potassium-efflux system protein KefC [*Paracidovorax citrulli*]
fig|80869.170.peg.94	T4S	hypothetical protein [*Paracidovorax citrulli*]
fig|80869.170.peg.4871	T4S	cryptochrome/photolyase family protein [*Paracidovorax citrulli*]
fig|80869.170.peg.1410	T4S	phosphopyruvate hydratase [*Paracidovorax citrulli*]
fig|80869.170.peg.3800	T4S	hypothetical protein [*Paracidovorax citrulli*]
fig|80869.170.peg.3529	T4S	hypothetical protein [*Paracidovorax citrulli*]
fig|80869.170.peg.1986	T4S	AAA family ATPase [*Paracidovorax citrulli*]
fig|80869.170.peg.4656	T4S	DUF3025 domain-containing protein [*Paracidovorax citrulli*]
fig|80869.170.peg.3858	T4S	serine/threonine protein kinase [*Paracidovorax citrulli*]
fig|80869.170.peg.2227	T4S	inositol monophosphatase family protein [*Paracidovorax citrulli*]
fig|80869.170.peg.4582	T4S	N-acetylmuramoyl-L-alanine amidase [*Paracidovorax citrulli*]
fig|80869.170.peg.635	T4S	AraC family transcriptional regulator [*Paracidovorax citrulli*]
fig|80869.170.peg.1512	T4S	SMC-Scp complex subunit ScpB [*Paracidovorax citrulli*]
fig|80869.170.peg.3587	T4S	hypothetical protein [*Paracidovorax citrulli*]
fig|80869.170.peg.4989	T4S	Fic family protein [*Paracidovorax citrulli*]
fig|80869.170.peg.4078	T4S	4-hydroxy-3-methylbut-2-enyl diphosphate reductase [*Paracidovorax citrulli*]
fig|80869.170.peg.3647	T4S	SsrA-binding protein SmpB [*Paracidovorax citrulli*]
fig|80869.170.peg.3149	T4S	hypothetical protein [*Paracidovorax citrulli*]
fig|80869.170.peg.397	T4S	F0F1 ATP synthase subunit gamma [*Paracidovorax citrulli*]
fig|80869.170.peg.2073	T4S	hypothetical protein [*Paracidovorax citrulli*]
fig|80869.170.peg.1539	T4S	hypothetical protein [*Paracidovorax citrulli*]
fig|80869.170.peg.2860	T4S	transglycosylase SLT domain-containing protein [*Paracidovorax citrulli*]
fig|80869.170.peg.4446	T4S	hypothetical protein [*Paracidovorax citrulli*]
fig|80869.170.peg.1833	T4S	hypothetical protein [*Paracidovorax citrulli*]
fig|80869.170.peg.4288	T4S	5'-nucleotidase [*Paracidovorax citrulli*]
fig|80869.170.peg.2544	T4S	hypothetical protein [*Paracidovorax citrulli*]
fig|80869.170.peg.670	T4S	30S ribosomal protein S4 [*Paracidovorax citrulli*]
fig|80869.170.peg.2769	T4S	adenylate kinase [*Paracidovorax citrulli*]
fig|80869.170.peg.3510	T4S	hypothetical protein [*Paracidovorax citrulli*]
fig|80869.170.peg.3827	T4S	type IV pilin protein [*Paracidovorax citrulli*]

Prot	Pred	Description
fig|80869.170.peg.4676	T4S	hypothetical protein [*Paracidovorax citrulli*]
fig|80869.170.peg.4173	T4S	BON domain-containing protein [*Paracidovorax citrulli*]
fig|80869.170.peg.2745	T4S	MetQ/NlpA family ABC transporter substrate-binding protein [*Paracidovorax citrulli*]
fig|80869.170.peg.4818	T4S	Bug family tripartite tricarboxylate transporter substrate binding protein [*Paracidovorax citrulli*]
fig|80869.170.peg.2941	T4S	hypothetical protein [*Paracidovorax citrulli*]
fig|80869.170.peg.2752	T4S	sulfate ABC transporter substrate-binding protein [*Paracidovorax citrulli*]
fig|80869.170.peg.4053	T4S	hypothetical protein [*Paracidovorax citrulli*]
fig|80869.170.peg.4952	T4S	OmpA family protein [*Paracidovorax citrulli*]
fig|80869.170.peg.1851	T4S	glycoside hydrolase family protein [*Paracidovorax citrulli*]
fig|80869.170.peg.326	T4S	DNA-binding transcriptional LysR family regulator [*Paracidovorax citrulli*]
fig|80869.170.peg.2043	T4S	sigma-54-dependent transcriptional regulator [*Paracidovorax citrulli*]
fig|80869.170.peg.4289	T4S	EF-hand domain-containing protein [*Paracidovorax citrulli*]

*P_citrulli*_AAC00-1_5593

Prot	Pred	Description
fig|80869.172.peg.1964	T3S	major facilitator superfamily MFS_1 [*Paracidovorax citrulli* AAC00-1]
fig|80869.172.peg.2076	T3S	hypothetical protein [*Paracidovorax citrulli*]
fig|80869.172.peg.3351	T3S	Ig domain protein, group 1 domain protein [*Paracidovorax citrulli* AAC00-1]
fig|80869.172.peg.3830	T3S	MULTISPECIES: DUF3577 domain-containing protein [Pseudomonadota]
fig|80869.172.peg.2160	T3S	acyl-CoA thioesterase [*Paracidovorax citrulli*]
fig|80869.172.peg.1808	T3S	STY0301 family protein [*Paracidovorax citrulli*]
fig|80869.172.peg.1209	T3S	ABC transporter ATP-binding protein [*Paracidovorax citrulli*]
fig|80869.172.peg.1694	T3S	Ku protein [*Paracidovorax citrulli*]
fig|80869.172.peg.2599	T3S	terminase small subunit [*Paracidovorax citrulli*]
fig|80869.172.peg.1006	T3S	2OG-Fe dioxygenase family protein [*Paracidovorax citrulli*]
fig|80869.172.peg.1029	T3S	TetR/AcrR family transcriptional regulator [*Paracidovorax citrulli*]
fig|80869.172.peg.1048	T3S	RDD domain containing protein [*Paracidovorax citrulli* AAC00-1]
fig|80869.172.peg.106	T3S	ABC transporter permease [*Paracidovorax citrulli*]
fig|80869.172.peg.1062	T3S	YbaN family protein [*Paracidovorax citrulli*]
fig|80869.172.peg.107	T3S	ABC transporter permease [*Paracidovorax citrulli*]
fig|80869.172.peg.1072	T3S	ATP-binding protein [*Paracidovorax citrulli*]
fig|80869.172.peg.1079	T3S	hypothetical protein [*Paracidovorax citrulli*]
fig|80869.172.peg.1139	T3S	tRNA (guanosine(46)-N7)-methyltransferase TrmB [*Paracidovorax citrulli*]
fig|80869.172.peg.1141	T3S	NAD(P)/FAD-dependent oxidoreductase [*Paracidovorax citrulli*]
fig|80869.172.peg.1149	T3S	SulP family inorganic anion transporter [*Paracidovorax citrulli*]
fig|80869.172.peg.1150	T3S	BON domain-containing protein [*Paracidovorax citrulli*]
fig|80869.172.peg.121	T3S	hypothetical protein [*Paracidovorax citrulli*]
fig|80869.172.peg.1229	T3S	protein of unknown function UPF0061 [*Paracidovorax citrulli* AAC00-1]
fig|80869.172.peg.1274	T3S	hypothetical protein [*Paracidovorax citrulli*]
fig|80869.172.peg.1346	T3S	pyridoxamine 5'-phosphate oxidase [*Paracidovorax citrulli*]
fig|80869.172.peg.1357	T3S	transcriptional repressor [*Paracidovorax citrulli*]
fig|80869.172.peg.1359	T3S	TetR/AcrR family transcriptional regulator [*Paracidovorax citrulli*]
fig|80869.172.peg.137	T3S	luciferase family protein [*Paracidovorax citrulli* AAC00-1]
fig|80869.172.peg.1378	T3S	EVE domain-containing protein [*Paracidovorax citrulli*]
fig|80869.172.peg.1391	T3S	XopAP family type III secretion system effector [*Paracidovorax citrulli*]

Prot	Pred	Description
fig|80869.172.peg.1482	T3S	glycoside hydrolase family 5 protein [*Paracidovorax citrulli*]
fig|80869.172.peg.1505	T3S	DEAD/DEAH box helicase [*Paracidovorax citrulli*]
fig|80869.172.peg.1515	T3S	hypothetical protein CQB05_01060 [*Paracidovorax citrulli*]
fig|80869.172.peg.1536	T3S	pseudouridine synthase [*Paracidovorax citrulli*]
fig|80869.172.peg.1540	T3S	translesion DNA synthesis-associated protein ImuA [*Paracidovorax citrulli*]
fig|80869.172.peg.1551	T3S	GTP cyclohydrolase subunit MoaA [*Paracidovorax citrulli* AAC00-1]
fig|80869.172.peg.1554	T3S	gephyrin-like molybdotransferase Glp [*Paracidovorax citrulli*]
fig|80869.172.peg.1583	T3S	phosphate ABC transporter permease PstC [*Paracidovorax citrulli*]
fig|80869.172.peg.1588	T3S	Polyphosphate kinase [*Paracidovorax citrulli* AAC00-1]
fig|80869.172.peg.1601	T3S	hypothetical protein [*Paracidovorax citrulli*]
fig|80869.172.peg.1602	T3S	D-serine/D-alanine/glycine transporter [*Paracidovorax citrulli*]
fig|80869.172.peg.1612	T3S	TonB-dependent receptor family protein [*Paracidovorax citrulli*]
fig|80869.172.peg.164	T3S	hypothetical protein [*Paracidovorax citrulli*]
fig|80869.172.peg.165	T3S	hypothetical protein C8E08_1237 [*Paracidovorax citrulli*]
fig|80869.172.peg.1705	T3S	GCN5-related N-acetyltransferase [*Paracidovorax citrulli* AAC00-1]
fig|80869.172.peg.1733	T3S	EAL domain-containing protein [*Paracidovorax citrulli*]
fig|80869.172.peg.1768	T3S	PAS domain-containing sensor histidine kinase [*Paracidovorax citrulli*]
fig|80869.172.peg.1816	T3S	KGG domain-containing protein [*Paracidovorax citrulli*]
fig|80869.172.peg.1833	T3S	hypothetical protein [*Paracidovorax citrulli*]
fig|80869.172.peg.1940	T3S	DODA-type extradiol aromatic ring-opening family dioxygenase [*Paracidovorax citrulli*]
fig|80869.172.peg.1945	T3S	glutamine--tRNA ligase/YqeY domain fusion protein [*Paracidovorax citrulli*]
fig|80869.172.peg.1957	T3S	esterase/lipase/thioesterase family protein [*Paracidovorax citrulli* AAC00-1]
fig|80869.172.peg.1960	T3S	Murein DD-endopeptidase MepM [*Paracidovorax citrulli*]
fig|80869.172.peg.1970	T3S	DHH family phosphoesterase [*Paracidovorax citrulli*]
fig|80869.172.peg.1974	T3S	redox-sensitive transcriptional activator SoxR [*Paracidovorax citrulli*]
fig|80869.172.peg.1976	T3S	MFS transporter [*Paracidovorax citrulli*]
fig|80869.172.peg.1983	T3S	GAF domain-containing sensor histidine kinase [*Paracidovorax citrulli*]
fig|80869.172.peg.1990	T3S	SMP-30/gluconolactonase/LRE family protein [*Paracidovorax citrulli*]
fig|80869.172.peg.1991	T3S	SDR family NAD(P)-dependent oxidoreductase [*Paracidovorax citrulli*]
fig|80869.172.peg.1997	T3S	multiple monosaccharide ABC transporter permease [*Paracidovorax citrulli*]
fig|80869.172.peg.2031	T3S	cytochrome o ubiquinol oxidase subunit IV [*Paracidovorax citrulli*]
fig|80869.172.peg.2046	T3S	transcriptional regulator, GntR family [*Paracidovorax citrulli* AAC00-1]
fig|80869.172.peg.2061	T3S	NAD-dependent succinate-semialdehyde dehydrogenase [*Paracidovorax citrulli*]
fig|80869.172.peg.2078	T3S	hypothetical protein [*Paracidovorax citrulli*]
fig|80869.172.peg.2107	T3S	hypothetical protein Aave_2148 [*Paracidovorax citrulli* AAC00-1]
fig|80869.172.peg.2128	T3S	DUF2169 domain-containing protein [*Paracidovorax citrulli*]
fig|80869.172.peg.2133	T3S	excinuclease ABC subunit UvrA [*Paracidovorax citrulli*]
fig|80869.172.peg.2141	T3S	carboxyl transferase domain-containing protein [*Paracidovorax citrulli*]
fig|80869.172.peg.2158	T3S	uroporphyrinogen-III C-methyltransferase [*Paracidovorax citrulli*]
fig|80869.172.peg.2181	T3S	succinate-semialdehyde dehydrogenase/glutarate-semialdehyde dehydrogenase [*Paracidovorax citrulli*]
fig|80869.172.peg.2224	T3S	efflux transporter outer membrane subunit [*Paracidovorax citrulli*]
fig|80869.172.peg.2235	T3S	TOBE domain-containing protein [*Paracidovorax citrulli*]
fig|80869.172.peg.2240	T3S	BCCT family transporter [*Paracidovorax citrulli*]
fig|80869.172.peg.2243	T3S	alpha-1,4-glucan--maltose-1-phosphate maltosyltransferase [*Paracidovorax citrulli*]
fig|80869.172.peg.2253	T3S	exodeoxyribonuclease VII small subunit [*Paracidovorax citrulli*]

Prot	Pred	Description
fig|80869.172.peg.2258	T3S	SfnB family sulfur acquisition oxidoreductase [*Paracidovorax citrulli*]
fig|80869.172.peg.2259	T3S	SfnB family sulfur acquisition oxidoreductase [*Paracidovorax citrulli*]
fig|80869.172.peg.2265	T3S	AI-2E family transporter [*Paracidovorax citrulli*]
fig|80869.172.peg.2267	T3S	phosphoenolpyruvate carboxylase [*Paracidovorax citrulli*]
fig|80869.172.peg.2268	T3S	hydroxymethylbilane synthase [*Paracidovorax citrulli*]
fig|80869.172.peg.2270	T3S	uroporphyrinogen-III C-methyltransferase [*Paracidovorax citrulli*]
fig|80869.172.peg.2272	T3S	ABC-F family ATP-binding cassette domain-containing protein [*Paracidovorax citrulli*]
fig|80869.172.peg.2285	T3S	hypothetical protein [*Paracidovorax citrulli*]
fig|80869.172.peg.2294	T3S	CDP-6-deoxy-delta-3,4-glucoseen reductase [*Paracidovorax citrulli*]
fig|80869.172.peg.232	T3S	MATE family efflux transporter [*Paracidovorax citrulli*]
fig|80869.172.peg.2322	T3S	argininosuccinate lyase [*Paracidovorax citrulli*]
fig|80869.172.peg.2406	T3S	DUF72 domain-containing protein [*Paracidovorax citrulli*]
fig|80869.172.peg.2409	T3S	DUF1624 domain-containing protein [*Paracidovorax citrulli*]
fig|80869.172.peg.2410	T3S	glutamate--tRNA ligase [*Paracidovorax citrulli*]
fig|80869.172.peg.243	T3S	signal recognition particle-docking protein FtsY [*Paracidovorax citrulli*]
fig|80869.172.peg.2443	T3S	UDP-3-O-(3-hydroxymyristoyl)glucosamine N-acyltransferase [*Paracidovorax citrulli*]
fig|80869.172.peg.2458	T3S	MlaD family protein [*Paracidovorax citrulli*]
fig|80869.172.peg.2462	T3S	YitT family protein [*Paracidovorax citrulli*]
fig|80869.172.peg.2473	T3S	P1 family peptidase [*Paracidovorax citrulli*]
fig|80869.172.peg.2478	T3S	ATP synthase F1 subunit epsilon [*Paracidovorax citrulli*]
fig|80869.172.peg.2492	T3S	structural protein P5 [*Paracidovorax citrulli*]
fig|80869.172.peg.2495	T3S	hypothetical protein [*Paracidovorax citrulli*]
fig|80869.172.peg.2602	T3S	hypothetical protein [*Paracidovorax citrulli*]
fig|80869.172.peg.2615	T3S	hypothetical protein Aave_1685 [*Paracidovorax citrulli* AAC00-1]
fig|80869.172.peg.264	T3S	3-methyl-2-oxobutanoate hydroxymethyltransferase [*Paracidovorax citrulli*]
fig|80869.172.peg.2673	T3S	hypothetical protein [*Paracidovorax citrulli*]
fig|80869.172.peg.2703	T3S	hypothetical protein Aave_1606 [*Paracidovorax citrulli* AAC00-1]
fig|80869.172.peg.2713	T3S	polyhydroxyalkanoate synthesis repressor PhaR [*Paracidovorax citrulli*]
fig|80869.172.peg.2715	T3S	type 1 glutamine amidotransferase domain-containing protein [*Paracidovorax citrulli*]
fig|80869.172.peg.2763	T3S	SDR family NAD(P)-dependent oxidoreductase [*Paracidovorax citrulli*]
fig|80869.172.peg.279	T3S	hypothetical protein [*Paracidovorax citrulli*]
fig|80869.172.peg.2793	T3S	heavy metal translocating P-type ATPase [*Paracidovorax citrulli*]
fig|80869.172.peg.280	T3S	YitT family protein [*Paracidovorax citrulli*]
fig|80869.172.peg.2801	T3S	malonate--CoA ligase [*Paracidovorax citrulli*]
fig|80869.172.peg.2821	T3S	glycine betaine/L-proline ABC transporter permease ProW [*Paracidovorax citrulli*]
fig|80869.172.peg.2863	T3S	farnesyl-diphosphate farnesyltransferase [*Paracidovorax citrulli* AAC00-1]
fig|80869.172.peg.2866	T3S	DUF2069 domain-containing protein [*Paracidovorax citrulli*]
fig|80869.172.peg.2891	T3S	GTPase HflX [*Paracidovorax citrulli*]
fig|80869.172.peg.2906	T3S	protein-L-isoaspartate(D-aspartate) O-methyltransferase [*Paracidovorax citrulli*]
fig|80869.172.peg.292	T3S	hypothetical protein [*Paracidovorax citrulli*]
fig|80869.172.peg.2934	T3S	CaiB/BaiF CoA transferase family protein [*Paracidovorax citrulli*]
fig|80869.172.peg.2967	T3S	uncharacterized protein DUF2132 [*Paracidovorax citrulli*]
fig|80869.172.peg.2975	T3S	DNA internalization-related competence protein ComEC/Rec2 [*Paracidovorax citrulli*]
fig|80869.172.peg.2981	T3S	pyridoxal kinase PdxY [*Paracidovorax citrulli*]

Prot	Pred	Description
fig|80869.172.peg.30	T3S	TRAP transporter small permease [*Paracidovorax citrulli*]
fig|80869.172.peg.3007	T3S	hypothetical protein [*Paracidovorax citrulli*]
fig|80869.172.peg.3011	T3S	diguanylate phosphodiesterase [*Paracidovorax citrulli* AAC00-1]
fig|80869.172.peg.3032	T3S	winged helix-turn-helix transcriptional regulator [*Paracidovorax citrulli*]
fig|80869.172.peg.3050	T3S	ABC transporter transmembrane domain-containing protein [*Paracidovorax citrulli*]
fig|80869.172.peg.3058	T3S	MULTISPECIES: NADH-quinone oxidoreductase subunit NuoI [Comamonadaceae]
fig|80869.172.peg.3077	T3S	hypothetical protein [*Paracidovorax citrulli*]
fig|80869.172.peg.3090	T3S	molybdopterin converting factor subunit 1 [*Paracidovorax citrulli*]
fig|80869.172.peg.3105	T3S	nucleotide exchange factor GrpE [*Paracidovorax citrulli*]
fig|80869.172.peg.3118	T3S	phosphoribosylanthranilate isomerase [*Paracidovorax citrulli* AAC00-1]
fig|80869.172.peg.3123	T3S	LON peptidase substrate-binding domain-containing protein [*Paracidovorax citrulli*]
fig|80869.172.peg.3130	T3S	lysine--tRNA ligase [*Paracidovorax citrulli*]
fig|80869.172.peg.3131	T3S	Phytochrome-like protein cph2 [*Paracidovorax citrulli*]
fig|80869.172.peg.3182	T3S	tryptophan 2,3-dioxygenase [*Paracidovorax citrulli*]
fig|80869.172.peg.3191	T3S	neutral zinc metallopeptidase [*Paracidovorax citrulli*]
fig|80869.172.peg.3229	T3S	glycerate kinase [*Paracidovorax citrulli*]
fig|80869.172.peg.3245	T3S	NCS1 family nucleobase:cation symporter-1 [*Paracidovorax citrulli*]
fig|80869.172.peg.3247	T3S	GntR family transcriptional regulator [*Paracidovorax citrulli*]
fig|80869.172.peg.3266	T3S	DMT family transporter [*Paracidovorax citrulli*]
fig|80869.172.peg.3311	T3S	PelD GGDEF domain-containing protein [*Paracidovorax citrulli*]
fig|80869.172.peg.3347	T3S	protein of unknown function DUF1415 [*Paracidovorax citrulli* AAC00-1]
fig|80869.172.peg.3357	T3S	penicillin-binding protein 1A [*Paracidovorax citrulli*]
fig|80869.172.peg.3380	T3S	peptidoglycan-binding domain-containing protein [*Paracidovorax citrulli*]
fig|80869.172.peg.3383	T3S	NCS1 family nucleobase:cation symporter-1 [*Paracidovorax citrulli*]
fig|80869.172.peg.3417	T3S	ABC transporter ATP-binding protein [*Paracidovorax citrulli*]
fig|80869.172.peg.3449	T3S	Holliday junction resolvase RuvX [*Paracidovorax citrulli*]
fig|80869.172.peg.3458	T3S	hydroxymethylpyrimidine/phosphomethylpyrimidine kinase [*Paracidovorax citrulli*]
fig|80869.172.peg.3492	T3S	ribonucleoside-diphosphate reductase subunit alpha [*Paracidovorax citrulli*]
fig|80869.172.peg.3504	T3S	3-deoxy-7-phosphoheptulonate synthase [*Paracidovorax citrulli*]
fig|80869.172.peg.3550	T3S	CobW family GTP-binding protein [*Paracidovorax citrulli*]
fig|80869.172.peg.3561	T3S	chromate efflux transporter [*Paracidovorax citrulli*]
fig|80869.172.peg.3568	T3S	M48 family metallopeptidase [*Paracidovorax citrulli*]
fig|80869.172.peg.3583	T3S	DNA-3-methyladenine glycosylase I [*Paracidovorax citrulli*]
fig|80869.172.peg.3584	T3S	putative zinc protease protein [*Paracidovorax citrulli* AAC00-1]
fig|80869.172.peg.3664	T3S	FMN-dependent NADH-azoreductase [*Paracidovorax citrulli*]
fig|80869.172.peg.3709	T3S	NarK family nitrate/nitrite MFS transporter [*Paracidovorax citrulli*]
fig|80869.172.peg.3711	T3S	transcriptional regulator, AraC family [*Paracidovorax citrulli* AAC00-1]
fig|80869.172.peg.3724	T3S	FAD-linked oxidase C-terminal domain-containing protein [*Paracidovorax citrulli*]
fig|80869.172.peg.3769	T3S	pyrroline-5-carboxylate reductase [*Paracidovorax citrulli*]
fig|80869.172.peg.3771	T3S	glycosyltransferase involved in cell wall biosynthesis [*Paracidovorax citrulli*]
fig|80869.172.peg.3785	T3S	uracil-DNA glycosylase [*Paracidovorax citrulli*]
fig|80869.172.peg.3820	T3S	HNH endonuclease [*Paracidovorax citrulli*]
fig|80869.172.peg.1964	T4S	major facilitator superfamily MFS_1 [*Paracidovorax citrulli* AAC00-1]
fig|80869.172.peg.2076	T4S	hypothetical protein [*Paracidovorax citrulli*]

Prot	Pred	Description
fig|80869.172.peg.3351	T4S	Ig domain protein, group 1 domain protein [*Paracidovorax citrulli* AAC00-1]
fig|80869.172.peg.3830	T4S	MULTISPECIES: DUF3577 domain-containing protein [Pseudomonadota]
fig|80869.172.peg.2160	T4S	acyl-CoA thioesterase [*Paracidovorax citrulli*]
fig|80869.172.peg.1808	T4S	STY0301 family protein [*Paracidovorax citrulli*]
fig|80869.172.peg.1209	T4S	ABC transporter ATP-binding protein [*Paracidovorax citrulli*]
fig|80869.172.peg.1694	T4S	Ku protein [*Paracidovorax citrulli*]
fig|80869.172.peg.2599	T4S	terminase small subunit [*Paracidovorax citrulli*]
fig|80869.172.peg.3083	T4S	molybdenum cofactor biosynthesis protein MoaE [*Paracidovorax citrulli*]
fig|80869.172.peg.250	T4S	bacterioferritin [*Paracidovorax citrulli*]
fig|80869.172.peg.2648	T4S	isoaspartyl peptidase/L-asparaginase family protein [*Paracidovorax citrulli*]
fig|80869.172.peg.352	T4S	abortive infection system antitoxin AbiGi family protein [*Paracidovorax citrulli*]
fig|80869.172.peg.2115	T4S	RNA recognition motif domain-containing protein [*Paracidovorax citrulli*]
fig|80869.172.peg.1514	T4S	hypothetical protein [*Paracidovorax citrulli*]
fig|80869.172.peg.1095	T4S	hypothetical protein Aave_3072 [*Paracidovorax citrulli* AAC00-1]
fig|80869.172.peg.1385	T4S	DUF1795 domain-containing protein [*Paracidovorax citrulli*]
fig|80869.172.peg.3908	T4S	type III secretion system outer membrane ring subunit SctC [*Paracidovorax citrulli*]
fig|80869.172.peg.2100	T4S	hypothetical protein [*Paracidovorax citrulli*]
fig|80869.172.peg.1659	T4S	MULTISPECIES: amino-acid N-acetyltransferase [Paracidovorax]
fig|80869.172.peg.4642	T4S	DUF924 family protein [*Paracidovorax citrulli*]
fig|80869.172.peg.417	T4S	hypothetical protein [*Paracidovorax citrulli*]
fig|80869.172.peg.4705	T4S	50S ribosomal protein L11 [*Paracidovorax citrulli*]
fig|80869.172.peg.1691	T4S	hypothetical protein [*Paracidovorax citrulli*]
fig|80869.172.peg.1989	T4S	conserved hypothetical protein [*Paracidovorax citrulli* AAC00-1]
fig|80869.172.peg.568	T4S	PP2C family protein-serine/threonine phosphatase [*Paracidovorax citrulli*]
fig|80869.172.peg.197	T4S	glutathione-regulated potassium-efflux system protein KefC [*Paracidovorax citrulli*]
fig|80869.172.peg.4326	T4S	hypothetical protein [*Paracidovorax citrulli*]
fig|80869.172.peg.4724	T4S	cryptochrome/photolyase family protein [*Paracidovorax citrulli*]
fig|80869.172.peg.3005	T4S	phosphopyruvate hydratase [*Paracidovorax citrulli*]
fig|80869.172.peg.625	T4S	hypothetical protein [*Paracidovorax citrulli*]
fig|80869.172.peg.900	T4S	hypothetical protein [*Paracidovorax citrulli*]
fig|80869.172.peg.2430	T4S	AAA family ATPase [*Paracidovorax citrulli*]
fig|80869.172.peg.4935	T4S	DUF3025 domain-containing protein [*Paracidovorax citrulli*]
fig|80869.172.peg.567	T4S	serine/threonine protein kinase [*Paracidovorax citrulli*]
fig|80869.172.peg.2193	T4S	inositol monophosphatase family protein [*Paracidovorax citrulli*]
fig|80869.172.peg.5010	T4S	N-acetylmuramoyl-L-alanine amidase [*Paracidovorax citrulli*]
fig|80869.172.peg.3779	T4S	AraC family transcriptional regulator [*Paracidovorax citrulli*]
fig|80869.172.peg.2903	T4S	SMC-Scp complex subunit ScpB [*Paracidovorax citrulli*]
fig|80869.172.peg.839	T4S	hypothetical protein [*Paracidovorax citrulli*]
fig|80869.172.peg.4606	T4S	Fic family protein [*Paracidovorax citrulli*]
fig|80869.172.peg.345	T4S	4-hydroxy-3-methylbut-2-enyl diphosphate reductase [*Paracidovorax citrulli*]
fig|80869.172.peg.779	T4S	SsrA-binding protein SmpB [*Paracidovorax citrulli*]
fig|80869.172.peg.1465	T4S	hypothetical protein [*Paracidovorax citrulli*]
fig|80869.172.peg.4021	T4S	F0F1 ATP synthase subunit gamma [*Paracidovorax citrulli*]
fig|80869.172.peg.2342	T4S	hypothetical protein [*Paracidovorax citrulli*]
fig|80869.172.peg.2876	T4S	hypothetical protein [*Paracidovorax citrulli*]

Prot	Pred	Description
fig|80869.172.peg.1560	T4S	transglycosylase SLT domain-containing protein [*Paracidovorax citrulli*]
fig|80869.172.peg.5146	T4S	hypothetical protein [*Paracidovorax citrulli*]
fig|80869.172.peg.2581	T4S	hypothetical protein [*Paracidovorax citrulli*]
fig|80869.172.peg.135	T4S	5'-nucleotidase [*Paracidovorax citrulli*]
fig|80869.172.peg.1873	T4S	hypothetical protein [*Paracidovorax citrulli*]
fig|80869.172.peg.3744	T4S	30S ribosomal protein S4 [*Paracidovorax citrulli*]
fig|80869.172.peg.1650	T4S	adenylate kinase [*Paracidovorax citrulli*]
fig|80869.172.peg.919	T4S	hypothetical protein [*Paracidovorax citrulli*]
fig|80869.172.peg.599	T4S	type IV pilin protein [*Paracidovorax citrulli*]
fig|80869.172.peg.249	T4S	BON domain-containing protein [*Paracidovorax citrulli*]
fig|80869.172.peg.1673	T4S	MetQ/NlpA family ABC transporter substrate-binding protein [*Paracidovorax citrulli*]
fig|80869.172.peg.4776	T4S	Bug family tripartite tricarboxylate transporter substrate binding protein [*Paracidovorax citrulli*]
fig|80869.172.peg.1257	T4S	hypothetical protein [*Paracidovorax citrulli*]
fig|80869.172.peg.1667	T4S	sulfate ABC transporter substrate-binding protein [*Paracidovorax citrulli*]
fig|80869.172.peg.370	T4S	hypothetical protein [*Paracidovorax citrulli*]
fig|80869.172.peg.4643	T4S	OmpA family protein [*Paracidovorax citrulli*]
fig|80869.172.peg.2564	T4S	glycoside hydrolase family protein [*Paracidovorax citrulli*]
fig|80869.172.peg.4094	T4S	DNA-binding transcriptional LysR family regulator [*Paracidovorax citrulli*]
fig|80869.172.peg.2373	T4S	sigma-54-dependent transcriptional regulator [*Paracidovorax citrulli*]
fig|80869.172.peg.134	T4S	EF-hand domain-containing protein [*Paracidovorax citrulli*]

*P_citrulli*_AAC00-1_5596

Prot	Pred	Description
fig|80869.173.peg.1964	T3S	major facilitator superfamily MFS_1 [*Paracidovorax citrulli* AAC00-1]
fig|80869.173.peg.2076	T3S	hypothetical protein [*Paracidovorax citrulli*]
fig|80869.173.peg.3357	T3S	Ig domain protein, group 1 domain protein [*Paracidovorax citrulli* AAC00-1]
fig|80869.173.peg.3835	T3S	MULTISPECIES: DUF3577 domain-containing protein [Pseudomonadota]
fig|80869.173.peg.2160	T3S	acyl-CoA thioesterase [*Paracidovorax citrulli*]
fig|80869.173.peg.1808	T3S	STY0301 family protein [*Paracidovorax citrulli*]
fig|80869.173.peg.1208	T3S	ABC transporter ATP-binding protein [*Paracidovorax citrulli*]
fig|80869.173.peg.1694	T3S	Ku protein [*Paracidovorax citrulli*]
fig|80869.173.peg.2600	T3S	terminase small subunit [*Paracidovorax citrulli*]
fig|80869.173.peg.1006	T3S	2OG-Fe dioxygenase family protein [*Paracidovorax citrulli*]
fig|80869.173.peg.1029	T3S	TetR/AcrR family transcriptional regulator [*Paracidovorax citrulli*]
fig|80869.173.peg.1048	T3S	RDD domain containing protein [*Paracidovorax citrulli* AAC00-1]
fig|80869.173.peg.105	T3S	ABC transporter permease [*Paracidovorax citrulli*]
fig|80869.173.peg.106	T3S	ABC transporter permease [*Paracidovorax citrulli*]
fig|80869.173.peg.1062	T3S	YbaN family protein [*Paracidovorax citrulli*]
fig|80869.173.peg.1072	T3S	ATP-binding protein [*Paracidovorax citrulli*]
fig|80869.173.peg.1079	T3S	hypothetical protein [*Paracidovorax citrulli*]
fig|80869.173.peg.1138	T3S	tRNA (guanosine(46)-N7)-methyltransferase TrmB [*Paracidovorax citrulli*]
fig|80869.173.peg.1140	T3S	NAD(P)/FAD-dependent oxidoreductase [*Paracidovorax citrulli*]
fig|80869.173.peg.1148	T3S	SulP family inorganic anion transporter [*Paracidovorax citrulli*]
fig|80869.173.peg.1149	T3S	BON domain-containing protein [*Paracidovorax citrulli*]
fig|80869.173.peg.120	T3S	hypothetical protein [*Paracidovorax citrulli*]

Prot	Pred	Description
fig|80869.173.peg.1228	T3S	protein of unknown function UPF0061 [*Paracidovorax citrulli* AAC00-1]
fig|80869.173.peg.1271	T3S	hypothetical protein [*Paracidovorax citrulli*]
fig|80869.173.peg.1343	T3S	pyridoxamine 5'-phosphate oxidase [*Paracidovorax citrulli*]
fig|80869.173.peg.1354	T3S	transcriptional repressor [*Paracidovorax citrulli*]
fig|80869.173.peg.1356	T3S	TetR/AcrR family transcriptional regulator [*Paracidovorax citrulli*]
fig|80869.173.peg.136	T3S	luciferase family protein [*Paracidovorax citrulli* AAC00-1]
fig|80869.173.peg.1375	T3S	EVE domain-containing protein [*Paracidovorax citrulli*]
fig|80869.173.peg.1387	T3S	XopAP family type III secretion system effector [*Paracidovorax citrulli*]
fig|80869.173.peg.1481	T3S	glycoside hydrolase family 5 protein [*Paracidovorax citrulli*]
fig|80869.173.peg.1503	T3S	DEAD/DEAH box helicase [*Paracidovorax citrulli*]
fig|80869.173.peg.1513	T3S	hypothetical protein CQB05_01060 [*Paracidovorax citrulli*]
fig|80869.173.peg.1534	T3S	pseudouridine synthase [*Paracidovorax citrulli*]
fig|80869.173.peg.1538	T3S	translesion DNA synthesis-associated protein ImuA [*Paracidovorax citrulli*]
fig|80869.173.peg.1549	T3S	GTP cyclohydrolase subunit MoaA [*Paracidovorax citrulli* AAC00-1]
fig|80869.173.peg.1552	T3S	gephyrin-like molybdotransferase Glp [*Paracidovorax citrulli*]
fig|80869.173.peg.1582	T3S	phosphate ABC transporter permease PstC [*Paracidovorax citrulli*]
fig|80869.173.peg.1587	T3S	Polyphosphate kinase [*Paracidovorax citrulli* AAC00-1]
fig|80869.173.peg.1600	T3S	hypothetical protein [*Paracidovorax citrulli*]
fig|80869.173.peg.1601	T3S	D-serine/D-alanine/glycine transporter [*Paracidovorax citrulli*]
fig|80869.173.peg.1612	T3S	TonB-dependent receptor family protein [*Paracidovorax citrulli*]
fig|80869.173.peg.163	T3S	hypothetical protein [*Paracidovorax citrulli*]
fig|80869.173.peg.164	T3S	hypothetical protein C8E08_1237 [*Paracidovorax citrulli*]
fig|80869.173.peg.1706	T3S	GCN5-related N-acetyltransferase [*Paracidovorax citrulli* AAC00-1]
fig|80869.173.peg.1734	T3S	EAL domain-containing protein [*Paracidovorax citrulli*]
fig|80869.173.peg.1768	T3S	PAS domain-containing sensor histidine kinase [*Paracidovorax citrulli*]
fig|80869.173.peg.1816	T3S	KGG domain-containing protein [*Paracidovorax citrulli*]
fig|80869.173.peg.1833	T3S	hypothetical protein [*Paracidovorax citrulli*]
fig|80869.173.peg.1940	T3S	DODA-type extradiol aromatic ring-opening family dioxygenase [*Paracidovorax citrulli*]
fig|80869.173.peg.1945	T3S	glutamine--tRNA ligase/YqeY domain fusion protein [*Paracidovorax citrulli*]
fig|80869.173.peg.1957	T3S	esterase/lipase/thioesterase family protein [*Paracidovorax citrulli* AAC00-1]
fig|80869.173.peg.1960	T3S	Murein DD-endopeptidase MepM [*Paracidovorax citrulli*]
fig|80869.173.peg.1970	T3S	DHH family phosphoesterase [*Paracidovorax citrulli*]
fig|80869.173.peg.1974	T3S	redox-sensitive transcriptional activator SoxR [*Paracidovorax citrulli*]
fig|80869.173.peg.1976	T3S	MFS transporter [*Paracidovorax citrulli*]
fig|80869.173.peg.1983	T3S	GAF domain-containing sensor histidine kinase [*Paracidovorax citrulli*]
fig|80869.173.peg.1990	T3S	SMP-30/gluconolactonase/LRE family protein [*Paracidovorax citrulli*]
fig|80869.173.peg.1991	T3S	SDR family NAD(P)-dependent oxidoreductase [*Paracidovorax citrulli*]
fig|80869.173.peg.1997	T3S	multiple monosaccharide ABC transporter permease [*Paracidovorax citrulli*]
fig|80869.173.peg.2031	T3S	cytochrome o ubiquinol oxidase subunit IV [*Paracidovorax citrulli*]
fig|80869.173.peg.2046	T3S	transcriptional regulator, GntR family [*Paracidovorax citrulli* AAC00-1]
fig|80869.173.peg.2061	T3S	NAD-dependent succinate-semialdehyde dehydrogenase [*Paracidovorax citrulli*]
fig|80869.173.peg.2078	T3S	hypothetical protein [*Paracidovorax citrulli*]
fig|80869.173.peg.2107	T3S	hypothetical protein Aave_2148 [*Paracidovorax citrulli* AAC00-1]
fig|80869.173.peg.2128	T3S	DUF2169 domain-containing protein [*Paracidovorax citrulli*]
fig|80869.173.peg.2133	T3S	excinuclease ABC subunit UvrA [*Paracidovorax citrulli*]
fig|80869.173.peg.2141	T3S	carboxyl transferase domain-containing protein [*Paracidovorax citrulli*]

Prot	Pred	Description
fig|80869.173.peg.2158	T3S	uroporphyrinogen-III C-methyltransferase [*Paracidovorax citrulli*]
fig|80869.173.peg.2181	T3S	succinate-semialdehyde dehydrogenase/glutarate-semialdehyde dehydrogenase [*Paracidovorax citrulli*]
fig|80869.173.peg.2224	T3S	efflux transporter outer membrane subunit [*Paracidovorax citrulli*]
fig|80869.173.peg.2235	T3S	TOBE domain-containing protein [*Paracidovorax citrulli*]
fig|80869.173.peg.2240	T3S	BCCT family transporter [*Paracidovorax citrulli*]
fig|80869.173.peg.2243	T3S	alpha-1,4-glucan--maltose-1-phosphate maltosyltransferase [*Paracidovorax citrulli*]
fig|80869.173.peg.2253	T3S	exodeoxyribonuclease VII small subunit [*Paracidovorax citrulli*]
fig|80869.173.peg.2258	T3S	SfnB family sulfur acquisition oxidoreductase [*Paracidovorax citrulli*]
fig|80869.173.peg.2259	T3S	SfnB family sulfur acquisition oxidoreductase [*Paracidovorax citrulli*]
fig|80869.173.peg.2265	T3S	AI-2E family transporter [*Paracidovorax citrulli*]
fig|80869.173.peg.2267	T3S	phosphoenolpyruvate carboxylase [*Paracidovorax citrulli*]
fig|80869.173.peg.2268	T3S	hydroxymethylbilane synthase [*Paracidovorax citrulli*]
fig|80869.173.peg.2270	T3S	uroporphyrinogen-III C-methyltransferase [*Paracidovorax citrulli*]
fig|80869.173.peg.2272	T3S	ABC-F family ATP-binding cassette domain-containing protein [*Paracidovorax citrulli*]
fig|80869.173.peg.2285	T3S	hypothetical protein [*Paracidovorax citrulli*]
fig|80869.173.peg.2294	T3S	CDP-6-deoxy-delta-3,4-glucoseen reductase [*Paracidovorax citrulli*]
fig|80869.173.peg.232	T3S	MATE family efflux transporter [*Paracidovorax citrulli*]
fig|80869.173.peg.2322	T3S	argininosuccinate lyase [*Paracidovorax citrulli*]
fig|80869.173.peg.2406	T3S	DUF72 domain-containing protein [*Paracidovorax citrulli*]
fig|80869.173.peg.2409	T3S	DUF1624 domain-containing protein [*Paracidovorax citrulli*]
fig|80869.173.peg.2410	T3S	glutamate--tRNA ligase [*Paracidovorax citrulli*]
fig|80869.173.peg.243	T3S	signal recognition particle-docking protein FtsY [*Paracidovorax citrulli*]
fig|80869.173.peg.2443	T3S	UDP-3-O-(3-hydroxymyristoyl)glucosamine N-acyltransferase [*Paracidovorax citrulli*]
fig|80869.173.peg.2458	T3S	MlaD family protein [*Paracidovorax citrulli*]
fig|80869.173.peg.2462	T3S	YitT family protein [*Paracidovorax citrulli*]
fig|80869.173.peg.2473	T3S	P1 family peptidase [*Paracidovorax citrulli*]
fig|80869.173.peg.2478	T3S	ATP synthase F1 subunit epsilon [*Paracidovorax citrulli*]
fig|80869.173.peg.2492	T3S	structural protein P5 [*Paracidovorax citrulli*]
fig|80869.173.peg.2495	T3S	hypothetical protein [*Paracidovorax citrulli*]
fig|80869.173.peg.2603	T3S	hypothetical protein [*Paracidovorax citrulli*]
fig|80869.173.peg.2616	T3S	hypothetical protein Aave_1685 [*Paracidovorax citrulli* AAC00-1]
fig|80869.173.peg.264	T3S	3-methyl-2-oxobutanoate hydroxymethyltransferase [*Paracidovorax citrulli*]
fig|80869.173.peg.2674	T3S	hypothetical protein [*Paracidovorax citrulli*]
fig|80869.173.peg.2704	T3S	hypothetical protein Aave_1606 [*Paracidovorax citrulli* AAC00-1]
fig|80869.173.peg.2714	T3S	polyhydroxyalkanoate synthesis repressor PhaR [*Paracidovorax citrulli*]
fig|80869.173.peg.2716	T3S	type 1 glutamine amidotransferase domain-containing protein [*Paracidovorax citrulli*]
fig|80869.173.peg.2764	T3S	SDR family NAD(P)-dependent oxidoreductase [*Paracidovorax citrulli*]
fig|80869.173.peg.2794	T3S	heavy metal translocating P-type ATPase [*Paracidovorax citrulli*]
fig|80869.173.peg.28	T3S	TRAP transporter small permease [*Paracidovorax citrulli*]
fig|80869.173.peg.280	T3S	hypothetical protein [*Paracidovorax citrulli*]
fig|80869.173.peg.2802	T3S	malonate--CoA ligase [*Paracidovorax citrulli*]
fig|80869.173.peg.281	T3S	YitT family protein [*Paracidovorax citrulli*]
fig|80869.173.peg.2822	T3S	glycine betaine/L-proline ABC transporter permease ProW [*Paracidovorax citrulli*]
fig|80869.173.peg.2862	T3S	farnesyl-diphosphate farnesyltransferase [*Paracidovorax citrulli* AAC00-1]

Prot	Pred	Description
fig|80869.173.peg.2865	T3S	DUF2069 domain-containing protein [*Paracidovorax citrulli*]
fig|80869.173.peg.2890	T3S	GTPase HflX [*Paracidovorax citrulli*]
fig|80869.173.peg.2905	T3S	protein-L-isoaspartate(D-aspartate) O-methyltransferase [*Paracidovorax citrulli*]
fig|80869.173.peg.293	T3S	hypothetical protein [*Paracidovorax citrulli*]
fig|80869.173.peg.2934	T3S	CaiB/BaiF CoA transferase family protein [*Paracidovorax citrulli*]
fig|80869.173.peg.2967	T3S	uncharacterized protein DUF2132 [*Paracidovorax citrulli*]
fig|80869.173.peg.2975	T3S	DNA internalization-related competence protein ComEC/Rec2 [*Paracidovorax citrulli*]
fig|80869.173.peg.2981	T3S	pyridoxal kinase PdxY [*Paracidovorax citrulli*]
fig|80869.173.peg.3007	T3S	hypothetical protein [*Paracidovorax citrulli*]
fig|80869.173.peg.3011	T3S	diguanylate phosphodiesterase [*Paracidovorax citrulli* AAC00-1]
fig|80869.173.peg.3032	T3S	winged helix-turn-helix transcriptional regulator [*Paracidovorax citrulli*]
fig|80869.173.peg.3050	T3S	ABC transporter transmembrane domain-containing protein [*Paracidovorax citrulli*]
fig|80869.173.peg.3058	T3S	MULTISPECIES: NADH-quinone oxidoreductase subunit NuoI [Comamonadaceae]
fig|80869.173.peg.3078	T3S	hypothetical protein [*Paracidovorax citrulli*]
fig|80869.173.peg.3091	T3S	molybdopterin converting factor subunit 1 [*Paracidovorax citrulli*]
fig|80869.173.peg.3106	T3S	nucleotide exchange factor GrpE [*Paracidovorax citrulli*]
fig|80869.173.peg.3124	T3S	LON peptidase substrate-binding domain-containing protein [*Paracidovorax citrulli*]
fig|80869.173.peg.3131	T3S	lysine--tRNA ligase [*Paracidovorax citrulli*]
fig|80869.173.peg.3132	T3S	Phytochrome-like protein cph2 [*Paracidovorax citrulli*]
fig|80869.173.peg.3184	T3S	tryptophan 2,3-dioxygenase [*Paracidovorax citrulli*]
fig|80869.173.peg.3193	T3S	neutral zinc metallopeptidase [*Paracidovorax citrulli*]
fig|80869.173.peg.3231	T3S	glycerate kinase [*Paracidovorax citrulli*]
fig|80869.173.peg.3251	T3S	NCS1 family nucleobase:cation symporter-1 [*Paracidovorax citrulli*]
fig|80869.173.peg.3253	T3S	GntR family transcriptional regulator [*Paracidovorax citrulli*]
fig|80869.173.peg.3272	T3S	DMT family transporter [*Paracidovorax citrulli*]
fig|80869.173.peg.3317	T3S	PelD GGDEF domain-containing protein [*Paracidovorax citrulli*]
fig|80869.173.peg.3353	T3S	protein of unknown function DUF1415 [*Paracidovorax citrulli* AAC00-1]
fig|80869.173.peg.3363	T3S	penicillin-binding protein 1A [*Paracidovorax citrulli*]
fig|80869.173.peg.3386	T3S	peptidoglycan-binding domain-containing protein [*Paracidovorax citrulli*]
fig|80869.173.peg.3389	T3S	NCS1 family nucleobase:cation symporter-1 [*Paracidovorax citrulli*]
fig|80869.173.peg.3422	T3S	ABC transporter ATP-binding protein [*Paracidovorax citrulli*]
fig|80869.173.peg.3454	T3S	Holliday junction resolvase RuvX [*Paracidovorax citrulli*]
fig|80869.173.peg.3463	T3S	hydroxymethylpyrimidine/phosphomethylpyrimidine kinase [*Paracidovorax citrulli*]
fig|80869.173.peg.3497	T3S	ribonucleoside-diphosphate reductase subunit alpha [*Paracidovorax citrulli*]
fig|80869.173.peg.3509	T3S	3-deoxy-7-phosphoheptulonate synthase [*Paracidovorax citrulli*]
fig|80869.173.peg.3555	T3S	CobW family GTP-binding protein [*Paracidovorax citrulli*]
fig|80869.173.peg.3566	T3S	chromate efflux transporter [*Paracidovorax citrulli*]
fig|80869.173.peg.3573	T3S	M48 family metallopeptidase [*Paracidovorax citrulli*]
fig|80869.173.peg.3588	T3S	DNA-3-methyladenine glycosylase I [*Paracidovorax citrulli*]
fig|80869.173.peg.3589	T3S	putative zinc protease protein [*Paracidovorax citrulli* AAC00-1]
fig|80869.173.peg.3669	T3S	FMN-dependent NADH-azoreductase [*Paracidovorax citrulli*]
fig|80869.173.peg.3714	T3S	NarK family nitrate/nitrite MFS transporter [*Paracidovorax citrulli*]
fig|80869.173.peg.3716	T3S	transcriptional regulator, AraC family [*Paracidovorax citrulli* AAC00-1]
fig|80869.173.peg.3729	T3S	FAD-linked oxidase C-terminal domain-containing protein [*Paracidovorax citrulli*]

Prot	Pred	Description
fig|80869.173.peg.3774	T3S	pyrroline-5-carboxylate reductase [*Paracidovorax citrulli*]
fig|80869.173.peg.3776	T3S	glycosyltransferase involved in cell wall biosynthesis [*Paracidovorax citrulli*]
fig|80869.173.peg.3790	T3S	uracil-DNA glycosylase [*Paracidovorax citrulli*]
fig|80869.173.peg.3825	T3S	HNH endonuclease [*Paracidovorax citrulli*]
fig|80869.173.peg.3845	T3S	MULTISPECIES: hypothetical protein [Pseudomonadota]
fig|80869.173.peg.1964	T4S	major facilitator superfamily MFS_1 [*Paracidovorax citrulli* AAC00-1]
fig|80869.173.peg.2076	T4S	hypothetical protein [*Paracidovorax citrulli*]
fig|80869.173.peg.3357	T4S	Ig domain protein, group 1 domain protein [*Paracidovorax citrulli* AAC00-1]
fig|80869.173.peg.3835	T4S	MULTISPECIES: DUF3577 domain-containing protein [Pseudomonadota]
fig|80869.173.peg.2160	T4S	acyl-CoA thioesterase [*Paracidovorax citrulli*]
fig|80869.173.peg.1808	T4S	STY0301 family protein [*Paracidovorax citrulli*]
fig|80869.173.peg.1208	T4S	ABC transporter ATP-binding protein [*Paracidovorax citrulli*]
fig|80869.173.peg.1694	T4S	Ku protein [*Paracidovorax citrulli*]
fig|80869.173.peg.2600	T4S	terminase small subunit [*Paracidovorax citrulli*]
fig|80869.173.peg.3084	T4S	molybdenum cofactor biosynthesis protein MoaE [*Paracidovorax citrulli*]
fig|80869.173.peg.250	T4S	bacterioferritin [*Paracidovorax citrulli*]
fig|80869.173.peg.2649	T4S	isoaspartyl peptidase/L-asparaginase family protein [*Paracidovorax citrulli*]
fig|80869.173.peg.352	T4S	abortive infection system antitoxin AbiGi family protein [*Paracidovorax citrulli*]
fig|80869.173.peg.2115	T4S	RNA recognition motif domain-containing protein [*Paracidovorax citrulli*]
fig|80869.173.peg.1512	T4S	hypothetical protein [*Paracidovorax citrulli*]
fig|80869.173.peg.1095	T4S	hypothetical protein Aave_3072 [*Paracidovorax citrulli* AAC00-1]
fig|80869.173.peg.1381	T4S	DUF1795 domain-containing protein [*Paracidovorax citrulli*]
fig|80869.173.peg.3913	T4S	type III secretion system outer membrane ring subunit SctC [*Paracidovorax citrulli*]
fig|80869.173.peg.2100	T4S	hypothetical protein [*Paracidovorax citrulli*]
fig|80869.173.peg.1659	T4S	MULTISPECIES: amino-acid N-acetyltransferase [Paracidovorax]
fig|80869.173.peg.4644	T4S	DUF924 family protein [*Paracidovorax citrulli*]
fig|80869.173.peg.416	T4S	hypothetical protein [*Paracidovorax citrulli*]
fig|80869.173.peg.4707	T4S	50S ribosomal protein L11 [*Paracidovorax citrulli*]
fig|80869.173.peg.1691	T4S	hypothetical protein [*Paracidovorax citrulli*]
fig|80869.173.peg.1989	T4S	conserved hypothetical protein [*Paracidovorax citrulli* AAC00-1]
fig|80869.173.peg.568	T4S	PP2C family protein-serine/threonine phosphatase [*Paracidovorax citrulli*]
fig|80869.173.peg.197	T4S	glutathione-regulated potassium-efflux system protein KefC [*Paracidovorax citrulli*]
fig|80869.173.peg.4331	T4S	hypothetical protein [*Paracidovorax citrulli*]
fig|80869.173.peg.4726	T4S	cryptochrome/photolyase family protein [*Paracidovorax citrulli*]
fig|80869.173.peg.3005	T4S	phosphopyruvate hydratase [*Paracidovorax citrulli*]
fig|80869.173.peg.626	T4S	hypothetical protein [*Paracidovorax citrulli*]
fig|80869.173.peg.900	T4S	hypothetical protein [*Paracidovorax citrulli*]
fig|80869.173.peg.2430	T4S	AAA family ATPase [*Paracidovorax citrulli*]
fig|80869.173.peg.4938	T4S	DUF3025 domain-containing protein [*Paracidovorax citrulli*]
fig|80869.173.peg.567	T4S	serine/threonine protein kinase [*Paracidovorax citrulli*]
fig|80869.173.peg.2193	T4S	inositol monophosphatase family protein [*Paracidovorax citrulli*]
fig|80869.173.peg.5014	T4S	N-acetylmuramoyl-L-alanine amidase [*Paracidovorax citrulli*]
fig|80869.173.peg.3784	T4S	AraC family transcriptional regulator [*Paracidovorax citrulli*]
fig|80869.173.peg.2902	T4S	SMC-Scp complex subunit ScpB [*Paracidovorax citrulli*]
fig|80869.173.peg.840	T4S	hypothetical protein [*Paracidovorax citrulli*]

Prot	Pred	Description
fig|80869.173.peg.4608	T4S	Fic family protein [*Paracidovorax citrulli*]
fig|80869.173.peg.345	T4S	4-hydroxy-3-methylbut-2-enyl diphosphate reductase [*Paracidovorax citrulli*]
fig|80869.173.peg.780	T4S	SsrA-binding protein SmpB [*Paracidovorax citrulli*]
fig|80869.173.peg.1464	T4S	hypothetical protein [*Paracidovorax citrulli*]
fig|80869.173.peg.4026	T4S	F0F1 ATP synthase subunit gamma [*Paracidovorax citrulli*]
fig|80869.173.peg.2342	T4S	hypothetical protein [*Paracidovorax citrulli*]
fig|80869.173.peg.2875	T4S	hypothetical protein [*Paracidovorax citrulli*]
fig|80869.173.peg.1558	T4S	transglycosylase SLT domain-containing protein [*Paracidovorax citrulli*]
fig|80869.173.peg.5150	T4S	hypothetical protein [*Paracidovorax citrulli*]
fig|80869.173.peg.2581	T4S	hypothetical protein [*Paracidovorax citrulli*]
fig|80869.173.peg.134	T4S	5'-nucleotidase [*Paracidovorax citrulli*]
fig|80869.173.peg.1873	T4S	hypothetical protein [*Paracidovorax citrulli*]
fig|80869.173.peg.3749	T4S	30S ribosomal protein S4 [*Paracidovorax citrulli*]
fig|80869.173.peg.1650	T4S	adenylate kinase [*Paracidovorax citrulli*]
fig|80869.173.peg.919	T4S	hypothetical protein [*Paracidovorax citrulli*]
fig|80869.173.peg.599	T4S	type IV pilin protein [*Paracidovorax citrulli*]
fig|80869.173.peg.249	T4S	BON domain-containing protein [*Paracidovorax citrulli*]
fig|80869.173.peg.1673	T4S	MetQ/NlpA family ABC transporter substrate-binding protein [*Paracidovorax citrulli*]
fig|80869.173.peg.4778	T4S	Bug family tripartite tricarboxylate transporter substrate binding protein [*Paracidovorax citrulli*]
fig|80869.173.peg.1255	T4S	hypothetical protein [*Paracidovorax citrulli*]
fig|80869.173.peg.1667	T4S	sulfate ABC transporter substrate-binding protein [*Paracidovorax citrulli*]
fig|80869.173.peg.370	T4S	hypothetical protein [*Paracidovorax citrulli*]
fig|80869.173.peg.4645	T4S	OmpA family protein [*Paracidovorax citrulli*]
fig|80869.173.peg.2563	T4S	glycoside hydrolase family protein [*Paracidovorax citrulli*]
fig|80869.173.peg.4099	T4S	DNA-binding transcriptional LysR family regulator [*Paracidovorax citrulli*]
fig|80869.173.peg.2373	T4S	sigma-54-dependent transcriptional regulator [*Paracidovorax citrulli*]
fig|80869.173.peg.133	T4S	EF-hand domain-containing protein [*Paracidovorax citrulli*]

*P_citrulli*_AAC00-1_5684

Prot	Pred	Description
fig|80869.174.peg.2784	T3S	MULTISPECIES: DUF3577 domain-containing protein [Pseudomonadota]
fig|80869.174.peg.3262	T3S	Ig domain protein, group 1 domain protein [*Paracidovorax citrulli* AAC00-1]
fig|80869.174.peg.4539	T3S	hypothetical protein [*Paracidovorax citrulli*]
fig|80869.174.peg.4651	T3S	major facilitator superfamily MFS_1 [*Paracidovorax citrulli* AAC00-1]
fig|80869.174.peg.4456	T3S	acyl-CoA thioesterase [*Paracidovorax citrulli*]
fig|80869.174.peg.4807	T3S	STY0301 family protein [*Paracidovorax citrulli*]
fig|80869.174.peg.231	T3S	ABC transporter ATP-binding protein [*Paracidovorax citrulli*]
fig|80869.174.peg.4921	T3S	Ku protein [*Paracidovorax citrulli*]
fig|80869.174.peg.4015	T3S	terminase small subunit [*Paracidovorax citrulli*]
fig|80869.174.peg.1038	T3S	hypothetical protein [*Paracidovorax citrulli*]
fig|80869.174.peg.1039	T3S	4'-phosphopantetheinyl transferase [*Paracidovorax citrulli* AAC00-1]
fig|80869.174.peg.1044	T3S	GNAT family N-acetyltransferase [*Paracidovorax citrulli*]
fig|80869.174.peg.1152	T3S	hypothetical protein [*Paracidovorax citrulli*]
fig|80869.174.peg.1164	T3S	YitT family protein [*Paracidovorax citrulli*]

Prot	Pred	Description
fig|80869.174.peg.1165	T3S	hypothetical protein [*Paracidovorax citrulli*]
fig|80869.174.peg.1181	T3S	3-methyl-2-oxobutanoate hydroxymethyltransferase [*Paracidovorax citrulli*]
fig|80869.174.peg.1202	T3S	signal recognition particle-docking protein FtsY [*Paracidovorax citrulli*]
fig|80869.174.peg.1213	T3S	MATE family efflux transporter [*Paracidovorax citrulli*]
fig|80869.174.peg.1280	T3S	type III secretion system chaperone [*Paracidovorax citrulli*]
fig|80869.174.peg.1281	T3S	hypothetical protein [*Paracidovorax citrulli*]
fig|80869.174.peg.1308	T3S	luciferase family protein [*Paracidovorax citrulli* AAC00-1]
fig|80869.174.peg.1324	T3S	hypothetical protein [*Paracidovorax citrulli*]
fig|80869.174.peg.1338	T3S	ABC transporter permease [*Paracidovorax citrulli*]
fig|80869.174.peg.1339	T3S	ABC transporter permease [*Paracidovorax citrulli*]
fig|80869.174.peg.1351	T3S	(2Fe-2S)-binding protein [*Paracidovorax citrulli*]
fig|80869.174.peg.1352	T3S	molybdenum cofactor cytidylyltransferase [*Paracidovorax citrulli*]
fig|80869.174.peg.1358	T3S	hypothetical protein [*Paracidovorax citrulli*]
fig|80869.174.peg.1359	T3S	NUDIX hydrolase [*Paracidovorax citrulli*]
fig|80869.174.peg.1370	T3S	alpha/beta hydrolase [*Paracidovorax citrulli*]
fig|80869.174.peg.1395	T3S	septal ring lytic transglycosylase RlpA family protein [*Paracidovorax citrulli*]
fig|80869.174.peg.1415	T3S	TRAP transporter small permease [*Paracidovorax citrulli*]
fig|80869.174.peg.1521	T3S	rhodanese-like domain-containing protein [*Paracidovorax citrulli*]
fig|80869.174.peg.1537	T3S	GNAT family N-acetyltransferase [*Paracidovorax citrulli*]
fig|80869.174.peg.1549	T3S	ABC transporter permease [*Paracidovorax citrulli*]
fig|80869.174.peg.1554	T3S	MBL fold metallo-hydrolase [*Paracidovorax citrulli*]
fig|80869.174.peg.1563	T3S	glycoside hydrolase 15-related protein [*Paracidovorax citrulli* AAC00-1]
fig|80869.174.peg.1591	T3S	hypothetical protein [*Paracidovorax citrulli*]
fig|80869.174.peg.1616	T3S	ABC transporter permease [*Paracidovorax citrulli*]
fig|80869.174.peg.168	T3S	hypothetical protein [*Paracidovorax citrulli*]
fig|80869.174.peg.1685	T3S	ABC transporter ATP-binding protein [*Paracidovorax citrulli*]
fig|80869.174.peg.1720	T3S	AraC family transcriptional regulator [*Paracidovorax citrulli*]
fig|80869.174.peg.1743	T3S	chemoreceptor glutamine deamidase CheD [*Paracidovorax citrulli*]
fig|80869.174.peg.1744	T3S	CheR family methyltransferase [*Paracidovorax citrulli*]
fig|80869.174.peg.1768	T3S	flagellin [*Paracidovorax citrulli*]
fig|80869.174.peg.1769	T3S	flagellin [*Paracidovorax citrulli*]
fig|80869.174.peg.1772	T3S	conserved hypothetical protein [*Paracidovorax citrulli* AAC00-1]
fig|80869.174.peg.1785	T3S	hypothetical protein [*Paracidovorax citrulli*]
fig|80869.174.peg.1790	T3S	flagellar hook assembly protein FlgD [*Paracidovorax citrulli*]
fig|80869.174.peg.1807	T3S	DMT family transporter [*Paracidovorax citrulli*]
fig|80869.174.peg.1813	T3S	hypothetical protein [*Paracidovorax citrulli*]
fig|80869.174.peg.1818	T3S	PepSY-associated TM helix domain-containing protein [*Paracidovorax citrulli*]
fig|80869.174.peg.1841	T3S	MarR family winged helix-turn-helix transcriptional regulator [*Paracidovorax citrulli*]
fig|80869.174.peg.1861	T3S	hypothetical protein C8E08_1798 [*Paracidovorax citrulli*]
fig|80869.174.peg.1883	T3S	PhaM family polyhydroxyalkanoate granule multifunctional regulatory protein [*Paracidovorax citrulli*]
fig|80869.174.peg.1901	T3S	sensor histidine kinase [*Paracidovorax citrulli*]
fig|80869.174.peg.1903	T3S	16S rRNA (cytosine(967)-C(5))-methyltransferase RsmB [*Paracidovorax citrulli*]
fig|80869.174.peg.1958	T3S	phosphatase PAP2 family protein [*Paracidovorax citrulli*]
fig|80869.174.peg.1977	T3S	gamma-glutamyl-gamma-aminobutyrate hydrolase family protein [*Paracidovorax citrulli*]

Prot	Pred	Description
fig|80869.174.peg.1997	T3S	transcriptional regulator, TetR family [*Paracidovorax citrulli* AAC00-1]
fig|80869.174.peg.2036	T3S	putative MFS family arabinose efflux permease [*Paracidovorax citrulli*]
fig|80869.174.peg.2052	T3S	CerR family C-terminal domain-containing protein [*Paracidovorax citrulli*]
fig|80869.174.peg.2095	T3S	O-acetylserine/cysteine efflux transporter [*Paracidovorax citrulli*]
fig|80869.174.peg.2097	T3S	DUF1800 family protein [*Paracidovorax citrulli*]
fig|80869.174.peg.211	T3S	protein of unknown function UPF0061 [*Paracidovorax citrulli* AAC00-1]
fig|80869.174.peg.2112	T3S	MBL fold metallo-hydrolase [*Paracidovorax citrulli*]
fig|80869.174.peg.2124	T3S	response regulator [*Paracidovorax citrulli*]
fig|80869.174.peg.2135	T3S	flagellar basal body protein [*Paracidovorax citrulli*]
fig|80869.174.peg.2136	T3S	transcriptional regulator, LysR family [*Paracidovorax citrulli* AAC00-1]
fig|80869.174.peg.2141	T3S	diguanylate cyclase [*Paracidovorax citrulli* AAC00-1]
fig|80869.174.peg.2161	T3S	protein of unknown function DUF558 [*Paracidovorax citrulli* AAC00-1]
fig|80869.174.peg.2180	T3S	KdsC family phosphatase [*Paracidovorax citrulli*]
fig|80869.174.peg.2184	T3S	PepSY domain-containing protein [*Paracidovorax citrulli*]
fig|80869.174.peg.2196	T3S	DNA topoisomerase (ATP-hydrolyzing) subunit B [*Paracidovorax citrulli*]
fig|80869.174.peg.2243	T3S	ATP-binding cassette domain-containing protein [*Paracidovorax citrulli*]
fig|80869.174.peg.2266	T3S	sirohydrochlorin chelatase [*Paracidovorax citrulli*]
fig|80869.174.peg.2303	T3S	hotdog fold thioesterase [*Paracidovorax citrulli*]
fig|80869.174.peg.2307	T3S	hypothetical protein [*Paracidovorax citrulli*]
fig|80869.174.peg.2315	T3S	MBL fold metallo-hydrolase [*Paracidovorax citrulli*]
fig|80869.174.peg.2319	T3S	5-demethoxyubiquinol-8 5-hydroxylase UbiM [*Paracidovorax citrulli*]
fig|80869.174.peg.2373	T3S	aromatic ring-hydroxylating dioxygenase subunit alpha [*Paracidovorax citrulli*]
fig|80869.174.peg.2385	T3S	Cupin 2, conserved barrel domain protein [*Paracidovorax citrulli* AAC00-1]
fig|80869.174.peg.2438	T3S	GNAT family N-acetyltransferase [Paracidovorax avenae]
fig|80869.174.peg.2459	T3S	ProQ/FINO family protein [*Paracidovorax citrulli*]
fig|80869.174.peg.2491	T3S	AmpG family muropeptide MFS transporter [*Paracidovorax citrulli*]
fig|80869.174.peg.2494	T3S	DME family drug/metabolite transporter [*Paracidovorax citrulli*]
fig|80869.174.peg.2496	T3S	SLC13 family permease [*Paracidovorax citrulli*]
fig|80869.174.peg.2515	T3S	amino acid ABC transporter ATP-binding protein [*Paracidovorax citrulli*]
fig|80869.174.peg.2540	T3S	DHA2 family efflux MFS transporter permease subunit [*Paracidovorax citrulli*]
fig|80869.174.peg.2577	T3S	potassium-transporting ATPase subunit KdpB [*Paracidovorax citrulli*]
fig|80869.174.peg.2600	T3S	TPM domain-containing protein [*Paracidovorax citrulli*]
fig|80869.174.peg.2608	T3S	Kelch repeat-containing protein [*Paracidovorax citrulli*]
fig|80869.174.peg.2645	T3S	TspO/MBR family protein [*Paracidovorax citrulli*]
fig|80869.174.peg.2650	T3S	hypothetical protein [*Paracidovorax citrulli*]
fig|80869.174.peg.2657	T3S	DUF4139 domain-containing protein [*Paracidovorax citrulli*]
fig|80869.174.peg.2686	T3S	hypothetical protein Aave_0457 [*Paracidovorax citrulli* AAC00-1]
fig|80869.174.peg.2690	T3S	hypothetical protein [*Paracidovorax citrulli*]
fig|80869.174.peg.2691	T3S	hypothetical protein [*Paracidovorax citrulli*]
fig|80869.174.peg.2702	T3S	hypothetical protein [*Paracidovorax citrulli*]
fig|80869.174.peg.2708	T3S	hypothetical protein [*Paracidovorax citrulli*]
fig|80869.174.peg.2774	T3S	MULTISPECIES: hypothetical protein [Pseudomonadota]
fig|80869.174.peg.2794	T3S	HNH endonuclease [*Paracidovorax citrulli*]
fig|80869.174.peg.2829	T3S	uracil-DNA glycosylase [*Paracidovorax citrulli*]
fig|80869.174.peg.2843	T3S	glycosyltransferase involved in cell wall biosynthesis [*Paracidovorax citrulli*]

Prot	Pred	Description
fig|80869.174.peg.2845	T3S	pyrroline-5-carboxylate reductase [*Paracidovorax citrulli*]
fig|80869.174.peg.2890	T3S	FAD-linked oxidase C-terminal domain-containing protein [*Paracidovorax citrulli*]
fig|80869.174.peg.290	T3S	BON domain-containing protein [*Paracidovorax citrulli*]
fig|80869.174.peg.2903	T3S	transcriptional regulator, AraC family [*Paracidovorax citrulli* AAC00-1]
fig|80869.174.peg.2905	T3S	NarK family nitrate/nitrite MFS transporter [*Paracidovorax citrulli*]
fig|80869.174.peg.291	T3S	SulP family inorganic anion transporter [*Paracidovorax citrulli*]
fig|80869.174.peg.2950	T3S	FMN-dependent NADH-azoreductase [*Paracidovorax citrulli*]
fig|80869.174.peg.299	T3S	NAD(P)/FAD-dependent oxidoreductase [*Paracidovorax citrulli*]
fig|80869.174.peg.301	T3S	tRNA (guanosine(46)-N7)-methyltransferase TrmB [*Paracidovorax citrulli*]
fig|80869.174.peg.3030	T3S	putative zinc protease protein [*Paracidovorax citrulli* AAC00-1]
fig|80869.174.peg.3031	T3S	DNA-3-methyladenine glycosylase I [*Paracidovorax citrulli*]
fig|80869.174.peg.3046	T3S	M48 family metallopeptidase [*Paracidovorax citrulli*]
fig|80869.174.peg.3053	T3S	chromate efflux transporter [*Paracidovorax citrulli*]
fig|80869.174.peg.3064	T3S	CobW family GTP-binding protein [*Paracidovorax citrulli*]
fig|80869.174.peg.3110	T3S	3-deoxy-7-phosphoheptulonate synthase [*Paracidovorax citrulli*]
fig|80869.174.peg.3122	T3S	ribonucleoside-diphosphate reductase subunit alpha [*Paracidovorax citrulli*]
fig|80869.174.peg.3156	T3S	hydroxymethylpyrimidine/phosphomethylpyrimidine kinase [*Paracidovorax citrulli*]
fig|80869.174.peg.3165	T3S	Holliday junction resolvase RuvX [*Paracidovorax citrulli*]
fig|80869.174.peg.3197	T3S	ABC transporter ATP-binding protein [*Paracidovorax citrulli*]
fig|80869.174.peg.3229	T3S	NCS1 family nucleobase:cation symporter-1 [*Paracidovorax citrulli*]
fig|80869.174.peg.3232	T3S	peptidoglycan-binding domain-containing protein [*Paracidovorax citrulli*]
fig|80869.174.peg.3234	T3S	hypothetical protein Aave_0973 [*Paracidovorax citrulli* AAC00-1]
fig|80869.174.peg.3256	T3S	penicillin-binding protein 1A [*Paracidovorax citrulli*]
fig|80869.174.peg.3266	T3S	protein of unknown function DUF1415 [*Paracidovorax citrulli* AAC00-1]
fig|80869.174.peg.3302	T3S	PelD GGDEF domain-containing protein [*Paracidovorax citrulli*]
fig|80869.174.peg.3346	T3S	DMT family transporter [*Paracidovorax citrulli*]
fig|80869.174.peg.3365	T3S	GntR family transcriptional regulator [*Paracidovorax citrulli*]
fig|80869.174.peg.3367	T3S	NCS1 family nucleobase:cation symporter-1 [*Paracidovorax citrulli*]
fig|80869.174.peg.3383	T3S	glycerate kinase [*Paracidovorax citrulli*]
fig|80869.174.peg.3421	T3S	neutral zinc metallopeptidase [*Paracidovorax citrulli*]
fig|80869.174.peg.3430	T3S	tryptophan 2,3-dioxygenase [*Paracidovorax citrulli*]
fig|80869.174.peg.3481	T3S	Phytochrome-like protein cph2 [*Paracidovorax citrulli*]
fig|80869.174.peg.3482	T3S	lysine--tRNA ligase [*Paracidovorax citrulli*]
fig|80869.174.peg.3489	T3S	LON peptidase substrate-binding domain-containing protein [*Paracidovorax citrulli*]
fig|80869.174.peg.3507	T3S	nucleotide exchange factor GrpE [*Paracidovorax citrulli*]
fig|80869.174.peg.3522	T3S	molybdopterin converting factor subunit 1 [*Paracidovorax citrulli*]
fig|80869.174.peg.3535	T3S	hypothetical protein [*Paracidovorax citrulli*]
fig|80869.174.peg.3555	T3S	MULTISPECIES: NADH-quinone oxidoreductase subunit NuoI [Comamonadaceae]
fig|80869.174.peg.3563	T3S	ABC transporter transmembrane domain-containing protein [*Paracidovorax citrulli*]
fig|80869.174.peg.3581	T3S	winged helix-turn-helix transcriptional regulator [*Paracidovorax citrulli*]
fig|80869.174.peg.3602	T3S	diguanylate phosphodiesterase [*Paracidovorax citrulli* AAC00-1]
fig|80869.174.peg.3606	T3S	hypothetical protein [*Paracidovorax citrulli*]
fig|80869.174.peg.361	T3S	hypothetical protein [*Paracidovorax citrulli*]
fig|80869.174.peg.3632	T3S	pyridoxal kinase PdxY [*Paracidovorax citrulli*]

Prot	Pred	Description
fig|80869.174.peg.3638	T3S	DNA internalization-related competence protein ComEC/Rec2 [*Paracidovorax citrulli*]
fig|80869.174.peg.3646	T3S	uncharacterized protein DUF2132 [*Paracidovorax citrulli*]
fig|80869.174.peg.3679	T3S	CaiB/BaiF CoA transferase family protein [*Paracidovorax citrulli*]
fig|80869.174.peg.368	T3S	ATP-binding protein [*Paracidovorax citrulli*]
fig|80869.174.peg.3707	T3S	protein-L-isoaspartate(D-aspartate) O-methyltransferase [*Paracidovorax citrulli*]
fig|80869.174.peg.3722	T3S	GTPase HflX [*Paracidovorax citrulli*]
fig|80869.174.peg.3747	T3S	DUF2069 domain-containing protein [*Paracidovorax citrulli*]
fig|80869.174.peg.3750	T3S	farnesyl-diphosphate farnesyltransferase [*Paracidovorax citrulli* AAC00-1]
fig|80869.174.peg.378	T3S	YbaN family protein [*Paracidovorax citrulli*]
fig|80869.174.peg.3792	T3S	glycine betaine/L-proline ABC transporter permease ProW [*Paracidovorax citrulli*]
fig|80869.174.peg.3812	T3S	malonate--CoA ligase [*Paracidovorax citrulli*]
fig|80869.174.peg.3820	T3S	heavy metal translocating P-type ATPase [*Paracidovorax citrulli*]
fig|80869.174.peg.3850	T3S	SDR family NAD(P)-dependent oxidoreductase [*Paracidovorax citrulli*]
fig|80869.174.peg.3899	T3S	type 1 glutamine amidotransferase domain-containing protein [*Paracidovorax citrulli*]
fig|80869.174.peg.2784	T4S	MULTISPECIES: DUF3577 domain-containing protein [Pseudomonadota]
fig|80869.174.peg.3262	T4S	Ig domain protein, group 1 domain protein [*Paracidovorax citrulli* AAC00-1]
fig|80869.174.peg.4539	T4S	hypothetical protein [*Paracidovorax citrulli*]
fig|80869.174.peg.4651	T4S	major facilitator superfamily MFS_1 [*Paracidovorax citrulli* AAC00-1]
fig|80869.174.peg.4456	T4S	acyl-CoA thioesterase [*Paracidovorax citrulli*]
fig|80869.174.peg.4807	T4S	STY0301 family protein [*Paracidovorax citrulli*]
fig|80869.174.peg.231	T4S	ABC transporter ATP-binding protein [*Paracidovorax citrulli*]
fig|80869.174.peg.4921	T4S	Ku protein [*Paracidovorax citrulli*]
fig|80869.174.peg.4015	T4S	terminase small subunit [*Paracidovorax citrulli*]
fig|80869.174.peg.3529	T4S	molybdenum cofactor biosynthesis protein MoaE [*Paracidovorax citrulli*]
fig|80869.174.peg.1195	T4S	bacterioferritin [*Paracidovorax citrulli*]
fig|80869.174.peg.3966	T4S	isoaspartyl peptidase/L-asparaginase family protein [*Paracidovorax citrulli*]
fig|80869.174.peg.1093	T4S	abortive infection system antitoxin AbiGi family protein [*Paracidovorax citrulli*]
fig|80869.174.peg.4501	T4S	RNA recognition motif domain-containing protein [*Paracidovorax citrulli*]
fig|80869.174.peg.5103	T4S	hypothetical protein [*Paracidovorax citrulli*]
fig|80869.174.peg.345	T4S	hypothetical protein Aave_3072 [*Paracidovorax citrulli* AAC00-1]
fig|80869.174.peg.58	T4S	DUF1795 domain-containing protein [*Paracidovorax citrulli*]
fig|80869.174.peg.2705	T4S	type III secretion system outer membrane ring subunit SctC [*Paracidovorax citrulli*]
fig|80869.174.peg.4516	T4S	hypothetical protein [*Paracidovorax citrulli*]
fig|80869.174.peg.4957	T4S	MULTISPECIES: amino-acid N-acetyltransferase [Paracidovorax]
fig|80869.174.peg.1973	T4S	DUF924 family protein [*Paracidovorax citrulli*]
fig|80869.174.peg.1029	T4S	hypothetical protein [*Paracidovorax citrulli*]
fig|80869.174.peg.1911	T4S	50S ribosomal protein L11 [*Paracidovorax citrulli*]
fig|80869.174.peg.4924	T4S	hypothetical protein [*Paracidovorax citrulli*]
fig|80869.174.peg.4626	T4S	conserved hypothetical protein [*Paracidovorax citrulli* AAC00-1]
fig|80869.174.peg.878	T4S	PP2C family protein-serine/threonine phosphatase [*Paracidovorax citrulli*]
fig|80869.174.peg.1248	T4S	glutathione-regulated potassium-efflux system protein KefC [*Paracidovorax citrulli*]
fig|80869.174.peg.2287	T4S	hypothetical protein [*Paracidovorax citrulli*]
fig|80869.174.peg.1892	T4S	cryptochrome/photolyase family protein [*Paracidovorax citrulli*]

Prot	Pred	Description
fig|80869.174.peg.3608	T4S	phosphopyruvate hydratase [*Paracidovorax citrulli*]
fig|80869.174.peg.822	T4S	hypothetical protein [*Paracidovorax citrulli*]
fig|80869.174.peg.549	T4S	hypothetical protein [*Paracidovorax citrulli*]
fig|80869.174.peg.4184	T4S	AAA family ATPase [*Paracidovorax citrulli*]
fig|80869.174.peg.1678	T4S	DUF3025 domain-containing protein [*Paracidovorax citrulli*]
fig|80869.174.peg.879	T4S	serine/threonine protein kinase [*Paracidovorax citrulli*]
fig|80869.174.peg.4423	T4S	inositol monophosphatase family protein [*Paracidovorax citrulli*]
fig|80869.174.peg.1603	T4S	N-acetylmuramoyl-L-alanine amidase [*Paracidovorax citrulli*]
fig|80869.174.peg.2835	T4S	AraC family transcriptional regulator [*Paracidovorax citrulli*]
fig|80869.174.peg.3710	T4S	SMC-Scp complex subunit ScpB [*Paracidovorax citrulli*]
fig|80869.174.peg.608	T4S	hypothetical protein [*Paracidovorax citrulli*]
fig|80869.174.peg.2009	T4S	Fic family protein [*Paracidovorax citrulli*]
fig|80869.174.peg.1100	T4S	4-hydroxy-3-methylbut-2-enyl diphosphate reductase [*Paracidovorax citrulli*]
fig|80869.174.peg.668	T4S	SsrA-binding protein SmpB [*Paracidovorax citrulli*]
fig|80869.174.peg.5151	T4S	hypothetical protein [*Paracidovorax citrulli*]
fig|80869.174.peg.2592	T4S	F0F1 ATP synthase subunit gamma [*Paracidovorax citrulli*]
fig|80869.174.peg.4271	T4S	hypothetical protein [*Paracidovorax citrulli*]
fig|80869.174.peg.3737	T4S	hypothetical protein [*Paracidovorax citrulli*]
fig|80869.174.peg.5057	T4S	transglycosylase SLT domain-containing protein [*Paracidovorax citrulli*]
fig|80869.174.peg.1467	T4S	hypothetical protein [*Paracidovorax citrulli*]
fig|80869.174.peg.4033	T4S	hypothetical protein [*Paracidovorax citrulli*]
fig|80869.174.peg.1310	T4S	5'-nucleotidase [*Paracidovorax citrulli*]
fig|80869.174.peg.4742	T4S	hypothetical protein [*Paracidovorax citrulli*]
fig|80869.174.peg.2870	T4S	30S ribosomal protein S4 [*Paracidovorax citrulli*]
fig|80869.174.peg.4966	T4S	adenylate kinase [*Paracidovorax citrulli*]
fig|80869.174.peg.530	T4S	hypothetical protein [*Paracidovorax citrulli*]
fig|80869.174.peg.1698	T4S	hypothetical protein [*Paracidovorax citrulli*]
fig|80869.174.peg.1196	T4S	BON domain-containing protein [*Paracidovorax citrulli*]
fig|80869.174.peg.4943	T4S	MetQ/NlpA family ABC transporter substrate-binding protein [*Paracidovorax citrulli*]
fig|80869.174.peg.1840	T4S	Bug family tripartite tricarboxylate transporter substrate binding protein [*Paracidovorax citrulli*]
fig|80869.174.peg.184	T4S	hypothetical protein [*Paracidovorax citrulli*]
fig|80869.174.peg.4949	T4S	sulfate ABC transporter substrate-binding protein [*Paracidovorax citrulli*]
fig|80869.174.peg.1075	T4S	hypothetical protein [*Paracidovorax citrulli*]
fig|80869.174.peg.1972	T4S	OmpA family protein [*Paracidovorax citrulli*]
fig|80869.174.peg.4050	T4S	glycoside hydrolase family protein [*Paracidovorax citrulli*]
fig|80869.174.peg.2519	T4S	DNA-binding transcriptional LysR family regulator [*Paracidovorax citrulli*]
fig|80869.174.peg.4240	T4S	sigma-54-dependent transcriptional regulator [*Paracidovorax citrulli*]
fig|80869.174.peg.1311	T4S	EF-hand domain-containing protein [*Paracidovorax citrulli*]

*P_citrulli_*DSM17060

Prot	Pred	Description
fig|80869.167.peg.1721	T3S	major facilitator superfamily MFS_1 [*Paracidovorax citrulli* AAC00-1]
fig|80869.167.peg.2473	T3S	Ig domain protein, group 1 domain protein [*Paracidovorax citrulli* AAC00-1]
fig|80869.167.peg.4480	T3S	hypothetical protein [*Paracidovorax citrulli*]

Prot	Pred	Description
fig|80869.167.peg.3897	T3S	acyl-CoA thioesterase [*Paracidovorax citrulli*]
fig|80869.167.peg.2855	T3S	STY0301 family protein [*Paracidovorax citrulli*]
fig|80869.167.peg.644	T3S	ABC transporter ATP-binding protein [*Paracidovorax citrulli*]
fig|80869.167.peg.3400	T3S	Ku protein [*Paracidovorax citrulli*]
fig|80869.167.peg.897	T3S	bacterioferritin [*Paracidovorax citrulli*]
fig|80869.167.peg.4174	T3S	terminase small subunit [*Paracidovorax citrulli*]
fig|80869.167.peg.1001	T3S	polyhydroxyalkanoate synthesis repressor PhaR [*Paracidovorax citrulli*]
fig|80869.167.peg.1003	T3S	type 1 glutamine amidotransferase domain-containing protein [*Paracidovorax citrulli*]
fig|80869.167.peg.104	T3S	GNAT family N-acetyltransferase [*Paracidovorax citrulli*]
fig|80869.167.peg.1049	T3S	SDR family NAD(P)-dependent oxidoreductase [*Paracidovorax citrulli*]
fig|80869.167.peg.1079	T3S	cation-translocating P-type ATPase [*Paracidovorax citrulli*]
fig|80869.167.peg.1084	T3S	malonate--CoA ligase [*Paracidovorax citrulli*]
fig|80869.167.peg.1147	T3S	DUF2069 domain-containing protein [*Paracidovorax citrulli*]
fig|80869.167.peg.1167	T3S	ABC transporter ATP-binding protein [*Paracidovorax citrulli*]
fig|80869.167.peg.1179	T3S	guanine deaminase [*Paracidovorax citrulli*]
fig|80869.167.peg.1193	T3S	tryptophan 2,3-dioxygenase [*Paracidovorax citrulli*]
fig|80869.167.peg.1244	T3S	Phytochrome-like protein cph2 [*Paracidovorax citrulli*]
fig|80869.167.peg.1245	T3S	lysine--tRNA ligase [*Paracidovorax citrulli*]
fig|80869.167.peg.1257	T3S	phosphoribosylanthranilate isomerase [*Paracidovorax citrulli* AAC00-1]
fig|80869.167.peg.1270	T3S	nucleotide exchange factor GrpE [*Paracidovorax citrulli*]
fig|80869.167.peg.1297	T3S	molybdopterin converting factor subunit 1 [*Paracidovorax citrulli*]
fig|80869.167.peg.1310	T3S	hypothetical protein [*Paracidovorax citrulli*]
fig|80869.167.peg.1330	T3S	MULTISPECIES: NADH-quinone oxidoreductase subunit NuoI [Comamonadaceae]
fig|80869.167.peg.1336	T3S	ABC transporter, permease/ATP-binding protein [*Paracidovorax citrulli*]
fig|80869.167.peg.1354	T3S	winged helix-turn-helix transcriptional regulator [*Paracidovorax citrulli*]
fig|80869.167.peg.1363	T3S	N-acetylmuramoyl-L-alanine amidase [*Paracidovorax citrulli*]
fig|80869.167.peg.1404	T3S	putative MFS family arabinose efflux permease [*Paracidovorax citrulli*]
fig|80869.167.peg.1422	T3S	CerR family C-terminal domain-containing protein [*Paracidovorax citrulli*]
fig|80869.167.peg.1464	T3S	putative amino-acid metabolite efflux pump [*Paracidovorax citrulli*]
fig|80869.167.peg.1466	T3S	DUF1800 family protein [*Paracidovorax citrulli*]
fig|80869.167.peg.1481	T3S	MBL fold metallo-hydrolase [*Paracidovorax citrulli*]
fig|80869.167.peg.1494	T3S	response regulator [*Paracidovorax citrulli*]
fig|80869.167.peg.1505	T3S	flagellar basal body protein [*Paracidovorax citrulli*]
fig|80869.167.peg.1506	T3S	LysR family transcriptional regulator [*Paracidovorax citrulli*]
fig|80869.167.peg.1531	T3S	GGDEF domain-containing protein [*Paracidovorax citrulli*]
fig|80869.167.peg.1545	T3S	Cardiolipin synthase B [*Paracidovorax citrulli*]
fig|80869.167.peg.1599	T3S	cyclopropane-fatty-acyl-phospholipid synthase family protein [*Paracidovorax citrulli*]
fig|80869.167.peg.1637	T3S	urease accessory protein UreG [*Paracidovorax citrulli*]
fig|80869.167.peg.1638	T3S	urease accessory protein [*Paracidovorax citrulli*]
fig|80869.167.peg.1698	T3S	DODA-type extradiol aromatic ring-opening family dioxygenase [*Paracidovorax citrulli*]
fig|80869.167.peg.1703	T3S	glutamine--tRNA ligase/YqeY domain fusion protein [*Paracidovorax citrulli*]
fig|80869.167.peg.1714	T3S	alpha/beta hydrolase [*Paracidovorax citrulli*]
fig|80869.167.peg.1717	T3S	Murein DD-endopeptidase MepM [*Paracidovorax citrulli*]
fig|80869.167.peg.1727	T3S	DHH family phosphoesterase [*Paracidovorax citrulli*]
fig|80869.167.peg.1731	T3S	redox-sensitive transcriptional activator SoxR [*Paracidovorax citrulli*]

Prot	Pred	Description
fig|80869.167.peg.1740	T3S	GAF domain-containing sensor histidine kinase [*Paracidovorax citrulli*]
fig|80869.167.peg.1755	T3S	multiple monosaccharide ABC transporter permease [*Paracidovorax citrulli*]
fig|80869.167.peg.1790	T3S	cytochrome o ubiquinol oxidase subunit IV [*Paracidovorax citrulli*]
fig|80869.167.peg.1805	T3S	transcriptional regulator, GntR family [*Paracidovorax citrulli*]
fig|80869.167.peg.1819	T3S	NAD-dependent succinate-semialdehyde dehydrogenase [*Paracidovorax citrulli*]
fig|80869.167.peg.1846	T3S	hypothetical protein [*Paracidovorax citrulli*]
fig|80869.167.peg.1853	T3S	ATP-binding protein [*Paracidovorax citrulli*]
fig|80869.167.peg.1863	T3S	YbaN family protein [*Paracidovorax citrulli*]
fig|80869.167.peg.1877	T3S	RDD domain containing protein [*Paracidovorax citrulli* AAC00-1]
fig|80869.167.peg.1896	T3S	TetR/AcrR family transcriptional regulator [*Paracidovorax citrulli*]
fig|80869.167.peg.1933	T3S	MULTISPECIES: CysB family HTH-type transcriptional regulator [Comamonadaceae]
fig|80869.167.peg.1965	T3S	GNAT family N-acetyltransferase [Paracidovorax avenae]
fig|80869.167.peg.1998	T3S	transcriptional regulator CynR [*Paracidovorax citrulli*]
fig|80869.167.peg.2018	T3S	Cupin 2, conserved barrel domain protein [*Paracidovorax citrulli* AAC00-1]
fig|80869.167.peg.2030	T3S	aromatic ring-hydroxylating dioxygenase subunit alpha [*Paracidovorax citrulli*]
fig|80869.167.peg.2086	T3S	PLxRFG domain-containing protein [*Paracidovorax citrulli*]
fig|80869.167.peg.2135	T3S	ATP synthase F1 subunit epsilon [*Paracidovorax citrulli*]
fig|80869.167.peg.2140	T3S	P1 family peptidase [*Paracidovorax citrulli*]
fig|80869.167.peg.2150	T3S	YitT family protein [*Paracidovorax citrulli*]
fig|80869.167.peg.2154	T3S	MlaD family protein [*Paracidovorax citrulli*]
fig|80869.167.peg.2169	T3S	UDP-3-O-(3-hydroxymyristoyl)glucosamine N-acyltransferase [*Paracidovorax citrulli*]
fig|80869.167.peg.2202	T3S	glutamate--tRNA ligase [*Paracidovorax citrulli*]
fig|80869.167.peg.2203	T3S	DUF1624 domain-containing protein [*Paracidovorax citrulli*]
fig|80869.167.peg.2206	T3S	DUF72 domain-containing protein [*Paracidovorax citrulli*]
fig|80869.167.peg.222	T3S	TRAP transporter small permease [*Paracidovorax citrulli*]
fig|80869.167.peg.2220	T3S	protein of unknown function DUF558 [*Paracidovorax citrulli* AAC00-1]
fig|80869.167.peg.2238	T3S	KdsC family phosphatase [*Paracidovorax citrulli*]
fig|80869.167.peg.2242	T3S	PepSY domain-containing protein [*Paracidovorax citrulli*]
fig|80869.167.peg.2253	T3S	DNA topoisomerase (ATP-hydrolyzing) subunit B [*Paracidovorax citrulli*]
fig|80869.167.peg.2272	T3S	2-isopropylmalate synthase [*Paracidovorax citrulli*]
fig|80869.167.peg.229	T3S	hypothetical protein [*Paracidovorax citrulli*]
fig|80869.167.peg.2301	T3S	ATP-binding cassette domain-containing protein [*Paracidovorax citrulli*]
fig|80869.167.peg.2324	T3S	hypothetical protein [*Paracidovorax citrulli*]
fig|80869.167.peg.2341	T3S	uracil-DNA glycosylase [*Paracidovorax citrulli*]
fig|80869.167.peg.2356	T3S	Glycosyltransferase involved in cell wall bisynthesis [*Paracidovorax citrulli*]
fig|80869.167.peg.2402	T3S	FAD-linked oxidase C-terminal domain-containing protein [*Paracidovorax citrulli*]
fig|80869.167.peg.2415	T3S	transcriptional regulator, AraC family [*Paracidovorax citrulli* AAC00-1]
fig|80869.167.peg.2417	T3S	NarK family nitrate/nitrite MFS transporter [*Paracidovorax citrulli*]
fig|80869.167.peg.242	T3S	septal ring lytic transglycosylase RlpA family protein [*Paracidovorax citrulli*]
fig|80869.167.peg.2443	T3S	peptidoglycan-binding domain-containing protein, partial [*Paracidovorax citrulli*]
fig|80869.167.peg.2467	T3S	penicillin-binding protein 1A [*Paracidovorax citrulli*]
fig|80869.167.peg.2477	T3S	protein of unknown function DUF1415 [*Paracidovorax citrulli* AAC00-1]
fig|80869.167.peg.25	T3S	ABC transporter permease [*Paracidovorax citrulli*]

Prot	Pred	Description
fig|80869.167.peg.2515	T3S	PelD GGDEF domain-containing protein [*Paracidovorax citrulli*]
fig|80869.167.peg.2579	T3S	phosphate ABC transporter permease PstC [*Paracidovorax citrulli*]
fig|80869.167.peg.2584	T3S	Polyphosphate kinase [*Paracidovorax citrulli* AAC00-1]
fig|80869.167.peg.2598	T3S	hypothetical protein [*Paracidovorax citrulli*]
fig|80869.167.peg.2599	T3S	D-serine/D-alanine/glycine transporter [*Paracidovorax citrulli*]
fig|80869.167.peg.2610	T3S	TonB-dependent receptor family protein [*Paracidovorax citrulli*]
fig|80869.167.peg.2652	T3S	gephyrin-like molybdotransferase Glp [*Paracidovorax citrulli*]
fig|80869.167.peg.2653	T3S	GNAT family N-acetyltransferase [*Paracidovorax citrulli*]
fig|80869.167.peg.2666	T3S	translesion DNA synthesis-associated protein ImuA [*Paracidovorax citrulli*]
fig|80869.167.peg.2670	T3S	pseudouridine synthase [*Paracidovorax citrulli*]
fig|80869.167.peg.2700	T3S	pyridoxamine 5'-phosphate oxidase [*Paracidovorax citrulli*]
fig|80869.167.peg.2711	T3S	transcriptional repressor [*Paracidovorax citrulli*]
fig|80869.167.peg.2713	T3S	TetR/AcrR family transcriptional regulator [*Paracidovorax citrulli*]
fig|80869.167.peg.272	T3S	alpha/beta hydrolase [*Paracidovorax citrulli*]
fig|80869.167.peg.2745	T3S	ProQ/FINO family protein [*Paracidovorax citrulli*]
fig|80869.167.peg.2777	T3S	AmpG family muropeptide MFS transporter [*Paracidovorax citrulli*]
fig|80869.167.peg.2780	T3S	DME family drug/metabolite transporter [*Paracidovorax citrulli*]
fig|80869.167.peg.2782	T3S	SLC13 family permease [*Paracidovorax citrulli*]
fig|80869.167.peg.1721	T4S	major facilitator superfamily MFS_1 [*Paracidovorax citrulli* AAC00-1]
fig|80869.167.peg.2473	T4S	Ig domain protein, group 1 domain protein [*Paracidovorax citrulli* AAC00-1]
fig|80869.167.peg.4480	T4S	hypothetical protein [*Paracidovorax citrulli*]
fig|80869.167.peg.3897	T4S	acyl-CoA thioesterase [*Paracidovorax citrulli*]
fig|80869.167.peg.2855	T4S	STY0301 family protein [*Paracidovorax citrulli*]
fig|80869.167.peg.644	T4S	ABC transporter ATP-binding protein [*Paracidovorax citrulli*]
fig|80869.167.peg.3400	T4S	Ku protein [*Paracidovorax citrulli*]
fig|80869.167.peg.897	T4S	bacterioferritin [*Paracidovorax citrulli*]
fig|80869.167.peg.4174	T4S	terminase small subunit [*Paracidovorax citrulli*]
fig|80869.167.peg.1304	T4S	molybdenum cofactor biosynthesis protein MoaE [*Paracidovorax citrulli*]
fig|80869.167.peg.1927	T4S	hypothetical protein [*Paracidovorax citrulli*]
fig|80869.167.peg.989	T4S	isoaspartyl peptidase/L-asparaginase family protein [*Paracidovorax citrulli*]
fig|80869.167.peg.610	T4S	DUF2800 domain-containing protein [*Paracidovorax citrulli*]
fig|80869.167.peg.4233	T4S	RNA recognition motif domain-containing protein [*Paracidovorax citrulli*]
fig|80869.167.peg.1831	T4S	hypothetical protein Aave_3072 [*Paracidovorax citrulli* AAC00-1]
fig|80869.167.peg.2739	T4S	DUF1795 domain-containing protein [*Paracidovorax citrulli*]
fig|80869.167.peg.3367	T4S	MULTISPECIES: amino-acid N-acetyltransferase [Paracidovorax]
fig|80869.167.peg.3301	T4S	DUF924 family protein [*Paracidovorax citrulli*]
fig|80869.167.peg.3935	T4S	50S ribosomal protein L11 [*Paracidovorax citrulli*]
fig|80869.167.peg.3397	T4S	hypothetical protein [*Paracidovorax citrulli*]
fig|80869.167.peg.1747	T4S	conserved hypothetical protein [*Paracidovorax citrulli* AAC00-1]
fig|80869.167.peg.2805	T4S	MULTISPECIES: LysR family transcriptional regulator [Paracidovorax]
fig|80869.167.peg.1592	T4S	PP2C family protein-serine/threonine phosphatase [*Paracidovorax citrulli*]
fig|80869.167.peg.4520	T4S	hypothetical protein [*Paracidovorax citrulli*]
fig|80869.167.peg.3955	T4S	cryptochrome/photolyase family protein [*Paracidovorax citrulli*]
fig|80869.167.peg.3010	T4S	phosphopyruvate hydratase [*Paracidovorax citrulli*]
fig|80869.167.peg.845	T4S	glutathione-regulated potassium-efflux system protein KefC [*Paracidovorax citrulli*]
fig|80869.167.peg.1648	T4S	hypothetical protein [*Paracidovorax citrulli*]

Prot	Pred	Description
fig|80869.167.peg.3115	T4S	hypothetical protein [*Paracidovorax citrulli*]
fig|80869.167.peg.2182	T4S	AAA family ATPase [*Paracidovorax citrulli*]
fig|80869.167.peg.4358	T4S	DUF3025 domain-containing protein [*Paracidovorax citrulli*]
fig|80869.167.peg.1591	T4S	serine/threonine protein kinase [*Paracidovorax citrulli*]
fig|80869.167.peg.4401	T4S	hypothetical protein [*Paracidovorax citrulli*]
fig|80869.167.peg.3998	T4S	inositol monophosphatase family protein [*Paracidovorax citrulli*]
fig|80869.167.peg.38	T4S	N-acetylmuramoyl-L-alanine amidase [*Paracidovorax citrulli*]
fig|80869.167.peg.2348	T4S	AraC family transcriptional regulator [*Paracidovorax citrulli*]
fig|80869.167.peg.4136	T4S	SMC-Scp complex subunit ScpB [*Paracidovorax citrulli*]
fig|80869.167.peg.3460	T4S	hypothetical protein [*Paracidovorax citrulli*]
fig|80869.167.peg.1377	T4S	Fic family protein [*Paracidovorax citrulli*]
fig|80869.167.peg.4419	T4S	4-hydroxy-3-methylbut-2-enyl diphosphate reductase [*Paracidovorax citrulli*]
fig|80869.167.peg.4095	T4S	SsrA-binding protein SmpB [*Paracidovorax citrulli*]
fig|80869.167.peg.173	T4S	hypothetical protein [*Paracidovorax citrulli*]
fig|80869.167.peg.3816	T4S	F0F1 ATP synthase subunit gamma [*Paracidovorax citrulli*]
fig|80869.167.peg.611	T4S	DUF2815 family protein [*Paracidovorax citrulli*]
fig|80869.167.peg.1157	T4S	hypothetical protein [*Paracidovorax citrulli*]
fig|80869.167.peg.2555	T4S	transglycosylase SLT domain-containing protein [*Paracidovorax citrulli*]
fig|80869.167.peg.2327	T4S	type III secretion system outer membrane ring subunit SctC [*Paracidovorax citrulli*]
fig|80869.167.peg.2080	T4S	hypothetical protein [*Paracidovorax citrulli*]
fig|80869.167.peg.1039	T4S	XopE/AvrPphe family type III secretion system effector [*Paracidovorax citrulli*]
fig|80869.167.peg.783	T4S	5'-nucleotidase [*Paracidovorax citrulli*]
fig|80869.167.peg.2383	T4S	30S ribosomal protein S4 [*Paracidovorax citrulli*]
fig|80869.167.peg.2648	T4S	adenylate kinase [*Paracidovorax citrulli*]
fig|80869.167.peg.3134	T4S	hypothetical protein [*Paracidovorax citrulli*]
fig|80869.167.peg.3086	T4S	hypothetical protein [*Paracidovorax citrulli*]
fig|80869.167.peg.1622	T4S	type IV pilin protein [*Paracidovorax citrulli*]
fig|80869.167.peg.896	T4S	BON domain-containing protein [*Paracidovorax citrulli*]
fig|80869.167.peg.3381	T4S	MetQ/NlpA family ABC transporter substrate-binding protein [*Paracidovorax citrulli*]
fig|80869.167.peg.4046	T4S	Bug family tripartite tricarboxylate transporter substrate binding protein [*Paracidovorax citrulli*]
fig|80869.167.peg.2176	T4S	Bug family tripartite tricarboxylate transporter substrate binding protein [*Paracidovorax citrulli*]
fig|80869.167.peg.3715	T4S	P-type conjugative transfer protein TrbL, partial [*Paracidovorax citrulli*]
fig|80869.167.peg.3375	T4S	sulfate ABC transporter substrate-binding protein [*Paracidovorax citrulli*]
fig|80869.167.peg.4637	T4S	hypothetical protein [*Paracidovorax citrulli*]
fig|80869.167.peg.3302	T4S	OmpA family protein [*Paracidovorax citrulli*]
fig|80869.167.peg.2094	T4S	glycoside hydrolase family protein [*Paracidovorax citrulli*]
fig|80869.167.peg.3193	T4S	sigma-54-dependent transcriptional regulator [*Paracidovorax citrulli*]
fig|80869.167.peg.782	T4S	EF-hand domain-containing protein [*Paracidovorax citrulli*]

*P_citrulli*_EP

Prot	Pred	Description
fig|80869.175.peg.5265	T3S	type VI secretion system membrane subunit TssM [*Paracidovorax citrulli*]
fig|80869.175.peg.551	T3S	methylmalonyl Co-A mutase-associated GTPase MeaB [*Paracidovorax citrulli*]

Prot	Pred	Description
fig|80869.175.peg.6281	T3S	hypothetical protein Aave_3072 [*Paracidovorax citrulli* AAC00-1]
fig|80869.175.peg.5968	T3S	MAG: hypothetical protein BECKLPF1236B_GA0070989_101629 [Candidatus Kentron sp. LPFa]
fig|80869.175.peg.3692	T3S	hypothetical protein C8E08_2412 [*Paracidovorax citrulli*]
fig|80869.175.peg.675	T3S	hypothetical protein [*Paracidovorax citrulli*]
fig|80869.175.peg.1725	T3S	GGDEF domain-containing protein [*Paracidovorax citrulli*]
fig|80869.175.peg.5583	T3S	hypothetical protein [*Paracidovorax citrulli*]
fig|80869.175.peg.6435	T3S	ABC transporter ATP-binding protein [*Paracidovorax citrulli*]
fig|80869.175.peg.1038	T3S	hypothetical protein [*Paracidovorax citrulli*]
fig|80869.175.peg.862	T3S	DUF1501 domain-containing protein [*Paracidovorax citrulli*]
fig|80869.175.peg.5557	T3S	terminase small subunit [*Paracidovorax citrulli*]
fig|80869.175.peg.1	T3S	hypothetical protein [*Paracidovorax citrulli*]
fig|80869.175.peg.1068	T3S	MULTISPECIES: hypothetical protein [Paracidovorax]
fig|80869.175.peg.1070	T3S	DUF2169 domain-containing protein [*Paracidovorax citrulli*]
fig|80869.175.peg.1089	T3S	carboxyl transferase domain-containing protein [*Paracidovorax citrulli*]
fig|80869.175.peg.1139	T3S	LysR substrate-binding domain-containing protein [*Paracidovorax citrulli*]
fig|80869.175.peg.1145	T3S	succinate-semialdehyde dehydrogenase/glutarate-semialdehyde dehydrogenase [*Paracidovorax citrulli*]
fig|80869.175.peg.1188	T3S	MAG: hypothetical protein GAK34_03682 [Delftia tsuruhatensis]
fig|80869.175.peg.1223	T3S	TOBE domain-containing protein [*Paracidovorax citrulli*]
fig|80869.175.peg.1228	T3S	BCCT family transporter [*Paracidovorax citrulli*]
fig|80869.175.peg.1235	T3S	Uncharacterised protein [Bordetella pertussis]
fig|80869.175.peg.125	T3S	DUF3299 domain-containing protein [*Paracidovorax citrulli*]
fig|80869.175.peg.1252	T3S	exodeoxyribonuclease VII, small subunit [*Paracidovorax citrulli* AAC00-1]
fig|80869.175.peg.1258	T3S	SfnB family sulfur acquisition oxidoreductase [*Paracidovorax citrulli*]
fig|80869.175.peg.1274	T3S	uroporphyrinogen-III C-methyltransferase [*Paracidovorax citrulli*]
fig|80869.175.peg.1294	T3S	hypothetical protein [*Paracidovorax citrulli*]
fig|80869.175.peg.1304	T3S	CDP-6-deoxy-delta-3,4-glucoseen reductase [*Paracidovorax citrulli*]
fig|80869.175.peg.1334	T3S	Ig-like domain-containing alpha-2-macroglobulin family protein [*Paracidovorax citrulli*]
fig|80869.175.peg.1344	T3S	argininosuccinate lyase [*Paracidovorax citrulli*]
fig|80869.175.peg.137	T3S	pyridoxamine 5'-phosphate oxidase [*Paracidovorax citrulli*]
fig|80869.175.peg.141	T3S	hypothetical protein [*Paracidovorax citrulli*]
fig|80869.175.peg.1475	T3S	hypothetical protein Aave_3452 [*Paracidovorax citrulli* AAC00-1]
fig|80869.175.peg.1490	T3S	tautomerase family protein [*Paracidovorax citrulli*]
fig|80869.175.peg.1523	T3S	diacylglycerol/lipid kinase family protein [*Paracidovorax citrulli*]
fig|80869.175.peg.1555	T3S	putative hydro-lyase [*Paracidovorax citrulli*]
fig|80869.175.peg.1575	T3S	urease accessory protein [*Paracidovorax citrulli*]
fig|80869.175.peg.1576	T3S	urease accessory protein UreG [*Paracidovorax citrulli*]
fig|80869.175.peg.1607	T3S	MAG: hypothetical protein GAK39_05929 [Variovorax sp.]
fig|80869.175.peg.1625	T3S	SAM-dependent methyltransferase [*Paracidovorax citrulli*]
fig|80869.175.peg.1657	T3S	hypothetical protein [Acidovorax sp. GBBC 3334]
fig|80869.175.peg.1705	T3S	cardiolipin synthase [*Paracidovorax citrulli*]
fig|80869.175.peg.173	T3S	hypothetical protein [*Paracidovorax citrulli*]
fig|80869.175.peg.1797	T3S	MFS transporter [*Paracidovorax citrulli*]
fig|80869.175.peg.1828	T3S	hypothetical protein [*Paracidovorax citrulli*]
fig|80869.175.peg.1905	T3S	hypothetical protein BMETH_284_2 [methanotrophic bacterial endosymbiont of Bathymodiolus sp.]
fig|80869.175.peg.1911	T3S	esterase-like activity of phytase family protein [*Paracidovorax citrulli*]

Prot	Pred	Description
fig|80869.175.peg.1955	T3S	hypothetical protein [*Paracidovorax citrulli*]
fig|80869.175.peg.1982	T3S	YitT family protein [*Paracidovorax citrulli*]
fig|80869.175.peg.2029	T3S	signal recognition particle-docking protein FtsY [*Paracidovorax citrulli*]
fig|80869.175.peg.2041	T3S	MATE family efflux transporter [*Paracidovorax citrulli*]
fig|80869.175.peg.2060	T3S	Uncharacterised protein [Mycobacterium tuberculosis]
fig|80869.175.peg.21	T3S	acyl-CoA dehydrogenase [*Paracidovorax citrulli*]
fig|80869.175.peg.2123	T3S	type III secretion system chaperone [*Paracidovorax citrulli*]
fig|80869.175.peg.2133	T3S	translocation/assembly module TamB domain-containing protein [*Paracidovorax citrulli*]
fig|80869.175.peg.2179	T3S	hypothetical protein [*Paracidovorax citrulli*]
fig|80869.175.peg.2198	T3S	ABC transporter permease subunit [*Paracidovorax citrulli*]
fig|80869.175.peg.2199	T3S	ABC transporter permease [*Paracidovorax citrulli*]
fig|80869.175.peg.2217	T3S	(2Fe-2S)-binding protein [*Paracidovorax citrulli*]
fig|80869.175.peg.2218	T3S	molybdenum cofactor cytidylyltransferase [*Paracidovorax citrulli*]
fig|80869.175.peg.2223	T3S	hypothetical protein [*Paracidovorax citrulli*]
fig|80869.175.peg.2251	T3S	murein DD-endopeptidase MepM/ murein hydrolase activator NlpD [*Paracidovorax citrulli*]
fig|80869.175.peg.233	T3S	hypothetical protein [*Paracidovorax citrulli*]
fig|80869.175.peg.2492	T3S	dTDP-4-dehydrorhamnose 3,5-epimerase [*Paracidovorax citrulli*]
fig|80869.175.peg.2502	T3S	rhodanese-like domain-containing protein [*Paracidovorax citrulli*]
fig|80869.175.peg.2513	T3S	c-type cytochrome [*Paracidovorax citrulli*]
fig|80869.175.peg.2525	T3S	GNAT family N-acetyltransferase [*Paracidovorax citrulli*]
fig|80869.175.peg.2539	T3S	ABC transporter permease [*Paracidovorax citrulli*]
fig|80869.175.peg.2544	T3S	Metallo-beta-lactamase superfamily protein [*Paracidovorax citrulli*]
fig|80869.175.peg.2585	T3S	hypothetical protein [*Paracidovorax citrulli*]
fig|80869.175.peg.266	T3S	type IV pilus twitching motility protein PilT [*Paracidovorax citrulli*]
fig|80869.175.peg.2677	T3S	NAD(P)/FAD-dependent oxidoreductase [*Paracidovorax citrulli*]
fig|80869.175.peg.2729	T3S	porin [*Paracidovorax citrulli*]
fig|80869.175.peg.2747	T3S	AraC family transcriptional regulator [*Paracidovorax citrulli*]
fig|80869.175.peg.2774	T3S	CheR family methyltransferase [*Paracidovorax citrulli*]
fig|80869.175.peg.2777	T3S	chemotaxis protein CheW [Paracidovorax cattleyae]
fig|80869.175.peg.2794	T3S	flagellar protein export ATPase FliI [*Paracidovorax citrulli*]
fig|80869.175.peg.2805	T3S	flagellin hook IN motif-containing protein, partial [Acidovorax sp. PRC11]
fig|80869.175.peg.2809	T3S	conserved hypothetical protein [*Paracidovorax citrulli* AAC00-1]
fig|80869.175.peg.2828	T3S	flagellar hook assembly protein FlgD [*Paracidovorax citrulli*]
fig|80869.175.peg.2869	T3S	hypothetical protein AJ61_04619 [Pseudomonas aeruginosa 3574]
fig|80869.175.peg.2882	T3S	uroporphyrinogen decarboxylase [*Paracidovorax citrulli*]
fig|80869.175.peg.289	T3S	DEAD/DEAH box helicase [*Paracidovorax citrulli*]
fig|80869.175.peg.2931	T3S	MULTISPECIES: PhaM family polyhydroxyalkanoate granule multifunctional regulatory protein [unclassified Acidovorax]
fig|80869.175.peg.299	T3S	hypothetical protein CQB05_01060 [*Paracidovorax citrulli*]
fig|80869.175.peg.3018	T3S	D-alanyl-D-alanine carboxypeptidase DacC [*Paracidovorax citrulli*]
fig|80869.175.peg.3049	T3S	gamma-glutamyl-gamma-aminobutyrate hydrolase family protein [*Paracidovorax citrulli*]
fig|80869.175.peg.3061	T3S	pirin family protein [*Paracidovorax citrulli*]
fig|80869.175.peg.3067	T3S	hypothetical protein [*Paracidovorax citrulli*]
fig|80869.175.peg.3216	T3S	MBL fold metallo-hydrolase [*Paracidovorax citrulli*]
fig|80869.175.peg.329	T3S	pseudouridine synthase [*Paracidovorax citrulli*]
fig|80869.175.peg.3294	T3S	LPS export ABC transporter periplasmic protein LptC [*Paracidovorax citrulli*]

Prot	Pred	Description
fig|80869.175.peg.3300	T3S	PepSY domain-containing protein [*Paracidovorax citrulli*]
fig|80869.175.peg.3329	T3S	hypothetical protein [*Paracidovorax citrulli*]
fig|80869.175.peg.3376	T3S	ATP-binding cassette domain-containing protein [*Paracidovorax citrulli*]
fig|80869.175.peg.340	T3S	Crp/Fnr family transcriptional regulator [*Paracidovorax citrulli*]
fig|80869.175.peg.3404	T3S	precorrin-2 C(20)-methyltransferase [*Paracidovorax citrulli*]
fig|80869.175.peg.3422	T3S	HdeA family protein [*Paracidovorax citrulli*]
fig|80869.175.peg.3440	T3S	hotdog fold thioesterase [*Paracidovorax citrulli*]
fig|80869.175.peg.3445	T3S	hypothetical protein [*Paracidovorax citrulli*]
fig|80869.175.peg.3455	T3S	MBL fold metallo-hydrolase [*Paracidovorax citrulli*]
fig|80869.175.peg.3504	T3S	hypothetical protein [*Paracidovorax citrulli*]
fig|80869.175.peg.3514	T3S	hypothetical protein Tamer19_19740 [Cupriavidus sp. TA19]
fig|80869.175.peg.352	T3S	gephyrin-like molybdotransferase Glp [*Paracidovorax citrulli*]
fig|80869.175.peg.3536	T3S	aromatic ring-hydroxylating dioxygenase subunit alpha [*Paracidovorax citrulli*]
fig|80869.175.peg.3543	T3S	hypothetical protein [*Paracidovorax citrulli*]
fig|80869.175.peg.3577	T3S	transcriptional regulator CynR [*Paracidovorax citrulli*]
fig|80869.175.peg.359	T3S	zonular occludens toxin domain-containing protein [*Paracidovorax citrulli*]
fig|80869.175.peg.362	T3S	hypothetical protein [*Paracidovorax citrulli*]
fig|80869.175.peg.3696	T3S	DME family drug/metabolite transporter [*Paracidovorax citrulli*]
fig|80869.175.peg.3699	T3S	SLC13 family permease [*Paracidovorax citrulli*]
fig|80869.175.peg.3720	T3S	penicillin-binding protein 2 [*Paracidovorax citrulli*]
fig|80869.175.peg.3752	T3S	DHA2 family efflux MFS transporter permease subunit [*Paracidovorax citrulli*]
fig|80869.175.peg.3818	T3S	TPM domain-containing protein [*Paracidovorax citrulli*]
fig|80869.175.peg.3846	T3S	tyrosine-type recombinase/integrase [*Paracidovorax citrulli*]
fig|80869.175.peg.3879	T3S	hypothetical protein [*Paracidovorax citrulli*]
fig|80869.175.peg.3927	T3S	hypothetical protein [*Paracidovorax citrulli*]
fig|80869.175.peg.394	T3S	hypothetical protein [*Paracidovorax citrulli*]
fig|80869.175.peg.3949	T3S	hypothetical protein [*Paracidovorax citrulli*]
fig|80869.175.peg.4009	T3S	MULTISPECIES: hypothetical protein [Pseudomonadota]
fig|80869.175.peg.4023	T3S	MULTISPECIES: DUF3577 domain-containing protein [Pseudomonadota]
fig|80869.175.peg.4024	T3S	DUF3577 domain-containing protein [*Paracidovorax citrulli*]
fig|80869.175.peg.4069	T3S	MULTISPECIES: RidA family protein [Pseudomonadota]
fig|80869.175.peg.4105	T3S	glycosyltransferase involved in cell wall biosynthesis [*Paracidovorax citrulli*]
fig|80869.175.peg.4161	T3S	FAD-linked oxidase C-terminal domain-containing protein [*Paracidovorax citrulli*]
fig|80869.175.peg.4179	T3S	transcriptional regulator, AraC family [*Paracidovorax citrulli* AAC00-1]
fig|80869.175.peg.4182	T3S	hypothetical protein ALP65_01559 [Pseudomonas aeruginosa]
fig|80869.175.peg.4195	T3S	hemerythrin domain-containing protein [*Paracidovorax citrulli*]
fig|80869.175.peg.4234	T3S	FMN-dependent NADH-azoreductase [*Paracidovorax citrulli*]
fig|80869.175.peg.4256	T3S	LysR family transcriptional regulator [*Paracidovorax citrulli*]
fig|80869.175.peg.426	T3S	D-serine/D-alanine/glycine transporter [*Paracidovorax citrulli*]
fig|80869.175.peg.4322	T3S	putative zinc protease protein [*Paracidovorax citrulli* AAC00-1]
fig|80869.175.peg.4323	T3S	DNA-3-methyladenine glycosylase I [Paracidovorax oryzae]
fig|80869.175.peg.4335	T3S	hypothetical protein Y695_00992 [Hydrogenophaga sp. T4]
fig|80869.175.peg.4355	T3S	chromate efflux transporter [*Paracidovorax citrulli*]
fig|80869.175.peg.4399	T3S	MULTISPECIES: cell division protein FtsZ [Paracidovorax]
fig|80869.175.peg.4430	T3S	3-deoxy-7-phosphoheptulonate synthase [*Paracidovorax citrulli*]
fig|80869.175.peg.4445	T3S	ribonucleoside-diphosphate reductase subunit alpha [*Paracidovorax citrulli*]

Prot	Pred	Description
fig|80869.175.peg.4448	T3S	sensor histidine kinase [*Paracidovorax citrulli*]
fig|80869.175.peg.4484	T3S	hydroxymethylpyrimidine/phosphomethylpyrimidine kinase [*Paracidovorax citrulli*]
fig|80869.175.peg.4494	T3S	Holliday junction resolvase RuvX [*Paracidovorax citrulli*]
fig|80869.175.peg.4529	T3S	ABC transporter ATP-binding protein [*Paracidovorax citrulli*]
fig|80869.175.peg.4576	T3S	NCS1 family nucleobase:cation symporter-1 [*Paracidovorax citrulli*]
fig|80869.175.peg.4580	T3S	peptidoglycan-binding domain-containing protein [*Paracidovorax citrulli*]
fig|80869.175.peg.4582	T3S	hypothetical protein Aave_0973 [*Paracidovorax citrulli* AAC00-1]
fig|80869.175.peg.4612	T3S	penicillin-binding protein 1A [*Paracidovorax citrulli*]
fig|80869.175.peg.4619	T3S	Ig domain protein, group 1 domain protein [*Paracidovorax citrulli* AAC00-1]
fig|80869.175.peg.4625	T3S	protein of unknown function DUF1415 [*Paracidovorax citrulli* AAC00-1]
fig|80869.175.peg.4666	T3S	tetratricopeptide repeat protein [*Paracidovorax citrulli*]
fig|80869.175.peg.4736	T3S	GntR family transcriptional regulator [*Paracidovorax citrulli*]
fig|80869.175.peg.5265	T4S	type VI secretion system membrane subunit TssM [*Paracidovorax citrulli*]
fig|80869.175.peg.551	T4S	methylmalonyl Co-A mutase-associated GTPase MeaB [*Paracidovorax citrulli*]
fig|80869.175.peg.6281	T4S	hypothetical protein Aave_3072 [*Paracidovorax citrulli* AAC00-1]
fig|80869.175.peg.5968	T4S	MAG: hypothetical protein BECKLPF1236B_GA0070989_101629 [Candidatus Kentron sp. LPFa]
fig|80869.175.peg.3692	T4S	hypothetical protein C8E08_2412 [*Paracidovorax citrulli*]
fig|80869.175.peg.675	T4S	hypothetical protein [*Paracidovorax citrulli*]
fig|80869.175.peg.1725	T4S	GGDEF domain-containing protein [*Paracidovorax citrulli*]
fig|80869.175.peg.5583	T4S	hypothetical protein [*Paracidovorax citrulli*]
fig|80869.175.peg.6435	T4S	ABC transporter ATP-binding protein [*Paracidovorax citrulli*]
fig|80869.175.peg.1038	T4S	hypothetical protein [*Paracidovorax citrulli*]
fig|80869.175.peg.862	T4S	DUF1501 domain-containing protein [*Paracidovorax citrulli*]
fig|80869.175.peg.5557	T4S	terminase small subunit [*Paracidovorax citrulli*]
fig|80869.175.peg.2021	T4S	bacterioferritin [*Paracidovorax citrulli*]
fig|80869.175.peg.3997	T4S	hypothetical protein [*Paracidovorax citrulli*]
fig|80869.175.peg.3456	T4S	nitrate/sulfonate/bicarbonate ABC transporter ATP-binding protein [*Paracidovorax citrulli*]
fig|80869.175.peg.1807	T4S	MULTISPECIES: RHS repeat-associated core domain-containing protein [unclassified Acidovorax]
fig|80869.175.peg.4239	T4S	IS3-like element ISAav4 family transposase [*Paracidovorax citrulli*]
fig|80869.175.peg.4240	T4S	IS3-like element ISAav4 family transposase [*Paracidovorax citrulli*]
fig|80869.175.peg.5218	T4S	hypothetical protein [Pseudomonadota bacterium]
fig|80869.175.peg.1052	T4S	RNA recognition motif domain-containing protein [*Paracidovorax citrulli*]
fig|80869.175.peg.3778	T4S	30S ribosomal protein S3 [*Paracidovorax citrulli*]
fig|80869.175.peg.2079	T4S	MULTISPECIES: LysE family transporter [Paracidovorax]
fig|80869.175.peg.373	T4S	transglycosylase SLT domain-containing protein [*Paracidovorax citrulli*]
fig|80869.175.peg.606	T4S	transglycosylase domain-containing protein [*Paracidovorax citrulli*]
fig|80869.175.peg.298	T4S	hypothetical protein [*Paracidovorax citrulli*]
fig|80869.175.peg.1284	T4S	valyl-tRNA synthetase [*Paracidovorax citrulli* AAC00-1]
fig|80869.175.peg.2159	T4S	5'-nucleotidase [*Paracidovorax citrulli*]
fig|80869.175.peg.4620	T4S	Ig-like domain-containing protein [*Paracidovorax citrulli*]
fig|80869.175.peg.1923	T4S	type VI secretion system Vgr family protein [*Paracidovorax citrulli*]
fig|80869.175.peg.2258	T4S	MULTISPECIES: 50S ribosomal protein L13 [Comamonadaceae]
fig|80869.175.peg.482	T4S	adenylate kinase [*Paracidovorax citrulli*]
fig|80869.175.peg.4936	T4S	Heat shock protein 70 [Hydrogenophaga sp. T4]
fig|80869.175.peg.5854	T4S	translation initiation factor IF-2 [Paracidovorax avenae]

Prot	Pred	Description
fig|80869.175.peg.5566	T4S	hypothetical protein [*Paracidovorax citrulli*]
fig|80869.175.peg.45	T4S	threonine--tRNA ligase [*Paracidovorax citrulli*]
fig|80869.175.peg.104	T4S	hypothetical protein AWB67_07665 [Caballeronia terrestris]
fig|80869.175.peg.4032	T4S	GTPase [Paracidovorax avenae]
fig|80869.175.peg.494	T4S	MULTISPECIES: amino-acid N-acetyltransferase [Paracidovorax]
fig|80869.175.peg.1026	T4S	histone deacetylase family protein [Paracidovorax avenae]
fig|80869.175.peg.3044	T4S	DUF924 family protein [*Paracidovorax citrulli*]
fig|80869.175.peg.6416	T4S	malto-oligosyltrehalose trehalohydrolase [*Paracidovorax citrulli*]
fig|80869.175.peg.3943	T4S	type III secretion system outer membrane ring subunit SctC [*Paracidovorax citrulli*]
fig|80869.175.peg.584	T4S	polyhydroxyalkanoate depolymerase [*Paracidovorax citrulli*]
fig|80869.175.peg.2970	T4S	50S ribosomal protein L11 [*Paracidovorax citrulli*]
fig|80869.175.peg.3494	T4S	LysR family transcriptional regulator [*Paracidovorax citrulli*]
fig|80869.175.peg.6502	T4S	DDE-type integrase/transposase/recombinase [*Paracidovorax citrulli*]
fig|80869.175.peg.4337	T4S	branched-chain amino acid ABC transporter permease [*Paracidovorax citrulli*]
fig|80869.175.peg.2903	T4S	hypothetical protein [*Paracidovorax citrulli*]
fig|80869.175.peg.4083	T4S	anthranilate/aminodeoxychorismate synthase component II [*Paracidovorax citrulli*]
fig|80869.175.peg.6440	T4S	urocanate hydratase [*Paracidovorax citrulli*]
fig|80869.175.peg.4016	T4S	MULTISPECIES: hypothetical protein [Pseudomonadota]
fig|80869.175.peg.3065	T4S	sulfatase [*Paracidovorax citrulli* AAC00-1]
fig|80869.175.peg.3045	T4S	putative DNA modification/repair radical SAM protein [*Paracidovorax citrulli*]
fig|80869.175.peg.1635	T4S	PP2C family protein-serine/threonine phosphatase [*Paracidovorax citrulli*]
fig|80869.175.peg.1006	T4S	hypothetical protein [*Paracidovorax citrulli*]
fig|80869.175.peg.2697	T4S	DUF3025 domain-containing protein [*Paracidovorax citrulli*]
fig|80869.175.peg.423	T4S	carbamoyl-phosphate synthase large subunit [*Paracidovorax citrulli*]
fig|80869.175.peg.229	T4S	hypothetical protein [*Paracidovorax citrulli*]
fig|80869.175.peg.3423	T4S	hypothetical protein [*Paracidovorax citrulli*]
fig|80869.175.peg.4890	T4S	GTPase Era [*Paracidovorax citrulli*]
fig|80869.175.peg.68	T4S	chromosome segregation protein SMC [Paracidovorax avenae]
fig|80869.175.peg.1565	T4S	hypothetical protein [*Paracidovorax citrulli*]
fig|80869.175.peg.3207	T4S	ubiquitin family protein [*Paracidovorax citrulli*]
fig|80869.175.peg.34	T4S	SDR family oxidoreductase [*Paracidovorax citrulli*]
fig|80869.175.peg.933	T4S	T6SS phospholipase effector Tle1-like catalytic domain-containing protein [*Paracidovorax citrulli*]
fig|80869.175.peg.1636	T4S	protein kinase [*Paracidovorax citrulli*]
fig|80869.175.peg.1163	T4S	inositol monophosphatase family protein [*Paracidovorax citrulli*]
fig|80869.175.peg.5248	T4S	trigger factor [*Paracidovorax citrulli*]
fig|80869.175.peg.4326	T4S	class I SAM-dependent methyltransferase [*Paracidovorax citrulli*]
fig|80869.175.peg.1553	T4S	5-oxoprolinase subunit PxpB [*Paracidovorax citrulli*]
fig|80869.175.peg.6414	T4S	malto-oligosyltrehalose synthase [*Paracidovorax citrulli*]
fig|80869.175.peg.2898	T4S	conserved hypothetical protein [*Paracidovorax citrulli* AAC00-1]
fig|80869.175.peg.6383	T4S	D-(-)-3-hydroxybutyrate oligomer hydrolase [*Paracidovorax citrulli*]
fig|80869.175.peg.5580	T4S	hypothetical protein [*Paracidovorax citrulli*]
fig|80869.175.peg.6084	T4S	molybdopterin-dependent oxidoreductase [*Paracidovorax citrulli*]
fig|80869.175.peg.5939	T4S	hypothetical protein [*Paracidovorax citrulli*]
fig|80869.175.peg.5855	T4S	translation initiation factor IF-2 [*Paracidovorax citrulli*]
fig|80869.175.peg.2940	T4S	cryptochrome/photolyase family protein [*Paracidovorax citrulli*]

Prot	Pred	Description
fig|80869.175.peg.4397	T4S	D-alanine--D-alanine ligase [*Paracidovorax citrulli*]
fig|80869.175.peg.3083	T4S	Fic family protein [*Paracidovorax citrulli*]
fig|80869.175.peg.4953	T4S	gephyrin-like molybdotransferase Glp [*Paracidovorax citrulli*]
fig|80869.175.peg.776	T4S	terminase large subunit domain-containing protein [*Paracidovorax citrulli*]
fig|80869.175.peg.2716	T4S	hypothetical protein [*Paracidovorax citrulli*]
fig|80869.175.peg.1771	T4S	protein of unknown function DUF1342 [Paracidovorax avenae ATCC 19860]
fig|80869.175.peg.3070	T4S	VOC family protein [*Paracidovorax citrulli*]
fig|80869.175.peg.3016	T4S	SGNH/GDSL hydrolase family protein [*Paracidovorax citrulli*]
fig|80869.175.peg.5594	T4S	hypothetical protein [*Paracidovorax citrulli*]
fig|80869.175.peg.6097	T4S	MBL fold metallo-hydrolase [*Paracidovorax citrulli*]
fig|80869.175.peg.2598	T4S	N-acetylmuramoyl-L-alanine amidase [*Paracidovorax citrulli*]
fig|80869.175.peg.6498	T4S	hypothetical protein [*Paracidovorax citrulli*]
fig|80869.175.peg.1365	T4S	hypothetical protein [*Paracidovorax citrulli*]
fig|80869.175.peg.5221	T4S	hypothetical protein [*Paracidovorax citrulli*]
fig|80869.175.peg.2390	T4S	hypothetical protein [*Paracidovorax citrulli*]
fig|80869.175.peg.2471	T4S	type II secretion system secretin GspD [*Paracidovorax citrulli*]
fig|80869.175.peg.760	T4S	hypothetical protein [*Paracidovorax citrulli*]
fig|80869.175.peg.857	T4S	MFS transporter [*Paracidovorax citrulli*]
fig|80869.175.peg.6436	T4S	ABC transporter ATP-binding protein [*Paracidovorax citrulli*]
fig|80869.175.peg.4962	T4S	AAA family ATPase, partial [Paracidovorax cattleyae]
fig|80869.175.peg.6357	T4S	GGDEF domain-containing protein [Paracidovorax avenae]
fig|80869.175.peg.5494	T4S	elongation factor P maturation arginine rhamnosyltransferase EarP [*Paracidovorax citrulli*]
fig|80869.175.peg.3313	T4S	DNA polymerase III subunit beta [Acidovorax temperans]
fig|80869.175.peg.5539	T4S	DNA methylase [*Paracidovorax citrulli*]
fig|80869.175.peg.2190	T4S	CHASE3 domain-containing protein, partial [Paracidovorax avenae]
fig|80869.175.peg.6500	T4S	AAA family ATPase [*Paracidovorax citrulli*]
fig|80869.175.peg.2593	T4S	D-aminoacyl-tRNA deacylase [*Paracidovorax citrulli*]
fig|80869.175.peg.4793	T4S	penicillin acylase family protein [*Paracidovorax citrulli*]
fig|80869.175.peg.2022	T4S	BON domain-containing protein [*Paracidovorax citrulli*]
fig|80869.175.peg.511	T4S	MetQ/NlpA family ABC transporter substrate-binding protein [*Paracidovorax citrulli*]
fig|80869.175.peg.6150	T4S	LPS export ABC transporter permease LptG [*Paracidovorax citrulli*]
fig|80869.175.peg.4421	T4S	MAG: hypothetical protein GAK34_02765 [Delftia tsuruhatensis]
fig|80869.175.peg.3891	T4S	thiamine-phosphate kinase [*Paracidovorax citrulli*]
fig|80869.175.peg.6277	T4S	3-hydroxyacyl-CoA dehydrogenase, partial [Paracidovorax avenae]
fig|80869.175.peg.1874	T4S	hypothetical protein [*Paracidovorax citrulli*]
fig|80869.175.peg.5266	T4S	type VI secretion system membrane subunit TssM [*Paracidovorax citrulli*]
fig|80869.175.peg.3917	T4S	type III secretion system export apparatus subunit SctV [*Paracidovorax citrulli*]
fig|80869.175.peg.3043	T4S	OmpA family protein [*Paracidovorax citrulli*]
fig|80869.175.peg.2581	T4S	DUF4136 domain-containing protein [Paracidovorax avenae]
fig|80869.175.peg.3390	T4S	hypothetical protein [*Paracidovorax citrulli*]
fig|80869.175.peg.587	T4S	methyl-accepting chemotaxis protein [*Paracidovorax citrulli*]

*P_citrulli*_HPP21_3_3B

Prot	Pred	Description
fig|80869.176.peg.1770	T3S	hypothetical protein [*Paracidovorax citrulli*]
fig|80869.176.peg.1882	T3S	major facilitator superfamily MFS_1 [*Paracidovorax citrulli* AAC00-1]
fig|80869.176.peg.3722	T3S	Ig domain protein, group 1 domain protein [*Paracidovorax citrulli* AAC00-1]
fig|80869.176.peg.1690	T3S	acyl-CoA thioesterase [*Paracidovorax citrulli*]
fig|80869.176.peg.2285	T3S	STY0301 family protein [*Paracidovorax citrulli*]
fig|80869.176.peg.2375	T3S	ABC transporter ATP-binding protein [*Paracidovorax citrulli*]
fig|80869.176.peg.2178	T3S	Ku protein [*Paracidovorax citrulli*]
fig|80869.176.peg.981	T3S	bacterioferritin [*Paracidovorax citrulli*]
fig|80869.176.peg.1010	T3S	hypothetical protein [*Paracidovorax citrulli*]
fig|80869.176.peg.1011	T3S	YitT family protein [*Paracidovorax citrulli*]
fig|80869.176.peg.1023	T3S	hypothetical protein [*Paracidovorax citrulli*]
fig|80869.176.peg.110	T3S	MBL fold metallo-hydrolase [*Paracidovorax citrulli*]
fig|80869.176.peg.1128	T3S	GNAT family N-acetyltransferase [*Paracidovorax citrulli*]
fig|80869.176.peg.1133	T3S	4'-phosphopantetheinyl transferase [*Paracidovorax citrulli* AAC00-1]
fig|80869.176.peg.1134	T3S	hypothetical protein [*Paracidovorax citrulli*]
fig|80869.176.peg.1204	T3S	glycerophosphodiester phosphodiesterase [*Paracidovorax citrulli*]
fig|80869.176.peg.1227	T3S	Cardiolipin synthase B [*Paracidovorax citrulli*]
fig|80869.176.peg.125	T3S	DUF1800 family protein [*Paracidovorax citrulli*]
fig|80869.176.peg.1253	T3S	Tetratricopeptide TPR_2 repeat protein [*Paracidovorax citrulli* AAC00-1]
fig|80869.176.peg.127	T3S	putative amino-acid metabolite efflux pump [*Paracidovorax citrulli*]
fig|80869.176.peg.1294	T3S	cyclopropane-fatty-acyl-phospholipid synthase family protein [*Paracidovorax citrulli*]
fig|80869.176.peg.1333	T3S	urease accessory protein UreG [*Paracidovorax citrulli*]
fig|80869.176.peg.1334	T3S	urease accessory protein [*Paracidovorax citrulli*]
fig|80869.176.peg.1374	T3S	PilZ domain-containing protein [*Paracidovorax citrulli*]
fig|80869.176.peg.1418	T3S	isoleucine--tRNA ligase [*Paracidovorax citrulli*]
fig|80869.176.peg.1433	T3S	protein of unknown function DUF1653 [*Paracidovorax citrulli* AAC00-1]
fig|80869.176.peg.1437	T3S	hypothetical protein [*Paracidovorax citrulli*]
fig|80869.176.peg.1526	T3S	argininosuccinate lyase [*Paracidovorax citrulli*]
fig|80869.176.peg.1554	T3S	CDP-6-deoxy-delta-3,4-glucoseen reductase [*Paracidovorax citrulli*]
fig|80869.176.peg.1563	T3S	hypothetical protein [*Paracidovorax citrulli*]
fig|80869.176.peg.1577	T3S	ABC-F family ATP-binding cassette domain-containing protein [*Paracidovorax citrulli*]
fig|80869.176.peg.1579	T3S	uroporphyrinogen-III C-methyltransferase [*Paracidovorax citrulli*]
fig|80869.176.peg.1581	T3S	hydroxymethylbilane synthase [*Paracidovorax citrulli*]
fig|80869.176.peg.1582	T3S	phosphoenolpyruvate carboxylase [*Paracidovorax citrulli*]
fig|80869.176.peg.1583	T3S	YccS family putative transporter [*Paracidovorax citrulli*]
fig|80869.176.peg.1585	T3S	AI-2E family transporter [*Paracidovorax citrulli*]
fig|80869.176.peg.1591	T3S	SfnB family sulfur acquisition oxidoreductase [*Paracidovorax citrulli*]
fig|80869.176.peg.1592	T3S	SfnB family sulfur acquisition oxidoreductase [*Paracidovorax citrulli*]
fig|80869.176.peg.1597	T3S	exodeoxyribonuclease VII small subunit [*Paracidovorax citrulli*]
fig|80869.176.peg.1608	T3S	alpha-1,4-glucan--maltose-1-phosphate maltosyltransferase [*Paracidovorax citrulli*]
fig|80869.176.peg.1611	T3S	BCCT family transporter [*Paracidovorax citrulli*]
fig|80869.176.peg.1616	T3S	TOBE domain-containing protein [*Paracidovorax citrulli*]
fig|80869.176.peg.1627	T3S	efflux transporter outer membrane subunit [*Paracidovorax citrulli*]

Prot	Pred	Description
fig|80869.176.peg.1670	T3S	succinate-semialdehyde dehydrogenase/glutarate-semialdehyde dehydrogenase [*Paracidovorax citrulli*]
fig|80869.176.peg.1692	T3S	uroporphyrinogen-III C-methyltransferase [*Paracidovorax citrulli*]
fig|80869.176.peg.170	T3S	CerR family C-terminal domain-containing protein [*Paracidovorax citrulli*]
fig|80869.176.peg.1718	T3S	MFS transporter [*Paracidovorax citrulli*]
fig|80869.176.peg.1767	T3S	hypothetical protein [*Paracidovorax citrulli*]
fig|80869.176.peg.1785	T3S	NAD-dependent succinate-semialdehyde dehydrogenase [*Paracidovorax citrulli*]
fig|80869.176.peg.1800	T3S	Mannosyl-D-glycerate transport/metabolism systemrepressor MngR [*Paracidovorax citrulli*]
fig|80869.176.peg.1815	T3S	cytochrome o ubiquinol oxidase subunit IV [*Paracidovorax citrulli*]
fig|80869.176.peg.1849	T3S	multiple monosaccharide ABC transporter permease [*Paracidovorax citrulli*]
fig|80869.176.peg.1855	T3S	SDR family NAD(P)-dependent oxidoreductase [*Paracidovorax citrulli*]
fig|80869.176.peg.1856	T3S	SMP-30/gluconolactonase/LRE family protein [*Paracidovorax citrulli*]
fig|80869.176.peg.1870	T3S	MFS transporter [*Paracidovorax citrulli*]
fig|80869.176.peg.1876	T3S	DHH family phosphoesterase [*Paracidovorax citrulli*]
fig|80869.176.peg.1886	T3S	Murein DD-endopeptidase MepM [*Paracidovorax citrulli*]
fig|80869.176.peg.1889	T3S	alpha/beta hydrolase [*Paracidovorax citrulli*]
fig|80869.176.peg.1900	T3S	glutamine--tRNA ligase/YqeY domain fusion protein [*Paracidovorax citrulli*]
fig|80869.176.peg.1905	T3S	class III extradiol ring-cleavage dioxygenase [*Paracidovorax citrulli*]
fig|80869.176.peg.1966	T3S	TetR/AcrR family transcriptional regulator [*Paracidovorax citrulli*]
fig|80869.176.peg.1968	T3S	transcriptional repressor [*Paracidovorax citrulli*]
fig|80869.176.peg.1979	T3S	pyridoxamine 5'-phosphate oxidase [*Paracidovorax citrulli*]
fig|80869.176.peg.2012	T3S	pseudouridine synthase [*Paracidovorax citrulli*]
fig|80869.176.peg.2016	T3S	translesion DNA synthesis-associated protein ImuA [*Paracidovorax citrulli*]
fig|80869.176.peg.2027	T3S	GTP cyclohydrolase subunit MoaA [*Paracidovorax citrulli* AAC00-1]
fig|80869.176.peg.2030	T3S	gephyrin-like molybdotransferase Glp [*Paracidovorax citrulli*]
fig|80869.176.peg.2036	T3S	hypothetical protein [*Paracidovorax citrulli*]
fig|80869.176.peg.2069	T3S	phosphate ABC transporter permease PstC [*Paracidovorax citrulli*]
fig|80869.176.peg.2074	T3S	Polyphosphate kinase [*Paracidovorax citrulli* AAC00-1]
fig|80869.176.peg.2087	T3S	hypothetical protein [*Paracidovorax citrulli*]
fig|80869.176.peg.2088	T3S	D-serine/D-alanine/glycine transporter [*Paracidovorax citrulli*]
fig|80869.176.peg.2099	T3S	TonB-dependent receptor [*Paracidovorax citrulli*]
fig|80869.176.peg.2216	T3S	EAL domain-containing protein [*Paracidovorax citrulli*]
fig|80869.176.peg.2249	T3S	PAS domain S-box protein [*Paracidovorax citrulli*]
fig|80869.176.peg.226	T3S	transcriptional regulator, TetR family [*Paracidovorax citrulli* AAC00-1]
fig|80869.176.peg.2292	T3S	KGG domain-containing protein [*Paracidovorax citrulli*]
fig|80869.176.peg.2305	T3S	XopAP family type III secretion system effector [*Paracidovorax citrulli*]
fig|80869.176.peg.2343	T3S	glycoside hydrolase family 5 protein [*Paracidovorax citrulli*]
fig|80869.176.peg.2355	T3S	YdiU family protein [*Paracidovorax citrulli*]
fig|80869.176.peg.2432	T3S	BON domain-containing protein [*Paracidovorax citrulli*]
fig|80869.176.peg.2433	T3S	SulP family inorganic anion transporter [*Paracidovorax citrulli*]
fig|80869.176.peg.2441	T3S	NAD(P)/FAD-dependent oxidoreductase [*Paracidovorax citrulli*]
fig|80869.176.peg.2443	T3S	tRNA (guanosine(46)-N7)-methyltransferase TrmB [*Paracidovorax citrulli*]
fig|80869.176.peg.245	T3S	gamma-glutamyl-gamma-aminobutyrate hydrolase family protein [*Paracidovorax citrulli*]
fig|80869.176.peg.2477	T3S	putative avirulence protein AvrRxo1 [*Paracidovorax citrulli* AAC00-1]
fig|80869.176.peg.2502	T3S	hypothetical protein [*Paracidovorax citrulli*]
fig|80869.176.peg.2509	T3S	ATP-binding protein [*Paracidovorax citrulli*]

Prot	Pred	Description
fig|80869.176.peg.2519	T3S	YbaN family protein [*Paracidovorax citrulli*]
fig|80869.176.peg.2533	T3S	RDD domain containing protein [*Paracidovorax citrulli* AAC00-1]
fig|80869.176.peg.2553	T3S	TetR/AcrR family transcriptional regulator [*Paracidovorax citrulli*]
fig|80869.176.peg.2577	T3S	2OG-Fe dioxygenase family protein [*Paracidovorax citrulli*]
fig|80869.176.peg.2590	T3S	MULTISPECIES: CysB family HTH-type transcriptional regulator [Comamonadaceae]
fig|80869.176.peg.26	T3S	DNA topoisomerase (ATP-hydrolyzing) subunit B [*Paracidovorax citrulli*]
fig|80869.176.peg.263	T3S	phosphatase PAP2 family protein [*Paracidovorax citrulli*]
fig|80869.176.peg.2678	T3S	ABC transporter permease [*Paracidovorax citrulli*]
fig|80869.176.peg.2748	T3S	hypothetical protein [*Paracidovorax citrulli*]
fig|80869.176.peg.2750	T3S	Bifunctional protein PutA [*Paracidovorax citrulli*]
fig|80869.176.peg.2796	T3S	AlpA family transcriptional regulator [*Paracidovorax citrulli*]
fig|80869.176.peg.2832	T3S	hypothetical protein [*Paracidovorax citrulli*]
fig|80869.176.peg.2840	T3S	thioredoxin family protein [*Paracidovorax citrulli*]
fig|80869.176.peg.2842	T3S	cytochrome c553-like protein [*Paracidovorax citrulli* AAC00-1]
fig|80869.176.peg.2858	T3S	ThiF family adenylyltransferase [*Paracidovorax citrulli*]
fig|80869.176.peg.2862	T3S	lipid A export permease/ATP-binding protein MsbA [*Paracidovorax citrulli*]
fig|80869.176.peg.2880	T3S	DUF72 domain-containing protein [*Paracidovorax citrulli*]
fig|80869.176.peg.2883	T3S	DUF1624 domain-containing protein [*Paracidovorax citrulli*]
fig|80869.176.peg.2884	T3S	glutamate--tRNA ligase [*Paracidovorax citrulli*]
fig|80869.176.peg.2917	T3S	UDP-3-O-(3-hydroxymyristoyl)glucosamine N-acyltransferase [*Paracidovorax citrulli*]
fig|80869.176.peg.2932	T3S	MlaD family protein [*Paracidovorax citrulli*]
fig|80869.176.peg.2936	T3S	YitT family protein [*Paracidovorax citrulli*]
fig|80869.176.peg.2945	T3S	P1 family peptidase [*Paracidovorax citrulli*]
fig|80869.176.peg.2950	T3S	ATP synthase F1 subunit epsilon [*Paracidovorax citrulli*]
fig|80869.176.peg.3029	T3S	hypothetical protein [*Paracidovorax citrulli*]
fig|80869.176.peg.3042	T3S	hypothetical protein Aave_1685 [*Paracidovorax citrulli* AAC00-1]
fig|80869.176.peg.307	T3S	hypothetical protein, partial [*Paracidovorax citrulli*]
fig|80869.176.peg.3086	T3S	polyhydroxyalkanoate synthesis repressor PhaR [*Paracidovorax citrulli*]
fig|80869.176.peg.3088	T3S	type 1 glutamine amidotransferase domain-containing protein [*Paracidovorax citrulli*]
fig|80869.176.peg.3134	T3S	SDR family NAD(P)-dependent oxidoreductase [*Paracidovorax citrulli*]
fig|80869.176.peg.3165	T3S	cation-translocating P-type ATPase [*Paracidovorax citrulli*]
fig|80869.176.peg.3170	T3S	malonyl-CoA synthase [*Paracidovorax citrulli*]
fig|80869.176.peg.3187	T3S	glycine betaine/L-proline ABC transporter permease ProW [*Paracidovorax citrulli*]
fig|80869.176.peg.320	T3S	16S rRNA (cytosine(967)-C(5))-methyltransferase RsmB [*Paracidovorax citrulli*]
fig|80869.176.peg.322	T3S	ATP-binding protein [*Paracidovorax citrulli*]
fig|80869.176.peg.3232	T3S	DUF2069 domain-containing protein [*Paracidovorax citrulli*]
fig|80869.176.peg.3257	T3S	GTPase HflX [*Paracidovorax citrulli*]
fig|80869.176.peg.3272	T3S	protein-L-isoaspartate(D-aspartate) O-methyltransferase [*Paracidovorax citrulli*]
fig|80869.176.peg.3298	T3S	CaiB/BaiF CoA-transferase family protein [*Paracidovorax citrulli*]
fig|80869.176.peg.3331	T3S	uncharacterized protein DUF2132 [*Paracidovorax citrulli*]
fig|80869.176.peg.3339	T3S	DNA internalization-related competence protein ComEC/Rec2 [*Paracidovorax citrulli*]
fig|80869.176.peg.3345	T3S	pyridoxal kinase PdxY [*Paracidovorax citrulli*]
fig|80869.176.peg.3371	T3S	hypothetical protein [*Paracidovorax citrulli*]
fig|80869.176.peg.3375	T3S	EAL and HDOD domain-containing protein [*Paracidovorax citrulli*]

Prot	Pred	Description
fig|80869.176.peg.3386	T3S	N-acetylmuramoyl-L-alanine amidase [*Paracidovorax citrulli*]
fig|80869.176.peg.3395	T3S	helix-turn-helix domain-containing protein [*Paracidovorax citrulli*]
fig|80869.176.peg.3413	T3S	ABC transporter transmembrane domain-containing protein [*Paracidovorax citrulli*]
fig|80869.176.peg.342	T3S	PhaM family polyhydroxyalkanoate granule multifunctional regulatory protein [*Paracidovorax citrulli*]
fig|80869.176.peg.3421	T3S	MULTISPECIES: NADH-quinone oxidoreductase subunit NuoI [Comamonadaceae]
fig|80869.176.peg.3441	T3S	hypothetical protein [*Paracidovorax citrulli*]
fig|80869.176.peg.3453	T3S	molybdopterin converting factor subunit 1 [*Paracidovorax citrulli*]
fig|80869.176.peg.3480	T3S	nucleotide exchange factor GrpE [*Paracidovorax citrulli*]
fig|80869.176.peg.3493	T3S	phosphoribosylanthranilate isomerase [*Paracidovorax citrulli* AAC00-1]
fig|80869.176.peg.3498	T3S	LON peptidase substrate-binding domain-containing protein [*Paracidovorax citrulli*]
fig|80869.176.peg.3505	T3S	lysine--tRNA ligase [*Paracidovorax citrulli*]
fig|80869.176.peg.3506	T3S	Phytochrome-like protein cph2 [*Paracidovorax citrulli*]
fig|80869.176.peg.3555	T3S	tryptophan 2,3-dioxygenase [*Paracidovorax citrulli*]
fig|80869.176.peg.3566	T3S	neutral zinc metallopeptidase [*Paracidovorax citrulli*]
fig|80869.176.peg.3582	T3S	ABC transporter ATP-binding protein [*Paracidovorax citrulli*]
fig|80869.176.peg.3602	T3S	glycerate kinase [*Paracidovorax citrulli*]
fig|80869.176.peg.3615	T3S	NCS1 family nucleobase:cation symporter-1 [*Paracidovorax citrulli*]
fig|80869.176.peg.3617	T3S	GntR family transcriptional regulator [*Paracidovorax citrulli*]
fig|80869.176.peg.362	T3S	hypothetical protein APS58_1500 [*Paracidovorax citrulli*]
fig|80869.176.peg.3636	T3S	DMT family transporter [*Paracidovorax citrulli*]
fig|80869.176.peg.3681	T3S	PelD GGDEF domain-containing protein [*Paracidovorax citrulli*]
fig|80869.176.peg.3718	T3S	protein of unknown function DUF1415 [*Paracidovorax citrulli* AAC00-1]
fig|80869.176.peg.3728	T3S	penicillin-binding protein 1A [*Paracidovorax citrulli*]
fig|80869.176.peg.3752	T3S	peptidoglycan-binding domain-containing protein [*Paracidovorax citrulli*]
fig|80869.176.peg.3788	T3S	ABC transporter ATP-binding protein [*Paracidovorax citrulli*]
fig|80869.176.peg.1770	T4S	hypothetical protein [*Paracidovorax citrulli*]
fig|80869.176.peg.1882	T4S	major facilitator superfamily MFS_1 [*Paracidovorax citrulli* AAC00-1]
fig|80869.176.peg.3722	T4S	Ig domain protein, group 1 domain protein [*Paracidovorax citrulli* AAC00-1]
fig|80869.176.peg.1690	T4S	acyl-CoA thioesterase [*Paracidovorax citrulli*]
fig|80869.176.peg.2285	T4S	STY0301 family protein [*Paracidovorax citrulli*]
fig|80869.176.peg.2375	T4S	ABC transporter ATP-binding protein [*Paracidovorax citrulli*]
fig|80869.176.peg.2178	T4S	Ku protein [*Paracidovorax citrulli*]
fig|80869.176.peg.981	T4S	bacterioferritin [*Paracidovorax citrulli*]
fig|80869.176.peg.3026	T4S	terminase small subunit [*Paracidovorax citrulli*]
fig|80869.176.peg.3447	T4S	molybdenum cofactor biosynthesis protein MoaE [*Paracidovorax citrulli*]
fig|80869.176.peg.2584	T4S	hypothetical protein [*Paracidovorax citrulli*]
fig|80869.176.peg.3074	T4S	isoaspartyl peptidase/L-asparaginase family protein [*Paracidovorax citrulli*
fig|80869.176.peg.1729	T4S	RNA recognition motif domain-containing protein [*Paracidovorax citrulli*]
fig|80869.176.peg.2487	T4S	hypothetical protein Aave_3072 [*Paracidovorax citrulli* AAC00-1]
fig|80869.176.peg.1938	T4S	DUF1795 domain-containing protein [*Paracidovorax citrulli*]
fig|80869.176.peg.2145	T4S	MULTISPECIES: amino-acid N-acetyltransferase [Paracidovorax]
fig|80869.176.peg.249	T4S	DUF924 family protein [*Paracidovorax citrulli*]
fig|80869.176.peg.70	T4S	hypothetical protein [*Paracidovorax citrulli*]
fig|80869.176.peg.312	T4S	50S ribosomal protein L11 [*Paracidovorax citrulli*]
fig|80869.176.peg.2175	T4S	hypothetical protein [*Paracidovorax citrulli*]

Prot	Pred	Description
fig|80869.176.peg.1857	T4S	conserved hypothetical protein [*Paracidovorax citrulli* AAC00-1]
fig|80869.176.peg.4290	T4S	MULTISPECIES: LysR family transcriptional regulator [Paracidovorax]
fig|80869.176.peg.3269	T4S	SMC-Scp complex subunit ScpB [*Paracidovorax citrulli*]
fig|80869.176.peg.1287	T4S	PP2C family protein-serine/threonine phosphatase [*Paracidovorax citrulli*]
fig|80869.176.peg.4524	T4S	hypothetical protein [*Paracidovorax citrulli*]
fig|80869.176.peg.333	T4S	cryptochrome/photolyase family protein [*Paracidovorax citrulli*]
fig|80869.176.peg.3369	T4S	phosphopyruvate hydratase [*Paracidovorax citrulli*]
fig|80869.176.peg.929	T4S	glutathione-regulated potassium-efflux system protein KefC [*Paracidovorax citrulli*]
fig|80869.176.peg.1344	T4S	hypothetical protein [*Paracidovorax citrulli*]
fig|80869.176.peg.2688	T4S	hypothetical protein [*Paracidovorax citrulli*]
fig|80869.176.peg.2904	T4S	AAA family ATPase [*Paracidovorax citrulli*]
fig|80869.176.peg.547	T4S	DUF3025 domain-containing protein [*Paracidovorax citrulli*]
fig|80869.176.peg.1286	T4S	serine/threonine protein kinase [*Paracidovorax citrulli*]
fig|80869.176.peg.1055	T4S	hypothetical protein [*Paracidovorax citrulli*]
fig|80869.176.peg.1657	T4S	inositol monophosphatase family protein [*Paracidovorax citrulli*]
fig|80869.176.peg.626	T4S	N-acetylmuramoyl-L-alanine amidase [*Paracidovorax citrulli*]
fig|80869.176.peg.1933	T4S	hypothetical protein [*Paracidovorax citrulli*]
fig|80869.176.peg.4087	T4S	AraC family transcriptional regulator [*Paracidovorax citrulli*]
fig|80869.176.peg.2746	T4S	hypothetical protein [*Paracidovorax citrulli*]
fig|80869.176.peg.214	T4S	Fic family protein [*Paracidovorax citrulli*]
fig|80869.176.peg.1073	T4S	4-hydroxy-3-methylbut-2-enyl diphosphate reductase [*Paracidovorax citrulli*]
fig|80869.176.peg.2827	T4S	SsrA-binding protein SmpB [*Paracidovorax citrulli*]
fig|80869.176.peg.4215	T4S	F0F1 ATP synthase subunit gamma [*Paracidovorax citrulli*]
fig|80869.176.peg.3242	T4S	hypothetical protein [*Paracidovorax citrulli*]
fig|80869.176.peg.2046	T4S	transglycosylase SLT domain-containing protein [*Paracidovorax citrulli*]
fig|80869.176.peg.4107	T4S	type III secretion system outer membrane ring subunit SctC [*Paracidovorax citrulli*]
fig|80869.176.peg.3007	T4S	hypothetical protein [*Paracidovorax citrulli*]
fig|80869.176.peg.3124	T4S	XopE/AvrPphe family type III secretion system effector [*Paracidovorax citrulli*]
fig|80869.176.peg.867	T4S	5'-nucleotidase [*Paracidovorax citrulli*]
fig|80869.176.peg.4052	T4S	30S ribosomal protein S4 [*Paracidovorax citrulli*]
fig|80869.176.peg.2136	T4S	adenylate kinase [*Paracidovorax citrulli*]
fig|80869.176.peg.2669	T4S	hypothetical protein [*Paracidovorax citrulli*]
fig|80869.176.peg.1507	T4S	hypothetical protein [*Paracidovorax citrulli*]
fig|80869.176.peg.1317	T4S	type IV pilin protein [*Paracidovorax citrulli*]
fig|80869.176.peg.980	T4S	BON domain-containing protein [*Paracidovorax citrulli*]
fig|80869.176.peg.2159	T4S	MetQ/NlpA family ABC transporter substrate-binding protein [*Paracidovorax citrulli*]
fig|80869.176.peg.386	T4S	Bug family tripartite tricarboxylate transporter substrate binding protein [*Paracidovorax citrulli*]
fig|80869.176.peg.2910	T4S	Bug family tripartite tricarboxylate transporter substrate binding protein [*Paracidovorax citrulli*]
fig|80869.176.peg.2153	T4S	sulfate ABC transporter substrate-binding protein [*Paracidovorax citrulli*]
fig|80869.176.peg.1099	T4S	hypothetical protein [*Paracidovorax citrulli*]
fig|80869.176.peg.250	T4S	OmpA family protein [*Paracidovorax citrulli*]
fig|80869.176.peg.2989	T4S	glycoside hydrolase family protein [*Paracidovorax citrulli*]
fig|80869.176.peg.1476	T4S	sigma-54-dependent transcriptional regulator [*Paracidovorax citrulli*]
fig|80869.176.peg.866	T4S	EF-hand domain-containing protein [*Paracidovorax citrulli*]

*P_citrulli*_HPP21_9_4B

Prot	Pred	Description
fig|80869.177.peg.1686	T3S	Ig domain protein, group 1 domain protein [*Paracidovorax citrulli* AAC00-1]
fig|80869.177.peg.3525	T3S	major facilitator superfamily MFS_1 [*Paracidovorax citrulli* AAC00-1]
fig|80869.177.peg.3637	T3S	hypothetical protein [*Paracidovorax citrulli*]
fig|80869.177.peg.3717	T3S	acyl-CoA thioesterase [*Paracidovorax citrulli*]
fig|80869.177.peg.3123	T3S	STY0301 family protein [*Paracidovorax citrulli*]
fig|80869.177.peg.3032	T3S	ABC transporter ATP-binding protein [*Paracidovorax citrulli*]
fig|80869.177.peg.3230	T3S	Ku protein [*Paracidovorax citrulli*]
fig|80869.177.peg.4424	T3S	bacterioferritin [*Paracidovorax citrulli*]
fig|80869.177.peg.2381	T3S	terminase small subunit [*Paracidovorax citrulli*]
fig|80869.177.peg.100	T3S	rhodanese-like domain-containing protein [*Paracidovorax citrulli*]
fig|80869.177.peg.1035	T3S	GNAT family N-acetyltransferase [Paracidovorax avenae]
fig|80869.177.peg.1056	T3S	ProQ/FINO family protein [*Paracidovorax citrulli*]
fig|80869.177.peg.1088	T3S	AmpG family muropeptide MFS transporter [*Paracidovorax citrulli*]
fig|80869.177.peg.1091	T3S	DME family drug/metabolite transporter [*Paracidovorax citrulli*]
fig|80869.177.peg.1093	T3S	SLC13 family permease [*Paracidovorax citrulli*]
fig|80869.177.peg.1112	T3S	amino acid ABC transporter ATP-binding protein [*Paracidovorax citrulli*]
fig|80869.177.peg.1121	T3S	MULTISPECIES: ABC transporter ATP-binding protein [Paracidovorax]
fig|80869.177.peg.1136	T3S	DHA2 family efflux MFS transporter permease subunit [*Paracidovorax citrulli*]
fig|80869.177.peg.1137	T3S	efflux RND transporter periplasmic adaptor subunit [*Paracidovorax citrulli*]
fig|80869.177.peg.116	T3S	GNAT family N-acetyltransferase [*Paracidovorax citrulli*]
fig|80869.177.peg.1175	T3S	potassium-transporting ATPase subunit KdpB [*Paracidovorax citrulli*]
fig|80869.177.peg.1199	T3S	NEL-type E3 ubiquitin ligase domain-containing protein [*Paracidovorax citrulli*]
fig|80869.177.peg.1208	T3S	N-acetylneuraminate epimerase [*Paracidovorax citrulli*]
fig|80869.177.peg.1242	T3S	TspO/MBR family protein [*Paracidovorax citrulli*]
fig|80869.177.peg.1247	T3S	hypothetical protein [*Paracidovorax citrulli*]
fig|80869.177.peg.1254	T3S	DUF4139 domain-containing protein [*Paracidovorax citrulli*]
fig|80869.177.peg.1281	T3S	pectate lyase [*Paracidovorax citrulli*]
fig|80869.177.peg.1285	T3S	hypothetical protein [*Paracidovorax citrulli*]
fig|80869.177.peg.1286	T3S	hypothetical protein [*Paracidovorax citrulli*]
fig|80869.177.peg.129	T3S	ABC transporter permease [*Paracidovorax citrulli*]
fig|80869.177.peg.1298	T3S	hypothetical protein [*Paracidovorax citrulli*]
fig|80869.177.peg.1314	T3S	uracil-DNA glycosylase [*Paracidovorax citrulli*]
fig|80869.177.peg.1329	T3S	hypothetical protein APS58_2360 [*Paracidovorax citrulli*]
fig|80869.177.peg.1331	T3S	pyrroline-5-carboxylate reductase [*Paracidovorax citrulli*]
fig|80869.177.peg.134	T3S	Metallo-beta-lactamase superfamily protein [*Paracidovorax citrulli*]
fig|80869.177.peg.1376	T3S	FAD-linked oxidase C-terminal domain-containing protein [*Paracidovorax citrulli*]
fig|80869.177.peg.1389	T3S	transcriptional regulator, AraC family [*Paracidovorax citrulli* AAC00-1]
fig|80869.177.peg.1391	T3S	NarK family nitrate/nitrite MFS transporter [*Paracidovorax citrulli*]
fig|80869.177.peg.1419	T3S	DUF2169 domain-containing protein [*Paracidovorax citrulli*]
fig|80869.177.peg.1450	T3S	putative zinc protease protein [*Paracidovorax citrulli* AAC00-1]
fig|80869.177.peg.1451	T3S	DNA-3-methyladenine glycosylase I [*Paracidovorax citrulli*]
fig|80869.177.peg.146	T3S	glycoside hydrolase family 15 protein [*Paracidovorax citrulli*]

Prot	Pred	Description
fig|80869.177.peg.1466	T3S	M48 family metallopeptidase [*Paracidovorax citrulli*]
fig|80869.177.peg.1473	T3S	chromate efflux transporter [*Paracidovorax citrulli*]
fig|80869.177.peg.1484	T3S	CobW family GTP-binding protein [*Paracidovorax citrulli*]
fig|80869.177.peg.1531	T3S	3-deoxy-7-phosphoheptulonate synthase [*Paracidovorax citrulli*]
fig|80869.177.peg.1544	T3S	ribonucleoside-diphosphate reductase subunit alpha [*Paracidovorax citrulli*]
fig|80869.177.peg.1578	T3S	hydroxymethylpyrimidine/phosphomethylpyrimidine kinase [*Paracidovorax citrulli*]
fig|80869.177.peg.1587	T3S	Holliday junction resolvase RuvX [*Paracidovorax citrulli*]
fig|80869.177.peg.1620	T3S	ABC transporter ATP-binding protein [*Paracidovorax citrulli*]
fig|80869.177.peg.1656	T3S	peptidoglycan-binding domain-containing protein [*Paracidovorax citrulli*]
fig|80869.177.peg.166	T3S	hypothetical protein [*Paracidovorax citrulli*]
fig|80869.177.peg.1680	T3S	penicillin-binding protein 1A [*Paracidovorax citrulli*]
fig|80869.177.peg.1690	T3S	protein of unknown function DUF1415 [*Paracidovorax citrulli* AAC00-1]
fig|80869.177.peg.1727	T3S	PelD GGDEF domain-containing protein [*Paracidovorax citrulli*]
fig|80869.177.peg.1770	T3S	DMT family transporter [*Paracidovorax citrulli*]
fig|80869.177.peg.1789	T3S	GntR family transcriptional regulator [*Paracidovorax citrulli*]
fig|80869.177.peg.1791	T3S	NCS1 family nucleobase:cation symporter-1 [*Paracidovorax citrulli*]
fig|80869.177.peg.1804	T3S	glycerate kinase [*Paracidovorax citrulli*]
fig|80869.177.peg.1824	T3S	ABC transporter ATP-binding protein [*Paracidovorax citrulli*]
fig|80869.177.peg.1840	T3S	neutral zinc metallopeptidase [*Paracidovorax citrulli*]
fig|80869.177.peg.1851	T3S	tryptophan 2,3-dioxygenase [*Paracidovorax citrulli*]
fig|80869.177.peg.1900	T3S	Phytochrome-like protein cph2 [*Paracidovorax citrulli*]
fig|80869.177.peg.1901	T3S	lysine--tRNA ligase [*Paracidovorax citrulli*]
fig|80869.177.peg.1908	T3S	LON peptidase substrate-binding domain-containing protein [*Paracidovorax citrulli*]
fig|80869.177.peg.1913	T3S	phosphoribosylanthranilate isomerase [*Paracidovorax citrulli* AAC00-1]
fig|80869.177.peg.1926	T3S	nucleotide exchange factor GrpE [*Paracidovorax citrulli*]
fig|80869.177.peg.1937	T3S	hypothetical protein [*Paracidovorax citrulli*]
fig|80869.177.peg.194	T3S	ABC transporter permease [*Paracidovorax citrulli*]
fig|80869.177.peg.1954	T3S	molybdopterin converting factor subunit 1 [*Paracidovorax citrulli*]
fig|80869.177.peg.1967	T3S	hypothetical protein [*Paracidovorax citrulli*]
fig|80869.177.peg.1986	T3S	MULTISPECIES: NADH-quinone oxidoreductase subunit NuoI [Comamonadaceae]
fig|80869.177.peg.1994	T3S	ABC transporter transmembrane domain-containing protein [*Paracidovorax citrulli*]
fig|80869.177.peg.2012	T3S	winged helix-turn-helix transcriptional regulator [*Paracidovorax citrulli*]
fig|80869.177.peg.2021	T3S	N-acetylmuramoyl-L-alanine amidase [*Paracidovorax citrulli*]
fig|80869.177.peg.2032	T3S	EAL and HDOD domain-containing protein [*Paracidovorax citrulli*]
fig|80869.177.peg.2036	T3S	hypothetical protein [*Paracidovorax citrulli*]
fig|80869.177.peg.2062	T3S	pyridoxal kinase PdxY [*Paracidovorax citrulli*]
fig|80869.177.peg.2068	T3S	DNA internalization-related competence protein ComEC/Rec2 [*Paracidovorax citrulli*]
fig|80869.177.peg.2076	T3S	uncharacterized protein DUF2132 [*Paracidovorax citrulli*]
fig|80869.177.peg.2109	T3S	CaiB/BaiF CoA transferase family protein [*Paracidovorax citrulli*]
fig|80869.177.peg.2135	T3S	protein-L-isoaspartate(D-aspartate) O-methyltransferase [*Paracidovorax citrulli*]
fig|80869.177.peg.2150	T3S	GTPase HflX [*Paracidovorax citrulli*]
fig|80869.177.peg.2175	T3S	DUF2069 domain-containing protein [*Paracidovorax citrulli*]
fig|80869.177.peg.2220	T3S	glycine betaine/L-proline ABC transporter permease ProW [*Paracidovorax citrulli*]
fig|80869.177.peg.2238	T3S	malonate--CoA ligase [*Paracidovorax citrulli*]

Prot	Pred	Description
fig|80869.177.peg.2243	T3S	heavy metal translocating P-type ATPase [*Paracidovorax citrulli*]
fig|80869.177.peg.2274	T3S	SDR family NAD(P)-dependent oxidoreductase [*Paracidovorax citrulli*]
fig|80869.177.peg.2319	T3S	type 1 glutamine amidotransferase domain-containing protein [*Paracidovorax citrulli*]
fig|80869.177.peg.2321	T3S	polyhydroxyalkanoate synthesis repressor PhaR [*Paracidovorax citrulli*]
fig|80869.177.peg.2365	T3S	hypothetical protein Aave_1685 [*Paracidovorax citrulli* AAC00-1]
fig|80869.177.peg.2368	T3S	hypothetical protein [*Paracidovorax citrulli*]
fig|80869.177.peg.2378	T3S	hypothetical protein [*Paracidovorax citrulli*]
fig|80869.177.peg.2457	T3S	ATP synthase F1 subunit epsilon [*Paracidovorax citrulli*]
fig|80869.177.peg.2462	T3S	P1 family peptidase [*Paracidovorax citrulli*]
fig|80869.177.peg.2471	T3S	YitT family protein [*Paracidovorax citrulli*]
fig|80869.177.peg.2475	T3S	MlaD family protein [*Paracidovorax citrulli*]
fig|80869.177.peg.2490	T3S	UDP-3-O-(3-hydroxymyristoyl)glucosamine N-acyltransferase [*Paracidovorax citrulli*]
fig|80869.177.peg.2523	T3S	glutamate--tRNA ligase [*Paracidovorax citrulli*]
fig|80869.177.peg.2524	T3S	DUF1624 domain-containing protein [*Paracidovorax citrulli*]
fig|80869.177.peg.2527	T3S	DUF72 domain-containing protein [*Paracidovorax citrulli*]
fig|80869.177.peg.2545	T3S	lipid A export permease/ATP-binding protein MsbA [*Paracidovorax citrulli*]
fig|80869.177.peg.2549	T3S	ThiF family adenylyltransferase [*Paracidovorax citrulli*]
fig|80869.177.peg.2565	T3S	cytochrome c553-like protein [*Paracidovorax citrulli* AAC00-1]
fig|80869.177.peg.2567	T3S	thioredoxin family protein [*Paracidovorax citrulli*]
fig|80869.177.peg.2575	T3S	hypothetical protein [*Paracidovorax citrulli*]
fig|80869.177.peg.2611	T3S	helix-turn-helix transcriptional regulator [*Paracidovorax citrulli*]
fig|80869.177.peg.2658	T3S	Bifunctional protein PutA [*Paracidovorax citrulli*]
fig|80869.177.peg.2660	T3S	hypothetical protein [*Paracidovorax citrulli*]
fig|80869.177.peg.267	T3S	ABC transporter ATP-binding protein [*Paracidovorax citrulli*]
fig|80869.177.peg.2730	T3S	ABC transporter permease [*Paracidovorax citrulli*]
fig|80869.177.peg.28	T3S	septal ring lytic transglycosylase RlpA family protein [*Paracidovorax citrulli*]
fig|80869.177.peg.2818	T3S	MULTISPECIES: CysB family HTH-type transcriptional regulator [Comamonadaceae]
fig|80869.177.peg.2831	T3S	2OG-Fe dioxygenase family protein [*Paracidovorax citrulli*]
fig|80869.177.peg.284	T3S	FmdB family transcriptional regulator [*Paracidovorax citrulli*]
fig|80869.177.peg.2855	T3S	TetR/AcrR family transcriptional regulator [*Paracidovorax citrulli*]
fig|80869.177.peg.2874	T3S	RDD domain containing protein [*Paracidovorax citrulli* AAC00-1]
fig|80869.177.peg.2888	T3S	YbaN family protein [*Paracidovorax citrulli*]
fig|80869.177.peg.289	T3S	porin [*Paracidovorax citrulli*]
fig|80869.177.peg.2898	T3S	ATP-binding protein [*Paracidovorax citrulli*]
fig|80869.177.peg.2905	T3S	hypothetical protein [*Paracidovorax citrulli*]
fig|80869.177.peg.2930	T3S	putative avirulence protein AvrRxo1 [*Paracidovorax citrulli* AAC00-1]
fig|80869.177.peg.2964	T3S	tRNA (guanosine(46)-N7)-methyltransferase TrmB [*Paracidovorax citrulli*]
fig|80869.177.peg.2966	T3S	NAD(P)/FAD-dependent oxidoreductase [*Paracidovorax citrulli*]
fig|80869.177.peg.2974	T3S	SulP family inorganic anion transporter [*Paracidovorax citrulli*]
fig|80869.177.peg.2975	T3S	BON domain-containing protein [*Paracidovorax citrulli*]
fig|80869.177.peg.305	T3S	hypothetical protein FRC90_19215 [*Paracidovorax citrulli*]
fig|80869.177.peg.3052	T3S	protein adenylyltransferase SelO [*Paracidovorax citrulli*]
fig|80869.177.peg.3064	T3S	glycoside hydrolase family 5 protein [*Paracidovorax citrulli*]
fig|80869.177.peg.3103	T3S	XopAP family type III secretion system effector [*Paracidovorax citrulli*]
fig|80869.177.peg.3116	T3S	KGG domain-containing protein [*Paracidovorax citrulli*]

Prot	Pred	Description
fig|80869.177.peg.3159	T3S	PAS domain S-box protein [*Paracidovorax citrulli*]
fig|80869.177.peg.3192	T3S	EAL domain-containing protein [*Paracidovorax citrulli*]
fig|80869.177.peg.325	T3S	chemoreceptor glutamine deamidase CheD [*Paracidovorax citrulli*]
fig|80869.177.peg.326	T3S	CheR family methyltransferase [*Paracidovorax citrulli*]
fig|80869.177.peg.3310	T3S	TonB-dependent receptor family protein [*Paracidovorax citrulli*]
fig|80869.177.peg.3320	T3S	D-serine/D-alanine/glycine transporter [*Paracidovorax citrulli*]
fig|80869.177.peg.3321	T3S	hypothetical protein [*Paracidovorax citrulli*]
fig|80869.177.peg.3334	T3S	Polyphosphate kinase [*Paracidovorax citrulli* AAC00-1]
fig|80869.177.peg.3339	T3S	phosphate ABC transporter permease PstC [*Paracidovorax citrulli*]
fig|80869.177.peg.3373	T3S	hypothetical protein [*Paracidovorax citrulli*]
fig|80869.177.peg.3379	T3S	gephyrin-like molybdotransferase Glp [*Paracidovorax citrulli*]
fig|80869.177.peg.3382	T3S	GTP cyclohydrolase subunit MoaA [*Paracidovorax citrulli* AAC00-1]
fig|80869.177.peg.3393	T3S	translesion DNA synthesis-associated protein ImuA [*Paracidovorax citrulli*]
fig|80869.177.peg.3397	T3S	pseudouridine synthase [*Paracidovorax citrulli*]
fig|80869.177.peg.3430	T3S	pyridoxamine 5'-phosphate oxidase [*Paracidovorax citrulli*]
fig|80869.177.peg.3441	T3S	transcriptional repressor [*Paracidovorax citrulli*]
fig|80869.177.peg.3443	T3S	TetR/AcrR family transcriptional regulator [*Paracidovorax citrulli*]
fig|80869.177.peg.350	T3S	flagellin [*Paracidovorax citrulli*]
fig|80869.177.peg.3502	T3S	DODA-type extradiol aromatic ring-opening family dioxygenase [*Paracidovorax citrulli*]
fig|80869.177.peg.3507	T3S	glutamine--tRNA ligase/YqeY domain fusion protein [*Paracidovorax citrulli*]
fig|80869.177.peg.351	T3S	flagellin [*Paracidovorax citrulli*]
fig|80869.177.peg.3518	T3S	alpha/beta hydrolase [*Paracidovorax citrulli*]
fig|80869.177.peg.3521	T3S	Murein DD-endopeptidase MepM [*Paracidovorax citrulli*]
fig|80869.177.peg.3531	T3S	DHH family phosphoesterase [*Paracidovorax citrulli*]
fig|80869.177.peg.3537	T3S	MFS transporter [*Paracidovorax citrulli*]
fig|80869.177.peg.354	T3S	conserved hypothetical protein [*Paracidovorax citrulli* AAC00-1]
fig|80869.177.peg.3544	T3S	GAF domain-containing sensor histidine kinase [*Paracidovorax citrulli*]
fig|80869.177.peg.3551	T3S	SMP-30/gluconolactonase/LRE family protein [*Paracidovorax citrulli*]
fig|80869.177.peg.1686	T4S	Ig domain protein, group 1 domain protein [*Paracidovorax citrulli* AAC00-1]
fig|80869.177.peg.3525	T4S	major facilitator superfamily MFS_1 [*Paracidovorax citrulli* AAC00-1]
fig|80869.177.peg.3637	T4S	hypothetical protein [*Paracidovorax citrulli*]
fig|80869.177.peg.3717	T4S	acyl-CoA thioesterase [*Paracidovorax citrulli*]
fig|80869.177.peg.3123	T4S	STY0301 family protein [*Paracidovorax citrulli*]
fig|80869.177.peg.3032	T4S	ABC transporter ATP-binding protein [*Paracidovorax citrulli*]
fig|80869.177.peg.3230	T4S	Ku protein [*Paracidovorax citrulli*]
fig|80869.177.peg.4424	T4S	bacterioferritin [*Paracidovorax citrulli*]
fig|80869.177.peg.2381	T4S	terminase small subunit [*Paracidovorax citrulli*]
fig|80869.177.peg.1961	T4S	molybdenum cofactor biosynthesis protein MoaE [*Paracidovorax citrulli*]
fig|80869.177.peg.2824	T4S	hypothetical protein [*Paracidovorax citrulli*]
fig|80869.177.peg.2333	T4S	isoaspartyl peptidase/L-asparaginase family protein [*Paracidovorax citrulli*]
fig|80869.177.peg.3678	T4S	RNA recognition motif domain-containing protein [*Paracidovorax citrulli*]
fig|80869.177.peg.2920	T4S	hypothetical protein Aave_3072 [*Paracidovorax citrulli* AAC00-1]
fig|80869.177.peg.3469	T4S	DUF1795 domain-containing protein [*Paracidovorax citrulli*]
fig|80869.177.peg.3264	T4S	MULTISPECIES: amino-acid N-acetyltransferase [Paracidovorax]
fig|80869.177.peg.558	T4S	DUF924 family protein [*Paracidovorax citrulli*]

Prot	Pred	Description
fig|80869.177.peg.736	T4S	hypothetical protein [*Paracidovorax citrulli*]
fig|80869.177.peg.496	T4S	50S ribosomal protein L11 [*Paracidovorax citrulli*]
fig|80869.177.peg.3233	T4S	hypothetical protein [*Paracidovorax citrulli*]
fig|80869.177.peg.3550	T4S	conserved hypothetical protein [*Paracidovorax citrulli* AAC00-1]
fig|80869.177.peg.2138	T4S	SMC-Scp complex subunit ScpB [*Paracidovorax citrulli*]
fig|80869.177.peg.4119	T4S	PP2C family protein-serine/threonine phosphatase [*Paracidovorax citrulli*]
fig|80869.177.peg.880	T4S	hypothetical protein [*Paracidovorax citrulli*]
fig|80869.177.peg.475	T4S	cryptochrome/photolyase family protein [*Paracidovorax citrulli*]
fig|80869.177.peg.2038	T4S	phosphopyruvate hydratase [*Paracidovorax citrulli*]
fig|80869.177.peg.4476	T4S	glutathione-regulated potassium-efflux system protein KefC [*Paracidovorax citrulli*]
fig|80869.177.peg.4063	T4S	hypothetical protein [*Paracidovorax citrulli*]
fig|80869.177.peg.2720	T4S	hypothetical protein [*Paracidovorax citrulli*]
fig|80869.177.peg.2503	T4S	AAA family ATPase [*Paracidovorax citrulli*]
fig|80869.177.peg.260	T4S	DUF3025 domain-containing protein [*Paracidovorax citrulli*]
fig|80869.177.peg.4120	T4S	serine/threonine protein kinase [*Paracidovorax citrulli*]
fig|80869.177.peg.4351	T4S	hypothetical protein [*Paracidovorax citrulli*]
fig|80869.177.peg.3750	T4S	inositol monophosphatase family protein [*Paracidovorax citrulli*]
fig|80869.177.peg.181	T4S	N-acetylmuramoyl-L-alanine amidase [*Paracidovorax citrulli*]
fig|80869.177.peg.3474	T4S	hypothetical protein [*Paracidovorax citrulli*]
fig|80869.177.peg.1321	T4S	AraC family transcriptional regulator [*Paracidovorax citrulli*]
fig|80869.177.peg.2662	T4S	hypothetical protein [*Paracidovorax citrulli*]
fig|80869.177.peg.593	T4S	Fic family protein [*Paracidovorax citrulli*]
fig|80869.177.peg.4333	T4S	4-hydroxy-3-methylbut-2-enyl diphosphate reductase [*Paracidovorax citrulli*]
fig|80869.177.peg.2580	T4S	SsrA-binding protein SmpB [*Paracidovorax citrulli*]
fig|80869.177.peg.1191	T4S	F0F1 ATP synthase subunit gamma [*Paracidovorax citrulli*]
fig|80869.177.peg.2165	T4S	hypothetical protein [*Paracidovorax citrulli*]
fig|80869.177.peg.3363	T4S	transglycosylase SLT domain-containing protein [*Paracidovorax citrulli*]
fig|80869.177.peg.1301	T4S	type III secretion system outer membrane ring subunit SctC [*Paracidovorax citrulli*]
fig|80869.177.peg.2399	T4S	hypothetical protein [*Paracidovorax citrulli*]
fig|80869.177.peg.2284	T4S	XopE/AvrPphe family type III secretion system effector [*Paracidovorax citrulli*]
fig|80869.177.peg.4538	T4S	5'-nucleotidase [*Paracidovorax citrulli*]
fig|80869.177.peg.1356	T4S	30S ribosomal protein S4 [*Paracidovorax citrulli*]
fig|80869.177.peg.3273	T4S	adenylate kinase [*Paracidovorax citrulli*]
fig|80869.177.peg.2739	T4S	hypothetical protein [*Paracidovorax citrulli*]
fig|80869.177.peg.3900	T4S	hypothetical protein [*Paracidovorax citrulli*]
fig|80869.177.peg.4089	T4S	type IV pilin protein [*Paracidovorax citrulli*]
fig|80869.177.peg.280	T4S	hypothetical protein [*Paracidovorax citrulli*]
fig|80869.177.peg.4425	T4S	BON domain-containing protein [*Paracidovorax citrulli*]
fig|80869.177.peg.3250	T4S	MetQ/NlpA family ABC transporter substrate-binding protein [*Paracidovorax citrulli*]
fig|80869.177.peg.422	T4S	Bug family tripartite tricarboxylate transporter substrate binding protein [*Paracidovorax citrulli*]
fig|80869.177.peg.2497	T4S	Bug family tripartite tricarboxylate transporter substrate binding protein [*Paracidovorax citrulli*]
fig|80869.177.peg.3256	T4S	sulfate ABC transporter substrate-binding protein [*Paracidovorax citrulli*]
fig|80869.177.peg.4307	T4S	hypothetical protein [*Paracidovorax citrulli*]
fig|80869.177.peg.557	T4S	OmpA family protein [*Paracidovorax citrulli*]

Prot	Pred	Description
fig|80869.177.peg.2417	T4S	glycoside hydrolase family protein [*Paracidovorax citrulli*]
fig|80869.177.peg.1116	T4S	HTH-type transcriptional regulator GltC [*Paracidovorax citrulli*]
fig|80869.177.peg.3931	T4S	sigma-54-dependent transcriptional regulator [*Paracidovorax citrulli*]
fig|80869.177.peg.4539	T4S	EF-hand domain-containing protein [*Paracidovorax citrulli*]

*P_citrulli*_M6

Prot	Pred	Description
fig|80869.171.peg.2800	T3S	Ig domain protein, group 1 domain protein [*Paracidovorax citrulli* AAC00-1]
fig|80869.171.peg.4217	T3S	hypothetical protein [*Paracidovorax citrulli*]
fig|80869.171.peg.4328	T3S	major facilitator superfamily MFS_1 [*Paracidovorax citrulli* AAC00-1]
fig|80869.171.peg.245	T3S	acyl-CoA thioesterase [*Paracidovorax citrulli*]
fig|80869.171.peg.160	T3S	STY0301 family protein [*Paracidovorax citrulli*]
fig|80869.171.peg.4125	T3S	ABC transporter ATP-binding protein [*Paracidovorax citrulli*]
fig|80869.171.peg.51	T3S	Ku protein [*Paracidovorax citrulli*]
fig|80869.171.peg.945	T3S	bacterioferritin [*Paracidovorax citrulli*]
fig|80869.171.peg.3499	T3S	terminase small subunit [*Paracidovorax citrulli*]
fig|80869.171.peg.1030	T3S	type III secretion system chaperone [*Paracidovorax citrulli*]
fig|80869.171.peg.1031	T3S	hypothetical protein [*Paracidovorax citrulli*]
fig|80869.171.peg.1058	T3S	luciferase family protein [*Paracidovorax citrulli* AAC00-1]
fig|80869.171.peg.1071	T3S	hypothetical protein [*Paracidovorax citrulli*]
fig|80869.171.peg.1084	T3S	ABC transporter permease [*Paracidovorax citrulli*]
fig|80869.171.peg.1085	T3S	ABC transporter permease [*Paracidovorax citrulli*]
fig|80869.171.peg.1099	T3S	(2Fe-2S)-binding protein [*Paracidovorax citrulli*]
fig|80869.171.peg.1100	T3S	molybdenum cofactor cytidylyltransferase [*Paracidovorax citrulli*]
fig|80869.171.peg.1105	T3S	hypothetical protein [*Paracidovorax citrulli*]
fig|80869.171.peg.1118	T3S	alpha/beta hydrolase [*Paracidovorax citrulli*]
fig|80869.171.peg.1138	T3S	murein DD-endopeptidase MepM/ murein hydrolase activator NlpD [*Paracidovorax citrulli*]
fig|80869.171.peg.1148	T3S	septal ring lytic transglycosylase RlpA family protein [*Paracidovorax citrulli*]
fig|80869.171.peg.1161	T3S	hypothetical protein [*Paracidovorax citrulli*]
fig|80869.171.peg.1168	T3S	MULTISPECIES: TRAP transporter small permease [Paracidovorax]
fig|80869.171.peg.1220	T3S	rhodanese-like domain-containing protein [*Paracidovorax citrulli*]
fig|80869.171.peg.123	T3S	PAS domain S-box protein [*Paracidovorax citrulli*]
fig|80869.171.peg.1236	T3S	GNAT family N-acetyltransferase [*Paracidovorax citrulli*]
fig|80869.171.peg.1248	T3S	ABC transporter permease [*Paracidovorax citrulli*]
fig|80869.171.peg.1253	T3S	Metallo-beta-lactamase superfamily protein [*Paracidovorax citrulli*]
fig|80869.171.peg.1265	T3S	glycoside hydrolase family 15 protein [*Paracidovorax citrulli*]
fig|80869.171.peg.1285	T3S	hypothetical protein [*Paracidovorax citrulli*]
fig|80869.171.peg.1313	T3S	ABC transporter permease [*Paracidovorax citrulli*]
fig|80869.171.peg.1385	T3S	ABC transporter ATP-binding protein [*Paracidovorax citrulli*]
fig|80869.171.peg.1442	T3S	chemoreceptor glutamine deamidase CheD [*Paracidovorax citrulli*]
fig|80869.171.peg.1443	T3S	CheR family methyltransferase [*Paracidovorax citrulli*]
fig|80869.171.peg.1467	T3S	flagellin [*Paracidovorax citrulli*]
fig|80869.171.peg.1468	T3S	flagellin [*Paracidovorax citrulli*]
fig|80869.171.peg.1471	T3S	conserved hypothetical protein [*Paracidovorax citrulli* AAC00-1]
fig|80869.171.peg.1484	T3S	hypothetical protein [*Paracidovorax citrulli*]

Prot	Pred	Description
fig|80869.171.peg.1489	T3S	flagellar hook assembly protein FlgD [*Paracidovorax citrulli*]
fig|80869.171.peg.1506	T3S	DMT family transporter [*Paracidovorax citrulli*]
fig|80869.171.peg.1512	T3S	hypothetical protein [*Paracidovorax citrulli*]
fig|80869.171.peg.1519	T3S	PepSY-associated TM helix domain-containing protein [*Paracidovorax citrulli*]
fig|80869.171.peg.1543	T3S	MarR family winged helix-turn-helix transcriptional regulator [*Paracidovorax citrulli*]
fig|80869.171.peg.1566	T3S	hypothetical protein APS58_1500 [*Paracidovorax citrulli*]
fig|80869.171.peg.1586	T3S	PhaM family polyhydroxyalkanoate granule multifunctional regulatory protein [*Paracidovorax citrulli*]
fig|80869.171.peg.1607	T3S	sensor histidine kinase [*Paracidovorax citrulli*]
fig|80869.171.peg.1609	T3S	16S rRNA (cytosine(967)-C(5))-methyltransferase RsmB [*Paracidovorax citrulli*]
fig|80869.171.peg.1665	T3S	phosphatase PAP2 family protein [*Paracidovorax citrulli*]
fig|80869.171.peg.1668	T3S	nucleotidyltransferase family protein [*Paracidovorax citrulli*]
fig|80869.171.peg.1683	T3S	gamma-glutamyl-gamma-aminobutyrate hydrolase family protein [*Paracidovorax citrulli*]
fig|80869.171.peg.170	T3S	KGG domain-containing protein [*Paracidovorax citrulli*]
fig|80869.171.peg.1702	T3S	transcriptional regulator, TetR family [*Paracidovorax citrulli* AAC00-1]
fig|80869.171.peg.1760	T3S	CerR family C-terminal domain-containing protein [*Paracidovorax citrulli*]
fig|80869.171.peg.1803	T3S	putative amino-acid metabolite efflux pump [*Paracidovorax citrulli*]
fig|80869.171.peg.1805	T3S	DUF1800 family protein [*Paracidovorax citrulli*]
fig|80869.171.peg.1820	T3S	MBL fold metallo-hydrolase [*Paracidovorax citrulli*]
fig|80869.171.peg.1833	T3S	response regulator [*Paracidovorax citrulli*]
fig|80869.171.peg.1844	T3S	flagellar basal body protein [*Paracidovorax citrulli*]
fig|80869.171.peg.1845	T3S	LysR family transcriptional regulator [*Paracidovorax citrulli*]
fig|80869.171.peg.1888	T3S	KdsC family phosphatase [*Paracidovorax citrulli*]
fig|80869.171.peg.1892	T3S	PepSY domain-containing protein [*Paracidovorax citrulli*]
fig|80869.171.peg.1904	T3S	DNA topoisomerase (ATP-hydrolyzing) subunit B [*Paracidovorax citrulli*]
fig|80869.171.peg.1926	T3S	2-isopropylmalate synthase [*Paracidovorax citrulli*]
fig|80869.171.peg.1955	T3S	ATP-binding cassette domain-containing protein [*Paracidovorax citrulli*]
fig|80869.171.peg.1978	T3S	sirohydrochlorin chelatase [*Paracidovorax citrulli*]
fig|80869.171.peg.1982	T3S	precorrin-2 C(20)-methyltransferase [*Paracidovorax citrulli*]
fig|80869.171.peg.2015	T3S	hotdog fold thioesterase [*Paracidovorax citrulli*]
fig|80869.171.peg.2019	T3S	hypothetical protein [*Paracidovorax citrulli*]
fig|80869.171.peg.2027	T3S	MBL fold metallo-hydrolase [*Paracidovorax citrulli*]
fig|80869.171.peg.2031	T3S	5-demethoxyubiquinol-8 5-hydroxylase UbiM [*Paracidovorax citrulli*]
fig|80869.171.peg.2090	T3S	aromatic ring-hydroxylating dioxygenase subunit alpha [*Paracidovorax citrulli*]
fig|80869.171.peg.2096	T3S	hypothetical protein [*Paracidovorax citrulli*]
fig|80869.171.peg.2102	T3S	Cupin 2, conserved barrel domain protein [*Paracidovorax citrulli* AAC00-1]
fig|80869.171.peg.2122	T3S	transcriptional regulator CynR [*Paracidovorax citrulli*]
fig|80869.171.peg.2154	T3S	GNAT family N-acetyltransferase [Paracidovorax avenae]
fig|80869.171.peg.217	T3S	MFS transporter [*Paracidovorax citrulli*]
fig|80869.171.peg.2206	T3S	AmpG family muropeptide MFS transporter [*Paracidovorax citrulli*]
fig|80869.171.peg.2209	T3S	DME family drug/metabolite transporter [*Paracidovorax citrulli*]
fig|80869.171.peg.2211	T3S	SLC13 family permease [*Paracidovorax citrulli*]
fig|80869.171.peg.2230	T3S	amino acid ABC transporter ATP-binding protein [*Paracidovorax citrulli*]
fig|80869.171.peg.2238	T3S	MULTISPECIES: ABC transporter ATP-binding protein [Paracidovorax]
fig|80869.171.peg.2253	T3S	DHA2 family efflux MFS transporter permease subunit [*Paracidovorax citrulli*]

Prot	Pred	Description
fig|80869.171.peg.2254	T3S	efflux RND transporter periplasmic adaptor subunit [*Paracidovorax citrulli*]
fig|80869.171.peg.2292	T3S	potassium-transporting ATPase subunit KdpB [*Paracidovorax citrulli*]
fig|80869.171.peg.2317	T3S	NEL-type E3 ubiquitin ligase domain-containing protein [*Paracidovorax citrulli*]
fig|80869.171.peg.2326	T3S	N-acetylneuraminate epimerase [*Paracidovorax citrulli*]
fig|80869.171.peg.2358	T3S	TspO/MBR family protein [*Paracidovorax citrulli*]
fig|80869.171.peg.2363	T3S	hypothetical protein [*Paracidovorax citrulli*]
fig|80869.171.peg.2370	T3S	DUF4139 domain-containing protein [*Paracidovorax citrulli*]
fig|80869.171.peg.2397	T3S	pectate lyase [*Paracidovorax citrulli*]
fig|80869.171.peg.2400	T3S	hypothetical protein [*Paracidovorax citrulli*]
fig|80869.171.peg.2401	T3S	hypothetical protein [*Paracidovorax citrulli*]
fig|80869.171.peg.2413	T3S	hypothetical protein [*Paracidovorax citrulli*]
fig|80869.171.peg.2429	T3S	uracil-DNA glycosylase [*Paracidovorax citrulli*]
fig|80869.171.peg.243	T3S	uroporphyrinogen-III C-methyltransferase [*Paracidovorax citrulli*]
fig|80869.171.peg.2445	T3S	hypothetical protein APS58_2360 [*Paracidovorax citrulli*]
fig|80869.171.peg.2446	T3S	glycosyltransferase family 39 protein [*Paracidovorax citrulli*]
fig|80869.171.peg.2447	T3S	pyrroline-5-carboxylate reductase [*Paracidovorax citrulli*]
fig|80869.171.peg.2491	T3S	FAD-linked oxidase C-terminal domain-containing protein [*Paracidovorax citrulli*]
fig|80869.171.peg.2504	T3S	transcriptional regulator, AraC family [*Paracidovorax citrulli* AAC00-1]
fig|80869.171.peg.2506	T3S	NarK family nitrate/nitrite MFS transporter [*Paracidovorax citrulli*]
fig|80869.171.peg.2533	T3S	DUF2169 domain-containing protein [*Paracidovorax citrulli*]
fig|80869.171.peg.2565	T3S	putative zinc protease protein [*Paracidovorax citrulli* AAC00-1]
fig|80869.171.peg.2566	T3S	DNA-3-methyladenine glycosylase I [*Paracidovorax citrulli*]
fig|80869.171.peg.2581	T3S	M48 family metallopeptidase [*Paracidovorax citrulli*]
fig|80869.171.peg.2588	T3S	chromate efflux transporter [*Paracidovorax citrulli*]
fig|80869.171.peg.2599	T3S	CobW family GTP-binding protein [*Paracidovorax citrulli*]
fig|80869.171.peg.2646	T3S	3-deoxy-7-phosphoheptulonate synthase [*Paracidovorax citrulli*]
fig|80869.171.peg.265	T3S	succinate-semialdehyde dehydrogenase/glutarate-semialdehyde dehydrogenase [*Paracidovorax citrulli*]
fig|80869.171.peg.2658	T3S	ribonucleoside-diphosphate reductase subunit alpha [*Paracidovorax citrulli*]
fig|80869.171.peg.2692	T3S	hydroxymethylpyrimidine/phosphomethylpyrimidine kinase [*Paracidovorax citrulli*]
fig|80869.171.peg.2701	T3S	Holliday junction resolvase RuvX [*Paracidovorax citrulli*]
fig|80869.171.peg.2734	T3S	ABC transporter ATP-binding protein [*Paracidovorax citrulli*]
fig|80869.171.peg.2770	T3S	peptidoglycan-binding domain-containing protein [*Paracidovorax citrulli*]
fig|80869.171.peg.2794	T3S	penicillin-binding protein 1A [*Paracidovorax citrulli*]
fig|80869.171.peg.2804	T3S	protein of unknown function DUF1415 [*Paracidovorax citrulli* AAC00-1]
fig|80869.171.peg.2841	T3S	PelD GGDEF domain-containing protein [*Paracidovorax citrulli*]
fig|80869.171.peg.2886	T3S	DMT family transporter [*Paracidovorax citrulli*]
fig|80869.171.peg.2905	T3S	GntR family transcriptional regulator [*Paracidovorax citrulli*]
fig|80869.171.peg.2907	T3S	NCS1 family nucleobase:cation symporter-1 [*Paracidovorax citrulli*]
fig|80869.171.peg.2920	T3S	glycerate kinase [*Paracidovorax citrulli*]
fig|80869.171.peg.2940	T3S	ABC transporter ATP-binding protein [*Paracidovorax citrulli*]
fig|80869.171.peg.2956	T3S	neutral zinc metallopeptidase [*Paracidovorax citrulli*]
fig|80869.171.peg.2967	T3S	tryptophan 2,3-dioxygenase [*Paracidovorax citrulli*]
fig|80869.171.peg.3016	T3S	Phytochrome-like protein cph2 [*Paracidovorax citrulli*]
fig|80869.171.peg.3017	T3S	lysine--tRNA ligase [*Paracidovorax citrulli*]
fig|80869.171.peg.3024	T3S	LON peptidase substrate-binding domain-containing protein [*Paracidovorax citrulli*]

Prot	Pred	Description
fig|80869.171.peg.3029	T3S	phosphoribosylanthranilate isomerase [*Paracidovorax citrulli* AAC00-1]
fig|80869.171.peg.3042	T3S	nucleotide exchange factor GrpE [*Paracidovorax citrulli*]
fig|80869.171.peg.3070	T3S	molybdopterin converting factor subunit 1 [*Paracidovorax citrulli*]
fig|80869.171.peg.3083	T3S	hypothetical protein [*Paracidovorax citrulli*]
fig|80869.171.peg.309	T3S	efflux transporter outer membrane subunit [*Paracidovorax citrulli*]
fig|80869.171.peg.3102	T3S	MULTISPECIES: NADH-quinone oxidoreductase subunit NuoI [Comamonadaceae]
fig|80869.171.peg.311	T3S	multidrug efflux pump [*Paracidovorax citrulli*]
fig|80869.171.peg.3110	T3S	ABC transporter transmembrane domain-containing protein [*Paracidovorax citrulli*]
fig|80869.171.peg.312	T3S	prepilin-type N-terminal cleavage/methylation domain-containing protein [Polaromonas sp.]
fig|80869.171.peg.3128	T3S	winged helix-turn-helix transcriptional regulator [*Paracidovorax citrulli*]
fig|80869.171.peg.3137	T3S	N-acetylmuramoyl-L-alanine amidase [*Paracidovorax citrulli*]
fig|80869.171.peg.3148	T3S	EAL and HDOD domain-containing protein [*Paracidovorax citrulli*]
fig|80869.171.peg.3152	T3S	hypothetical protein [*Paracidovorax citrulli*]
fig|80869.171.peg.3178	T3S	pyridoxal kinase PdxY [*Paracidovorax citrulli*]
fig|80869.171.peg.3184	T3S	DNA internalization-related competence protein ComEC/Rec2 [*Paracidovorax citrulli*]
fig|80869.171.peg.3192	T3S	uncharacterized protein DUF2132 [*Paracidovorax citrulli*]
fig|80869.171.peg.3225	T3S	CaiB/BaiF CoA transferase family protein [*Paracidovorax citrulli*]
fig|80869.171.peg.323	T3S	TOBE domain-containing protein [*Paracidovorax citrulli*]
fig|80869.171.peg.3236	T3S	ketopantoate reductase [*Paracidovorax citrulli*]
fig|80869.171.peg.3251	T3S	protein-L-isoaspartate(D-aspartate) O-methyltransferase [*Paracidovorax citrulli*]
fig|80869.171.peg.3267	T3S	GTPase HflX [*Paracidovorax citrulli*]
fig|80869.171.peg.328	T3S	BCCT family transporter [*Paracidovorax citrulli*]
fig|80869.171.peg.3292	T3S	DUF2069 domain-containing protein [*Paracidovorax citrulli*]
fig|80869.171.peg.3295	T3S	farnesyl-diphosphate farnesyltransferase [*Paracidovorax citrulli* AAC00-1]
fig|80869.171.peg.331	T3S	alpha-1,4-glucan--maltose-1-phosphate maltosyltransferase [*Paracidovorax citrulli*]
fig|80869.171.peg.3337	T3S	glycine betaine/L-proline ABC transporter permease ProW [*Paracidovorax citrulli*]
fig|80869.171.peg.3355	T3S	malonate--CoA ligase [*Paracidovorax citrulli*]
fig|80869.171.peg.3359	T3S	heavy metal translocating P-type ATPase [*Paracidovorax citrulli*]
fig|80869.171.peg.3389	T3S	SDR family NAD(P)-dependent oxidoreductase [*Paracidovorax citrulli*]
fig|80869.171.peg.342	T3S	exodeoxyribonuclease VII small subunit [*Paracidovorax citrulli*]
fig|80869.171.peg.3435	T3S	type 1 glutamine amidotransferase domain-containing protein [*Paracidovorax citrulli*]
fig|80869.171.peg.2800	T4S	Ig domain protein, group 1 domain protein [*Paracidovorax citrulli* AAC00-1]
fig|80869.171.peg.4217	T4S	hypothetical protein [*Paracidovorax citrulli*]
fig|80869.171.peg.4328	T4S	major facilitator superfamily MFS_1 [*Paracidovorax citrulli* AAC00-1]
fig|80869.171.peg.245	T4S	acyl-CoA thioesterase [*Paracidovorax citrulli*]
fig|80869.171.peg.160	T4S	STY0301 family protein [*Paracidovorax citrulli*]
fig|80869.171.peg.4125	T4S	ABC transporter ATP-binding protein [*Paracidovorax citrulli*]
fig|80869.171.peg.51	T4S	Ku protein [*Paracidovorax citrulli*]
fig|80869.171.peg.945	T4S	bacterioferritin [*Paracidovorax citrulli*]
fig|80869.171.peg.3499	T4S	terminase small subunit [*Paracidovorax citrulli*]
fig|80869.171.peg.3077	T4S	molybdenum cofactor biosynthesis protein MoaE [*Paracidovorax citrulli*]
fig|80869.171.peg.3916	T4S	hypothetical protein [*Paracidovorax citrulli*]
fig|80869.171.peg.3449	T4S	isoaspartyl peptidase/L-asparaginase family protein [*Paracidovorax citrulli*]

Prot	Pred	Description
fig|80869.171.peg.206	T4S	RNA recognition motif domain-containing protein [*Paracidovorax citrulli*]
fig|80869.171.peg.4013	T4S	hypothetical protein Aave_3072 [*Paracidovorax citrulli* AAC00-1]
fig|80869.171.peg.4386	T4S	DUF1795 domain-containing protein [*Paracidovorax citrulli*]
fig|80869.171.peg.9	T4S	MULTISPECIES: amino-acid N-acetyltransferase [Paracidovorax]
fig|80869.171.peg.1679	T4S	DUF924 family protein [*Paracidovorax citrulli*]
fig|80869.171.peg.1860	T4S	hypothetical protein [*Paracidovorax citrulli*]
fig|80869.171.peg.1617	T4S	50S ribosomal protein L11 [*Paracidovorax citrulli*]
fig|80869.171.peg.39	T4S	hypothetical protein [*Paracidovorax citrulli*]
fig|80869.171.peg.4303	T4S	conserved hypothetical protein [*Paracidovorax citrulli* AAC00-1]
fig|80869.171.peg.3255	T4S	SMC-Scp complex subunit ScpB [*Paracidovorax citrulli*]
fig|80869.171.peg.652	T4S	PP2C family protein-serine/threonine phosphatase [*Paracidovorax citrulli*]
fig|80869.171.peg.1999	T4S	hypothetical protein [*Paracidovorax citrulli*]
fig|80869.171.peg.1595	T4S	cryptochrome/photolyase family protein [*Paracidovorax citrulli*]
fig|80869.171.peg.3154	T4S	phosphopyruvate hydratase [*Paracidovorax citrulli*]
fig|80869.171.peg.997	T4S	glutathione-regulated potassium-efflux system protein KefC [*Paracidovorax citrulli*]
fig|80869.171.peg.596	T4S	hypothetical protein [*Paracidovorax citrulli*]
fig|80869.171.peg.3813	T4S	hypothetical protein [*Paracidovorax citrulli*]
fig|80869.171.peg.3620	T4S	AAA family ATPase [*Paracidovorax citrulli*]
fig|80869.171.peg.1378	T4S	DUF3025 domain-containing protein [*Paracidovorax citrulli*]
fig|80869.171.peg.653	T4S	serine/threonine protein kinase [*Paracidovorax citrulli*]
fig|80869.171.peg.869	T4S	hypothetical protein [*Paracidovorax citrulli*]
fig|80869.171.peg.279	T4S	inositol monophosphatase family protein [*Paracidovorax citrulli*]
fig|80869.171.peg.1300	T4S	N-acetylmuramoyl-L-alanine amidase [*Paracidovorax citrulli*]
fig|80869.171.peg.2437	T4S	AraC family transcriptional regulator [*Paracidovorax citrulli*]
fig|80869.171.peg.3756	T4S	hypothetical protein [*Paracidovorax citrulli*]
fig|80869.171.peg.1714	T4S	Fic family protein [*Paracidovorax citrulli*]
fig|80869.171.peg.851	T4S	4-hydroxy-3-methylbut-2-enyl diphosphate reductase [*Paracidovorax citrulli*]
fig|80869.171.peg.3697	T4S	SsrA-binding protein SmpB [*Paracidovorax citrulli*]
fig|80869.171.peg.2308	T4S	F0F1 ATP synthase subunit gamma [*Paracidovorax citrulli*]
fig|80869.171.peg.3282	T4S	hypothetical protein [*Paracidovorax citrulli*]
fig|80869.171.peg.4485	T4S	transglycosylase SLT domain-containing protein [*Paracidovorax citrulli*]
fig|80869.171.peg.2416	T4S	type III secretion system outer membrane ring subunit SctC [*Paracidovorax citrulli*]
fig|80869.171.peg.3517	T4S	hypothetical protein [*Paracidovorax citrulli*]
fig|80869.171.peg.3399	T4S	XopE/AvrPphe family type III secretion system effector [*Paracidovorax citrulli*]
fig|80869.171.peg.1060	T4S	5'-nucleotidase [*Paracidovorax citrulli*]
fig|80869.171.peg.2472	T4S	30S ribosomal protein S4 [*Paracidovorax citrulli*]
fig|80869.171.peg.4578	T4S	adenylate kinase [*Paracidovorax citrulli*]
fig|80869.171.peg.3832	T4S	hypothetical protein [*Paracidovorax citrulli*]
fig|80869.171.peg.434	T4S	hypothetical protein [*Paracidovorax citrulli*]
fig|80869.171.peg.622	T4S	type IV pilin protein [*Paracidovorax citrulli*]
fig|80869.171.peg.946	T4S	BON domain-containing protein [*Paracidovorax citrulli*]
fig|80869.171.peg.23	T4S	MetQ/NlpA family ABC transporter substrate-binding protein [*Paracidovorax citrulli*]
fig|80869.171.peg.1542	T4S	Bug family tripartite tricarboxylate transporter substrate binding protein [*Paracidovorax citrulli*]
fig|80869.171.peg.3614	T4S	Bug family tripartite tricarboxylate transporter substrate binding protein [*Paracidovorax citrulli*]

Prot	Pred	Description
fig|80869.171.peg.17	T4S	sulfate ABC transporter substrate-binding protein [*Paracidovorax citrulli*]
fig|80869.171.peg.828	T4S	hypothetical protein [*Paracidovorax citrulli*]
fig|80869.171.peg.1678	T4S	OmpA family protein [*Paracidovorax citrulli*]
fig|80869.171.peg.3535	T4S	glycoside hydrolase family protein [*Paracidovorax citrulli*]
fig|80869.171.peg.2234	T4S	HTH-type transcriptional regulator GltC [*Paracidovorax citrulli*]
fig|80869.171.peg.465	T4S	sigma-54-dependent transcriptional regulator [*Paracidovorax citrulli*]
fig|80869.171.peg.1061	T4S	EF-hand domain-containing protein [*Paracidovorax citrulli*]

*P_citrulli*_Pslb65

Prot	Pred	Description
fig|80869.182.peg.2562	T3S	Ig domain protein, group 1 domain protein [*Paracidovorax citrulli* AAC00-1]
fig|80869.182.peg.3071	T3S	hypothetical protein [*Paracidovorax citrulli*]
fig|80869.182.peg.599	T3S	major facilitator superfamily MFS_1 [*Paracidovorax citrulli* AAC00-1]
fig|80869.182.peg.214	T3S	acyl-CoA thioesterase [*Paracidovorax citrulli*]
fig|80869.182.peg.756	T3S	STY0301 family protein [*Paracidovorax citrulli*]
fig|80869.182.peg.3841	T3S	ABC transporter ATP-binding protein [*Paracidovorax citrulli*]
fig|80869.182.peg.865	T3S	Ku protein [*Paracidovorax citrulli*]
fig|80869.182.peg.3974	T3S	hypothetical protein [*Paracidovorax citrulli*]
fig|80869.182.peg.2917	T3S	bacterioferritin [*Paracidovorax citrulli*]
fig|80869.182.peg.1328	T3S	terminase small subunit [*Paracidovorax citrulli*]
fig|80869.182.peg.1045	T3S	SAM-dependent methyltransferase [*Paracidovorax citrulli*]
fig|80869.182.peg.1083	T3S	urease accessory protein UreG [*Paracidovorax citrulli*]
fig|80869.182.peg.1084	T3S	urease accessory protein [*Paracidovorax citrulli*]
fig|80869.182.peg.1124	T3S	phosphoenolpyruvate carboxylase [*Paracidovorax citrulli*]
fig|80869.182.peg.1125	T3S	YccS/YhfK family membrane protein [*Paracidovorax citrulli*]
fig|80869.182.peg.1128	T3S	PilZ domain-containing protein [*Paracidovorax citrulli*]
fig|80869.182.peg.1144	T3S	DNA topoisomerase (ATP-hydrolyzing) subunit B [*Paracidovorax citrulli*]
fig|80869.182.peg.1155	T3S	PepSY domain-containing protein [*Paracidovorax citrulli*]
fig|80869.182.peg.1159	T3S	KdsC family phosphatase [*Paracidovorax citrulli*]
fig|80869.182.peg.12	T3S	AI-2E family transporter [*Paracidovorax citrulli*]
fig|80869.182.peg.1204	T3S	LysR family transcriptional regulator [*Paracidovorax citrulli*]
fig|80869.182.peg.1205	T3S	flagellar basal body protein [*Paracidovorax citrulli*]
fig|80869.182.peg.1216	T3S	response regulator [*Paracidovorax citrulli*]
fig|80869.182.peg.1228	T3S	MBL fold metallo-hydrolase [*Paracidovorax citrulli*]
fig|80869.182.peg.1243	T3S	DUF1800 family protein [*Paracidovorax citrulli*]
fig|80869.182.peg.1245	T3S	putative amino-acid metabolite efflux pump [*Paracidovorax citrulli*]
fig|80869.182.peg.128	T3S	hypothetical protein [*Paracidovorax citrulli*]
fig|80869.182.peg.1289	T3S	CerR family C-terminal domain-containing protein [*Paracidovorax citrulli*]
fig|80869.182.peg.1312	T3S	hypothetical protein Aave_1685 [*Paracidovorax citrulli* AAC00-1]
fig|80869.182.peg.1315	T3S	hypothetical protein [*Paracidovorax citrulli*]
fig|80869.182.peg.1325	T3S	hypothetical protein [*Paracidovorax citrulli*]
fig|80869.182.peg.1404	T3S	ATP synthase F1 subunit epsilon [*Paracidovorax citrulli*]
fig|80869.182.peg.1409	T3S	P1 family peptidase [*Paracidovorax citrulli*]
fig|80869.182.peg.1419	T3S	YitT family protein [*Paracidovorax citrulli*]
fig|80869.182.peg.1423	T3S	MlaD family protein [*Paracidovorax citrulli*]

Prot	Pred	Description
fig|80869.182.peg.1436	T3S	UDP-3-O-(3-hydroxymyristoyl)glucosamine N-acyltransferase [*Paracidovorax citrulli*]
fig|80869.182.peg.1470	T3S	glutamate--tRNA ligase [*Paracidovorax citrulli*]
fig|80869.182.peg.1471	T3S	DUF1624 domain-containing protein [*Paracidovorax citrulli*]
fig|80869.182.peg.1474	T3S	DUF72 domain-containing protein [*Paracidovorax citrulli*]
fig|80869.182.peg.1492	T3S	lipid A export permease/ATP-binding protein MsbA [*Paracidovorax citrulli*]
fig|80869.182.peg.1496	T3S	ThiF family adenylyltransferase [*Paracidovorax citrulli*]
fig|80869.182.peg.1565	T3S	aromatic ring-hydroxylating dioxygenase subunit alpha [*Paracidovorax citrulli*]
fig|80869.182.peg.1577	T3S	Cupin 2, conserved barrel domain protein [*Paracidovorax citrulli* AAC00-1]
fig|80869.182.peg.1597	T3S	transcriptional regulator CynR [*Paracidovorax citrulli*]
fig|80869.182.peg.1623	T3S	5-demethoxyubiquinol-8 5-hydroxylase UbiM [*Paracidovorax citrulli*]
fig|80869.182.peg.1627	T3S	MBL fold metallo-hydrolase [*Paracidovorax citrulli*]
fig|80869.182.peg.1635	T3S	hypothetical protein [*Paracidovorax citrulli*]
fig|80869.182.peg.1639	T3S	hotdog fold thioesterase [*Paracidovorax citrulli*]
fig|80869.182.peg.166	T3S	Conjugal transfer protein TraX [*Paracidovorax citrulli*]
fig|80869.182.peg.1693	T3S	hypothetical protein [*Paracidovorax citrulli*]
fig|80869.182.peg.1697	T3S	protein of unknown function DUF1653 [*Paracidovorax citrulli* AAC00-1]
fig|80869.182.peg.1712	T3S	isoleucine--tRNA ligase [*Paracidovorax citrulli*]
fig|80869.182.peg.177	T3S	helix-turn-helix transcriptional regulator [*Paracidovorax citrulli*]
fig|80869.182.peg.1774	T3S	DUF2169 domain-containing protein [*Paracidovorax citrulli*]
fig|80869.182.peg.1794	T3S	GNAT family N-acetyltransferase [Paracidovorax avenae]
fig|80869.182.peg.18	T3S	SfnB family sulfur acquisition oxidoreductase [*Paracidovorax citrulli*]
fig|80869.182.peg.1876	T3S	AmpG family muropeptide MFS transporter [*Paracidovorax citrulli*]
fig|80869.182.peg.1879	T3S	DME family drug/metabolite transporter [*Paracidovorax citrulli*]
fig|80869.182.peg.1881	T3S	SLC13 family permease [*Paracidovorax citrulli*]
fig|80869.182.peg.19	T3S	SfnB family sulfur acquisition oxidoreductase [*Paracidovorax citrulli*]
fig|80869.182.peg.1900	T3S	amino acid ABC transporter ATP-binding protein [*Paracidovorax citrulli*]
fig|80869.182.peg.1909	T3S	MULTISPECIES: ABC transporter ATP-binding protein [Paracidovorax]
fig|80869.182.peg.1924	T3S	DHA2 family efflux MFS transporter permease subunit [*Paracidovorax citrulli*]
fig|80869.182.peg.1925	T3S	efflux RND transporter periplasmic adaptor subunit [*Paracidovorax citrulli*]
fig|80869.182.peg.194	T3S	hypothetical protein [*Paracidovorax citrulli*]
fig|80869.182.peg.1963	T3S	potassium-transporting ATPase subunit KdpB [*Paracidovorax citrulli*]
fig|80869.182.peg.1987	T3S	NEL-type E3 ubiquitin ligase domain-containing protein [*Paracidovorax citrulli*]
fig|80869.182.peg.1996	T3S	N-acetylneuraminate epimerase [*Paracidovorax citrulli*]
fig|80869.182.peg.202	T3S	thioredoxin family protein [*Paracidovorax citrulli*]
fig|80869.182.peg.2030	T3S	TspO/MBR family protein [*Paracidovorax citrulli*]
fig|80869.182.peg.2035	T3S	hypothetical protein [*Paracidovorax citrulli*]
fig|80869.182.peg.204	T3S	cytochrome c553-like protein [*Paracidovorax citrulli* AAC00-1]
fig|80869.182.peg.2042	T3S	DUF4139 domain-containing protein [*Paracidovorax citrulli*]
fig|80869.182.peg.2069	T3S	pectate lyase [*Paracidovorax citrulli*]
fig|80869.182.peg.2072	T3S	hypothetical protein [*Paracidovorax citrulli*]
fig|80869.182.peg.2073	T3S	hypothetical protein [*Paracidovorax citrulli*]
fig|80869.182.peg.2083	T3S	hypothetical protein [*Paracidovorax citrulli*]
fig|80869.182.peg.2098	T3S	uracil-DNA glycosylase [*Paracidovorax citrulli*]
fig|80869.182.peg.2113	T3S	hypothetical protein APS58_2360 [*Paracidovorax citrulli*]

Prot	Pred	Description
fig|80869.182.peg.2115	T3S	pyrroline-5-carboxylate reductase [*Paracidovorax citrulli*]
fig|80869.182.peg.216	T3S	uroporphyrinogen-III C-methyltransferase [*Paracidovorax citrulli*]
fig|80869.182.peg.2160	T3S	FAD-linked oxidase C-terminal domain-containing protein [*Paracidovorax citrulli*]
fig|80869.182.peg.2173	T3S	transcriptional regulator, AraC family [*Paracidovorax citrulli* AAC00-1]
fig|80869.182.peg.2175	T3S	NarK family nitrate/nitrite MFS transporter [*Paracidovorax citrulli*]
fig|80869.182.peg.2265	T3S	TonB-dependent receptor family protein [*Paracidovorax citrulli*]
fig|80869.182.peg.2275	T3S	D-serine/D-alanine/glycine transporter [*Paracidovorax citrulli*]
fig|80869.182.peg.2276	T3S	hypothetical protein [*Paracidovorax citrulli*]
fig|80869.182.peg.2291	T3S	Polyphosphate kinase [*Paracidovorax citrulli* AAC00-1]
fig|80869.182.peg.2296	T3S	phosphate ABC transporter permease PstC [*Paracidovorax citrulli*]
fig|80869.182.peg.2323	T3S	hypothetical protein [*Paracidovorax citrulli*]
fig|80869.182.peg.2335	T3S	gephyrin-like molybdotransferase Glp [*Paracidovorax citrulli*]
fig|80869.182.peg.2338	T3S	GTP cyclohydrolase subunit MoaA [*Paracidovorax citrulli* AAC00-1]
fig|80869.182.peg.2349	T3S	translesion DNA synthesis-associated protein ImuA [*Paracidovorax citrulli*]
fig|80869.182.peg.2353	T3S	pseudouridine synthase [*Paracidovorax citrulli*]
fig|80869.182.peg.2388	T3S	pyridoxamine 5'-phosphate oxidase [*Paracidovorax citrulli*]
fig|80869.182.peg.2399	T3S	transcriptional repressor [*Paracidovorax citrulli*]
fig|80869.182.peg.24	T3S	exodeoxyribonuclease VII small subunit [*Paracidovorax citrulli*]
fig|80869.182.peg.2401	T3S	TetR/AcrR family transcriptional regulator [*Paracidovorax citrulli*]
fig|80869.182.peg.242	T3S	MFS transporter [*Paracidovorax citrulli*]
fig|80869.182.peg.2462	T3S	transcriptional regulator, TetR family [*Paracidovorax citrulli* AAC00-1]
fig|80869.182.peg.2522	T3S	PelD GGDEF domain-containing protein [*Paracidovorax citrulli*]
fig|80869.182.peg.2558	T3S	protein of unknown function DUF1415 [*Paracidovorax citrulli* AAC00-1]
fig|80869.182.peg.2568	T3S	penicillin-binding protein 1A [*Paracidovorax citrulli*]
fig|80869.182.peg.261	T3S	hypothetical protein APS58_0185 [*Paracidovorax citrulli*]
fig|80869.182.peg.2616	T3S	precorrin-2 C(20)-methyltransferase [*Paracidovorax citrulli*]
fig|80869.182.peg.2620	T3S	sirohydrochlorin chelatase [*Paracidovorax citrulli*]
fig|80869.182.peg.2623	T3S	peptidoglycan-binding domain-containing protein [*Paracidovorax citrulli*]
fig|80869.182.peg.2659	T3S	ABC transporter ATP-binding protein [*Paracidovorax citrulli*]
fig|80869.182.peg.2694	T3S	Holliday junction resolvase RuvX [*Paracidovorax citrulli*]
fig|80869.182.peg.2703	T3S	hydroxymethylpyrimidine/phosphomethylpyrimidine kinase [*Paracidovorax citrulli*]
fig|80869.182.peg.2737	T3S	ribonucleoside-diphosphate reductase subunit alpha [*Paracidovorax citrulli*]
fig|80869.182.peg.2749	T3S	3-deoxy-7-phosphoheptulonate synthase [*Paracidovorax citrulli*]
fig|80869.182.peg.2795	T3S	CobW family GTP-binding protein [*Paracidovorax citrulli*]
fig|80869.182.peg.2806	T3S	chromate efflux transporter [*Paracidovorax citrulli*]
fig|80869.182.peg.2813	T3S	M48 family metallopeptidase [*Paracidovorax citrulli*]
fig|80869.182.peg.2830	T3S	DNA-3-methyladenine glycosylase I [*Paracidovorax citrulli*]
fig|80869.182.peg.2831	T3S	putative zinc protease protein [*Paracidovorax citrulli* AAC00-1]
fig|80869.182.peg.2875	T3S	hypothetical protein [*Paracidovorax citrulli*]
fig|80869.182.peg.2888	T3S	hypothetical protein [*Paracidovorax citrulli*]
fig|80869.182.peg.2903	T3S	3-methyl-2-oxobutanoate hydroxymethyltransferase [*Paracidovorax citrulli*]
fig|80869.182.peg.2924	T3S	signal recognition particle-docking protein FtsY [*Paracidovorax citrulli*]
fig|80869.182.peg.2934	T3S	MATE family efflux transporter [*Paracidovorax citrulli*]
fig|80869.182.peg.3004	T3S	hypothetical protein C8E08_1237 [*Paracidovorax citrulli*]
fig|80869.182.peg.3005	T3S	hypothetical protein [*Paracidovorax citrulli*]

Prot	Pred	Description
fig|80869.182.peg.3032	T3S	luciferase family protein [*Paracidovorax citrulli* AAC00-1]
fig|80869.182.peg.3045	T3S	hypothetical protein [*Paracidovorax citrulli*]
fig|80869.182.peg.305	T3S	MarR family winged helix-turn-helix transcriptional regulator [*Paracidovorax citrulli*]
fig|80869.182.peg.3058	T3S	ABC transporter permease [*Paracidovorax citrulli*]
fig|80869.182.peg.3059	T3S	ABC transporter permease [*Paracidovorax citrulli*]
fig|80869.182.peg.3068	T3S	hypothetical protein [*Paracidovorax citrulli*]
fig|80869.182.peg.3100	T3S	succinate-semialdehyde dehydrogenase/glutarate-semialdehyde dehydrogenase [*Paracidovorax citrulli*]
fig|80869.182.peg.3156	T3S	(2Fe-2S)-binding protein [*Paracidovorax citrulli*]
fig|80869.182.peg.3157	T3S	molybdenum cofactor cytidylyltransferase [*Paracidovorax citrulli*]
fig|80869.182.peg.3163	T3S	hypothetical protein [*Paracidovorax citrulli*]
fig|80869.182.peg.3176	T3S	alpha/beta hydrolase [*Paracidovorax citrulli*]
fig|80869.182.peg.3205	T3S	septal ring lytic transglycosylase RlpA family protein [*Paracidovorax citrulli*]
fig|80869.182.peg.3218	T3S	hypothetical protein [*Paracidovorax citrulli*]
fig|80869.182.peg.3225	T3S	MULTISPECIES: TRAP transporter small permease [Paracidovorax]
fig|80869.182.peg.3277	T3S	rhodanese-like domain-containing protein [*Paracidovorax citrulli*]
fig|80869.182.peg.328	T3S	PepSY-associated TM helix domain-containing protein [*Paracidovorax citrulli*]
fig|80869.182.peg.3293	T3S	GNAT family N-acetyltransferase [*Paracidovorax citrulli*]
fig|80869.182.peg.3306	T3S	ABC transporter permease [*Paracidovorax citrulli*]
fig|80869.182.peg.3311	T3S	Metallo-beta-lactamase superfamily protein [*Paracidovorax citrulli*]
fig|80869.182.peg.3323	T3S	glycoside hydrolase family 15 protein [*Paracidovorax citrulli*]
fig|80869.182.peg.333	T3S	hypothetical protein [*Paracidovorax citrulli*]
fig|80869.182.peg.3343	T3S	hypothetical protein [*Paracidovorax citrulli*]
fig|80869.182.peg.3371	T3S	ABC transporter permease [*Paracidovorax citrulli*]
fig|80869.182.peg.339	T3S	DMT family transporter [*Paracidovorax citrulli*]
fig|80869.182.peg.3439	T3S	GNAT family N-acetyltransferase [*Paracidovorax citrulli*]
fig|80869.182.peg.3445	T3S	4'-phosphopantetheinyl transferase [*Paracidovorax citrulli* AAC00-1]
fig|80869.182.peg.3446	T3S	hypothetical protein [*Paracidovorax citrulli*]
fig|80869.182.peg.3459	T3S	Bifunctional protein PutA [*Paracidovorax citrulli*]
fig|80869.182.peg.3461	T3S	hypothetical protein [*Paracidovorax citrulli*]
fig|80869.182.peg.35	T3S	alpha-1,4-glucan--maltose-1-phosphate maltosyltransferase [*Paracidovorax citrulli*]
fig|80869.182.peg.3534	T3S	ABC transporter permease [*Paracidovorax citrulli*]
fig|80869.182.peg.356	T3S	flagellar hook assembly protein FlgD [*Paracidovorax citrulli*]
fig|80869.182.peg.361	T3S	hypothetical protein [*Paracidovorax citrulli*]
fig|80869.182.peg.2562	T4S	Ig domain protein, group 1 domain protein [*Paracidovorax citrulli* AAC00-1]
fig|80869.182.peg.3071	T4S	hypothetical protein [*Paracidovorax citrulli*]
fig|80869.182.peg.599	T4S	major facilitator superfamily MFS_1 [*Paracidovorax citrulli* AAC00-1]
fig|80869.182.peg.214	T4S	acyl-CoA thioesterase [*Paracidovorax citrulli*]
fig|80869.182.peg.756	T4S	STY0301 family protein [*Paracidovorax citrulli*]
fig|80869.182.peg.3841	T4S	ABC transporter ATP-binding protein [*Paracidovorax citrulli*]
fig|80869.182.peg.865	T4S	Ku protein [*Paracidovorax citrulli*]
fig|80869.182.peg.3974	T4S	hypothetical protein [*Paracidovorax citrulli*]
fig|80869.182.peg.2917	T4S	bacterioferritin [*Paracidovorax citrulli*]
fig|80869.182.peg.1328	T4S	terminase small subunit [*Paracidovorax citrulli*]
fig|80869.182.peg.4354	T4S	molybdenum cofactor biosynthesis protein MoaE [*Paracidovorax citrulli*]

Prot	Pred	Description
fig|80869.182.peg.3629	T4S	hypothetical protein [*Paracidovorax citrulli*]
fig|80869.182.peg.3988	T4S	isoaspartyl peptidase/L-asparaginase family protein [*Paracidovorax citrulli*]
fig|80869.182.peg.253	T4S	RNA recognition motif domain-containing protein [*Paracidovorax citrulli*]
fig|80869.182.peg.3726	T4S	hypothetical protein Aave_3072 [*Paracidovorax citrulli* AAC00-1]
fig|80869.182.peg.2427	T4S	DUF1795 domain-containing protein [*Paracidovorax citrulli*]
fig|80869.182.peg.3905	T4S	hypothetical protein [*Paracidovorax citrulli*]
fig|80869.182.peg.2218	T4S	MULTISPECIES: amino-acid N-acetyltransferase [Paracidovorax]
fig|80869.182.peg.91	T4S	DUF924 family protein [*Paracidovorax citrulli*]
fig|80869.182.peg.1189	T4S	hypothetical protein [*Paracidovorax citrulli*]
fig|80869.182.peg.4678	T4S	XopE/AvrPphe family type III secretion system effector [*Paracidovorax citrulli*]
fig|80869.182.peg.660	T4S	50S ribosomal protein L11 [*Paracidovorax citrulli*]
fig|80869.182.peg.573	T4S	conserved hypothetical protein [*Paracidovorax citrulli* AAC00-1]
fig|80869.182.peg.4534	T4S	SMC-Scp complex subunit ScpB [*Paracidovorax citrulli*]
fig|80869.182.peg.1037	T4S	PP2C family protein-serine/threonine phosphatase [*Paracidovorax citrulli*]
fig|80869.182.peg.2599	T4S	hypothetical protein [*Paracidovorax citrulli*]
fig|80869.182.peg.680	T4S	cryptochrome/photolyase family protein [*Paracidovorax citrulli*]
fig|80869.182.peg.4432	T4S	phosphopyruvate hydratase [*Paracidovorax citrulli*]
fig|80869.182.peg.2971	T4S	glutathione-regulated potassium-efflux system protein KefC [*Paracidovorax citrulli*]
fig|80869.182.peg.1094	T4S	hypothetical protein [*Paracidovorax citrulli*]
fig|80869.182.peg.3524	T4S	hypothetical protein [*Paracidovorax citrulli*]
fig|80869.182.peg.1450	T4S	AAA family ATPase [*Paracidovorax citrulli*]
fig|80869.182.peg.4024	T4S	DUF3025 domain-containing protein [*Paracidovorax citrulli*]
fig|80869.182.peg.1036	T4S	serine/threonine protein kinase [*Paracidovorax citrulli*]
fig|80869.182.peg.279	T4S	hypothetical protein [*Paracidovorax citrulli*]
fig|80869.182.peg.3113	T4S	inositol monophosphatase family protein [*Paracidovorax citrulli*]
fig|80869.182.peg.3358	T4S	N-acetylmuramoyl-L-alanine amidase [*Paracidovorax citrulli*]
fig|80869.182.peg.2105	T4S	AraC family transcriptional regulator [*Paracidovorax citrulli*]
fig|80869.182.peg.3463	T4S	hypothetical protein [*Paracidovorax citrulli*]
fig|80869.182.peg.2474	T4S	Fic family protein [*Paracidovorax citrulli*]
fig|80869.182.peg.298	T4S	4-hydroxy-3-methylbut-2-enyl diphosphate reductase [*Paracidovorax citrulli*]
fig|80869.182.peg.189	T4S	SsrA-binding protein SmpB [*Paracidovorax citrulli*]
fig|80869.182.peg.1979	T4S	F0F1 ATP synthase subunit gamma [*Paracidovorax citrulli*]
fig|80869.182.peg.4560	T4S	hypothetical protein [*Paracidovorax citrulli*]
fig|80869.182.peg.2319	T4S	transglycosylase SLT domain-containing protein [*Paracidovorax citrulli*]
fig|80869.182.peg.2086	T4S	type III secretion system outer membrane ring subunit SctC [*Paracidovorax citrulli*]
fig|80869.182.peg.1346	T4S	hypothetical protein [*Paracidovorax citrulli*]
fig|80869.182.peg.3034	T4S	5'-nucleotidase [*Paracidovorax citrulli*]
fig|80869.182.peg.2141	T4S	30S ribosomal protein S4 [*Paracidovorax citrulli*]
fig|80869.182.peg.2228	T4S	adenylate kinase [*Paracidovorax citrulli*]
fig|80869.182.peg.3543	T4S	hypothetical protein [*Paracidovorax citrulli*]
fig|80869.182.peg.480	T4S	hypothetical protein [*Paracidovorax citrulli*]
fig|80869.182.peg.1068	T4S	type IV pilin protein [*Paracidovorax citrulli*]
fig|80869.182.peg.4043	T4S	hypothetical protein [*Paracidovorax citrulli*]
fig|80869.182.peg.1119	T4S	urea ABC transporter substrate-binding protein [Paracidovorax anthurii]

Prot	Pred	Description
fig|80869.182.peg.2918	T4S	BON domain-containing protein [*Paracidovorax citrulli*]
fig|80869.182.peg.2204	T4S	MetQ/NlpA family ABC transporter substrate-binding protein [*Paracidovorax citrulli*]
fig|80869.182.peg.306	T4S	Bug family tripartite tricarboxylate transporter substrate binding protein [*Paracidovorax citrulli*]
fig|80869.182.peg.1443	T4S	Bug family tripartite tricarboxylate transporter substrate binding protein [*Paracidovorax citrulli*]
fig|80869.182.peg.2210	T4S	sulfate ABC transporter substrate-binding protein [*Paracidovorax citrulli*]
fig|80869.182.peg.3409	T4S	hypothetical protein [*Paracidovorax citrulli*]
fig|80869.182.peg.90	T4S	OmpA family protein [*Paracidovorax citrulli*]
fig|80869.182.peg.1364	T4S	glycoside hydrolase family protein [*Paracidovorax citrulli*]
fig|80869.182.peg.1904	T4S	HTH-type transcriptional regulator GltC [*Paracidovorax citrulli*]
fig|80869.182.peg.1653	T4S	sigma-54-dependent transcriptional regulator [*Paracidovorax citrulli*]
fig|80869.182.peg.3035	T4S	EF-hand domain-containing protein [*Paracidovorax citrulli*]

*P_citrulli*_NWBS196

Prot	Pred	Description
fig|80869.181.peg.1961	T3S	Ig domain protein, group 1 domain protein [*Paracidovorax citrulli* AAC00-1]
fig|80869.181.peg.3767	T3S	major facilitator superfamily MFS_1 [*Paracidovorax citrulli* AAC00-1]
fig|80869.181.peg.3880	T3S	hypothetical protein [*Paracidovorax citrulli*]
fig|80869.181.peg.3260	T3S	acyl-CoA thioesterase [*Paracidovorax citrulli*]
fig|80869.181.peg.3342	T3S	STY0301 family protein [*Paracidovorax citrulli*]
fig|80869.181.peg.4020	T3S	ABC transporter ATP-binding protein [*Paracidovorax citrulli*]
fig|80869.181.peg.3447	T3S	Ku protein [*Paracidovorax citrulli*]
fig|80869.181.peg.3460	T3S	hypothetical protein [*Paracidovorax citrulli*]
fig|80869.181.peg.112	T3S	bacterioferritin [*Paracidovorax citrulli*]
fig|80869.181.peg.4650	T3S	terminase small subunit [*Paracidovorax citrulli*]
fig|80869.181.peg.1010	T3S	flagellar basal body protein [*Paracidovorax citrulli*]
fig|80869.181.peg.1011	T3S	LysR family transcriptional regulator [*Paracidovorax citrulli*]
fig|80869.181.peg.1036	T3S	Ribosomal RNA small subunit methyltransferase E [*Paracidovorax citrulli*]
fig|80869.181.peg.1055	T3S	KdsC family phosphatase [*Paracidovorax citrulli*]
fig|80869.181.peg.1059	T3S	PepSY domain-containing protein [*Paracidovorax citrulli*]
fig|80869.181.peg.1070	T3S	DNA topoisomerase (ATP-hydrolyzing) subunit B [*Paracidovorax citrulli*]
fig|80869.181.peg.1092	T3S	2-isopropylmalate synthase [*Paracidovorax citrulli*]
fig|80869.181.peg.1121	T3S	ATP-binding cassette domain-containing protein [*Paracidovorax citrulli*]
fig|80869.181.peg.1144	T3S	sirohydrochlorin chelatase [*Paracidovorax citrulli*]
fig|80869.181.peg.1148	T3S	precorrin-2 C(20)-methyltransferase [*Paracidovorax citrulli*]
fig|80869.181.peg.1180	T3S	hotdog fold thioesterase [*Paracidovorax citrulli*]
fig|80869.181.peg.1184	T3S	hypothetical protein [*Paracidovorax citrulli*]
fig|80869.181.peg.119	T3S	signal recognition particle-docking protein FtsY [*Paracidovorax citrulli*]
fig|80869.181.peg.1192	T3S	MBL fold metallo-hydrolase [*Paracidovorax citrulli*]
fig|80869.181.peg.1196	T3S	5-demethoxyubiquinol-8 5-hydroxylase UbiM [*Paracidovorax citrulli*]
fig|80869.181.peg.1267	T3S	Cupin 2, conserved barrel domain protein [*Paracidovorax citrulli* AAC00-1]
fig|80869.181.peg.129	T3S	MATE family efflux transporter [*Paracidovorax citrulli*]
fig|80869.181.peg.1319	T3S	GNAT family N-acetyltransferase [Paracidovorax avenae]
fig|80869.181.peg.1340	T3S	ProQ/FINO family protein [*Paracidovorax citrulli*]
fig|80869.181.peg.1371	T3S	AmpG family muropeptide MFS transporter [*Paracidovorax citrulli*]

Prot	Pred	Description
fig|80869.181.peg.1374	T3S	DME family drug/metabolite transporter [*Paracidovorax citrulli*]
fig|80869.181.peg.1376	T3S	SLC13 family permease [*Paracidovorax citrulli*]
fig|80869.181.peg.1395	T3S	amino acid ABC transporter ATP-binding protein [*Paracidovorax citrulli*]
fig|80869.181.peg.1403	T3S	MULTISPECIES: ABC transporter ATP-binding protein [Paracidovorax]
fig|80869.181.peg.1418	T3S	DHA2 family efflux MFS transporter permease subunit [*Paracidovorax citrulli*]
fig|80869.181.peg.1419	T3S	efflux RND transporter periplasmic adaptor subunit [*Paracidovorax citrulli*]
fig|80869.181.peg.1457	T3S	potassium-transporting ATPase subunit KdpB [*Paracidovorax citrulli*]
fig|80869.181.peg.1481	T3S	NEL-type E3 ubiquitin ligase domain-containing protein [*Paracidovorax citrulli*]
fig|80869.181.peg.1490	T3S	N-acetylneuraminate epimerase [*Paracidovorax citrulli*]
fig|80869.181.peg.1522	T3S	TspO/MBR family protein [*Paracidovorax citrulli*]
fig|80869.181.peg.1527	T3S	hypothetical protein [*Paracidovorax citrulli*]
fig|80869.181.peg.1534	T3S	DUF4139 domain-containing protein [*Paracidovorax citrulli*]
fig|80869.181.peg.1561	T3S	pectate lyase [*Paracidovorax citrulli*]
fig|80869.181.peg.1564	T3S	hypothetical protein [*Paracidovorax citrulli*]
fig|80869.181.peg.1565	T3S	hypothetical protein [*Paracidovorax citrulli*]
fig|80869.181.peg.1577	T3S	hypothetical protein [*Paracidovorax citrulli*]
fig|80869.181.peg.1593	T3S	uracil-DNA glycosylase [*Paracidovorax citrulli*]
fig|80869.181.peg.1608	T3S	hypothetical protein APS58_2360 [*Paracidovorax citrulli*]
fig|80869.181.peg.1610	T3S	pyrroline-5-carboxylate reductase [*Paracidovorax citrulli*]
fig|80869.181.peg.1654	T3S	FAD-linked oxidase C-terminal domain-containing protein [*Paracidovorax citrulli*]
fig|80869.181.peg.1667	T3S	transcriptional regulator, AraC family [*Paracidovorax citrulli* AAC00-1]
fig|80869.181.peg.1669	T3S	NarK family nitrate/nitrite MFS transporter [*Paracidovorax citrulli*]
fig|80869.181.peg.1696	T3S	DUF2169 domain-containing protein [*Paracidovorax citrulli*]
fig|80869.181.peg.1727	T3S	putative zinc protease protein [*Paracidovorax citrulli* AAC00-1]
fig|80869.181.peg.1728	T3S	DNA-3-methyladenine glycosylase I [*Paracidovorax citrulli*]
fig|80869.181.peg.1744	T3S	M48 family metallopeptidase [*Paracidovorax citrulli*]
fig|80869.181.peg.1751	T3S	chromate efflux transporter [*Paracidovorax citrulli*]
fig|80869.181.peg.1807	T3S	3-deoxy-7-phosphoheptulonate synthase [*Paracidovorax citrulli*]
fig|80869.181.peg.1819	T3S	ribonucleoside-diphosphate reductase subunit alpha [*Paracidovorax citrulli*]
fig|80869.181.peg.1853	T3S	hydroxymethylpyrimidine/phosphomethylpyrimidine kinase [*Paracidovorax citrulli*]
fig|80869.181.peg.1862	T3S	Holliday junction resolvase RuvX [*Paracidovorax citrulli*]
fig|80869.181.peg.1895	T3S	ABC transporter ATP-binding protein [*Paracidovorax citrulli*]
fig|80869.181.peg.1931	T3S	peptidoglycan-binding domain-containing protein [*Paracidovorax citrulli*]
fig|80869.181.peg.1955	T3S	penicillin-binding protein 1A [*Paracidovorax citrulli*]
fig|80869.181.peg.1965	T3S	protein of unknown function DUF1415 [*Paracidovorax citrulli* AAC00-1]
fig|80869.181.peg.198	T3S	type III secretion system chaperone [*Paracidovorax citrulli*]
fig|80869.181.peg.199	T3S	hypothetical protein [*Paracidovorax citrulli*]
fig|80869.181.peg.2002	T3S	PelD GGDEF domain-containing protein [*Paracidovorax citrulli*]
fig|80869.181.peg.2044	T3S	DMT family transporter [*Paracidovorax citrulli*]
fig|80869.181.peg.2063	T3S	GntR family transcriptional regulator [*Paracidovorax citrulli*]
fig|80869.181.peg.2065	T3S	NCS1 family nucleobase:cation symporter-1 [*Paracidovorax citrulli*]
fig|80869.181.peg.2078	T3S	glycerate kinase [*Paracidovorax citrulli*]
fig|80869.181.peg.2098	T3S	ABC transporter ATP-binding protein [*Paracidovorax citrulli*]
fig|80869.181.peg.2114	T3S	neutral zinc metallopeptidase [*Paracidovorax citrulli*]
fig|80869.181.peg.2125	T3S	tryptophan 2,3-dioxygenase [*Paracidovorax citrulli*]

Prot	Pred	Description
fig|80869.181.peg.2175	T3S	Phytochrome-like protein cph2 [*Paracidovorax citrulli*]
fig|80869.181.peg.2176	T3S	lysine--tRNA ligase [*Paracidovorax citrulli*]
fig|80869.181.peg.2183	T3S	LON peptidase substrate-binding domain-containing protein [*Paracidovorax citrulli*]
fig|80869.181.peg.2188	T3S	phosphoribosylanthranilate isomerase [*Paracidovorax citrulli* AAC00-1]
fig|80869.181.peg.2201	T3S	nucleotide exchange factor GrpE [*Paracidovorax citrulli*]
fig|80869.181.peg.2216	T3S	molybdopterin converting factor subunit 1 [*Paracidovorax citrulli*]
fig|80869.181.peg.2229	T3S	hypothetical protein [*Paracidovorax citrulli*]
fig|80869.181.peg.2249	T3S	MULTISPECIES: NADH-quinone oxidoreductase subunit NuoI [Comamonadaceae]
fig|80869.181.peg.2257	T3S	ABC transporter transmembrane domain-containing protein [*Paracidovorax citrulli*]
fig|80869.181.peg.226	T3S	luciferase family protein [*Paracidovorax citrulli* AAC00-1]
fig|80869.181.peg.2275	T3S	winged helix-turn-helix transcriptional regulator [*Paracidovorax citrulli*]
fig|80869.181.peg.2284	T3S	N-acetylmuramoyl-L-alanine amidase [*Paracidovorax citrulli*]
fig|80869.181.peg.2295	T3S	EAL and HDOD domain-containing protein [*Paracidovorax citrulli*]
fig|80869.181.peg.2299	T3S	hypothetical protein [*Paracidovorax citrulli*]
fig|80869.181.peg.2325	T3S	pyridoxal kinase PdxY [*Paracidovorax citrulli*]
fig|80869.181.peg.2331	T3S	DNA internalization-related competence protein ComEC/Rec2 [*Paracidovorax citrulli*]
fig|80869.181.peg.2340	T3S	uncharacterized protein DUF2132 [*Paracidovorax citrulli*]
fig|80869.181.peg.2373	T3S	CaiB/BaiF CoA transferase family protein [*Paracidovorax citrulli*]
fig|80869.181.peg.2384	T3S	ketopantoate reductase [*Paracidovorax citrulli*]
fig|80869.181.peg.239	T3S	hypothetical protein [*Paracidovorax citrulli*]
fig|80869.181.peg.2399	T3S	protein-L-isoaspartate(D-aspartate) O-methyltransferase [*Paracidovorax citrulli*]
fig|80869.181.peg.24	T3S	hypothetical protein [*Paracidovorax citrulli*]
fig|80869.181.peg.2414	T3S	GTPase HflX [*Paracidovorax citrulli*]
fig|80869.181.peg.2439	T3S	DUF2069 domain-containing protein [*Paracidovorax citrulli*]
fig|80869.181.peg.2483	T3S	glycine betaine/L-proline ABC transporter permease ProW [*Paracidovorax citrulli*]
fig|80869.181.peg.2501	T3S	malonate--CoA ligase [*Paracidovorax citrulli*]
fig|80869.181.peg.2506	T3S	heavy metal translocating P-type ATPase [*Paracidovorax citrulli*]
fig|80869.181.peg.252	T3S	ABC transporter permease [*Paracidovorax citrulli*]
fig|80869.181.peg.253	T3S	ABC transporter permease [*Paracidovorax citrulli*]
fig|80869.181.peg.2536	T3S	SDR family NAD(P)-dependent oxidoreductase [*Paracidovorax citrulli*]
fig|80869.181.peg.2582	T3S	type 1 glutamine amidotransferase domain-containing protein [*Paracidovorax citrulli*]
fig|80869.181.peg.2584	T3S	polyhydroxyalkanoate synthesis repressor PhaR [*Paracidovorax citrulli*]
fig|80869.181.peg.265	T3S	(2Fe-2S)-binding protein [*Paracidovorax citrulli*]
fig|80869.181.peg.266	T3S	molybdenum cofactor cytidylyltransferase [*Paracidovorax citrulli*]
fig|80869.181.peg.2698	T3S	GNAT family N-acetyltransferase [*Paracidovorax citrulli*]
fig|80869.181.peg.2704	T3S	4'-phosphopantetheinyl transferase [*Paracidovorax citrulli* AAC00-1]
fig|80869.181.peg.2705	T3S	hypothetical protein [*Paracidovorax citrulli*]
fig|80869.181.peg.272	T3S	hypothetical protein [*Paracidovorax citrulli*]
fig|80869.181.peg.273	T3S	NUDIX hydrolase [*Paracidovorax citrulli*]
fig|80869.181.peg.2772	T3S	glycerophosphodiester phosphodiesterase [*Paracidovorax citrulli*]
fig|80869.181.peg.2795	T3S	Cardiolipin synthase B [*Paracidovorax citrulli*]
fig|80869.181.peg.285	T3S	alpha/beta hydrolase [*Paracidovorax citrulli*]
fig|80869.181.peg.2864	T3S	SAM-dependent methyltransferase [*Paracidovorax citrulli*]
fig|80869.181.peg.2902	T3S	urease accessory protein UreG [*Paracidovorax citrulli*]

Prot	Pred	Description
fig|80869.181.peg.2903	T3S	urease accessory protein [*Paracidovorax citrulli*]
fig|80869.181.peg.2942	T3S	PilZ domain-containing protein [*Paracidovorax citrulli*]
fig|80869.181.peg.2984	T3S	hypothetical protein, partial [*Paracidovorax citrulli*]
fig|80869.181.peg.2986	T3S	isoleucine--tRNA ligase [*Paracidovorax citrulli*]
fig|80869.181.peg.3001	T3S	protein of unknown function DUF1653 [*Paracidovorax citrulli* AAC00-1]
fig|80869.181.peg.3005	T3S	hypothetical protein [*Paracidovorax citrulli*]
fig|80869.181.peg.3094	T3S	argininosuccinate lyase [*Paracidovorax citrulli*]
fig|80869.181.peg.3124	T3S	CDP-6-deoxy-delta-3,4-glucoseen reductase [*Paracidovorax citrulli*]
fig|80869.181.peg.3133	T3S	hypothetical protein [*Paracidovorax citrulli*]
fig|80869.181.peg.3147	T3S	ABC-F family ATP-binding cassette domain-containing protein [*Paracidovorax citrulli*]
fig|80869.181.peg.3149	T3S	uroporphyrinogen-III C-methyltransferase [*Paracidovorax citrulli*]
fig|80869.181.peg.3151	T3S	hydroxymethylbilane synthase [*Paracidovorax citrulli*]
fig|80869.181.peg.3152	T3S	phosphoenolpyruvate carboxylase [*Paracidovorax citrulli*]
fig|80869.181.peg.3153	T3S	YccS/YhfK family membrane protein [*Paracidovorax citrulli*]
fig|80869.181.peg.3155	T3S	AI-2E family transporter [*Paracidovorax citrulli*]
fig|80869.181.peg.316	T3S	septal ring lytic transglycosylase RlpA family protein [*Paracidovorax citrulli*]
fig|80869.181.peg.3161	T3S	SfnB family sulfur acquisition oxidoreductase [*Paracidovorax citrulli*]
fig|80869.181.peg.3162	T3S	SfnB family sulfur acquisition oxidoreductase [*Paracidovorax citrulli*]
fig|80869.181.peg.3167	T3S	exodeoxyribonuclease VII small subunit [*Paracidovorax citrulli*]
fig|80869.181.peg.3178	T3S	alpha-1,4-glucan--maltose-1-phosphate maltosyltransferase [*Paracidovorax citrulli*]
fig|80869.181.peg.3186	T3S	TOBE domain-containing protein [*Paracidovorax citrulli*]
fig|80869.181.peg.3197	T3S	efflux transporter outer membrane subunit [*Paracidovorax citrulli*]
fig|80869.181.peg.3240	T3S	succinate-semialdehyde dehydrogenase/glutarate-semialdehyde dehydrogenase [*Paracidovorax citrulli*]
fig|80869.181.peg.3262	T3S	uroporphyrinogen-III C-methyltransferase [*Paracidovorax citrulli*]
fig|80869.181.peg.3288	T3S	MFS transporter [*Paracidovorax citrulli*]
fig|80869.181.peg.329	T3S	hypothetical protein [*Paracidovorax citrulli*]
fig|80869.181.peg.3335	T3S	KGG domain-containing protein [*Paracidovorax citrulli*]
fig|80869.181.peg.336	T3S	MULTISPECIES: TRAP transporter small permease [Paracidovorax]
fig|80869.181.peg.3378	T3S	PAS domain S-box protein [*Paracidovorax citrulli*]
fig|80869.181.peg.3410	T3S	EAL domain-containing protein [*Paracidovorax citrulli*]
fig|80869.181.peg.3537	T3S	TonB-dependent receptor family protein [*Paracidovorax citrulli*]
fig|80869.181.peg.3548	T3S	D-serine/D-alanine/glycine transporter [*Paracidovorax citrulli*]
fig|80869.181.peg.3549	T3S	hypothetical protein [*Paracidovorax citrulli*]
fig|80869.181.peg.3562	T3S	Polyphosphate kinase [*Paracidovorax citrulli* AAC00-1]
fig|80869.181.peg.3567	T3S	phosphate ABC transporter permease PstC [*Paracidovorax citrulli*]
fig|80869.181.peg.3600	T3S	hypothetical protein [*Paracidovorax citrulli*]
fig|80869.181.peg.3606	T3S	gephyrin-like molybdotransferase Glp [*Paracidovorax citrulli*]
fig|80869.181.peg.3620	T3S	translesion DNA synthesis-associated protein ImuA [*Paracidovorax citrulli*]
fig|80869.181.peg.3624	T3S	pseudouridine synthase [*Paracidovorax citrulli*]
fig|80869.181.peg.3657	T3S	pyridoxamine 5'-phosphate oxidase [*Paracidovorax citrulli*]
fig|80869.181.peg.3668	T3S	transcriptional repressor [*Paracidovorax citrulli*]
fig|80869.181.peg.3670	T3S	TetR/AcrR family transcriptional regulator [*Paracidovorax citrulli*]
fig|80869.181.peg.1961	T4S	Ig domain protein, group 1 domain protein [*Paracidovorax citrulli* AAC00-1]
fig|80869.181.peg.3767	T4S	major facilitator superfamily MFS_1 [*Paracidovorax citrulli* AAC00-1]
fig|80869.181.peg.3880	T4S	hypothetical protein [*Paracidovorax citrulli*]

Prot	Pred	Description
fig|80869.181.peg.3260	T4S	acyl-CoA thioesterase [*Paracidovorax citrulli*]
fig|80869.181.peg.3342	T4S	STY0301 family protein [*Paracidovorax citrulli*]
fig|80869.181.peg.4020	T4S	ABC transporter ATP-binding protein [*Paracidovorax citrulli*]
fig|80869.181.peg.3447	T4S	Ku protein [*Paracidovorax citrulli*]
fig|80869.181.peg.3460	T4S	hypothetical protein [*Paracidovorax citrulli*]
fig|80869.181.peg.112	T4S	bacterioferritin [*Paracidovorax citrulli*]
fig|80869.181.peg.4650	T4S	terminase small subunit [*Paracidovorax citrulli*]
fig|80869.181.peg.2223	T4S	molybdenum cofactor biosynthesis protein MoaE [*Paracidovorax citrulli*]
fig|80869.181.peg.4229	T4S	hypothetical protein [*Paracidovorax citrulli*]
fig|80869.181.peg.2596	T4S	isoaspartyl peptidase/L-asparaginase family protein [*Paracidovorax citrulli*]
fig|80869.181.peg.3299	T4S	RNA recognition motif domain-containing protein [*Paracidovorax citrulli*]
fig|80869.181.peg.4132	T4S	hypothetical protein Aave_3072 [*Paracidovorax citrulli* AAC00-1]
fig|80869.181.peg.3696	T4S	DUF1795 domain-containing protein [*Paracidovorax citrulli*]
fig|80869.181.peg.3490	T4S	MULTISPECIES: amino-acid N-acetyltransferase [Paracidovorax]
fig|80869.181.peg.848	T4S	DUF924 family protein [*Paracidovorax citrulli*]
fig|80869.181.peg.1026	T4S	hypothetical protein [*Paracidovorax citrulli*]
fig|80869.181.peg.784	T4S	50S ribosomal protein L11 [*Paracidovorax citrulli*]
fig|80869.181.peg.3792	T4S	conserved hypothetical protein [*Paracidovorax citrulli* AAC00-1]
fig|80869.181.peg.2402	T4S	SMC-Scp complex subunit ScpB [*Paracidovorax citrulli*]
fig|80869.181.peg.2857	T4S	PP2C family protein-serine/threonine phosphatase [*Paracidovorax citrulli*]
fig|80869.181.peg.1165	T4S	hypothetical protein [*Paracidovorax citrulli*]
fig|80869.181.peg.40	T4S	hypothetical protein APS58_p00028 [*Paracidovorax citrulli*]
fig|80869.181.peg.764	T4S	cryptochrome/photolyase family protein [*Paracidovorax citrulli*]
fig|80869.181.peg.2301	T4S	phosphopyruvate hydratase [*Paracidovorax citrulli*]
fig|80869.181.peg.165	T4S	glutathione-regulated potassium-efflux system protein KefC [*Paracidovorax citrulli*]
fig|80869.181.peg.2913	T4S	hypothetical protein [*Paracidovorax citrulli*]
fig|80869.181.peg.4333	T4S	hypothetical protein [*Paracidovorax citrulli*]
fig|80869.181.peg.4527	T4S	AAA family ATPase [*Paracidovorax citrulli*]
fig|80869.181.peg.549	T4S	DUF3025 domain-containing protein [*Paracidovorax citrulli*]
fig|80869.181.peg.2856	T4S	serine/threonine protein kinase [*Paracidovorax citrulli*]
fig|80869.181.peg.2628	T4S	hypothetical protein [*Paracidovorax citrulli*]
fig|80869.181.peg.3227	T4S	inositol monophosphatase family protein [*Paracidovorax citrulli*]
fig|80869.181.peg.469	T4S	N-acetylmuramoyl-L-alanine amidase [*Paracidovorax citrulli*]
fig|80869.181.peg.1600	T4S	AraC family transcriptional regulator [*Paracidovorax citrulli*]
fig|80869.181.peg.4391	T4S	hypothetical protein [*Paracidovorax citrulli*]
fig|80869.181.peg.883	T4S	Fic family protein [*Paracidovorax citrulli*]
fig|80869.181.peg.2646	T4S	4-hydroxy-3-methylbut-2-enyl diphosphate reductase [*Paracidovorax citrulli*]
fig|80869.181.peg.4450	T4S	SsrA-binding protein SmpB [*Paracidovorax citrulli*]
fig|80869.181.peg.1473	T4S	F0F1 ATP synthase subunit gamma [*Paracidovorax citrulli*]
fig|80869.181.peg.2429	T4S	hypothetical protein [*Paracidovorax citrulli*]
fig|80869.181.peg.3590	T4S	transglycosylase SLT domain-containing protein [*Paracidovorax citrulli*]
fig|80869.181.peg.1580	T4S	type III secretion system outer membrane ring subunit SctC [*Paracidovorax citrulli*]
fig|80869.181.peg.4631	T4S	hypothetical protein [*Paracidovorax citrulli*]
fig|80869.181.peg.2546	T4S	XopE/AvrPphe family type III secretion system effector [*Paracidovorax citrulli*]
fig|80869.181.peg.228	T4S	5'-nucleotidase [*Paracidovorax citrulli*]

Prot	Pred	Description
fig|80869.181.peg.1635	T4S	30S ribosomal protein S4 [*Paracidovorax citrulli*]
fig|80869.181.peg.3499	T4S	adenylate kinase [*Paracidovorax citrulli*]
fig|80869.181.peg.4314	T4S	hypothetical protein [*Paracidovorax citrulli*]
fig|80869.181.peg.3075	T4S	hypothetical protein [*Paracidovorax citrulli*]
fig|80869.181.peg.2887	T4S	type IV pilin protein [*Paracidovorax citrulli*]
fig|80869.181.peg.568	T4S	hypothetical protein [*Paracidovorax citrulli*]
fig|80869.181.peg.113	T4S	BON domain-containing protein [*Paracidovorax citrulli*]
fig|80869.181.peg.3476	T4S	MetQ/NlpA family ABC transporter substrate-binding protein [*Paracidovorax citrulli*]
fig|80869.181.peg.709	T4S	Bug family tripartite tricarboxylate transporter substrate binding protein [*Paracidovorax citrulli*]
fig|80869.181.peg.4533	T4S	Bug family tripartite tricarboxylate transporter substrate binding protein [*Paracidovorax citrulli*]
fig|80869.181.peg.3482	T4S	sulfate ABC transporter substrate-binding protein [*Paracidovorax citrulli*]
fig|80869.181.peg.2669	T4S	hypothetical protein [*Paracidovorax citrulli*]
fig|80869.181.peg.847	T4S	OmpA family protein [*Paracidovorax citrulli*]
fig|80869.181.peg.4613	T4S	glycoside hydrolase family protein [*Paracidovorax citrulli*]
fig|80869.181.peg.1399	T4S	HTH-type transcriptional regulator GltC [*Paracidovorax citrulli*]
fig|80869.181.peg.3044	T4S	sigma-54-dependent transcriptional regulator [*Paracidovorax citrulli*]
fig|80869.181.peg.229	T4S	EF-hand domain-containing protein [*Paracidovorax citrulli*]

*P_citrulli_*T1

Prot	Pred	Description
fig|80869.183.peg.1149	T3S	hypothetical protein [*Paracidovorax citrulli*]
fig|80869.183.peg.2061	T3S	hypothetical protein [*Paracidovorax citrulli*]
fig|80869.183.peg.4572	T3S	hydroxymethylpyrimidine/phosphomethylpyrimidine kinase [*Paracidovorax citrulli*]
fig|80869.183.peg.5923	T3S	CerR family C-terminal domain-containing protein [*Paracidovorax citrulli*]
fig|80869.183.peg.6791	T3S	aldehyde oxidoreductase molybdenum-binding subunit PaoC [*Paracidovorax citrulli*]
fig|80869.183.peg.3471	T3S	methyl-accepting chemotaxis protein [*Paracidovorax citrulli*]
fig|80869.183.peg.2485	T3S	acyl-CoA thioesterase [*Paracidovorax citrulli*]
fig|80869.183.peg.764	T3S	BON domain-containing protein [*Paracidovorax citrulli*]
fig|80869.183.peg.6000	T3S	hypothetical protein [*Paracidovorax citrulli*]
fig|80869.183.peg.5087	T3S	type VI secretion system Vgr family protein [*Paracidovorax citrulli*]
fig|80869.183.peg.6553	T3S	hypothetical protein [*Paracidovorax citrulli*]
fig|80869.183.peg.2259	T3S	GAF domain-containing sensor histidine kinase [*Paracidovorax citrulli*]
fig|80869.183.peg.3210	T3S	DUF3577 domain-containing protein [*Paracidovorax citrulli*]
fig|80869.183.peg.1356	T3S	ABC transporter ATP-binding protein [*Paracidovorax citrulli*]
fig|80869.183.peg.6241	T3S	hypothetical protein [*Paracidovorax citrulli*]
fig|80869.183.peg.2314	T3S	transcription antitermination factor NusB [*Paracidovorax citrulli*]
fig|80869.183.peg.1141	T3S	phage Gp37/Gp68 family protein [*Paracidovorax citrulli*]
fig|80869.183.peg.3518	T3S	phosphatase-like protein [*Paracidovorax citrulli* AAC00-1]
fig|80869.183.peg.4095	T3S	4-hydroxy-tetrahydrodipicolinate synthase [*Paracidovorax citrulli*]
fig|80869.183.peg.6577	T3S	terminase small subunit [*Paracidovorax citrulli*]
fig|80869.183.peg.5126	T3S	type III secretion system outer membrane ring subunit SctC [*Paracidovorax citrulli*]
fig|80869.183.peg.1001	T3S	murein DD-endopeptidase MepM/ murein hydrolase activator NlpD [*Paracidovorax citrulli*]
fig|80869.183.peg.1030	T3S	hypothetical protein [*Paracidovorax citrulli*]

Prot	Pred	Description
fig|80869.183.peg.1043	T3S	TRAP transporter small permease [*Paracidovorax citrulli*]
fig|80869.183.peg.1113	T3S	helix-turn-helix domain-containing protein [*Paracidovorax citrulli*]
fig|80869.183.peg.1172	T3S	PLxRFG domain-containing protein [*Paracidovorax citrulli*]
fig|80869.183.peg.1192	T3S	lysophospholipid acyltransferase family protein [*Paracidovorax citrulli*]
fig|80869.183.peg.1213	T3S	NCS1 family nucleobase:cation symporter-1 [*Paracidovorax citrulli*]
fig|80869.183.peg.124	T3S	prepilin-type N-terminal cleavage/methylation domain-containing protein [*Paracidovorax citrulli*]
fig|80869.183.peg.1319	T3S	cellulase family glycosylhydrolase [*Paracidovorax citrulli*]
fig|80869.183.peg.1332	T3S	protein of unknown function UPF0061 [*Paracidovorax citrulli* AAC00-1]
fig|80869.183.peg.137	T3S	hypothetical protein [*Paracidovorax citrulli*]
fig|80869.183.peg.1435	T3S	BON domain-containing protein [*Paracidovorax citrulli*]
fig|80869.183.peg.1443	T3S	LysR family transcriptional regulator [*Paracidovorax citrulli*]
fig|80869.183.peg.145	T3S	urease accessory protein UreG [*Paracidovorax citrulli*]
fig|80869.183.peg.1482	T3S	putative avirulence protein AvrRxo1 [*Paracidovorax citrulli* AAC00-1]
fig|80869.183.peg.1517	T3S	hypothetical protein [*Paracidovorax citrulli*]
fig|80869.183.peg.1538	T3S	YbaN family protein [*Paracidovorax citrulli*]
fig|80869.183.peg.1591	T3S	electron transfer flavoprotein-ubiquinone oxidoreductase [*Paracidovorax citrulli*]
fig|80869.183.peg.1607	T3S	short-chain dehydrogenase/reductase SDR [*Paracidovorax citrulli* AAC00-1]
fig|80869.183.peg.1628	T3S	CysB family HTH-type transcriptional regulator [*Paracidovorax citrulli*]
fig|80869.183.peg.1735	T3S	ABC transporter permease [Paracidovorax avenae]
fig|80869.183.peg.1800	T3S	hypothetical protein [*Paracidovorax citrulli*]
fig|80869.183.peg.1831	T3S	transport system permease protein [*Paracidovorax citrulli* AAC00-1]
fig|80869.183.peg.1853	T3S	extracellular solute-binding protein, partial [*Paracidovorax citrulli*]
fig|80869.183.peg.1882	T3S	helix-turn-helix transcriptional regulator [*Paracidovorax citrulli*]
fig|80869.183.peg.1901	T3S	hypothetical protein [*Paracidovorax citrulli*]
fig|80869.183.peg.1913	T3S	c-type cytochrome [*Paracidovorax citrulli*]
fig|80869.183.peg.1938	T3S	MAG: hypothetical protein BWX79_02790 [Alphaproteobacteria bacterium ADurb.Bin100]
fig|80869.183.peg.1958	T3S	DUF72 domain-containing protein [Paracidovorax cattleyae]
fig|80869.183.peg.1961	T3S	DUF1624 domain-containing protein [*Paracidovorax citrulli*]
fig|80869.183.peg.1962	T3S	glutamate--tRNA ligase [*Paracidovorax citrulli*]
fig|80869.183.peg.2000	T3S	UDP-3-O-(3-hydroxymyristoyl)glucosamine N-acyltransferase [*Paracidovorax citrulli*]
fig|80869.183.peg.2035	T3S	P1 family peptidase [*Paracidovorax citrulli*]
fig|80869.183.peg.2043	T3S	ATP synthase F1 subunit epsilon [*Paracidovorax citrulli*]
fig|80869.183.peg.2058	T3S	structural protein P5 [*Paracidovorax citrulli*]
fig|80869.183.peg.2152	T3S	hemolysin family protein [*Paracidovorax citrulli*]
fig|80869.183.peg.2154	T3S	threonine/serine dehydratase [*Paracidovorax citrulli*]
fig|80869.183.peg.2184	T3S	Uncharacterised protein [Mycobacterium tuberculosis]
fig|80869.183.peg.2252	T3S	MFS transporter [*Paracidovorax citrulli*]
fig|80869.183.peg.2273	T3S	SMP-30/gluconolactonase/LRE family protein [*Paracidovorax citrulli*]
fig|80869.183.peg.2325	T3S	cytochrome o ubiquinol oxidase subunit IV [*Paracidovorax citrulli*]
fig|80869.183.peg.2358	T3S	NAD-dependent succinate-semialdehyde dehydrogenase [*Paracidovorax citrulli*]
fig|80869.183.peg.2379	T3S	hypothetical protein [*Paracidovorax citrulli*]
fig|80869.183.peg.2380	T3S	secretion protein EspV [*Paracidovorax citrulli*]
fig|80869.183.peg.2415	T3S	hypothetical protein Aave_2148 [*Paracidovorax citrulli* AAC00-1]
fig|80869.183.peg.2418	T3S	nitrate ABC transporter permease [*Paracidovorax citrulli*]
fig|80869.183.peg.2439	T3S	DUF2169 domain-containing protein [*Paracidovorax citrulli*]

Prot	Pred	Description
fig|80869.183.peg.2457	T3S	carboxyl transferase domain-containing protein [*Paracidovorax citrulli*]
fig|80869.183.peg.2480	T3S	bacterioferritin-associated ferredoxin, partial [Paracidovorax avenae]
fig|80869.183.peg.2483	T3S	uroporphyrinogen-III C-methyltransferase [*Paracidovorax citrulli*]
fig|80869.183.peg.2516	T3S	YXWGXW repeat-containing protein [*Paracidovorax citrulli*]
fig|80869.183.peg.2576	T3S	ABC transporter permease [*Paracidovorax citrulli*]
fig|80869.183.peg.2605	T3S	hypothetical protein [*Paracidovorax citrulli*]
fig|80869.183.peg.2652	T3S	Metallo-beta-lactamase superfamily protein [*Paracidovorax citrulli*]
fig|80869.183.peg.2659	T3S	ABC transporter permease [*Paracidovorax citrulli*]
fig|80869.183.peg.2675	T3S	GNAT family N-acetyltransferase [*Paracidovorax citrulli*]
fig|80869.183.peg.2676	T3S	hypothetical protein [*Paracidovorax citrulli*]
fig|80869.183.peg.273	T3S	RsmB/NOP family class I SAM-dependent RNA methyltransferase [*Paracidovorax citrulli*]
fig|80869.183.peg.275	T3S	hypothetical protein [*Paracidovorax citrulli*]
fig|80869.183.peg.277	T3S	ribosomal protein [Pseudomonadota bacterium]
fig|80869.183.peg.2806	T3S	hypothetical protein [*Paracidovorax citrulli*]
fig|80869.183.peg.2834	T3S	ParA family protein [*Paracidovorax citrulli*]
fig|80869.183.peg.2888	T3S	acetyl-CoA C-acyltransferase [Paracidovorax avenae]
fig|80869.183.peg.3049	T3S	hypothetical protein [*Paracidovorax citrulli*]
fig|80869.183.peg.3111	T3S	hypothetical protein GY14_26970 [Delftia tsuruhatensis]
fig|80869.183.peg.3192	T3S	DEAD/DEAH box helicase [*Paracidovorax citrulli*]
fig|80869.183.peg.3205	T3S	hypothetical protein CQB05_01060 [*Paracidovorax citrulli*]
fig|80869.183.peg.3243	T3S	MAG: hypothetical protein BWX79_03004 [Alphaproteobacteria bacterium ADurb.Bin100]
fig|80869.183.peg.3257	T3S	gephyrin-like molybdotransferase Glp [*Paracidovorax citrulli*]
fig|80869.183.peg.3273	T3S	HI0933 family protein [*Paracidovorax citrulli* AAC00-1]
fig|80869.183.peg.3305	T3S	Polyphosphate kinase [*Paracidovorax citrulli* AAC00-1]
fig|80869.183.peg.3324	T3S	D-serine/D-alanine/glycine transporter [*Paracidovorax citrulli*]
fig|80869.183.peg.3347	T3S	tripartite tricarboxylate transporter substrate binding protein [*Paracidovorax citrulli*]
fig|80869.183.peg.3371	T3S	exodeoxyribonuclease VII large subunit [*Paracidovorax citrulli*]
fig|80869.183.peg.341	T3S	DUF1513 domain-containing protein [*Paracidovorax citrulli*]
fig|80869.183.peg.3482	T3S	EAL domain-containing protein [*Paracidovorax citrulli*]
fig|80869.183.peg.3485	T3S	polyhydroxyalkanoate depolymerase [*Paracidovorax citrulli*]
fig|80869.183.peg.3596	T3S	KGG domain-containing protein [*Paracidovorax citrulli*]
fig|80869.183.peg.36	T3S	binding-protein-dependent transport systems inner membrane component [*Paracidovorax citrulli* AAC00-1]
fig|80869.183.peg.3615	T3S	hypothetical protein [*Paracidovorax citrulli*]
fig|80869.183.peg.3664	T3S	alpha/beta hydrolase [*Paracidovorax citrulli*]
fig|80869.183.peg.3701	T3S	binding-protein-dependent transport systems inner membrane component [*Paracidovorax citrulli* AAC00-1]
fig|80869.183.peg.3704	T3S	ABC transporter ATP-binding protein [*Paracidovorax citrulli*]
fig|80869.183.peg.3752	T3S	glycerophosphodiester phosphodiesterase [*Paracidovorax citrulli*]
fig|80869.183.peg.377	T3S	argininosuccinate lyase [*Paracidovorax citrulli*]
fig|80869.183.peg.3807	T3S	MFS transporter [*Paracidovorax citrulli*]
fig|80869.183.peg.3839	T3S	hypothetical protein [*Paracidovorax citrulli*]
fig|80869.183.peg.3906	T3S	SDR family NAD(P)-dependent oxidoreductase [*Paracidovorax citrulli*]
fig|80869.183.peg.3915	T3S	CaiB/BaiF CoA transferase family protein [*Paracidovorax citrulli*]
fig|80869.183.peg.3967	T3S	pyridoxal kinase PdxY [*Paracidovorax citrulli*]
fig|80869.183.peg.4000	T3S	hypothetical protein [*Paracidovorax citrulli*]
fig|80869.183.peg.4031	T3S	hypothetical protein [Cypionkella sp.]

Prot	Pred	Description
fig|80869.183.peg.4060	T3S	NADH-quinone oxidoreductase subunit NuoI [*Paracidovorax citrulli*]
fig|80869.183.peg.4115	T3S	nucleotide exchange factor GrpE [*Paracidovorax citrulli*]
fig|80869.183.peg.4154	T3S	DUF3300 domain-containing protein [*Paracidovorax citrulli*]
fig|80869.183.peg.4155	T3S	DUF3300 domain-containing protein [*Paracidovorax citrulli*]
fig|80869.183.peg.4225	T3S	tryptophan 2,3-dioxygenase [*Paracidovorax citrulli*]
fig|80869.183.peg.4239	T3S	neutral zinc metallopeptidase [*Paracidovorax citrulli*]
fig|80869.183.peg.429	T3S	hypothetical protein [*Paracidovorax citrulli*]
fig|80869.183.peg.4306	T3S	NCS1 family nucleobase:cation symporter-1 [*Paracidovorax citrulli*]
fig|80869.183.peg.4307	T3S	GntR family transcriptional regulator [*Paracidovorax citrulli*]
fig|80869.183.peg.4329	T3S	DMT family transporter [*Paracidovorax citrulli*]
fig|80869.183.peg.4370	T3S	D-hexose-6-phosphate mutarotase [*Paracidovorax citrulli*]
fig|80869.183.peg.4379	T3S	PelD GGDEF domain-containing protein [*Paracidovorax citrulli*]
fig|80869.183.peg.4426	T3S	protein of unknown function DUF1415 [*Paracidovorax citrulli* AAC00-1]
fig|80869.183.peg.4431	T3S	Ig domain protein, group 1 domain protein [*Paracidovorax citrulli* AAC00-1]
fig|80869.183.peg.4437	T3S	penicillin-binding protein 1A [*Paracidovorax citrulli*]
fig|80869.183.peg.4467	T3S	peptidoglycan-binding domain-containing protein [*Paracidovorax citrulli*]
fig|80869.183.peg.4472	T3S	NAD(P)-dependent oxidoreductase [*Paracidovorax citrulli*]
fig|80869.183.peg.4521	T3S	dipeptide ABC transporter ATP-binding protein [*Paracidovorax citrulli*]
fig|80869.183.peg.455	T3S	phosphoenolpyruvate carboxylase [*Paracidovorax citrulli*]
fig|80869.183.peg.457	T3S	YccS/YhfK family membrane protein [*Paracidovorax citrulli*]
fig|80869.183.peg.459	T3S	AI-2E family transporter [*Paracidovorax citrulli*]
fig|80869.183.peg.4595	T3S	dimethylsulfone monooxygenase SfnG [*Paracidovorax citrulli*]
fig|80869.183.peg.4608	T3S	two-component system sensor histidine kinase NtrB [Acidovorax sp. PRC11]
fig|80869.183.peg.4611	T3S	ribonucleoside-diphosphate reductase subunit alpha [*Paracidovorax citrulli*]
fig|80869.183.peg.4624	T3S	3-deoxy-7-phosphoheptulonate synthase [Paracidovorax avenae]
fig|80869.183.peg.4634	T3S	RNB domain-containing ribonuclease [*Paracidovorax citrulli*]
fig|80869.183.peg.4694	T3S	chromate efflux transporter [*Paracidovorax citrulli*]
fig|80869.183.peg.4702	T3S	M48 family metalloprotease [*Paracidovorax citrulli*]
fig|80869.183.peg.4718	T3S	DNA-3-methyladenine glycosylase I [*Paracidovorax citrulli*]
fig|80869.183.peg.4812	T3S	FMN-dependent NADH-azoreductase [*Paracidovorax citrulli*]
fig|80869.183.peg.4953	T3S	uracil-DNA glycosylase [*Paracidovorax citrulli*]
fig|80869.183.peg.1149	T4S	hypothetical protein [*Paracidovorax citrulli*]
fig|80869.183.peg.2061	T4S	hypothetical protein [*Paracidovorax citrulli*]
fig|80869.183.peg.4572	T4S	hydroxymethylpyrimidine/phosphomethylpyrimidine kinase [*Paracidovorax citrulli*]
fig|80869.183.peg.5923	T4S	CerR family C-terminal domain-containing protein [*Paracidovorax citrulli*]
fig|80869.183.peg.6791	T4S	aldehyde oxidoreductase molybdenum-binding subunit PaoC [*Paracidovorax citrulli*]
fig|80869.183.peg.3471	T4S	methyl-accepting chemotaxis protein [*Paracidovorax citrulli*]
fig|80869.183.peg.2485	T4S	acyl-CoA thioesterase [*Paracidovorax citrulli*]
fig|80869.183.peg.764	T4S	BON domain-containing protein [*Paracidovorax citrulli*]
fig|80869.183.peg.6000	T4S	hypothetical protein [*Paracidovorax citrulli*]
fig|80869.183.peg.5087	T4S	type VI secretion system Vgr family protein [*Paracidovorax citrulli*]
fig|80869.183.peg.6553	T4S	hypothetical protein [*Paracidovorax citrulli*]
fig|80869.183.peg.2259	T4S	GAF domain-containing sensor histidine kinase [*Paracidovorax citrulli*]
fig|80869.183.peg.3210	T4S	DUF3577 domain-containing protein [*Paracidovorax citrulli*]
fig|80869.183.peg.1356	T4S	ABC transporter ATP-binding protein [*Paracidovorax citrulli*]
fig|80869.183.peg.6241	T4S	hypothetical protein [*Paracidovorax citrulli*]

Prot	Pred	Description
fig|80869.183.peg.2314	T4S	transcription antitermination factor NusB [*Paracidovorax citrulli*]
fig|80869.183.peg.1141	T4S	phage Gp37/Gp68 family protein [*Paracidovorax citrulli*]
fig|80869.183.peg.3518	T4S	phosphatase-like protein [*Paracidovorax citrulli* AAC00-1]
fig|80869.183.peg.4095	T4S	4-hydroxy-tetrahydrodipicolinate synthase [*Paracidovorax citrulli*]
fig|80869.183.peg.6577	T4S	terminase small subunit [*Paracidovorax citrulli*]
fig|80869.183.peg.5126	T4S	type III secretion system outer membrane ring subunit SctC [*Paracidovorax citrulli*]
fig|80869.183.peg.6435	T4S	Gp49 family protein [*Paracidovorax citrulli*]
fig|80869.183.peg.1718	T4S	hypothetical protein [Xanthomonas sacchari]
fig|80869.183.peg.5230	T4S	galactose oxidase [*Paracidovorax citrulli*]
fig|80869.183.peg.894	T4S	nuclear transport factor 2 family protein [*Paracidovorax citrulli*]
fig|80869.183.peg.3878	T4S	Hsp70 family protein [*Paracidovorax citrulli*]
fig|80869.183.peg.880	T4S	hypothetical protein [Acidovorax sp. NO-1]
fig|80869.183.peg.1214	T4S	NAD-dependent dihydropyrimidine dehydrogenase subunit PreA [*Paracidovorax citrulli*]
fig|80869.183.peg.6334	T4S	Ulp1 family type III secretion system effector isopeptidase XopD [*Paracidovorax citrulli*]
fig|80869.183.peg.2422	T4S	RNA recognition motif domain-containing protein [*Paracidovorax citrulli*]
fig|80869.183.peg.4268	T4S	diguanylate cyclase [*Paracidovorax citrulli*]
fig|80869.183.peg.3164	T4S	type IV pilus twitching motility protein PilT [Paracidovorax oryzae]
fig|80869.183.peg.1878	T4S	class I SAM-dependent DNA methyltransferase [*Paracidovorax citrulli*]
fig|80869.183.peg.1088	T4S	ParA family protein [*Paracidovorax citrulli*]
fig|80869.183.peg.1730	T4S	diguanylate cyclase domain-containing protein [*Paracidovorax citrulli*]
fig|80869.183.peg.841	T4S	hypothetical protein LMG26696_05193 [Achromobacter pulmonis]
fig|80869.183.peg.3204	T4S	hypothetical protein [*Paracidovorax citrulli*]
fig|80869.183.peg.4430	T4S	Ig domain protein, group 1 domain protein [*Paracidovorax citrulli* AAC00-1]
fig|80869.183.peg.1497	T4S	hypothetical protein [*Paracidovorax citrulli*]
fig|80869.183.peg.1907	T4S	translation initiation factor IF-2 [Paracidovorax avenae]
fig|80869.183.peg.2991	T4S	DUF1795 domain-containing protein [*Paracidovorax citrulli*]
fig|80869.183.peg.3558	T4S	YgiQ family radical SAM protein [*Paracidovorax citrulli*]
fig|80869.183.peg.6290	T4S	flagellar motor switch protein FliM [Paracidovorax cattleyae]
fig|80869.183.peg.6775	T4S	glycine betaine/L-proline ABC transporter substrate-binding protein ProX [*Paracidovorax citrulli*]
fig|80869.183.peg.1968	T4S	SPOR domain-containing protein [*Paracidovorax citrulli*]
fig|80869.183.peg.3387	T4S	MULTISPECIES: amino-acid N-acetyltransferase [Paracidovorax]
fig|80869.183.peg.909	T4S	cyanophycin synthetase [*Paracidovorax citrulli*]
fig|80869.183.peg.6020	T4S	DUF924 family protein [*Paracidovorax citrulli*]
fig|80869.183.peg.3824	T4S	hypothetical protein [*Paracidovorax citrulli*]
fig|80869.183.peg.3445	T4S	methylmalonyl Co-A mutase-associated GTPase MeaB [*Paracidovorax citrulli*]
fig|80869.183.peg.5780	T4S	hypothetical protein [*Paracidovorax citrulli*]
fig|80869.183.peg.1266	T4S	mannose-1-phosphate guanylyltransferase/mannose-6-phosphate isomerase [*Paracidovorax citrulli*]
fig|80869.183.peg.6189	T4S	Bug family tripartite tricarboxylate transporter substrate binding protein [*Paracidovorax citrulli*]
fig|80869.183.peg.6102	T4S	50S ribosomal protein L11 [*Paracidovorax citrulli*]
fig|80869.183.peg.4248	T4S	penicillin acylase family protein [*Paracidovorax citrulli*]
fig|80869.183.peg.4044	T4S	lipocalin-like domain-containing protein [*Paracidovorax citrulli*]
fig|80869.183.peg.5414	T4S	type VI secretion system Vgr family protein, partial [*Paracidovorax citrulli*]
fig|80869.183.peg.1974	T4S	UDP-N-acetylmuramate dehydrogenase [*Paracidovorax citrulli*]
fig|80869.183.peg.590	T4S	class I SAM-dependent rRNA methyltransferase [*Paracidovorax citrulli*]
fig|80869.183.peg.6160	T4S	hypothetical protein [*Paracidovorax citrulli*]

Prot	Pred	Description
fig|80869.183.peg.2311	T4S	T6SS phospholipase effector Tle1-like catalytic domain-containing protein [*Paracidovorax citrulli*]
fig|80869.183.peg.582	T4S	glycoside hydrolase family 10 protein [*Paracidovorax citrulli*]
fig|80869.183.peg.3404	T4S	MetQ/NlpA family ABC transporter substrate-binding protein [*Paracidovorax citrulli*]
fig|80869.183.peg.6021	T4S	OmpA family protein [*Paracidovorax citrulli*]
fig|80869.183.peg.2376	T4S	hypothetical protein [*Paracidovorax citrulli*]
fig|80869.183.peg.5621	T4S	hypothetical protein [*Paracidovorax citrulli*]
fig|80869.183.peg.3998	T4S	phosphopyruvate hydratase [*Paracidovorax citrulli*]
fig|80869.183.peg.5618	T4S	hypothetical protein [*Paracidovorax citrulli*]
fig|80869.183.peg.2202	T4S	hypothetical protein [*Paracidovorax citrulli*]
fig|80869.183.peg.2841	T4S	hypothetical protein [*Paracidovorax citrulli*]
fig|80869.183.peg.1747	T4S	hypothetical protein [*Paracidovorax citrulli*]
fig|80869.183.peg.2144	T4S	PLxRFG domain-containing protein [*Paracidovorax citrulli*]
fig|80869.183.peg.5338	T4S	ABC transporter substrate-binding protein [*Paracidovorax citrulli*]
fig|80869.183.peg.2607	T4S	phage tail protein [*Paracidovorax citrulli*]
fig|80869.183.peg.79	T4S	serine/threonine protein kinase [*Paracidovorax citrulli*]
fig|80869.183.peg.558	T4S	inositol monophosphatase family protein [*Paracidovorax citrulli*]
fig|80869.183.peg.1499	T4S	3-hydroxyacyl-CoA dehydrogenase [*Paracidovorax citrulli*]
fig|80869.183.peg.964	T4S	zinc-dependent alcohol dehydrogenase [*Paracidovorax citrulli*]
fig|80869.183.peg.4942	T4S	AraC family transcriptional regulator [*Paracidovorax citrulli*]
fig|80869.183.peg.200	T4S	diacylglycerol kinase family enzyme [*Paracidovorax citrulli*]
fig|80869.183.peg.4605	T4S	signal recognition particle receptor subunit alpha, partial [Acidovorax sp.]
fig|80869.183.peg.2269	T4S	hypothetical protein C8E08_4485 [*Paracidovorax citrulli*]
fig|80869.183.peg.6128	T4S	cryptochrome/photolyase family protein [*Paracidovorax citrulli*]
fig|80869.183.peg.1241	T4S	methyltransferase domain-containing protein [*Paracidovorax citrulli*]
fig|80869.183.peg.5983	T4S	Fic family protein [*Paracidovorax citrulli*]
fig|80869.183.peg.4599	T4S	tRNA (N6-isopentenyl adenosine(37)-C2)-methylthiotransferase MiaB [*Paracidovorax citrulli*]
fig|80869.183.peg.1755	T4S	conserved hypothetical protein [*Paracidovorax citrulli* AAC00-1]
fig|80869.183.peg.5044	T4S	type IV conjugative transfer system coupling protein TraD [*Paracidovorax citrulli*]
fig|80869.183.peg.1896	T4S	SsrA-binding protein SmpB [*Paracidovorax citrulli*]
fig|80869.183.peg.4536	T4S	hypothetical protein R69749_03487 [Paraburkholderia domus]
fig|80869.183.peg.3264	T4S	transglycosylase SLT domain-containing protein [*Paracidovorax citrulli*]
fig|80869.183.peg.2760	T4S	hypothetical protein [*Paracidovorax citrulli*]
fig|80869.183.peg.904	T4S	5'-nucleotidase [*Paracidovorax citrulli*]
fig|80869.183.peg.1615	T4S	2OG-Fe dioxygenase family protein [*Paracidovorax citrulli*]
fig|80869.183.peg.4904	T4S	30S ribosomal protein S4 [*Paracidovorax citrulli*]
fig|80869.183.peg.5975	T4S	DNA topoisomerase III [Paracidovorax avenae]
fig|80869.183.peg.5739	T4S	DNA polymerase III subunit beta [Acidovorax temperans]
fig|80869.183.peg.3428	T4S	Ku protein [*Paracidovorax citrulli*]
fig|80869.183.peg.6364	T4S	hypothetical protein [*Paracidovorax citrulli*]
fig|80869.183.peg.2899	T4S	hypothetical protein [*Paracidovorax citrulli*]
fig|80869.183.peg.6568	T4S	DUF6682 family protein [*Paracidovorax citrulli*]
fig|80869.183.peg.1280	T4S	hypothetical protein [*Paracidovorax citrulli*]
fig|80869.183.peg.4565	T4S	Hpt domain-containing protein [*Paracidovorax citrulli*]
fig|80869.183.peg.1151	T4S	hypothetical protein [*Paracidovorax citrulli*]
fig|80869.183.peg.5334	T4S	LysR family transcriptional regulator [*Paracidovorax citrulli*]

Prot	Pred	Description
fig|80869.183.peg.171	T4S	acetyl/propionyl/methylcrotonyl-CoA carboxylase subunit alpha [*Paracidovorax citrulli*]
fig|80869.183.peg.1152	T4S	endolysin [*Paracidovorax citrulli*]
fig|80869.183.peg.123	T4S	type IV pilin protein [*Paracidovorax citrulli*]
fig|80869.183.peg.2133	T4S	endolysin [*Paracidovorax citrulli*]
fig|80869.183.peg.6526	T4S	endolysin [*Paracidovorax citrulli*]
fig|80869.183.peg.3048	T4S	hypothetical protein [*Paracidovorax citrulli*]
fig|80869.183.peg.905	T4S	EF-hand domain-containing protein [*Paracidovorax citrulli*]
fig|80869.183.peg.3338	T4S	Bug family tripartite tricarboxylate transporter substrate binding protein [*Paracidovorax citrulli*]
fig|80869.183.peg.287	T4S	methyl-accepting chemotaxis protein [*Paracidovorax citrulli*]

*P_citrulli*_tw6

Prot	Pred	Description
fig|80869.184.peg.3497	T3S	Ig domain protein, group 1 domain protein [*Paracidovorax citrulli* AAC00-1]
fig|80869.184.peg.4037	T3S	serine/threonine protein kinase [*Paracidovorax citrulli*]
fig|80869.184.peg.835	T3S	hypothetical protein [*Paracidovorax citrulli*]
fig|80869.184.peg.819	T3S	acyl-CoA thioesterase [*Paracidovorax citrulli*]
fig|80869.184.peg.137	T3S	ABC transporter ATP-binding protein [*Paracidovorax citrulli*]
fig|80869.184.peg.483	T3S	Ku protein [*Paracidovorax citrulli*]
fig|80869.184.peg.4491	T3S	bacterioferritin [*Paracidovorax citrulli*]
fig|80869.184.peg.2076	T3S	terminase small subunit [*Paracidovorax citrulli*]
fig|80869.184.peg.1081	T3S	replication endonuclease [*Paracidovorax citrulli*]
fig|80869.184.peg.1138	T3S	hypothetical protein [*Paracidovorax citrulli*]
fig|80869.184.peg.1142	T3S	protein of unknown function DUF1653 [*Paracidovorax citrulli* AAC00-1]
fig|80869.184.peg.1157	T3S	isoleucine--tRNA ligase [*Paracidovorax citrulli*]
fig|80869.184.peg.117	T3S	PepSY-associated TM helix domain-containing protein [*Paracidovorax citrulli*]
fig|80869.184.peg.1182	T3S	AraC family transcriptional regulator [*Paracidovorax citrulli*]
fig|80869.184.peg.1200	T3S	FmdB family transcriptional regulator [*Paracidovorax citrulli*]
fig|80869.184.peg.1216	T3S	ABC transporter ATP-binding protein [*Paracidovorax citrulli*]
fig|80869.184.peg.1292	T3S	ABC transporter permease [*Paracidovorax citrulli*]
fig|80869.184.peg.1322	T3S	hypothetical protein [*Paracidovorax citrulli*]
fig|80869.184.peg.1358	T3S	glycoside hydrolase 15-related protein [*Paracidovorax citrulli* AAC00-1]
fig|80869.184.peg.1369	T3S	Metallo-beta-lactamase superfamily protein [*Paracidovorax citrulli*]
fig|80869.184.peg.1374	T3S	ABC transporter permease [*Paracidovorax citrulli*]
fig|80869.184.peg.1404	T3S	rhodanese-like domain-containing protein [*Paracidovorax citrulli*]
fig|80869.184.peg.1457	T3S	TRAP transporter small permease [*Paracidovorax citrulli*]
fig|80869.184.peg.1465	T3S	hypothetical protein [*Paracidovorax citrulli*]
fig|80869.184.peg.1478	T3S	septal ring lytic transglycosylase RlpA family protein [*Paracidovorax citrulli*]
fig|80869.184.peg.1513	T3S	alpha/beta hydrolase [*Paracidovorax citrulli*]
fig|80869.184.peg.1525	T3S	hypothetical protein [*Paracidovorax citrulli*]
fig|80869.184.peg.1531	T3S	molybdenum cofactor cytidylyltransferase [*Paracidovorax citrulli*]
fig|80869.184.peg.1532	T3S	(2Fe-2S)-binding protein, partial [*Paracidovorax citrulli*]
fig|80869.184.peg.1539	T3S	hotdog fold thioesterase [*Paracidovorax citrulli*]
fig|80869.184.peg.1543	T3S	hypothetical protein [*Paracidovorax citrulli*]
fig|80869.184.peg.1551	T3S	MBL fold metallo-hydrolase [*Paracidovorax citrulli*]

Prot	Pred	Description
fig|80869.184.peg.1555	T3S	5-demethoxyubiquinol-8 5-hydroxylase UbiM [*Paracidovorax citrulli*]
fig|80869.184.peg.1614	T3S	aromatic ring-hydroxylating dioxygenase subunit alpha [*Paracidovorax citrulli*]
fig|80869.184.peg.1626	T3S	Cupin 2, conserved barrel domain protein [*Paracidovorax citrulli* AAC00-1]
fig|80869.184.peg.1679	T3S	GNAT family N-acetyltransferase [Paracidovorax avenae]
fig|80869.184.peg.1728	T3S	ProQ/FINO family protein [*Paracidovorax citrulli*]
fig|80869.184.peg.1759	T3S	AmpG family muropeptide MFS transporter [*Paracidovorax citrulli*]
fig|80869.184.peg.1762	T3S	DME family drug/metabolite transporter [*Paracidovorax citrulli*]
fig|80869.184.peg.1764	T3S	SLC13 family permease [*Paracidovorax citrulli*]
fig|80869.184.peg.1783	T3S	amino acid ABC transporter ATP-binding protein [*Paracidovorax citrulli*]
fig|80869.184.peg.1791	T3S	MULTISPECIES: ABC transporter ATP-binding protein [Paracidovorax]
fig|80869.184.peg.1807	T3S	DHA2 family efflux MFS transporter permease subunit [*Paracidovorax citrulli*]
fig|80869.184.peg.1808	T3S	efflux RND transporter periplasmic adaptor subunit [*Paracidovorax citrulli*]
fig|80869.184.peg.1846	T3S	potassium-transporting ATPase subunit KdpB [*Paracidovorax citrulli*]
fig|80869.184.peg.1858	T3S	16S rRNA (cytosine(967)-C(5))-methyltransferase RsmB [*Paracidovorax citrulli*]
fig|80869.184.peg.1860	T3S	sensor histidine kinase [*Paracidovorax citrulli*]
fig|80869.184.peg.1879	T3S	PhaM family polyhydroxyalkanoate granule multifunctional regulatory protein [*Paracidovorax citrulli*]
fig|80869.184.peg.1897	T3S	hypothetical protein [*Paracidovorax citrulli*]
fig|80869.184.peg.1903	T3S	DMT family transporter [*Paracidovorax citrulli*]
fig|80869.184.peg.1919	T3S	flagellar hook assembly protein FlgD [*Paracidovorax citrulli*]
fig|80869.184.peg.1924	T3S	hypothetical protein [*Paracidovorax citrulli*]
fig|80869.184.peg.1937	T3S	conserved hypothetical protein [*Paracidovorax citrulli* AAC00-1]
fig|80869.184.peg.194	T3S	BON domain-containing protein [*Paracidovorax citrulli*]
fig|80869.184.peg.1940	T3S	flagellin [*Paracidovorax citrulli*]
fig|80869.184.peg.1941	T3S	flagellin [*Paracidovorax citrulli*]
fig|80869.184.peg.195	T3S	SulP family inorganic anion transporter [*Paracidovorax citrulli*]
fig|80869.184.peg.1960	T3S	hypothetical protein Aave_1748 [*Paracidovorax citrulli* AAC00-1]
fig|80869.184.peg.204	T3S	tRNA (guanosine(46)-N7)-methyltransferase TrmB [*Paracidovorax citrulli*]
fig|80869.184.peg.2073	T3S	hypothetical protein [*Paracidovorax citrulli*]
fig|80869.184.peg.2164	T3S	pyocin knob domain-containing protein [*Paracidovorax citrulli*]
fig|80869.184.peg.2167	T3S	structural protein P5 [*Paracidovorax citrulli*]
fig|80869.184.peg.2178	T3S	ATP synthase F1 subunit epsilon [*Paracidovorax citrulli*]
fig|80869.184.peg.2183	T3S	P1 family peptidase [*Paracidovorax citrulli*]
fig|80869.184.peg.2192	T3S	YitT family protein [*Paracidovorax citrulli*]
fig|80869.184.peg.2196	T3S	MlaD family protein [*Paracidovorax citrulli*]
fig|80869.184.peg.22	T3S	urease accessory protein UreG [*Paracidovorax citrulli*]
fig|80869.184.peg.2211	T3S	UDP-3-O-(3-hydroxymyristoyl)glucosamine N-acyltransferase [*Paracidovorax citrulli*]
fig|80869.184.peg.2244	T3S	glutamate--tRNA ligase [*Paracidovorax citrulli*]
fig|80869.184.peg.2245	T3S	DUF1624 domain-containing protein [*Paracidovorax citrulli*]
fig|80869.184.peg.2248	T3S	DUF72 domain-containing protein [*Paracidovorax citrulli*]
fig|80869.184.peg.2266	T3S	lipid A export permease/ATP-binding protein MsbA [*Paracidovorax citrulli*]
fig|80869.184.peg.2270	T3S	ThiF family adenylyltransferase [*Paracidovorax citrulli*]
fig|80869.184.peg.2286	T3S	cytochrome c553-like protein [*Paracidovorax citrulli* AAC00-1]
fig|80869.184.peg.2287	T3S	thioredoxin family protein [*Paracidovorax citrulli*]
fig|80869.184.peg.2295	T3S	hypothetical protein [*Paracidovorax citrulli*]
fig|80869.184.peg.23	T3S	urease accessory protein [*Paracidovorax citrulli*]

Prot	Pred	Description
fig|80869.184.peg.2312	T3S	helix-turn-helix transcriptional regulator [*Paracidovorax citrulli*]
fig|80869.184.peg.2357	T3S	Bifunctional protein PutA [*Paracidovorax citrulli*]
fig|80869.184.peg.2359	T3S	hypothetical protein [*Paracidovorax citrulli*]
fig|80869.184.peg.2433	T3S	ABC transporter permease [*Paracidovorax citrulli*]
fig|80869.184.peg.2526	T3S	MULTISPECIES: CysB family HTH-type transcriptional regulator [Comamonadaceae]
fig|80869.184.peg.2539	T3S	2OG-Fe dioxygenase family protein [*Paracidovorax citrulli*]
fig|80869.184.peg.2563	T3S	TetR/AcrR family transcriptional regulator [*Paracidovorax citrulli*]
fig|80869.184.peg.2586	T3S	RDD domain containing protein [*Paracidovorax citrulli* AAC00-1]
fig|80869.184.peg.2600	T3S	YbaN family protein [*Paracidovorax citrulli*]
fig|80869.184.peg.2610	T3S	ATP-binding protein [*Paracidovorax citrulli*]
fig|80869.184.peg.2617	T3S	hypothetical protein [*Paracidovorax citrulli*]
fig|80869.184.peg.2687	T3S	glycoside hydrolase family 5 protein [*Paracidovorax citrulli*]
fig|80869.184.peg.2699	T3S	protein of unknown function UPF0061 [*Paracidovorax citrulli* AAC00-1]
fig|80869.184.peg.2708	T3S	hypothetical protein [*Paracidovorax citrulli*]
fig|80869.184.peg.271	T3S	TetR/AcrR family transcriptional regulator [*Paracidovorax citrulli*]
fig|80869.184.peg.2710	T3S	structural protein P5 [*Paracidovorax citrulli*]
fig|80869.184.peg.2713	T3S	hypothetical protein [*Paracidovorax citrulli*]
fig|80869.184.peg.273	T3S	transcriptional repressor [*Paracidovorax citrulli*]
fig|80869.184.peg.2754	T3S	polyhydroxyalkanoate synthesis repressor PhaR [*Paracidovorax citrulli*]
fig|80869.184.peg.2756	T3S	type 1 glutamine amidotransferase domain-containing protein [*Paracidovorax citrulli*]
fig|80869.184.peg.2807	T3S	SDR family NAD(P)-dependent oxidoreductase [*Paracidovorax citrulli*]
fig|80869.184.peg.283	T3S	pyridoxamine 5'-phosphate oxidase [*Paracidovorax citrulli*]
fig|80869.184.peg.2837	T3S	heavy metal translocating P-type ATPase [*Paracidovorax citrulli*]
fig|80869.184.peg.2841	T3S	malonate--CoA ligase [*Paracidovorax citrulli*]
fig|80869.184.peg.2862	T3S	glycine betaine/L-proline ABC transporter permease ProW [*Paracidovorax citrulli*]
fig|80869.184.peg.2904	T3S	farnesyl-diphosphate farnesyltransferase [*Paracidovorax citrulli* AAC00-1]
fig|80869.184.peg.2907	T3S	DUF2069 domain-containing protein [*Paracidovorax citrulli*]
fig|80869.184.peg.2932	T3S	HNH/endonuclease VII fold toxin-2 domain-containing protein [*Paracidovorax citrulli*]
fig|80869.184.peg.2947	T3S	efflux transporter outer membrane subunit [*Paracidovorax citrulli*]
fig|80869.184.peg.2958	T3S	TOBE domain-containing protein [*Paracidovorax citrulli*]
fig|80869.184.peg.2963	T3S	BCCT family transporter [*Paracidovorax citrulli*]
fig|80869.184.peg.3003	T3S	N-acetylneuraminate epimerase [*Paracidovorax citrulli*]
fig|80869.184.peg.3040	T3S	TspO/MBR family protein [*Paracidovorax citrulli*]
fig|80869.184.peg.3045	T3S	hypothetical protein [*Paracidovorax citrulli*]
fig|80869.184.peg.3052	T3S	DUF4139 domain-containing protein [*Paracidovorax citrulli*]
fig|80869.184.peg.3078	T3S	pectate lyase [*Paracidovorax citrulli*]
fig|80869.184.peg.3081	T3S	hypothetical protein [*Paracidovorax citrulli*]
fig|80869.184.peg.3082	T3S	hypothetical protein [*Paracidovorax citrulli*]
fig|80869.184.peg.3092	T3S	hypothetical protein [*Paracidovorax citrulli*]
fig|80869.184.peg.3097	T3S	hypothetical protein [*Paracidovorax citrulli*]
fig|80869.184.peg.3117	T3S	uracil-DNA glycosylase [*Paracidovorax citrulli*]
fig|80869.184.peg.3133	T3S	hypothetical protein APS58_2360 [*Paracidovorax citrulli*]
fig|80869.184.peg.3135	T3S	pyrroline-5-carboxylate reductase [*Paracidovorax citrulli*]
fig|80869.184.peg.317	T3S	pseudouridine synthase [*Paracidovorax citrulli*]
fig|80869.184.peg.3182	T3S	FAD-linked oxidase C-terminal domain-containing protein [*Paracidovorax citrulli*]

Prot	Pred	Description
fig|80869.184.peg.3195	T3S	transcriptional regulator, AraC family [*Paracidovorax citrulli* AAC00-1]
fig|80869.184.peg.3197	T3S	NarK family nitrate/nitrite MFS transporter [*Paracidovorax citrulli*]
fig|80869.184.peg.321	T3S	translesion DNA synthesis-associated protein ImuA [*Paracidovorax citrulli*]
fig|80869.184.peg.3229	T3S	DUF2169 domain-containing protein [*Paracidovorax citrulli*]
fig|80869.184.peg.3261	T3S	putative zinc protease protein [*Paracidovorax citrulli* AAC00-1]
fig|80869.184.peg.3262	T3S	DNA-3-methyladenine glycosylase I [*Paracidovorax citrulli*]
fig|80869.184.peg.3277	T3S	M48 family metallopeptidase [*Paracidovorax citrulli*]
fig|80869.184.peg.3284	T3S	chromate efflux transporter [*Paracidovorax citrulli*]
fig|80869.184.peg.331	T3S	GTP cyclohydrolase subunit MoaA [*Paracidovorax citrulli* AAC00-1]
fig|80869.184.peg.334	T3S	gephyrin-like molybdotransferase Glp [*Paracidovorax citrulli*]
fig|80869.184.peg.3340	T3S	3-deoxy-7-phosphoheptulonate synthase [*Paracidovorax citrulli*]
fig|80869.184.peg.3353	T3S	ribonucleoside-diphosphate reductase subunit alpha [*Paracidovorax citrulli*]
fig|80869.184.peg.3387	T3S	hydroxymethylpyrimidine/phosphomethylpyrimidine kinase [*Paracidovorax citrulli*]
fig|80869.184.peg.3396	T3S	Holliday junction resolvase RuvX [*Paracidovorax citrulli*]
fig|80869.184.peg.3429	T3S	ABC transporter ATP-binding protein [*Paracidovorax citrulli*]
fig|80869.184.peg.3461	T3S	NCS1 family nucleobase:cation symporter-1 [*Paracidovorax citrulli*]
fig|80869.184.peg.3464	T3S	peptidoglycan-binding domain-containing protein [*Paracidovorax citrulli*]
fig|80869.184.peg.3491	T3S	penicillin-binding protein 1A [*Paracidovorax citrulli*]
fig|80869.184.peg.3501	T3S	protein of unknown function DUF1415 [*Paracidovorax citrulli* AAC00-1]
fig|80869.184.peg.3537	T3S	PelD GGDEF domain-containing protein [*Paracidovorax citrulli*]
fig|80869.184.peg.3598	T3S	GNAT family N-acetyltransferase [*Paracidovorax citrulli*]
fig|80869.184.peg.3606	T3S	4'-phosphopantetheinyl transferase superfamily protein [*Paracidovorax citrulli*]
fig|80869.184.peg.3607	T3S	hypothetical protein [*Paracidovorax citrulli*]
fig|80869.184.peg.3622	T3S	hypothetical protein C1O66_15085 [Kinneretia aquatilis]
fig|80869.184.peg.364	T3S	phosphate ABC transporter permease PstC [*Paracidovorax citrulli*]
fig|80869.184.peg.3688	T3S	helix-turn-helix domain-containing protein [*Paracidovorax citrulli*]
fig|80869.184.peg.369	T3S	Polyphosphate kinase [*Paracidovorax citrulli* AAC00-1]
fig|80869.184.peg.3718	T3S	glycerophosphodiester phosphodiesterase [*Paracidovorax citrulli*]
fig|80869.184.peg.3728	T3S	GGDEF domain-containing protein [*Paracidovorax citrulli*]
fig|80869.184.peg.3742	T3S	cardiolipin synthase [*Paracidovorax citrulli*]
fig|80869.184.peg.3796	T3S	SAM-dependent methyltransferase [*Paracidovorax citrulli*]
fig|80869.184.peg.382	T3S	hypothetical protein [*Paracidovorax citrulli*]
fig|80869.184.peg.383	T3S	D-serine/D-alanine/glycine transporter [*Paracidovorax citrulli*]
fig|80869.184.peg.3837	T3S	ketopantoate reductase [*Paracidovorax citrulli*]
fig|80869.184.peg.3863	T3S	precorrin-2 C(20)-methyltransferase [*Paracidovorax citrulli*]
fig|80869.184.peg.3867	T3S	sirohydrochlorin chelatase [*Paracidovorax citrulli*]
fig|80869.184.peg.3880	T3S	hypothetical protein C8E06_4186 [*Paracidovorax citrulli*]
fig|80869.184.peg.3889	T3S	ATP-binding cassette domain-containing protein [*Paracidovorax citrulli*]
fig|80869.184.peg.3497	T4S	Ig domain protein, group 1 domain protein [*Paracidovorax citrulli* AAC00-1]
fig|80869.184.peg.4037	T4S	serine/threonine protein kinase [*Paracidovorax citrulli*]
fig|80869.184.peg.835	T4S	hypothetical protein [*Paracidovorax citrulli*]
fig|80869.184.peg.819	T4S	acyl-CoA thioesterase [*Paracidovorax citrulli*]
fig|80869.184.peg.137	T4S	ABC transporter ATP-binding protein [*Paracidovorax citrulli*]
fig|80869.184.peg.483	T4S	Ku protein [*Paracidovorax citrulli*]
fig|80869.184.peg.4491	T4S	bacterioferritin [*Paracidovorax citrulli*]

Prot	Pred	Description
fig|80869.184.peg.2076	T4S	terminase small subunit [*Paracidovorax citrulli*]
fig|80869.184.peg.4768	T4S	molybdenum cofactor biosynthesis protein MoaE [*Paracidovorax citrulli*]
fig|80869.184.peg.2532	T4S	hypothetical protein [*Paracidovorax citrulli*]
fig|80869.184.peg.2026	T4S	isoaspartyl peptidase/L-asparaginase family protein [*Paracidovorax citrulli*]
fig|80869.184.peg.2139	T4S	DUF2800 domain-containing protein [*Paracidovorax citrulli*]
fig|80869.184.peg.874	T4S	RNA recognition motif domain-containing protein [*Paracidovorax citrulli*]
fig|80869.184.peg.2632	T4S	hypothetical protein [*Paracidovorax citrulli*]
fig|80869.184.peg.2324	T4S	DUF1795 domain-containing protein [*Paracidovorax citrulli*]
fig|80869.184.peg.683	T4S	hypothetical protein C8E08_4485 [*Paracidovorax citrulli*]
fig|80869.184.peg.440	T4S	MULTISPECIES: amino-acid N-acetyltransferase [Paracidovorax]
fig|80869.184.peg.4175	T4S	DUF924 family protein [*Paracidovorax citrulli*]
fig|80869.184.peg.2916	T4S	hypothetical protein [*Paracidovorax citrulli*]
fig|80869.184.peg.1850	T4S	50S ribosomal protein L11 [*Paracidovorax citrulli*]
fig|80869.184.peg.3838	T4S	IS5 family transposase, partial [Paracidovorax oryzae]
fig|80869.184.peg.1	T4S	AAA family ATPase [*Paracidovorax citrulli*]
fig|80869.184.peg.470	T4S	hypothetical protein [*Paracidovorax citrulli*]
fig|80869.184.peg.1787	T4S	MULTISPECIES: LysR family transcriptional regulator [Paracidovorax]
fig|80869.184.peg.3948	T4S	virulence RhuM family protein [*Paracidovorax citrulli*]
fig|80869.184.peg.3788	T4S	SDR family NAD(P)-dependent oxidoreductase [*Paracidovorax citrulli*]
fig|80869.184.peg.3846	T4S	hypothetical protein [*Paracidovorax citrulli*]
fig|80869.184.peg.1870	T4S	cryptochrome/photolyase family protein [*Paracidovorax citrulli*]
fig|80869.184.peg.4847	T4S	phosphopyruvate hydratase [*Paracidovorax citrulli*]
fig|80869.184.peg.4543	T4S	glutathione-regulated potassium-efflux system protein KefC [*Paracidovorax citrulli*]
fig|80869.184.peg.33	T4S	hypothetical protein [*Paracidovorax citrulli*]
fig|80869.184.peg.2423	T4S	hypothetical protein [*Paracidovorax citrulli*]
fig|80869.184.peg.2224	T4S	AAA family ATPase [*Paracidovorax citrulli*]
fig|80869.184.peg.1223	T4S	DUF3025 domain-containing protein [*Paracidovorax citrulli*]
fig|80869.184.peg.3787	T4S	serine/threonine protein kinase [*Paracidovorax citrulli*]
fig|80869.184.peg.4407	T4S	hypothetical protein [*Paracidovorax citrulli*]
fig|80869.184.peg.782	T4S	inositol monophosphatase family protein [*Paracidovorax citrulli*]
fig|80869.184.peg.1306	T4S	N-acetylmuramoyl-L-alanine amidase [*Paracidovorax citrulli*]
fig|80869.184.peg.3125	T4S	AraC family transcriptional regulator [*Paracidovorax citrulli*]
fig|80869.184.peg.4950	T4S	SMC-Scp complex subunit ScpB [*Paracidovorax citrulli*]
fig|80869.184.peg.2361	T4S	hypothetical protein [*Paracidovorax citrulli*]
fig|80869.184.peg.4144	T4S	Fic family protein [*Paracidovorax citrulli*]
fig|80869.184.peg.4389	T4S	MULTISPECIES: 4-hydroxy-3-methylbut-2-enyl diphosphate reductase [Paracidovorax]
fig|80869.184.peg.2300	T4S	SsrA-binding protein SmpB [*Paracidovorax citrulli*]
fig|80869.184.peg.2985	T4S	F0F1 ATP synthase subunit gamma [*Paracidovorax citrulli*]
fig|80869.184.peg.1010	T4S	hypothetical protein [*Paracidovorax citrulli*]
fig|80869.184.peg.2138	T4S	DUF2815 family protein [*Paracidovorax citrulli*]
fig|80869.184.peg.4258	T4S	hypothetical protein [*Paracidovorax citrulli*]
fig|80869.184.peg.340	T4S	transglycosylase SLT domain-containing protein [*Paracidovorax citrulli*]
fig|80869.184.peg.3100	T4S	type III secretion system outer membrane ring subunit SctC [*Paracidovorax citrulli*]
fig|80869.184.peg.2094	T4S	hypothetical protein [*Paracidovorax citrulli*]
fig|80869.184.peg.2797	T4S	XopE/AvrPphe family type III secretion system effector [*Paracidovorax citrulli*]

Prot	Pred	Description
fig|80869.184.peg.4309	T4S	5'-nucleotidase [*Paracidovorax citrulli*]
fig|80869.184.peg.3160	T4S	30S ribosomal protein S4 [*Paracidovorax citrulli*]
fig|80869.184.peg.432	T4S	adenylate kinase [*Paracidovorax citrulli*]
fig|80869.184.peg.2443	T4S	hypothetical protein [*Paracidovorax citrulli*]
fig|80869.184.peg.3573	T4S	hypothetical protein [*Paracidovorax citrulli*]
fig|80869.184.peg.3819	T4S	type IV pilin protein [*Paracidovorax citrulli*]
fig|80869.184.peg.1204	T4S	hypothetical protein [*Paracidovorax citrulli*]
fig|80869.184.peg.657	T4S	major facilitator superfamily MFS_1 [*Paracidovorax citrulli* AAC00-1]
fig|80869.184.peg.4492	T4S	BON domain-containing protein [*Paracidovorax citrulli*]
fig|80869.184.peg.454	T4S	MetQ/NlpA family ABC transporter substrate-binding protein [*Paracidovorax citrulli*]
fig|80869.184.peg.94	T4S	Bug family tripartite tricarboxylate transporter substrate binding protein [*Paracidovorax citrulli*]
fig|80869.184.peg.2218	T4S	Bug family tripartite tricarboxylate transporter substrate binding protein [*Paracidovorax citrulli*]
fig|80869.184.peg.448	T4S	sulfate ABC transporter substrate-binding protein [*Paracidovorax citrulli*]
fig|80869.184.peg.4176	T4S	OmpA family protein [*Paracidovorax citrulli*]
fig|80869.184.peg.2112	T4S	glycoside hydrolase family protein [*Paracidovorax citrulli*]
fig|80869.184.peg.1099	T4S	sigma-54-dependent transcriptional regulator [*Paracidovorax citrulli*]
fig|80869.184.peg.4310	T4S	EF-hand domain-containing protein [*Paracidovorax citrulli*]

*P_citrulli*_ZJU1106

Prot	Pred	Description
fig|80869.185.peg.3399	T3S	hypothetical protein [*Paracidovorax citrulli*]
fig|80869.185.peg.4979	T3S	serine/threonine protein kinase [*Paracidovorax citrulli*]
fig|80869.185.peg.5115	T3S	type III secretion system outer membrane ring subunit SctC [*Paracidovorax citrulli*]
fig|80869.185.peg.5320	T3S	redox-sensitive transcriptional activator SoxR [*Paracidovorax citrulli*]
fig|80869.185.peg.5985	T3S	multi-sensor hybrid histidine kinase [*Paracidovorax citrulli* AAC00-1]
fig|80869.185.peg.2390	T3S	acyl-CoA thioesterase [*Paracidovorax citrulli*]
fig|80869.185.peg.4557	T3S	DUF6531 domain-containing protein [*Paracidovorax citrulli*]
fig|80869.185.peg.4958	T3S	hypothetical protein Y695_03211 [Hydrogenophaga sp. T4]
fig|80869.185.peg.1205	T3S	ABC transporter ATP-binding protein [*Paracidovorax citrulli*]
fig|80869.185.peg.2864	T3S	MurR/RpiR family transcriptional regulator [*Paracidovorax citrulli*]
fig|80869.185.peg.2581	T3S	molybdenum cofactor biosynthesis protein MoaE [*Paracidovorax citrulli*]
fig|80869.185.peg.3783	T3S	Ku protein [*Paracidovorax citrulli*]
fig|80869.185.peg.316	T3S	bacterioferritin [*Paracidovorax citrulli*]
fig|80869.185.peg.147	T3S	terminase small subunit [*Paracidovorax citrulli*]
fig|80869.185.peg.1015	T3S	glutamine--tRNA ligase/YqeY domain fusion protein [*Paracidovorax citrulli*]
fig|80869.185.peg.1027	T3S	alpha/beta hydrolase [*Paracidovorax citrulli*]
fig|80869.185.peg.105	T3S	MULTISPECIES: TRAP transporter small permease [Paracidovorax]
fig|80869.185.peg.1099	T3S	ABC transporter transmembrane domain-containing protein [*Paracidovorax citrulli*]
fig|80869.185.peg.1152	T3S	DUF2169 domain-containing protein [*Paracidovorax citrulli*]
fig|80869.185.peg.1161	T3S	nucleotide exchange factor GrpE [*Paracidovorax citrulli*]
fig|80869.185.peg.1230	T3S	DNA topoisomerase (ATP-hydrolyzing) subunit B [*Paracidovorax citrulli*]
fig|80869.185.peg.1238	T3S	ABC-F family ATP-binding cassette domain-containing protein [*Paracidovorax citrulli*]

Prot	Pred	Description
fig|80869.185.peg.1267	T3S	CerR family C-terminal domain-containing protein [*Paracidovorax citrulli*]
fig|80869.185.peg.1280	T3S	hypothetical protein [*Paracidovorax citrulli*]
fig|80869.185.peg.1345	T3S	lipid A export permease/ATP-binding protein MsbA [*Paracidovorax citrulli*]
fig|80869.185.peg.1349	T3S	ThiF family adenylyltransferase [*Paracidovorax citrulli*]
fig|80869.185.peg.1375	T3S	YiaA/YiaB family inner membrane protein [*Paracidovorax citrulli*]
fig|80869.185.peg.1387	T3S	cytochrome o ubiquinol oxidase subunit IV [*Paracidovorax citrulli*]
fig|80869.185.peg.1389	T3S	MATE family efflux transporter [*Paracidovorax citrulli*]
fig|80869.185.peg.1422	T3S	glycerophosphodiester phosphodiesterase [*Paracidovorax citrulli*]
fig|80869.185.peg.1438	T3S	pseudouridine synthase [*Paracidovorax citrulli*]
fig|80869.185.peg.1473	T3S	Polyphosphate kinase [*Paracidovorax citrulli* AAC00-1]
fig|80869.185.peg.150	T3S	hypothetical protein [*Paracidovorax citrulli*]
fig|80869.185.peg.1533	T3S	hypothetical protein [*Paracidovorax citrulli*]
fig|80869.185.peg.1574	T3S	UDP-3-O-(3-hydroxymyristoyl)glucosamine N-acyltransferase [*Paracidovorax citrulli*]
fig|80869.185.peg.1591	T3S	argininosuccinate lyase [*Paracidovorax citrulli*]
fig|80869.185.peg.1615	T3S	hypothetical protein [*Paracidovorax citrulli*]
fig|80869.185.peg.1620	T3S	DODA-type extradiol aromatic ring-opening family dioxygenase [*Paracidovorax citrulli*]
fig|80869.185.peg.1627	T3S	Tetratricopeptide TPR_2 repeat protein [*Paracidovorax citrulli* AAC00-1]
fig|80869.185.peg.1639	T3S	glutamate--tRNA ligase [*Paracidovorax citrulli*]
fig|80869.185.peg.1640	T3S	DUF1624 domain-containing protein [*Paracidovorax citrulli*]
fig|80869.185.peg.1648	T3S	MarR family winged helix-turn-helix transcriptional regulator, partial [*Paracidovorax citrulli*]
fig|80869.185.peg.1673	T3S	urease accessory protein UreG [*Paracidovorax citrulli*]
fig|80869.185.peg.1776	T3S	TonB-dependent siderophore receptor [*Paracidovorax citrulli*]
fig|80869.185.peg.1777	T3S	MHS family citrate/tricarballylate:H+ symporter-like MFS transporter [*Paracidovorax citrulli*]
fig|80869.185.peg.1780	T3S	polyhydroxyalkanoate synthesis repressor PhaR [*Paracidovorax citrulli*]
fig|80869.185.peg.1782	T3S	type 1 glutamine amidotransferase domain-containing protein [*Paracidovorax citrulli*]
fig|80869.185.peg.1806	T3S	SLC13 family permease [*Paracidovorax citrulli*]
fig|80869.185.peg.1873	T3S	MULTISPECIES: alpha/beta hydrolase [Paracidovorax]
fig|80869.185.peg.1914	T3S	potassium-transporting ATPase subunit KdpB [*Paracidovorax citrulli*]
fig|80869.185.peg.1916	T3S	malonate--CoA ligase [*Paracidovorax citrulli*]
fig|80869.185.peg.192	T3S	aromatic ring-hydroxylating dioxygenase subunit alpha [*Paracidovorax citrulli*]
fig|80869.185.peg.1920	T3S	heavy metal translocating P-type ATPase [*Paracidovorax citrulli*]
fig|80869.185.peg.1922	T3S	2OG-Fe dioxygenase family protein [*Paracidovorax citrulli*]
fig|80869.185.peg.1972	T3S	uncharacterized protein DUF2132 [*Paracidovorax citrulli*]
fig|80869.185.peg.1986	T3S	flagellar hook assembly protein FlgD [*Paracidovorax citrulli*]
fig|80869.185.peg.1998	T3S	neutral zinc metallopeptidase [*Paracidovorax citrulli*]
fig|80869.185.peg.2056	T3S	BON domain-containing protein [*Paracidovorax citrulli*]
fig|80869.185.peg.2057	T3S	SulP family inorganic anion transporter [*Paracidovorax citrulli*]
fig|80869.185.peg.2098	T3S	P1 family peptidase [*Paracidovorax citrulli*]
fig|80869.185.peg.2119	T3S	hotdog fold thioesterase [*Paracidovorax citrulli*]
fig|80869.185.peg.2183	T3S	isoleucine--tRNA ligase [*Paracidovorax citrulli*]
fig|80869.185.peg.2322	T3S	BCCT family transporter [*Paracidovorax citrulli*]
fig|80869.185.peg.233	T3S	helix-turn-helix transcriptional regulator [*Paracidovorax citrulli*]
fig|80869.185.peg.2330	T3S	chemoreceptor glutamine deamidase CheD [*Paracidovorax citrulli*]

Prot	Pred	Description
fig|80869.185.peg.2331	T3S	CheR family methyltransferase [*Paracidovorax citrulli*]
fig|80869.185.peg.2342	T3S	D-serine/D-alanine/glycine transporter [*Paracidovorax citrulli*]
fig|80869.185.peg.2363	T3S	hypothetical protein [*Paracidovorax citrulli*]
fig|80869.185.peg.2382	T3S	ComEC/Rec2 family competence protein [Paracidovorax avenae]
fig|80869.185.peg.2388	T3S	pyridoxal kinase PdxY [*Paracidovorax citrulli*]
fig|80869.185.peg.2408	T3S	tryptophan 2,3-dioxygenase [*Paracidovorax citrulli*]
fig|80869.185.peg.2410	T3S	RecQ family ATP-dependent DNA helicase [*Paracidovorax citrulli*]
fig|80869.185.peg.2520	T3S	hypothetical protein [*Paracidovorax citrulli*]
fig|80869.185.peg.2559	T3S	thioredoxin family protein [*Paracidovorax citrulli*]
fig|80869.185.peg.2585	T3S	multiple monosaccharide ABC transporter permease [*Paracidovorax citrulli*]
fig|80869.185.peg.2665	T3S	hypothetical protein MPLDJ20_410001 [Mesorhizobium plurifarium]
fig|80869.185.peg.2695	T3S	luciferase family protein [*Paracidovorax citrulli* AAC00-1]
fig|80869.185.peg.2720	T3S	gamma-glutamyl-gamma-aminobutyrate hydrolase family protein [*Paracidovorax citrulli*]
fig|80869.185.peg.2737	T3S	EAL domain-containing protein [*Paracidovorax citrulli*]
fig|80869.185.peg.2751	T3S	hypothetical protein APS58_2360 [*Paracidovorax citrulli*]
fig|80869.185.peg.2807	T3S	ketopantoate reductase [*Paracidovorax citrulli*]
fig|80869.185.peg.2823	T3S	amino acid ABC transporter ATP-binding protein [*Paracidovorax citrulli*]
fig|80869.185.peg.2855	T3S	Uncharacterised protein [Yersinia enterocolitica]
fig|80869.185.peg.2888	T3S	putative avirulence protein AvrRxo1 [*Paracidovorax citrulli* AAC00-1]
fig|80869.185.peg.2903	T3S	ABC-F family ATP-binding cassette domain-containing protein [*Paracidovorax citrulli*]
fig|80869.185.peg.2914	T3S	peptidoglycan-binding domain-containing protein, partial [*Paracidovorax citrulli*]
fig|80869.185.peg.2916	T3S	MlaD family protein [*Paracidovorax citrulli*]
fig|80869.185.peg.2958	T3S	DHA2 family efflux MFS transporter permease subunit [*Paracidovorax citrulli*]
fig|80869.185.peg.2990	T3S	SAM-dependent methyltransferase [*Paracidovorax citrulli*]
fig|80869.185.peg.3006	T3S	hypothetical protein [*Paracidovorax citrulli*]
fig|80869.185.peg.3032	T3S	AraC family transcriptional regulator, partial [*Paracidovorax citrulli*]
fig|80869.185.peg.3035	T3S	2-isopropylmalate synthase [*Paracidovorax citrulli*]
fig|80869.185.peg.3041	T3S	succinate-semialdehyde dehydrogenase/glutarate-semialdehyde dehydrogenase [*Paracidovorax citrulli*]
fig|80869.185.peg.3050	T3S	MFS transporter [*Paracidovorax citrulli*]
fig|80869.185.peg.3068	T3S	hypothetical protein APS58_2974 [*Paracidovorax citrulli*]
fig|80869.185.peg.3091	T3S	group I truncated hemoglobin [*Paracidovorax citrulli*]
fig|80869.185.peg.3103	T3S	septal ring lytic transglycosylase RlpA family protein [*Paracidovorax citrulli*]
fig|80869.185.peg.3136	T3S	flagellar basal body protein [*Paracidovorax citrulli*]
fig|80869.185.peg.3138	T3S	hydroxymethylpyrimidine/phosphomethylpyrimidine kinase [*Paracidovorax citrulli*]
fig|80869.185.peg.3155	T3S	exodeoxyribonuclease VII small subunit [*Paracidovorax citrulli*]
fig|80869.185.peg.3160	T3S	pyridoxamine 5'-phosphate oxidase [*Paracidovorax citrulli*]
fig|80869.185.peg.3226	T3S	N-acetylneuraminate epimerase [*Paracidovorax citrulli*]
fig|80869.185.peg.3272	T3S	NCS1 family nucleobase:cation symporter-1 [*Paracidovorax citrulli*]
fig|80869.185.peg.3282	T3S	hypothetical protein [Diaphorobacter nitroreducens]
fig|80869.185.peg.331	T3S	3-methyl-2-oxobutanoate hydroxymethyltransferase [*Paracidovorax citrulli*]
fig|80869.185.peg.3370	T3S	hypothetical protein [*Paracidovorax citrulli*]
fig|80869.185.peg.3372	T3S	Bifunctional protein PutA [*Paracidovorax citrulli*]
fig|80869.185.peg.3393	T3S	AmpG family muropeptide MFS transporter [*Paracidovorax citrulli*]

Prot	Pred	Description
fig|80869.185.peg.3396	T3S	putative amino-acid metabolite efflux pump [*Paracidovorax citrulli*]
fig|80869.185.peg.3438	T3S	TOBE domain-containing protein [*Paracidovorax citrulli*]
fig|80869.185.peg.3503	T3S	MBL fold metallo-hydrolase [*Paracidovorax citrulli*]
fig|80869.185.peg.3524	T3S	DUF2069 domain-containing protein [*Paracidovorax citrulli*]
fig|80869.185.peg.3534	T3S	GNAT family N-acetyltransferase [*Paracidovorax citrulli*]
fig|80869.185.peg.3556	T3S	TetR/AcrR family transcriptional regulator [*Paracidovorax citrulli*]
fig|80869.185.peg.3569	T3S	uracil-DNA glycosylase [*Paracidovorax citrulli*]
fig|80869.185.peg.3632	T3S	AMIN domain-containing protein, partial [*Paracidovorax citrulli*]
fig|80869.185.peg.3639	T3S	ABC transporter permease [*Paracidovorax citrulli*]
fig|80869.185.peg.3649	T3S	AsmA family protein [*Paracidovorax citrulli*]
fig|80869.185.peg.3677	T3S	transcriptional regulator domain protein [*Paracidovorax citrulli* AAC00-1]
fig|80869.185.peg.3691	T3S	phospholipase A [Paracidovorax avenae]
fig|80869.185.peg.3704	T3S	protein of unknown function DUF1653 [*Paracidovorax citrulli* AAC00-1]
fig|80869.185.peg.3716	T3S	GTP cyclohydrolase subunit MoaA [*Paracidovorax citrulli* AAC00-1]
fig|80869.185.peg.3725	T3S	CDP-6-deoxy-delta-3,4-glucoseen reductase [*Paracidovorax citrulli*]
fig|80869.185.peg.374	T3S	LON peptidase substrate-binding domain-containing protein [*Paracidovorax citrulli*]
fig|80869.185.peg.3745	T3S	sirohydrochlorin chelatase [*Paracidovorax citrulli*]
fig|80869.185.peg.3755	T3S	bifunctional metallophosphatase/5'-nucleotidase [*Paracidovorax citrulli*]
fig|80869.185.peg.3779	T3S	ammonium transporter [*Paracidovorax citrulli*]
fig|80869.185.peg.38	T3S	Conjugal transfer protein TraX [*Paracidovorax citrulli*]
fig|80869.185.peg.3808	T3S	hypothetical protein [*Paracidovorax citrulli*]
fig|80869.185.peg.381	T3S	lysine--tRNA ligase [*Paracidovorax citrulli*]
fig|80869.185.peg.3812	T3S	Cupin 2, conserved barrel domain protein [*Paracidovorax citrulli* AAC00-1]
fig|80869.185.peg.3816	T3S	sensor histidine kinase [Paracidovorax oryzae]
fig|80869.185.peg.382	T3S	Phytochrome-like protein cph2 [*Paracidovorax citrulli*]
fig|80869.185.peg.3829	T3S	ribonuclease catalytic domain-containing protein [*Paracidovorax citrulli*]
fig|80869.185.peg.385	T3S	MFS transporter [*Paracidovorax citrulli*]
fig|80869.185.peg.3874	T3S	ATP-binding protein [*Paracidovorax citrulli*]
fig|80869.185.peg.3877	T3S	YitT family protein [*Paracidovorax citrulli*]
fig|80869.185.peg.3903	T3S	3-deoxy-7-phosphoheptulonate synthase [*Paracidovorax citrulli*]
fig|80869.185.peg.3934	T3S	PhaM family polyhydroxyalkanoate granule multifunctional regulatory protein [*Paracidovorax citrulli*]
fig|80869.185.peg.4007	T3S	ABC transporter ATP-binding protein [*Paracidovorax citrulli*]
fig|80869.185.peg.4017	T3S	glycine betaine/L-proline ABC transporter permease ProW [*Paracidovorax citrulli*]
fig|80869.185.peg.4038	T3S	ABC transporter ATP-binding protein [*Paracidovorax citrulli*]
fig|80869.185.peg.4041	T3S	SDR family NAD(P)-dependent oxidoreductase [*Paracidovorax citrulli*]
fig|80869.185.peg.4044	T3S	septum formation protein [*Paracidovorax citrulli*]
fig|80869.185.peg.4071	T3S	hypothetical protein [*Paracidovorax citrulli*]
fig|80869.185.peg.4122	T3S	Holliday junction resolvase RuvX [*Paracidovorax citrulli*]
fig|80869.185.peg.4127	T3S	ribonucleoside-diphosphate reductase subunit alpha [*Paracidovorax citrulli*]
fig|80869.185.peg.4146	T3S	MULTISPECIES: CysB family HTH-type transcriptional regulator [Comamonadaceae]
fig|80869.185.peg.4177	T3S	hypothetical protein [*Paracidovorax citrulli*]
fig|80869.185.peg.4216	T3S	alpha/beta hydrolase [*Paracidovorax citrulli*]
fig|80869.185.peg.4252	T3S	hypothetical protein [*Paracidovorax citrulli*]
fig|80869.185.peg.4269	T3S	CaiB/BaiF CoA transferase family protein [*Paracidovorax citrulli*]

Prot	Pred	Description
fig|80869.185.peg.432	T3S	histidyl-tRNA synthetase [*Paracidovorax citrulli* AAC00-1]
fig|80869.185.peg.4327	T3S	acetyl-CoA carboxylase biotin carboxylase subunit [*Paracidovorax citrulli*]
fig|80869.185.peg.4351	T3S	flagellin [*Paracidovorax citrulli*]
fig|80869.185.peg.4352	T3S	flagellin [*Paracidovorax citrulli*]
fig|80869.185.peg.437	T3S	GTPase HflX [*Paracidovorax citrulli*]
fig|80869.185.peg.4372	T3S	winged helix-turn-helix transcriptional regulator [*Paracidovorax citrulli*]
fig|80869.185.peg.3399	T4S	hypothetical protein [*Paracidovorax citrulli*]
fig|80869.185.peg.4979	T4S	serine/threonine protein kinase [*Paracidovorax citrulli*]
fig|80869.185.peg.5115	T4S	type III secretion system outer membrane ring subunit SctC [*Paracidovorax citrulli*]
fig|80869.185.peg.5320	T4S	redox-sensitive transcriptional activator SoxR [*Paracidovorax citrulli*]
fig|80869.185.peg.5985	T4S	multi-sensor hybrid histidine kinase [*Paracidovorax citrulli* AAC00-1]
fig|80869.185.peg.2390	T4S	acyl-CoA thioesterase [*Paracidovorax citrulli*]
fig|80869.185.peg.4557	T4S	DUF6531 domain-containing protein [*Paracidovorax citrulli*]
fig|80869.185.peg.4958	T4S	hypothetical protein Y695_03211 [Hydrogenophaga sp. T4]
fig|80869.185.peg.1205	T4S	ABC transporter ATP-binding protein [*Paracidovorax citrulli*]
fig|80869.185.peg.2864	T4S	MurR/RpiR family transcriptional regulator [*Paracidovorax citrulli*]
fig|80869.185.peg.2581	T4S	molybdenum cofactor biosynthesis protein MoaE [*Paracidovorax citrulli*]
fig|80869.185.peg.3783	T4S	Ku protein [*Paracidovorax citrulli*]
fig|80869.185.peg.316	T4S	bacterioferritin [*Paracidovorax citrulli*]
fig|80869.185.peg.147	T4S	terminase small subunit [*Paracidovorax citrulli*]
fig|80869.185.peg.1715	T4S	hypothetical protein [*Paracidovorax citrulli*]
fig|80869.185.peg.396	T4S	RNA recognition motif domain-containing protein [*Paracidovorax citrulli*]
fig|80869.185.peg.5907	T4S	hypothetical protein [*Paracidovorax citrulli*]
fig|80869.185.peg.3170	T4S	DUF1795 domain-containing protein [*Paracidovorax citrulli*]
fig|80869.185.peg.906	T4S	Ig-like domain-containing protein [*Paracidovorax citrulli*]
fig|80869.185.peg.3867	T4S	OmpA family protein [*Paracidovorax citrulli*]
fig|80869.185.peg.1043	T4S	YgiQ family radical SAM protein [*Paracidovorax citrulli*]
fig|80869.185.peg.3937	T4S	DNA mismatch repair endonuclease MutL, partial [*Paracidovorax citrulli*]
fig|80869.185.peg.5470	T4S	hypothetical protein [*Paracidovorax citrulli*]
fig|80869.185.peg.4083	T4S	lipopolysaccharide transport periplasmic protein LptA [*Paracidovorax citrulli*]
fig|80869.185.peg.3862	T4S	DEAD/DEAH box helicase [*Paracidovorax citrulli*]
fig|80869.185.peg.2937	T4S	MULTISPECIES: amino-acid N-acetyltransferase [Paracidovorax]
fig|80869.185.peg.3868	T4S	DUF924 family protein [*Paracidovorax citrulli*]
fig|80869.185.peg.2238	T4S	type III secretion system outer membrane ring subunit SctC [*Paracidovorax citrulli*]
fig|80869.185.peg.475	T4S	50S ribosomal protein L11 [*Paracidovorax citrulli*]
fig|80869.185.peg.4474	T4S	response regulator [*Paracidovorax citrulli*]
fig|80869.185.peg.1289	T4S	hypothetical protein [*Paracidovorax citrulli*]
fig|80869.185.peg.3968	T4S	MFS transporter [*Paracidovorax citrulli*]
fig|80869.185.peg.4305	T4S	AAA family ATPase, partial [Paracidovorax cattleyae]
fig|80869.185.peg.5913	T4S	5'-nucleotidase [*Paracidovorax citrulli*]
fig|80869.185.peg.3014	T4S	PP2C family protein-serine/threonine phosphatase [*Paracidovorax citrulli*]
fig|80869.185.peg.4073	T4S	hypothetical protein [*Paracidovorax citrulli*]
fig|80869.185.peg.2517	T4S	XopE/AvrPphe family type III secretion system effector [*Paracidovorax citrulli*]
fig|80869.185.peg.1069	T4S	hypothetical protein [*Paracidovorax citrulli*]
fig|80869.185.peg.1315	T4S	phosphopyruvate hydratase [*Paracidovorax citrulli*]

Prot	Pred	Description
fig|80869.185.peg.5710	T4S	hypothetical protein [*Paracidovorax citrulli*]
fig|80869.185.peg.1210	T4S	hypothetical protein [*Paracidovorax citrulli*]
fig|80869.185.peg.424	T4S	AAA family ATPase [*Paracidovorax citrulli*]
fig|80869.185.peg.3208	T4S	DUF3025 domain-containing protein [*Paracidovorax citrulli*]
fig|80869.185.peg.3015	T4S	serine/threonine protein kinase [*Paracidovorax citrulli*]
fig|80869.185.peg.2872	T4S	hypothetical protein [*Paracidovorax citrulli*]
fig|80869.185.peg.3185	T4S	inositol monophosphatase family protein [*Paracidovorax citrulli*]
fig|80869.185.peg.3253	T4S	N-acetylmuramoyl-L-alanine amidase [*Paracidovorax citrulli*]
fig|80869.185.peg.4520	T4S	LysM peptidoglycan-binding domain-containing protein [*Paracidovorax citrulli*]
fig|80869.185.peg.1581	T4S	Fic family protein [*Paracidovorax citrulli*]
fig|80869.185.peg.2441	T4S	4-hydroxy-3-methylbut-2-enyl diphosphate reductase [*Paracidovorax citrulli*]
fig|80869.185.peg.3306	T4S	SsrA-binding protein SmpB [*Paracidovorax citrulli*]
fig|80869.185.peg.559	T4S	F0F1 ATP synthase subunit gamma [*Paracidovorax citrulli*]
fig|80869.185.peg.274	T4S	hypothetical protein [*Paracidovorax citrulli*]
fig|80869.185.peg.1963	T4S	transglycosylase SLT domain-containing protein [*Paracidovorax citrulli*]
fig|80869.185.peg.128	T4S	hypothetical protein [*Paracidovorax citrulli*]
fig|80869.185.peg.607	T4S	30S ribosomal protein S4 [*Paracidovorax citrulli*]
fig|80869.185.peg.1834	T4S	cryptochrome/photolyase family protein [*Paracidovorax citrulli*]
fig|80869.185.peg.4532	T4S	RHS repeat-associated core domain-containing protein [*Paracidovorax citrulli*]
fig|80869.185.peg.4344	T4S	adenylate kinase [*Paracidovorax citrulli*]
fig|80869.185.peg.5580	T4S	5-oxoprolinase subunit PxpB [*Paracidovorax citrulli*]
fig|80869.185.peg.726	T4S	type IV pilin protein [*Paracidovorax citrulli*]
fig|80869.185.peg.4013	T4S	trifunctional transcriptional regulator/proline dehydrogenase/L-glutamate gamma-semialdehyde dehydrogenase [*Paracidovorax citrulli*]
fig|80869.185.peg.1855	T4S	hypothetical protein [*Paracidovorax citrulli*]
fig|80869.185.peg.4510	T4S	BMP family ABC transporter substrate-binding protein [*Paracidovorax citrulli*]
fig|80869.185.peg.3969	T4S	methyl-accepting chemotaxis protein [*Paracidovorax citrulli*]
fig|80869.185.peg.771	T4S	glutaredoxin family protein [*Paracidovorax citrulli*]
fig|80869.185.peg.1996	T4S	glutathione-regulated potassium-efflux system protein KefC [*Paracidovorax citrulli*]
fig|80869.185.peg.2681	T4S	methyl-accepting chemotaxis protein [*Paracidovorax citrulli*]
fig|80869.185.peg.315	T4S	BON domain-containing protein [*Paracidovorax citrulli*]
fig|80869.185.peg.3645	T4S	MetQ/NlpA family ABC transporter substrate-binding protein [*Paracidovorax citrulli*]
fig|80869.185.peg.5501	T4S	late embryogenesis abundant protein, group 3 [Brassica rapa]
fig|80869.185.peg.1647	T4S	Bug family tripartite tricarboxylate transporter substrate binding protein [*Paracidovorax citrulli*]
fig|80869.185.peg.2253	T4S	Bug family tripartite tricarboxylate transporter substrate binding protein [*Paracidovorax citrulli*]
fig|80869.185.peg.3163	T4S	sulfate ABC transporter substrate-binding protein [*Paracidovorax citrulli*]
fig|80869.185.peg.2067	T4S	tetratricopeptide repeat protein [*Paracidovorax citrulli*]
fig|80869.185.peg.2111	T4S	hypothetical protein [*Paracidovorax citrulli*]
fig|80869.185.peg.869	T4S	glycoside hydrolase family protein [*Paracidovorax citrulli*]
fig|80869.185.peg.3386	T4S	transglycosylase domain-containing protein [*Paracidovorax citrulli*]
fig|80869.185.peg.3484	T4S	sigma-54-dependent transcriptional regulator [*Paracidovorax citrulli*]

*P_citrulli*_KACC17001

Prot	Pred	Description
fig|80869.200.peg.1818	T3S	hypothetical protein [*Paracidovorax citrulli*]
fig|80869.200.peg.1928	T3S	major facilitator superfamily MFS_1 [*Paracidovorax citrulli* AAC00-1]
fig|80869.200.peg.3907	T3S	Ig domain protein, group 1 domain protein [*Paracidovorax citrulli* AAC00-1]
fig|80869.200.peg.1734	T3S	acyl-CoA thioesterase [*Paracidovorax citrulli*]
fig|80869.200.peg.2417	T3S	STY0301 family protein [*Paracidovorax citrulli*]
fig|80869.200.peg.2570	T3S	ABC transporter ATP-binding protein [*Paracidovorax citrulli*]
fig|80869.200.peg.2307	T3S	Ku protein [*Paracidovorax citrulli*]
fig|80869.200.peg.1025	T3S	bacterioferritin [*Paracidovorax citrulli*]
fig|80869.200.peg.3207	T3S	terminase small subunit [*Paracidovorax citrulli*]
fig|80869.200.peg.1008	T3S	MATE family efflux transporter [*Paracidovorax citrulli*]
fig|80869.200.peg.1018	T3S	signal recognition particle-docking protein FtsY [*Paracidovorax citrulli*]
fig|80869.200.peg.1039	T3S	3-methyl-2-oxobutanoate hydroxymethyltransferase [*Paracidovorax citrulli*]
fig|80869.200.peg.1054	T3S	hypothetical protein [*Paracidovorax citrulli*]
fig|80869.200.peg.1055	T3S	YitT family protein [*Paracidovorax citrulli*]
fig|80869.200.peg.1067	T3S	hypothetical protein [*Paracidovorax citrulli*]
fig|80869.200.peg.107	T3S	MBL fold metallo-hydrolase [*Paracidovorax citrulli*]
fig|80869.200.peg.1172	T3S	GNAT family N-acetyltransferase [*Paracidovorax citrulli*]
fig|80869.200.peg.1177	T3S	4'-phosphopantetheinyl transferase superfamily protein [*Paracidovorax citrulli*]
fig|80869.200.peg.1178	T3S	hypothetical protein [*Paracidovorax citrulli*]
fig|80869.200.peg.122	T3S	DUF1800 family protein [*Paracidovorax citrulli*]
fig|80869.200.peg.124	T3S	putative amino-acid metabolite efflux pump [*Paracidovorax citrulli*]
fig|80869.200.peg.1284	T3S	Cardiolipin synthase B [*Paracidovorax citrulli*]
fig|80869.200.peg.1337	T3S	SAM-dependent methyltransferase [*Paracidovorax citrulli*]
fig|80869.200.peg.1375	T3S	urease accessory protein UreG [*Paracidovorax citrulli*]
fig|80869.200.peg.1376	T3S	urease accessory protein [*Paracidovorax citrulli*]
fig|80869.200.peg.1416	T3S	PilZ domain-containing protein [*Paracidovorax citrulli*]
fig|80869.200.peg.1459	T3S	isoleucine--tRNA ligase [*Paracidovorax citrulli*]
fig|80869.200.peg.1474	T3S	protein of unknown function DUF1653 [*Paracidovorax citrulli* AAC00-1]
fig|80869.200.peg.1478	T3S	hypothetical protein [*Paracidovorax citrulli*]
fig|80869.200.peg.1569	T3S	argininosuccinate lyase [*Paracidovorax citrulli*]
fig|80869.200.peg.1598	T3S	CDP-6-deoxy-delta-3,4-glucoseen reductase [*Paracidovorax citrulli*]
fig|80869.200.peg.1607	T3S	hypothetical protein [*Paracidovorax citrulli*]
fig|80869.200.peg.1621	T3S	ABC-F family ATP-binding cassette domain-containing protein [*Paracidovorax citrulli*]
fig|80869.200.peg.1623	T3S	uroporphyrinogen-III C-methyltransferase [*Paracidovorax citrulli*]
fig|80869.200.peg.1625	T3S	hydroxymethylbilane synthase [*Paracidovorax citrulli*]
fig|80869.200.peg.1626	T3S	phosphoenolpyruvate carboxylase [*Paracidovorax citrulli*]
fig|80869.200.peg.1627	T3S	YccS family putative transporter [*Paracidovorax citrulli*]
fig|80869.200.peg.1629	T3S	AI-2E family transporter [*Paracidovorax citrulli*]
fig|80869.200.peg.1635	T3S	SfnB family sulfur acquisition oxidoreductase [*Paracidovorax citrulli*]
fig|80869.200.peg.1640	T3S	exodeoxyribonuclease VII small subunit [*Paracidovorax citrulli*]
fig|80869.200.peg.1652	T3S	alpha-1,4-glucan--maltose-1-phosphate maltosyltransferase [*Paracidovorax citrulli*]
fig|80869.200.peg.1655	T3S	BCCT family transporter [*Paracidovorax citrulli*]
fig|80869.200.peg.1660	T3S	TOBE domain-containing protein [*Paracidovorax citrulli*]

Prot	Pred	Description
fig|80869.200.peg.167	T3S	CerR family C-terminal domain-containing protein [*Paracidovorax citrulli*]
fig|80869.200.peg.1671	T3S	efflux transporter outer membrane subunit [*Paracidovorax citrulli*]
fig|80869.200.peg.1713	T3S	succinate-semialdehyde dehydrogenase/glutarate-semialdehyde dehydrogenase [*Paracidovorax citrulli*]
fig|80869.200.peg.1736	T3S	uroporphyrinogen-III C-methyltransferase [*Paracidovorax citrulli*]
fig|80869.200.peg.1753	T3S	carboxyl transferase domain-containing protein [*Paracidovorax citrulli*]
fig|80869.200.peg.1764	T3S	MFS transporter [*Paracidovorax citrulli*]
fig|80869.200.peg.1786	T3S	hypothetical protein FRC90_13055 [*Paracidovorax citrulli*]
fig|80869.200.peg.1816	T3S	hypothetical protein [*Paracidovorax citrulli*]
fig|80869.200.peg.1833	T3S	NAD-dependent succinate-semialdehyde dehydrogenase [*Paracidovorax citrulli*]
fig|80869.200.peg.1847	T3S	transcriptional regulator, GntR family [*Paracidovorax citrulli*]
fig|80869.200.peg.1862	T3S	cytochrome o ubiquinol oxidase subunit IV [*Paracidovorax citrulli*]
fig|80869.200.peg.1895	T3S	multiple monosaccharide ABC transporter permease [*Paracidovorax citrulli*]
fig|80869.200.peg.1901	T3S	SDR family NAD(P)-dependent oxidoreductase [*Paracidovorax citrulli*]
fig|80869.200.peg.1902	T3S	SMP-30/gluconolactonase/LRE family protein [*Paracidovorax citrulli*]
fig|80869.200.peg.1909	T3S	GAF domain-containing sensor histidine kinase [*Paracidovorax citrulli*]
fig|80869.200.peg.1916	T3S	MFS transporter [*Paracidovorax citrulli*]
fig|80869.200.peg.1918	T3S	redox-sensitive transcriptional activator SoxR [*Paracidovorax citrulli*]
fig|80869.200.peg.1922	T3S	DHH family phosphoesterase [*Paracidovorax citrulli*]
fig|80869.200.peg.1932	T3S	Murein DD-endopeptidase MepM [*Paracidovorax citrulli*]
fig|80869.200.peg.1935	T3S	alpha/beta hydrolase [*Paracidovorax citrulli*]
fig|80869.200.peg.1947	T3S	glutamine--tRNA ligase/YqeY domain fusion protein [*Paracidovorax citrulli*]
fig|80869.200.peg.1952	T3S	DODA-type extradiol aromatic ring-opening family dioxygenase [*Paracidovorax citrulli*]
fig|80869.200.peg.2014	T3S	TetR/AcrR family transcriptional regulator [*Paracidovorax citrulli*]
fig|80869.200.peg.2016	T3S	transcriptional repressor [*Paracidovorax citrulli*]
fig|80869.200.peg.2027	T3S	pyridoxamine 5'-phosphate oxidase [*Paracidovorax citrulli*]
fig|80869.200.peg.2118	T3S	DEAD/DEAH box helicase [*Paracidovorax citrulli*]
fig|80869.200.peg.2128	T3S	hypothetical protein CQB05_01060 [*Paracidovorax citrulli*]
fig|80869.200.peg.2148	T3S	pseudouridine synthase [*Paracidovorax citrulli*]
fig|80869.200.peg.2152	T3S	translesion DNA synthesis-associated protein ImuA [*Paracidovorax citrulli*]
fig|80869.200.peg.2163	T3S	GTP cyclohydrolase subunit MoaA [*Paracidovorax citrulli* AAC00-1]
fig|80869.200.peg.2165	T3S	GNAT family N-acetyltransferase [*Paracidovorax citrulli*]
fig|80869.200.peg.2166	T3S	gephyrin-like molybdotransferase Glp [*Paracidovorax citrulli*]
fig|80869.200.peg.2196	T3S	phosphate ABC transporter permease PstC [*Paracidovorax citrulli*]
fig|80869.200.peg.2201	T3S	Polyphosphate kinase [*Paracidovorax citrulli* AAC00-1]
fig|80869.200.peg.2215	T3S	hypothetical protein [*Paracidovorax citrulli*]
fig|80869.200.peg.2216	T3S	D-serine/D-alanine/glycine transporter [*Paracidovorax citrulli*]
fig|80869.200.peg.2226	T3S	TonB-dependent receptor family protein [*Paracidovorax citrulli*]
fig|80869.200.peg.227	T3S	hypothetical protein [*Paracidovorax citrulli*]
fig|80869.200.peg.2270	T3S	ATP-dependent RNA helicase HrpA [*Paracidovorax citrulli*]
fig|80869.200.peg.23	T3S	DNA topoisomerase (ATP-hydrolyzing) subunit B [*Paracidovorax citrulli*]
fig|80869.200.peg.2348	T3S	EAL domain-containing protein [*Paracidovorax citrulli*]
fig|80869.200.peg.2381	T3S	PAS domain S-box protein [*Paracidovorax citrulli*]
fig|80869.200.peg.2425	T3S	KGG domain-containing protein [*Paracidovorax citrulli*]

Prot	Pred	Description
fig|80869.200.peg.243	T3S	gamma-glutamyl-gamma-aminobutyrate hydrolase family protein [*Paracidovorax citrulli*]
fig|80869.200.peg.2478	T3S	glycoside hydrolase family 5 protein [*Paracidovorax citrulli*]
fig|80869.200.peg.2491	T3S	Uncharacterized conserved protein YdiU, UPF0061 family [*Paracidovorax citrulli*]
fig|80869.200.peg.2504	T3S	structural protein P5 [*Paracidovorax citrulli*]
fig|80869.200.peg.2507	T3S	hypothetical protein [*Paracidovorax citrulli*]
fig|80869.200.peg.2512	T3S	conserved hypothetical protein [*Paracidovorax citrulli* AAC00-1]
fig|80869.200.peg.258	T3S	nucleotidyltransferase family protein [*Paracidovorax citrulli*]
fig|80869.200.peg.261	T3S	phosphatase PAP2 family protein [*Paracidovorax citrulli*]
fig|80869.200.peg.2628	T3S	BON domain-containing protein [*Paracidovorax citrulli*]
fig|80869.200.peg.2629	T3S	SulP family inorganic anion transporter [*Paracidovorax citrulli*]
fig|80869.200.peg.2637	T3S	NAD(P)/FAD-dependent oxidoreductase [*Paracidovorax citrulli*]
fig|80869.200.peg.2639	T3S	tRNA (guanosine(46)-N7)-methyltransferase TrmB [*Paracidovorax citrulli*]
fig|80869.200.peg.2673	T3S	putative avirulence protein AvrRxo1 [*Paracidovorax citrulli* AAC00-1]
fig|80869.200.peg.2699	T3S	hypothetical protein [*Paracidovorax citrulli*]
fig|80869.200.peg.2706	T3S	ATP-binding protein [*Paracidovorax citrulli*]
fig|80869.200.peg.2716	T3S	YbaN family protein [*Paracidovorax citrulli*]
fig|80869.200.peg.2730	T3S	RDD domain containing protein [*Paracidovorax citrulli* AAC00-1]
fig|80869.200.peg.2749	T3S	TetR/AcrR family transcriptional regulator [*Paracidovorax citrulli*]
fig|80869.200.peg.2774	T3S	2OG-Fe dioxygenase family protein [*Paracidovorax citrulli*]
fig|80869.200.peg.2787	T3S	MULTISPECIES: CysB family HTH-type transcriptional regulator [Comamonadaceae]
fig|80869.200.peg.2880	T3S	ABC transporter permease [*Paracidovorax citrulli*]
fig|80869.200.peg.2952	T3S	hypothetical protein [*Paracidovorax citrulli*]
fig|80869.200.peg.2954	T3S	Bifunctional protein PutA [*Paracidovorax citrulli*]
fig|80869.200.peg.2997	T3S	helix-turn-helix transcriptional regulator [*Paracidovorax citrulli*]
fig|80869.200.peg.3023	T3S	thioredoxin family protein [*Paracidovorax citrulli*]
fig|80869.200.peg.3025	T3S	cytochrome c553-like protein [*Paracidovorax citrulli* AAC00-1]
fig|80869.200.peg.3041	T3S	ThiF family adenylyltransferase [*Paracidovorax citrulli*]
fig|80869.200.peg.3045	T3S	lipid A export permease/ATP-binding protein MsbA [*Paracidovorax citrulli*]
fig|80869.200.peg.3063	T3S	DUF72 domain-containing protein [*Paracidovorax citrulli*]
fig|80869.200.peg.3066	T3S	DUF1624 domain-containing protein [*Paracidovorax citrulli*]
fig|80869.200.peg.3067	T3S	glutamate--tRNA ligase [*Paracidovorax citrulli*]
fig|80869.200.peg.3100	T3S	UDP-3-O-(3-hydroxymyristoyl)glucosamine N-acyltransferase [*Paracidovorax citrulli*]
fig|80869.200.peg.3115	T3S	MlaD family protein [*Paracidovorax citrulli*]
fig|80869.200.peg.3119	T3S	YitT family protein [*Paracidovorax citrulli*]
fig|80869.200.peg.3129	T3S	P1 family peptidase [*Paracidovorax citrulli*]
fig|80869.200.peg.3134	T3S	ATP synthase F1 subunit epsilon [*Paracidovorax citrulli*]
fig|80869.200.peg.317	T3S	16S rRNA (cytosine(967)-C(5))-methyltransferase RsmB [*Paracidovorax citrulli*]
fig|80869.200.peg.3182	T3S	PLxRFG domain-containing protein [*Paracidovorax citrulli*]
fig|80869.200.peg.319	T3S	sensor histidine kinase [*Paracidovorax citrulli*]
fig|80869.200.peg.3210	T3S	hypothetical protein [*Paracidovorax citrulli*]
fig|80869.200.peg.3272	T3S	MHS family citrate/tricarballylate:H+ symporter-like MFS transporter [*Paracidovorax citrulli*]
fig|80869.200.peg.3274	T3S	polyhydroxyalkanoate synthesis repressor PhaR [*Paracidovorax citrulli*]
fig|80869.200.peg.3276	T3S	type 1 glutamine amidotransferase domain-containing protein [*Paracidovorax citrulli*]

Prot	Pred	Description
fig|80869.200.peg.3322	T3S	SDR family NAD(P)-dependent oxidoreductase [*Paracidovorax citrulli*]
fig|80869.200.peg.3352	T3S	heavy metal translocating P-type ATPase [*Paracidovorax citrulli*]
fig|80869.200.peg.3357	T3S	malonate--CoA ligase [*Paracidovorax citrulli*]
fig|80869.200.peg.3374	T3S	glycine betaine/L-proline ABC transporter permease ProW [*Paracidovorax citrulli*]
fig|80869.200.peg.339	T3S	PhaM family polyhydroxyalkanoate granule multifunctional regulatory protein [*Paracidovorax citrulli*]
fig|80869.200.peg.3419	T3S	DUF2069 domain-containing protein [*Paracidovorax citrulli*]
fig|80869.200.peg.3444	T3S	GTPase HflX [*Paracidovorax citrulli*]
fig|80869.200.peg.3459	T3S	protein-L-isoaspartate(D-aspartate) O-methyltransferase [*Paracidovorax citrulli*]
fig|80869.200.peg.3474	T3S	ketopantoate reductase [*Paracidovorax citrulli*]
fig|80869.200.peg.3485	T3S	CaiB/BaiF CoA transferase family protein [*Paracidovorax citrulli*]
fig|80869.200.peg.35	T3S	PepSY domain-containing protein [*Paracidovorax citrulli*]
fig|80869.200.peg.3518	T3S	uncharacterized protein DUF2132 [*Paracidovorax citrulli*]
fig|80869.200.peg.3526	T3S	DNA internalization-related competence protein ComEC/Rec2 [*Paracidovorax citrulli*]
fig|80869.200.peg.3532	T3S	pyridoxal kinase PdxY [*Paracidovorax citrulli*]
fig|80869.200.peg.3558	T3S	hypothetical protein [*Paracidovorax citrulli*]
fig|80869.200.peg.3562	T3S	HDOD domain-containing protein [*Paracidovorax citrulli*]
fig|80869.200.peg.3573	T3S	N-acetylmuramoyl-L-alanine amidase [*Paracidovorax citrulli*]
fig|80869.200.peg.3582	T3S	winged helix-turn-helix transcriptional regulator [*Paracidovorax citrulli*]
fig|80869.200.peg.360	T3S	YiaA/YiaB family inner membrane protein [*Paracidovorax citrulli*]
fig|80869.200.peg.3600	T3S	ABC transporter, permease/ATP-binding protein [*Paracidovorax citrulli*]
fig|80869.200.peg.3606	T3S	MULTISPECIES: NADH-quinone oxidoreductase subunit NuoI [Comamonadaceae]
fig|80869.200.peg.3625	T3S	hypothetical protein [*Paracidovorax citrulli*]
fig|80869.200.peg.3637	T3S	molybdopterin converting factor subunit 1 [*Paracidovorax citrulli*]
fig|80869.200.peg.3664	T3S	nucleotide exchange factor GrpE [*Paracidovorax citrulli*]
fig|80869.200.peg.3677	T3S	phosphoribosylanthranilate isomerase [*Paracidovorax citrulli* AAC00-1]
fig|80869.200.peg.3682	T3S	LON peptidase substrate-binding domain-containing protein [*Paracidovorax citrulli*]
fig|80869.200.peg.3689	T3S	lysine--tRNA ligase [*Paracidovorax citrulli*]
fig|80869.200.peg.3690	T3S	Phytochrome-like protein cph2 [*Paracidovorax citrulli*]
fig|80869.200.peg.3744	T3S	tryptophan 2,3-dioxygenase [*Paracidovorax citrulli*]
fig|80869.200.peg.3753	T3S	neutral zinc metallopeptidase [*Paracidovorax citrulli*]
fig|80869.200.peg.3757	T3S	guanine deaminase [*Paracidovorax citrulli*]
fig|80869.200.peg.1818	T4S	hypothetical protein [*Paracidovorax citrulli*]
fig|80869.200.peg.1928	T4S	major facilitator superfamily MFS_1 [*Paracidovorax citrulli* AAC00-1]
fig|80869.200.peg.3907	T4S	Ig domain protein, group 1 domain protein [*Paracidovorax citrulli* AAC00-1]
fig|80869.200.peg.1734	T4S	acyl-CoA thioesterase [*Paracidovorax citrulli*]
fig|80869.200.peg.2417	T4S	STY0301 family protein [*Paracidovorax citrulli*]
fig|80869.200.peg.2570	T4S	ABC transporter ATP-binding protein [*Paracidovorax citrulli*]
fig|80869.200.peg.2307	T4S	Ku protein [*Paracidovorax citrulli*]
fig|80869.200.peg.1025	T4S	bacterioferritin [*Paracidovorax citrulli*]
fig|80869.200.peg.3207	T4S	terminase small subunit [*Paracidovorax citrulli*]
fig|80869.200.peg.3631	T4S	molybdenum cofactor biosynthesis protein MoaE [*Paracidovorax citrulli*]
fig|80869.200.peg.2781	T4S	hypothetical protein [*Paracidovorax citrulli*]
fig|80869.200.peg.3262	T4S	isoaspartyl peptidase/L-asparaginase family protein [*Paracidovorax citrulli*]

Prot	Pred	Description
fig|80869.200.peg.2536	T4S	DUF2800 domain-containing protein [*Paracidovorax citrulli*]
fig|80869.200.peg.1778	T4S	RNA recognition motif domain-containing protein [*Paracidovorax citrulli*]
fig|80869.200.peg.2127	T4S	hypothetical protein [*Paracidovorax citrulli*]
fig|80869.200.peg.2684	T4S	hypothetical protein Aave_3072 [*Paracidovorax citrulli* AAC00-1]
fig|80869.200.peg.1986	T4S	DUF1795 domain-containing protein [*Paracidovorax citrulli*]
fig|80869.200.peg.2274	T4S	MULTISPECIES: amino-acid N-acetyltransferase [Paracidovorax]
fig|80869.200.peg.247	T4S	DUF924 family protein [*Paracidovorax citrulli*]
fig|80869.200.peg.309	T4S	50S ribosomal protein L11 [*Paracidovorax citrulli*]
fig|80869.200.peg.2304	T4S	hypothetical protein [*Paracidovorax citrulli*]
fig|80869.200.peg.1903	T4S	conserved hypothetical protein [*Paracidovorax citrulli* AAC00-1]
fig|80869.200.peg.4471	T4S	MULTISPECIES: LysR family transcriptional regulator [Paracidovorax]
fig|80869.200.peg.1330	T4S	PP2C family protein-serine/threonine phosphatase [*Paracidovorax citrulli*]
fig|80869.200.peg.4711	T4S	hypothetical protein [*Paracidovorax citrulli*]
fig|80869.200.peg.330	T4S	cryptochrome/photolyase family protein [*Paracidovorax citrulli*]
fig|80869.200.peg.3556	T4S	phosphopyruvate hydratase [*Paracidovorax citrulli*]
fig|80869.200.peg.973	T4S	glutathione-regulated potassium-efflux system protein KefC [*Paracidovorax citrulli*]
fig|80869.200.peg.1386	T4S	hypothetical protein [*Paracidovorax citrulli*]
fig|80869.200.peg.2890	T4S	hypothetical protein [*Paracidovorax citrulli*]
fig|80869.200.peg.3087	T4S	AAA family ATPase [*Paracidovorax citrulli*]
fig|80869.200.peg.545	T4S	DUF3025 domain-containing protein [*Paracidovorax citrulli*]
fig|80869.200.peg.1329	T4S	serine/threonine protein kinase [*Paracidovorax citrulli*]
fig|80869.200.peg.1099	T4S	hypothetical protein [*Paracidovorax citrulli*]
fig|80869.200.peg.1701	T4S	inositol monophosphatase family protein [*Paracidovorax citrulli*]
fig|80869.200.peg.618	T4S	N-acetylmuramoyl-L-alanine amidase [*Paracidovorax citrulli*]
fig|80869.200.peg.4264	T4S	AraC family transcriptional regulator [*Paracidovorax citrulli*]
fig|80869.200.peg.3456	T4S	SMC-Scp complex subunit ScpB [*Paracidovorax citrulli*]
fig|80869.200.peg.2950	T4S	hypothetical protein [*Paracidovorax citrulli*]
fig|80869.200.peg.212	T4S	Fic family protein [*Paracidovorax citrulli*]
fig|80869.200.peg.1117	T4S	4-hydroxy-3-methylbut-2-enyl diphosphate reductase [*Paracidovorax citrulli*]
fig|80869.200.peg.3010	T4S	SsrA-binding protein SmpB [*Paracidovorax citrulli*]
fig|80869.200.peg.751	T4S	hypothetical protein [*Paracidovorax citrulli*]
fig|80869.200.peg.4396	T4S	F0F1 ATP synthase subunit gamma [*Paracidovorax citrulli*]
fig|80869.200.peg.2537	T4S	DUF2815 family protein [*Paracidovorax citrulli*]
fig|80869.200.peg.3429	T4S	hypothetical protein [*Paracidovorax citrulli*]
fig|80869.200.peg.2172	T4S	transglycosylase SLT domain-containing protein [*Paracidovorax citrulli*]
fig|80869.200.peg.4285	T4S	type III secretion system outer membrane ring subunit SctC [*Paracidovorax citrulli*]
fig|80869.200.peg.3188	T4S	hypothetical protein [*Paracidovorax citrulli*]
fig|80869.200.peg.3312	T4S	XopE/AvrPphe family type III secretion system effector [*Paracidovorax citrulli*]
fig|80869.200.peg.909	T4S	5'-nucleotidase [*Paracidovorax citrulli*]
fig|80869.200.peg.4228	T4S	30S ribosomal protein S4 [*Paracidovorax citrulli*]
fig|80869.200.peg.2265	T4S	adenylate kinase [*Paracidovorax citrulli*]
fig|80869.200.peg.2871	T4S	hypothetical protein [*Paracidovorax citrulli*]
fig|80869.200.peg.1550	T4S	hypothetical protein [*Paracidovorax citrulli*]
fig|80869.200.peg.1360	T4S	type IV pilin protein [*Paracidovorax citrulli*]
fig|80869.200.peg.1024	T4S	BON domain-containing protein [*Paracidovorax citrulli*]

Prot	Pred	Description
fig|80869.200.peg.2288	T4S	MetQ/NlpA family ABC transporter substrate-binding protein [*Paracidovorax citrulli*]
fig|80869.200.peg.381	T4S	Bug family tripartite tricarboxylate transporter substrate binding protein [*Paracidovorax citrulli*]
fig|80869.200.peg.3093	T4S	Bug family tripartite tricarboxylate transporter substrate binding protein [*Paracidovorax citrulli*]
fig|80869.200.peg.2282	T4S	sulfate ABC transporter substrate-binding protein [*Paracidovorax citrulli*]
fig|80869.200.peg.1141	T4S	hypothetical protein [*Paracidovorax citrulli*]
fig|80869.200.peg.248	T4S	OmpA family protein [*Paracidovorax citrulli*]
fig|80869.200.peg.3174	T4S	glycoside hydrolase family protein [*Paracidovorax citrulli*]
fig|80869.200.peg.1518	T4S	sigma-54-dependent transcriptional regulator [*Paracidovorax citrulli*]
fig|80869.200.peg.908	T4S	EF-hand domain-containing protein [*Paracidovorax citrulli*]

*P_citrulli*_KACC17005_2

Prot	Pred	Description
fig|80869.197.peg.3403	T3S	Ig domain protein, group 1 domain protein [*Paracidovorax citrulli* AAC00-1]
fig|80869.197.peg.3885	T3S	DUF3577 domain-containing protein [*Paracidovorax citrulli*]
fig|80869.197.peg.690	T3S	major facilitator superfamily MFS_1 [*Paracidovorax citrulli* AAC00-1]
fig|80869.197.peg.805	T3S	hypothetical protein [*Paracidovorax citrulli*]
fig|80869.197.peg.888	T3S	acyl-CoA thioesterase [*Paracidovorax citrulli*]
fig|80869.197.peg.530	T3S	STY0301 family protein [*Paracidovorax citrulli*]
fig|80869.197.peg.1952	T3S	ABC transporter ATP-binding protein [*Paracidovorax citrulli*]
fig|80869.197.peg.416	T3S	Ku protein [*Paracidovorax citrulli*]
fig|80869.197.peg.2644	T3S	terminase small subunit [*Paracidovorax citrulli*]
fig|80869.197.peg.1000	T3S	AI-2E family transporter [*Paracidovorax citrulli*]
fig|80869.197.peg.1002	T3S	phosphoenolpyruvate carboxylase [*Paracidovorax citrulli*]
fig|80869.197.peg.1005	T3S	uroporphyrinogen-III C-methyltransferase [*Paracidovorax citrulli*]
fig|80869.197.peg.1007	T3S	ABC-F family ATP-binding cassette domain-containing protein [*Paracidovorax citrulli*]
fig|80869.197.peg.1020	T3S	hypothetical protein [*Paracidovorax citrulli*]
fig|80869.197.peg.1029	T3S	CDP-6-deoxy-delta-3,4-glucoseen reductase [*Paracidovorax citrulli*]
fig|80869.197.peg.104	T3S	pyridoxamine 5'-phosphate oxidase [*Paracidovorax citrulli*]
fig|80869.197.peg.1059	T3S	argininosuccinate lyase [*Paracidovorax citrulli*]
fig|80869.197.peg.1153	T3S	hypothetical protein [*Paracidovorax citrulli*]
fig|80869.197.peg.1157	T3S	protein of unknown function DUF1653 [*Paracidovorax citrulli* AAC00-1]
fig|80869.197.peg.1172	T3S	isoleucine--tRNA ligase [*Paracidovorax citrulli*]
fig|80869.197.peg.1222	T3S	PilZ domain-containing protein [*Paracidovorax citrulli*]
fig|80869.197.peg.1261	T3S	urease accessory protein [*Paracidovorax citrulli*]
fig|80869.197.peg.1262	T3S	urease accessory protein UreG [*Paracidovorax citrulli*]
fig|80869.197.peg.1300	T3S	SAM-dependent methyltransferase [*Paracidovorax citrulli*]
fig|80869.197.peg.1370	T3S	cardiolipin synthase [*Paracidovorax citrulli*]
fig|80869.197.peg.1385	T3S	GGDEF domain-containing protein [*Paracidovorax citrulli*]
fig|80869.197.peg.1394	T3S	glycerophosphodiester phosphodiesterase [*Paracidovorax citrulli*]
fig|80869.197.peg.1470	T3S	hypothetical protein [*Paracidovorax citrulli*]
fig|80869.197.peg.1471	T3S	4'-phosphopantetheinyl transferase [*Paracidovorax citrulli* AAC00-1]
fig|80869.197.peg.1476	T3S	GNAT family N-acetyltransferase [*Paracidovorax citrulli*]
fig|80869.197.peg.1584	T3S	hypothetical protein [*Paracidovorax citrulli*]

Prot	Pred	Description
fig|80869.197.peg.1596	T3S	YitT family protein [*Paracidovorax citrulli*]
fig|80869.197.peg.1597	T3S	hypothetical protein [*Paracidovorax citrulli*]
fig|80869.197.peg.1609	T3S	hypothetical protein [*Paracidovorax citrulli*]
fig|80869.197.peg.1613	T3S	3-methyl-2-oxobutanoate hydroxymethyltransferase [*Paracidovorax citrulli*]
fig|80869.197.peg.1634	T3S	signal recognition particle-docking protein FtsY [*Paracidovorax citrulli*]
fig|80869.197.peg.1645	T3S	MATE family efflux transporter [*Paracidovorax citrulli*]
fig|80869.197.peg.1714	T3S	type III secretion system chaperone [*Paracidovorax citrulli*]
fig|80869.197.peg.1715	T3S	hypothetical protein [*Paracidovorax citrulli*]
fig|80869.197.peg.1742	T3S	luciferase family protein [*Paracidovorax citrulli* AAC00-1]
fig|80869.197.peg.1759	T3S	hypothetical protein [*Paracidovorax citrulli*]
fig|80869.197.peg.176	T3S	hypothetical protein [*Paracidovorax citrulli*]
fig|80869.197.peg.1773	T3S	ABC transporter permease [*Paracidovorax citrulli*]
fig|80869.197.peg.1774	T3S	ABC transporter permease [*Paracidovorax citrulli*]
fig|80869.197.peg.1786	T3S	(2Fe-2S)-binding protein [*Paracidovorax citrulli*]
fig|80869.197.peg.1787	T3S	molybdenum cofactor cytidylyltransferase [*Paracidovorax citrulli*]
fig|80869.197.peg.1793	T3S	hypothetical protein [*Paracidovorax citrulli*]
fig|80869.197.peg.1794	T3S	NUDIX hydrolase [*Paracidovorax citrulli*]
fig|80869.197.peg.1804	T3S	alpha/beta hydrolase [*Paracidovorax citrulli*]
fig|80869.197.peg.1830	T3S	septal ring lytic transglycosylase RlpA family protein [*Paracidovorax citrulli*]
fig|80869.197.peg.1843	T3S	hypothetical protein [*Paracidovorax citrulli*]
fig|80869.197.peg.1850	T3S	TRAP transporter small permease [*Paracidovorax citrulli*]
fig|80869.197.peg.1918	T3S	glycoside hydrolase family 5 protein [*Paracidovorax citrulli*]
fig|80869.197.peg.1930	T3S	protein of unknown function UPF0061 [*Paracidovorax citrulli* AAC00-1]
fig|80869.197.peg.2011	T3S	BON domain-containing protein [*Paracidovorax citrulli*]
fig|80869.197.peg.2012	T3S	SulP family inorganic anion transporter [*Paracidovorax citrulli*]
fig|80869.197.peg.2019	T3S	NAD(P)/FAD-dependent oxidoreductase [*Paracidovorax citrulli*]
fig|80869.197.peg.2021	T3S	tRNA (guanosine(46)-N7)-methyltransferase TrmB [*Paracidovorax citrulli*]
fig|80869.197.peg.2082	T3S	hypothetical protein [*Paracidovorax citrulli*]
fig|80869.197.peg.2089	T3S	ATP-binding protein [*Paracidovorax citrulli*]
fig|80869.197.peg.2099	T3S	YbaN family protein [*Paracidovorax citrulli*]
fig|80869.197.peg.2114	T3S	RDD domain containing protein [*Paracidovorax citrulli* AAC00-1]
fig|80869.197.peg.2134	T3S	TetR/AcrR family transcriptional regulator [*Paracidovorax citrulli*]
fig|80869.197.peg.2159	T3S	2OG-Fe dioxygenase family protein [*Paracidovorax citrulli*]
fig|80869.197.peg.2172	T3S	MULTISPECIES: CysB family HTH-type transcriptional regulator [Comamonadaceae]
fig|80869.197.peg.218	T3S	DEAD/DEAH box helicase [*Paracidovorax citrulli*]
fig|80869.197.peg.2228	T3S	DUF4230 domain-containing protein [*Paracidovorax citrulli*]
fig|80869.197.peg.2264	T3S	ABC transporter permease [*Paracidovorax citrulli*]
fig|80869.197.peg.230	T3S	hypothetical protein CQB05_01060 [*Paracidovorax citrulli*]
fig|80869.197.peg.2334	T3S	hypothetical protein [*Paracidovorax citrulli*]
fig|80869.197.peg.2336	T3S	L-proline dehydrogenase /delta-1-pyrroline-5-carboxylate dehydrogenase [*Paracidovorax citrulli*]
fig|80869.197.peg.2379	T3S	helix-turn-helix transcriptional regulator [*Paracidovorax citrulli*]
fig|80869.197.peg.2405	T3S	thioredoxin family protein [*Paracidovorax citrulli*]
fig|80869.197.peg.2407	T3S	cytochrome c553-like protein [*Paracidovorax citrulli* AAC00-1]
fig|80869.197.peg.2423	T3S	ThiF family adenylyltransferase [*Paracidovorax citrulli*]
fig|80869.197.peg.2446	T3S	DUF72 domain-containing protein [*Paracidovorax citrulli*]

Prot	Pred	Description
fig|80869.197.peg.2449	T3S	DUF1624 domain-containing protein [*Paracidovorax citrulli*]
fig|80869.197.peg.2450	T3S	glutamate--tRNA ligase [*Paracidovorax citrulli*]
fig|80869.197.peg.2483	T3S	UDP-3-O-(3-hydroxymyristoyl)glucosamine N-acyltransferase [*Paracidovorax citrulli*]
fig|80869.197.peg.2498	T3S	MlaD family protein [*Paracidovorax citrulli*]
fig|80869.197.peg.2502	T3S	YitT family protein [*Paracidovorax citrulli*]
fig|80869.197.peg.2513	T3S	P1 family peptidase [*Paracidovorax citrulli*]
fig|80869.197.peg.2518	T3S	ATP synthase F1 subunit epsilon [*Paracidovorax citrulli*]
fig|80869.197.peg.253	T3S	pseudouridine synthase [*Paracidovorax citrulli*]
fig|80869.197.peg.2533	T3S	structural protein P5 [*Paracidovorax citrulli*]
fig|80869.197.peg.2535	T3S	hypothetical protein [*Paracidovorax citrulli*]
fig|80869.197.peg.257	T3S	translesion DNA synthesis-associated protein ImuA [*Paracidovorax citrulli*]
fig|80869.197.peg.2647	T3S	hypothetical protein [*Paracidovorax citrulli*]
fig|80869.197.peg.2660	T3S	hypothetical protein Aave_1685 [*Paracidovorax citrulli* AAC00-1]
fig|80869.197.peg.268	T3S	GTP cyclohydrolase subunit MoaA [*Paracidovorax citrulli* AAC00-1]
fig|80869.197.peg.271	T3S	gephyrin-like molybdotransferase Glp [*Paracidovorax citrulli*]
fig|80869.197.peg.2718	T3S	hypothetical protein [*Paracidovorax citrulli*]
fig|80869.197.peg.2748	T3S	hypothetical protein Aave_1606 [*Paracidovorax citrulli* AAC00-1]
fig|80869.197.peg.2758	T3S	polyhydroxyalkanoate synthesis repressor PhaR [*Paracidovorax citrulli*]
fig|80869.197.peg.2760	T3S	type 1 glutamine amidotransferase domain-containing protein [*Paracidovorax citrulli*]
fig|80869.197.peg.2807	T3S	SDR family NAD(P)-dependent oxidoreductase [*Paracidovorax citrulli*]
fig|80869.197.peg.2837	T3S	heavy metal translocating P-type ATPase [*Paracidovorax citrulli*]
fig|80869.197.peg.2845	T3S	malonate--CoA ligase [*Paracidovorax citrulli*]
fig|80869.197.peg.2864	T3S	glycine betaine/L-proline ABC transporter permease ProW [*Paracidovorax citrulli*]
fig|80869.197.peg.2907	T3S	farnesyl-diphosphate farnesyltransferase [*Paracidovorax citrulli* AAC00-1]
fig|80869.197.peg.2910	T3S	DUF2069 domain-containing protein [*Paracidovorax citrulli*]
fig|80869.197.peg.2936	T3S	GTPase HflX [*Paracidovorax citrulli*]
fig|80869.197.peg.2952	T3S	protein-L-isoaspartate(D-aspartate) O-methyltransferase [*Paracidovorax citrulli*]
fig|80869.197.peg.2979	T3S	CaiB/BaiF CoA transferase family protein [*Paracidovorax citrulli*]
fig|80869.197.peg.3011	T3S	uncharacterized protein DUF2132 [*Paracidovorax citrulli*]
fig|80869.197.peg.3019	T3S	DNA internalization-related competence protein ComEC/Rec2 [*Paracidovorax citrulli*]
fig|80869.197.peg.3025	T3S	pyridoxal kinase PdxY [*Paracidovorax citrulli*]
fig|80869.197.peg.303	T3S	phosphate ABC transporter permease PstC [*Paracidovorax citrulli*]
fig|80869.197.peg.3051	T3S	hypothetical protein [*Paracidovorax citrulli*]
fig|80869.197.peg.3055	T3S	diguanylate phosphodiesterase [*Paracidovorax citrulli* AAC00-1]
fig|80869.197.peg.3077	T3S	winged helix-turn-helix transcriptional regulator [*Paracidovorax citrulli*]
fig|80869.197.peg.309	T3S	Polyphosphate kinase [*Paracidovorax citrulli* AAC00-1]
fig|80869.197.peg.3095	T3S	ABC transporter transmembrane domain-containing protein [*Paracidovorax citrulli*]
fig|80869.197.peg.3104	T3S	NADH-quinone oxidoreductase subunit NuoI [*Paracidovorax citrulli*]
fig|80869.197.peg.3125	T3S	hypothetical protein [*Paracidovorax citrulli*]
fig|80869.197.peg.3137	T3S	molybdopterin converting factor subunit 1 [*Paracidovorax citrulli*]
fig|80869.197.peg.3165	T3S	phosphoribosylanthranilate isomerase [*Paracidovorax citrulli* AAC00-1]
fig|80869.197.peg.3170	T3S	LON peptidase substrate-binding domain-containing protein [*Paracidovorax citrulli*]
fig|80869.197.peg.3178	T3S	Phytochrome-like protein cph2 [*Paracidovorax citrulli*]

Prot	Pred	Description
fig|80869.197.peg.322	T3S	hypothetical protein [*Paracidovorax citrulli*]
fig|80869.197.peg.323	T3S	D-serine/D-alanine/glycine transporter [*Paracidovorax citrulli*]
fig|80869.197.peg.3232	T3S	tryptophan 2,3-dioxygenase [*Paracidovorax citrulli*]
fig|80869.197.peg.3242	T3S	neutral zinc metallopeptidase [*Paracidovorax citrulli*]
fig|80869.197.peg.3281	T3S	glycerate kinase [*Paracidovorax citrulli*]
fig|80869.197.peg.3297	T3S	NCS1 family nucleobase:cation symporter-1 [*Paracidovorax citrulli*]
fig|80869.197.peg.3299	T3S	GntR family transcriptional regulator [*Paracidovorax citrulli*]
fig|80869.197.peg.3318	T3S	DMT family transporter [*Paracidovorax citrulli*]
fig|80869.197.peg.334	T3S	TonB-dependent receptor family protein [*Paracidovorax citrulli*]
fig|80869.197.peg.3399	T3S	protein of unknown function DUF1415 [*Paracidovorax citrulli* AAC00-1]
fig|80869.197.peg.3409	T3S	penicillin-binding protein 1A [*Paracidovorax citrulli*]
fig|80869.197.peg.3434	T3S	peptidoglycan-binding domain-containing protein [*Paracidovorax citrulli*]
fig|80869.197.peg.3437	T3S	NCS1 family nucleobase:cation symporter-1 [*Paracidovorax citrulli*]
fig|80869.197.peg.3470	T3S	ABC transporter ATP-binding protein [*Paracidovorax citrulli*]
fig|80869.197.peg.3502	T3S	Holliday junction resolvase RuvX [*Paracidovorax citrulli*]
fig|80869.197.peg.3511	T3S	hydroxymethylpyrimidine/phosphomethylpyrimidine kinase [*Paracidovorax citrulli*]
fig|80869.197.peg.3545	T3S	ribonucleoside-diphosphate reductase subunit alpha [*Paracidovorax citrulli*]
fig|80869.197.peg.3558	T3S	3-deoxy-7-phosphoheptulonate synthase [*Paracidovorax citrulli*]
fig|80869.197.peg.3606	T3S	CobW family GTP-binding protein [*Paracidovorax citrulli*]
fig|80869.197.peg.3617	T3S	chromate efflux transporter [*Paracidovorax citrulli*]
fig|80869.197.peg.3624	T3S	M48 family metallopeptidase [*Paracidovorax citrulli*]
fig|80869.197.peg.3639	T3S	DNA-3-methyladenine glycosylase I [*Paracidovorax citrulli*]
fig|80869.197.peg.3719	T3S	FMN-dependent NADH-azoreductase [*Paracidovorax citrulli*]
fig|80869.197.peg.3765	T3S	NarK family nitrate/nitrite MFS transporter [*Paracidovorax citrulli*]
fig|80869.197.peg.3767	T3S	transcriptional regulator, AraC family [*Paracidovorax citrulli* AAC00-1]
fig|80869.197.peg.3780	T3S	FAD-linked oxidase C-terminal domain-containing protein [*Paracidovorax citrulli*]
fig|80869.197.peg.3827	T3S	glycosyltransferase involved in cell wall biosynthesis [*Paracidovorax citrulli*]
fig|80869.197.peg.3840	T3S	uracil-DNA glycosylase [*Paracidovorax citrulli*]
fig|80869.197.peg.3875	T3S	HNH endonuclease [*Paracidovorax citrulli*]
fig|80869.197.peg.3895	T3S	MULTISPECIES: hypothetical protein [Pseudomonadota]
fig|80869.197.peg.3962	T3S	hypothetical protein [*Paracidovorax citrulli*]
fig|80869.197.peg.3968	T3S	hypothetical protein [*Paracidovorax citrulli*]
fig|80869.197.peg.3979	T3S	hypothetical protein [*Paracidovorax citrulli*]
fig|80869.197.peg.3980	T3S	hypothetical protein [*Paracidovorax citrulli*]
fig|80869.197.peg.3984	T3S	hypothetical protein Aave_0457 [*Paracidovorax citrulli* AAC00-1]
fig|80869.197.peg.4020	T3S	hypothetical protein [*Paracidovorax citrulli*]
fig|80869.197.peg.4025	T3S	TspO/MBR family protein [*Paracidovorax citrulli*]
fig|80869.197.peg.4060	T3S	Kelch repeat-containing protein [*Paracidovorax citrulli*]
fig|80869.197.peg.4068	T3S	TPM domain-containing protein [*Paracidovorax citrulli*]
fig|80869.197.peg.4091	T3S	potassium-transporting ATPase subunit KdpB [*Paracidovorax citrulli*]
fig|80869.197.peg.4127	T3S	efflux RND transporter periplasmic adaptor subunit [*Paracidovorax citrulli*]
fig|80869.197.peg.4128	T3S	DHA2 family efflux MFS transporter permease subunit [*Paracidovorax citrulli*]
fig|80869.197.peg.3403	T4S	Ig domain protein, group 1 domain protein [*Paracidovorax citrulli* AAC00-1]

Prot	Pred	Description
fig|80869.197.peg.3885	T4S	DUF3577 domain-containing protein [*Paracidovorax citrulli*]
fig|80869.197.peg.690	T4S	major facilitator superfamily MFS_1 [*Paracidovorax citrulli* AAC00-1]
fig|80869.197.peg.805	T4S	hypothetical protein [*Paracidovorax citrulli*]
fig|80869.197.peg.888	T4S	acyl-CoA thioesterase [*Paracidovorax citrulli*]
fig|80869.197.peg.530	T4S	STY0301 family protein [*Paracidovorax citrulli*]
fig|80869.197.peg.1952	T4S	ABC transporter ATP-binding protein [*Paracidovorax citrulli*]
fig|80869.197.peg.416	T4S	Ku protein [*Paracidovorax citrulli*]
fig|80869.197.peg.2644	T4S	terminase small subunit [*Paracidovorax citrulli*]
fig|80869.197.peg.3131	T4S	molybdenum cofactor biosynthesis protein MoaE [*Paracidovorax citrulli*]
fig|80869.197.peg.1627	T4S	bacterioferritin [*Paracidovorax citrulli*]
fig|80869.197.peg.2693	T4S	isoaspartyl peptidase/L-asparaginase family protein [*Paracidovorax citrulli*]
fig|80869.197.peg.2950	T4S	RluA family pseudouridine synthase [*Paracidovorax citrulli*]
fig|80869.197.peg.1435	T4S	cytochrome b [*Paracidovorax citrulli*]
fig|80869.197.peg.1525	T4S	abortive infection system antitoxin AbiGi family protein [*Paracidovorax citrulli*]
fig|80869.197.peg.844	T4S	RNA recognition motif domain-containing protein [*Paracidovorax citrulli*]
fig|80869.197.peg.229	T4S	hypothetical protein [*Paracidovorax citrulli*]
fig|80869.197.peg.2066	T4S	hypothetical protein Aave_3072 [*Paracidovorax citrulli* AAC00-1]
fig|80869.197.peg.65	T4S	DUF1795 domain-containing protein [*Paracidovorax citrulli*]
fig|80869.197.peg.3965	T4S	type III secretion system outer membrane ring subunit SctC [*Paracidovorax citrulli*]
fig|80869.197.peg.463	T4S	succinyl-diaminopimelate desuccinylase [*Paracidovorax citrulli*]
fig|80869.197.peg.829	T4S	hypothetical protein [*Paracidovorax citrulli*]
fig|80869.197.peg.381	T4S	MULTISPECIES: amino-acid N-acetyltransferase [Paracidovorax]
fig|80869.197.peg.4709	T4S	DUF924 family protein [*Paracidovorax citrulli*]
fig|80869.197.peg.1461	T4S	hypothetical protein [*Paracidovorax citrulli*]
fig|80869.197.peg.4772	T4S	50S ribosomal protein L11 [*Paracidovorax citrulli*]
fig|80869.197.peg.2600	T4S	Gp49 family protein [*Paracidovorax citrulli*]
fig|80869.197.peg.715	T4S	conserved hypothetical protein [*Paracidovorax citrulli* AAC00-1]
fig|80869.197.peg.4710	T4S	OmpA family protein [*Paracidovorax citrulli*]
fig|80869.197.peg.1308	T4S	PP2C family protein-serine/threonine phosphatase [*Paracidovorax citrulli*]
fig|80869.197.peg.1681	T4S	glutathione-regulated potassium-efflux system protein KefC [*Paracidovorax citrulli*]
fig|80869.197.peg.2613	T4S	PLxRFG domain-containing protein [*Paracidovorax citrulli*]
fig|80869.197.peg.4387	T4S	hypothetical protein [*Paracidovorax citrulli*]
fig|80869.197.peg.4791	T4S	cryptochrome/photolyase family protein [*Paracidovorax citrulli*]
fig|80869.197.peg.3049	T4S	phosphopyruvate hydratase [*Paracidovorax citrulli*]
fig|80869.197.peg.1251	T4S	hypothetical protein [*Paracidovorax citrulli*]
fig|80869.197.peg.2274	T4S	hypothetical protein [*Paracidovorax citrulli*]
fig|80869.197.peg.2470	T4S	AAA family ATPase [*Paracidovorax citrulli*]
fig|80869.197.peg.1309	T4S	serine/threonine protein kinase [*Paracidovorax citrulli*]
fig|80869.197.peg.923	T4S	inositol monophosphatase family protein [*Paracidovorax citrulli*]
fig|80869.197.peg.5081	T4S	N-acetylmuramoyl-L-alanine amidase [*Paracidovorax citrulli*]
fig|80869.197.peg.3835	T4S	AraC family transcriptional regulator [*Paracidovorax citrulli*]
fig|80869.197.peg.2948	T4S	SMC-Scp complex subunit ScpB [*Paracidovorax citrulli*]
fig|80869.197.peg.2332	T4S	hypothetical protein [*Paracidovorax citrulli*]
fig|80869.197.peg.4673	T4S	Fic family protein [*Paracidovorax citrulli*]

Prot	Pred	Description
fig|80869.197.peg.1532	T4S	4-hydroxy-3-methylbut-2-enyl diphosphate reductase [*Paracidovorax citrulli*]
fig|80869.197.peg.2391	T4S	SsrA-binding protein SmpB [*Paracidovorax citrulli*]
fig|80869.197.peg.1901	T4S	hypothetical protein [*Paracidovorax citrulli*]
fig|80869.197.peg.4076	T4S	F0F1 ATP synthase subunit gamma [*Paracidovorax citrulli*]
fig|80869.197.peg.1079	T4S	hypothetical protein [*Paracidovorax citrulli*]
fig|80869.197.peg.2921	T4S	hypothetical protein [*Paracidovorax citrulli*]
fig|80869.197.peg.277	T4S	transglycosylase SLT domain-containing protein [*Paracidovorax citrulli*]
fig|80869.197.peg.5217	T4S	hypothetical protein [*Paracidovorax citrulli*]
fig|80869.197.peg.3251	T4S	penicillin acylase family protein [*Paracidovorax citrulli*]
fig|80869.197.peg.2625	T4S	hypothetical protein [*Paracidovorax citrulli*]
fig|80869.197.peg.1744	T4S	5'-nucleotidase [*Paracidovorax citrulli*]
fig|80869.197.peg.599	T4S	hypothetical protein [*Paracidovorax citrulli*]
fig|80869.197.peg.3801	T4S	30S ribosomal protein S4 [*Paracidovorax citrulli*]
fig|80869.197.peg.372	T4S	adenylate kinase [*Paracidovorax citrulli*]
fig|80869.197.peg.2255	T4S	hypothetical protein [*Paracidovorax citrulli*]
fig|80869.197.peg.1277	T4S	type IV pilin protein [*Paracidovorax citrulli*]
fig|80869.197.peg.4987	T4S	hypothetical protein [*Paracidovorax citrulli*]
fig|80869.197.peg.1628	T4S	BON domain-containing protein [*Paracidovorax citrulli*]
fig|80869.197.peg.395	T4S	MetQ/NlpA family ABC transporter substrate-binding protein [*Paracidovorax citrulli*]
fig|80869.197.peg.4843	T4S	Bug family tripartite tricarboxylate transporter substrate binding protein [*Paracidovorax citrulli*]
fig|80869.197.peg.194	T4S	hypothetical protein [*Paracidovorax citrulli*]
fig|80869.197.peg.389	T4S	sulfate ABC transporter substrate-binding protein [*Paracidovorax citrulli*]
fig|80869.197.peg.1507	T4S	hypothetical protein [*Paracidovorax citrulli*]
fig|80869.197.peg.2604	T4S	glycoside hydrolase family protein [*Paracidovorax citrulli*]
fig|80869.197.peg.4149	T4S	DNA-binding transcriptional LysR family regulator [*Paracidovorax citrulli*]
fig|80869.197.peg.1114	T4S	sigma-54-dependent transcriptional regulator [*Paracidovorax citrulli*]
fig|80869.197.peg.1745	T4S	EF-hand domain-containing protein [*Paracidovorax citrulli*]

*P_citrulli*_KACC17913

Prot	Pred	Description
fig|80869.196.peg.1047	T3S	Ig domain protein, group 1 domain protein [*Paracidovorax citrulli* AAC00-1]
fig|80869.196.peg.3223	T3S	major facilitator superfamily MFS_1 [*Paracidovorax citrulli* AAC00-1]
fig|80869.196.peg.3335	T3S	hypothetical protein [*Paracidovorax citrulli*]
fig|80869.196.peg.568	T3S	DUF3577 domain-containing protein [*Paracidovorax citrulli*]
fig|80869.196.peg.3420	T3S	acyl-CoA thioesterase [*Paracidovorax citrulli*]
fig|80869.196.peg.3063	T3S	STY0301 family protein [*Paracidovorax citrulli*]
fig|80869.196.peg.2476	T3S	ABC transporter ATP-binding protein [*Paracidovorax citrulli*]
fig|80869.196.peg.2950	T3S	Ku protein [*Paracidovorax citrulli*]
fig|80869.196.peg.1796	T3S	terminase small subunit [*Paracidovorax citrulli*]
fig|80869.196.peg.1014	T3S	NCS1 family nucleobase:cation symporter-1 [*Paracidovorax citrulli*]
fig|80869.196.peg.1017	T3S	peptidoglycan-binding domain-containing protein [*Paracidovorax citrulli*]
fig|80869.196.peg.102	T3S	5-demethoxyubiquinol-8 5-hydroxylase UbiM [*Paracidovorax citrulli*]
fig|80869.196.peg.1041	T3S	penicillin-binding protein 1A [*Paracidovorax citrulli*]

Prot	Pred	Description
fig|80869.196.peg.1051	T3S	protein of unknown function DUF1415 [*Paracidovorax citrulli* AAC00-1]
fig|80869.196.peg.1087	T3S	PelD GGDEF domain-containing protein [*Paracidovorax citrulli*]
fig|80869.196.peg.1130	T3S	DMT family transporter [*Paracidovorax citrulli*]
fig|80869.196.peg.1149	T3S	GntR family transcriptional regulator [*Paracidovorax citrulli*]
fig|80869.196.peg.1151	T3S	NCS1 family nucleobase:cation symporter-1 [*Paracidovorax citrulli*]
fig|80869.196.peg.1167	T3S	glycerate kinase [*Paracidovorax citrulli*]
fig|80869.196.peg.1205	T3S	neutral zinc metallopeptidase [*Paracidovorax citrulli*]
fig|80869.196.peg.1214	T3S	tryptophan 2,3-dioxygenase [*Paracidovorax citrulli*]
fig|80869.196.peg.1265	T3S	Phytochrome-like protein cph2 [*Paracidovorax citrulli*]
fig|80869.196.peg.1266	T3S	lysine--tRNA ligase [*Paracidovorax citrulli*]
fig|80869.196.peg.1273	T3S	LON peptidase substrate-binding domain-containing protein [*Paracidovorax citrulli*]
fig|80869.196.peg.1278	T3S	phosphoribosylanthranilate isomerase [*Paracidovorax citrulli* AAC00-1]
fig|80869.196.peg.1291	T3S	nucleotide exchange factor GrpE [*Paracidovorax citrulli*]
fig|80869.196.peg.1306	T3S	molybdopterin converting factor subunit 1 [*Paracidovorax citrulli*]
fig|80869.196.peg.1319	T3S	hypothetical protein [*Paracidovorax citrulli*]
fig|80869.196.peg.1338	T3S	MULTISPECIES: NADH-quinone oxidoreductase subunit NuoI [Comamonadaceae]
fig|80869.196.peg.1346	T3S	ABC transporter transmembrane domain-containing protein [*Paracidovorax citrulli*]
fig|80869.196.peg.1364	T3S	winged helix-turn-helix transcriptional regulator [*Paracidovorax citrulli*]
fig|80869.196.peg.1385	T3S	diguanylate phosphodiesterase [*Paracidovorax citrulli* AAC00-1]
fig|80869.196.peg.1389	T3S	hypothetical protein [*Paracidovorax citrulli*]
fig|80869.196.peg.1415	T3S	pyridoxal kinase PdxY [*Paracidovorax citrulli*]
fig|80869.196.peg.1421	T3S	DNA internalization-related competence protein ComEC/Rec2 [*Paracidovorax citrulli*]
fig|80869.196.peg.1429	T3S	uncharacterized protein DUF2132 [*Paracidovorax citrulli*]
fig|80869.196.peg.1462	T3S	CaiB/BaiF CoA transferase family protein [*Paracidovorax citrulli*]
fig|80869.196.peg.1490	T3S	protein-L-isoaspartate(D-aspartate) O-methyltransferase [*Paracidovorax citrulli*]
fig|80869.196.peg.1505	T3S	GTPase HflX [*Paracidovorax citrulli*]
fig|80869.196.peg.1530	T3S	DUF2069 domain-containing protein [*Paracidovorax citrulli*]
fig|80869.196.peg.1533	T3S	farnesyl-diphosphate farnesyltransferase [*Paracidovorax citrulli* AAC00-1]
fig|80869.196.peg.156	T3S	aromatic ring-hydroxylating dioxygenase subunit alpha [*Paracidovorax citrulli*]
fig|80869.196.peg.1574	T3S	glycine betaine/L-proline ABC transporter permease ProW [*Paracidovorax citrulli*]
fig|80869.196.peg.1594	T3S	malonate--CoA ligase [*Paracidovorax citrulli*]
fig|80869.196.peg.1602	T3S	heavy metal translocating P-type ATPase [*Paracidovorax citrulli*]
fig|80869.196.peg.1632	T3S	SDR family NAD(P)-dependent oxidoreductase [*Paracidovorax citrulli*]
fig|80869.196.peg.168	T3S	Cupin 2, conserved barrel domain protein [*Paracidovorax citrulli* AAC00-1]
fig|80869.196.peg.1680	T3S	type 1 glutamine amidotransferase domain-containing protein [*Paracidovorax citrulli*]
fig|80869.196.peg.1682	T3S	polyhydroxyalkanoate synthesis repressor PhaR [*Paracidovorax citrulli*]
fig|80869.196.peg.1692	T3S	hypothetical protein Aave_1606 [*Paracidovorax citrulli* AAC00-1]
fig|80869.196.peg.1722	T3S	hypothetical protein [*Paracidovorax citrulli*]
fig|80869.196.peg.1793	T3S	hypothetical protein [*Paracidovorax citrulli*]
fig|80869.196.peg.1900	T3S	hypothetical protein [*Paracidovorax citrulli*]

Prot	Pred	Description
fig|80869.196.peg.1903	T3S	structural protein P5 [*Paracidovorax citrulli*]
fig|80869.196.peg.1918	T3S	ATP synthase F1 subunit epsilon [*Paracidovorax citrulli*]
fig|80869.196.peg.1923	T3S	P1 family peptidase [*Paracidovorax citrulli*]
fig|80869.196.peg.1934	T3S	YitT family protein [*Paracidovorax citrulli*]
fig|80869.196.peg.1938	T3S	MlaD family protein [*Paracidovorax citrulli*]
fig|80869.196.peg.1953	T3S	UDP-3-O-(3-hydroxymyristoyl)glucosamine N-acyltransferase [*Paracidovorax citrulli*]
fig|80869.196.peg.1986	T3S	glutamate--tRNA ligase [*Paracidovorax citrulli*]
fig|80869.196.peg.1987	T3S	DUF1624 domain-containing protein [*Paracidovorax citrulli*]
fig|80869.196.peg.1990	T3S	DUF72 domain-containing protein [*Paracidovorax citrulli*]
fig|80869.196.peg.2008	T3S	lipid A export permease/ATP-binding protein MsbA [*Paracidovorax citrulli*]
fig|80869.196.peg.2012	T3S	ThiF family adenylyltransferase [*Paracidovorax citrulli*]
fig|80869.196.peg.2028	T3S	cytochrome c553-like protein [*Paracidovorax citrulli* AAC00-1]
fig|80869.196.peg.2030	T3S	thioredoxin family protein [*Paracidovorax citrulli*]
fig|80869.196.peg.2038	T3S	hypothetical protein [*Paracidovorax citrulli*]
fig|80869.196.peg.2055	T3S	helix-turn-helix transcriptional regulator [*Paracidovorax citrulli*]
fig|80869.196.peg.2099	T3S	L-proline dehydrogenase /delta-1-pyrroline-5-carboxylate dehydrogenase [*Paracidovorax citrulli*]
fig|80869.196.peg.2101	T3S	hypothetical protein [*Paracidovorax citrulli*]
fig|80869.196.peg.2173	T3S	ABC transporter permease [*Paracidovorax citrulli*]
fig|80869.196.peg.220	T3S	GNAT family N-acetyltransferase [Paracidovorax avenae]
fig|80869.196.peg.2208	T3S	DUF4230 domain-containing protein [*Paracidovorax citrulli*]
fig|80869.196.peg.2262	T3S	MULTISPECIES: CysB family HTH-type transcriptional regulator [Comamonadaceae]
fig|80869.196.peg.2273	T3S	2OG-Fe dioxygenase family protein [*Paracidovorax citrulli*]
fig|80869.196.peg.2296	T3S	TetR/AcrR family transcriptional regulator [*Paracidovorax citrulli*]
fig|80869.196.peg.2315	T3S	RDD domain containing protein [*Paracidovorax citrulli* AAC00-1]
fig|80869.196.peg.2329	T3S	YbaN family protein [*Paracidovorax citrulli*]
fig|80869.196.peg.2339	T3S	ATP-binding protein [*Paracidovorax citrulli*]
fig|80869.196.peg.2346	T3S	hypothetical protein [*Paracidovorax citrulli*]
fig|80869.196.peg.2406	T3S	tRNA (guanosine(46)-N7)-methyltransferase TrmB [*Paracidovorax citrulli*]
fig|80869.196.peg.2408	T3S	NAD(P)/FAD-dependent oxidoreductase [*Paracidovorax citrulli*]
fig|80869.196.peg.241	T3S	ProQ/FINO family protein [*Paracidovorax citrulli*]
fig|80869.196.peg.2416	T3S	SulP family inorganic anion transporter [*Paracidovorax citrulli*]
fig|80869.196.peg.2417	T3S	BON domain-containing protein [*Paracidovorax citrulli*]
fig|80869.196.peg.2496	T3S	protein of unknown function UPF0061 [*Paracidovorax citrulli* AAC00-1]
fig|80869.196.peg.2508	T3S	glycoside hydrolase family 5 protein [*Paracidovorax citrulli*]
fig|80869.196.peg.2602	T3S	XopAP family type III secretion system effector [*Paracidovorax citrulli*]
fig|80869.196.peg.2613	T3S	EVE domain-containing protein [*Paracidovorax citrulli*]
fig|80869.196.peg.2632	T3S	TetR/AcrR family transcriptional regulator [*Paracidovorax citrulli*]
fig|80869.196.peg.2634	T3S	transcriptional repressor [*Paracidovorax citrulli*]
fig|80869.196.peg.2645	T3S	pyridoxamine 5'-phosphate oxidase [*Paracidovorax citrulli*]
fig|80869.196.peg.2718	T3S	hypothetical protein [*Paracidovorax citrulli*]
fig|80869.196.peg.273	T3S	AmpG family muropeptide MFS transporter [*Paracidovorax citrulli*]
fig|80869.196.peg.2759	T3S	DEAD/DEAH box helicase [*Paracidovorax citrulli*]
fig|80869.196.peg.276	T3S	DME family drug/metabolite transporter [*Paracidovorax citrulli*]
fig|80869.196.peg.2769	T3S	hypothetical protein CQB05_01060 [*Paracidovorax citrulli*]

Prot	Pred	Description
fig|80869.196.peg.278	T3S	SLC13 family permease [*Paracidovorax citrulli*]
fig|80869.196.peg.2791	T3S	pseudouridine synthase [*Paracidovorax citrulli*]
fig|80869.196.peg.2795	T3S	translesion DNA synthesis-associated protein ImuA [*Paracidovorax citrulli*]
fig|80869.196.peg.28	T3S	ATP-binding cassette domain-containing protein [*Paracidovorax citrulli*]
fig|80869.196.peg.2806	T3S	GTP cyclohydrolase subunit MoaA [*Paracidovorax citrulli* AAC00-1]
fig|80869.196.peg.2809	T3S	gephyrin-like molybdotransferase Glp [*Paracidovorax citrulli*]
fig|80869.196.peg.2838	T3S	phosphate ABC transporter permease PstC [*Paracidovorax citrulli*]
fig|80869.196.peg.2843	T3S	Polyphosphate kinase [*Paracidovorax citrulli* AAC00-1]
fig|80869.196.peg.2856	T3S	hypothetical protein [*Paracidovorax citrulli*]
fig|80869.196.peg.2857	T3S	D-serine/D-alanine/glycine transporter [*Paracidovorax citrulli*]
fig|80869.196.peg.2868	T3S	TonB-dependent receptor family protein [*Paracidovorax citrulli*]
fig|80869.196.peg.2962	T3S	GCN5-related N-acetyltransferase [*Paracidovorax citrulli* AAC00-1]
fig|80869.196.peg.297	T3S	amino acid ABC transporter ATP-binding protein [*Paracidovorax citrulli*]
fig|80869.196.peg.2989	T3S	EAL domain-containing protein [*Paracidovorax citrulli*]
fig|80869.196.peg.3023	T3S	PAS domain-containing sensor histidine kinase [*Paracidovorax citrulli*]
fig|80869.196.peg.3072	T3S	KGG domain-containing protein [*Paracidovorax citrulli*]
fig|80869.196.peg.3199	T3S	DODA-type extradiol aromatic ring-opening family dioxygenase [*Paracidovorax citrulli*]
fig|80869.196.peg.3204	T3S	glutamine--tRNA ligase/YqeY domain fusion protein [*Paracidovorax citrulli*]
fig|80869.196.peg.3216	T3S	esterase/lipase/thioesterase family protein [*Paracidovorax citrulli* AAC00-1]
fig|80869.196.peg.3219	T3S	Murein DD-endopeptidase MepM [*Paracidovorax citrulli*]
fig|80869.196.peg.322	T3S	DHA2 family efflux MFS transporter permease subunit [*Paracidovorax citrulli*]
fig|80869.196.peg.3229	T3S	DHH family phosphoesterase [*Paracidovorax citrulli*]
fig|80869.196.peg.323	T3S	efflux RND transporter periplasmic adaptor subunit [*Paracidovorax citrulli*]
fig|80869.196.peg.3233	T3S	redox-sensitive transcriptional activator SoxR [*Paracidovorax citrulli*]
fig|80869.196.peg.3235	T3S	MFS transporter [*Paracidovorax citrulli*]
fig|80869.196.peg.3242	T3S	GAF domain-containing sensor histidine kinase [*Paracidovorax citrulli*]
fig|80869.196.peg.3250	T3S	SMP-30/gluconolactonase/LRE family protein [*Paracidovorax citrulli*]
fig|80869.196.peg.3251	T3S	SDR family NAD(P)-dependent oxidoreductase [*Paracidovorax citrulli*]
fig|80869.196.peg.3257	T3S	multiple monosaccharide ABC transporter permease [*Paracidovorax citrulli*]
fig|80869.196.peg.3291	T3S	cytochrome o ubiquinol oxidase subunit IV [*Paracidovorax citrulli*]
fig|80869.196.peg.3306	T3S	transcriptional regulator, GntR family [*Paracidovorax citrulli* AAC00-1]
fig|80869.196.peg.3320	T3S	NAD-dependent succinate-semialdehyde dehydrogenase [*Paracidovorax citrulli*]
fig|80869.196.peg.3337	T3S	hypothetical protein [*Paracidovorax citrulli*]
fig|80869.196.peg.3366	T3S	hypothetical protein Aave_2148 [*Paracidovorax citrulli* AAC00-1]
fig|80869.196.peg.3387	T3S	DUF2169 domain-containing protein [*Paracidovorax citrulli*]
fig|80869.196.peg.3392	T3S	excinuclease ABC subunit UvrA [*Paracidovorax citrulli*]
fig|80869.196.peg.3400	T3S	carboxyl transferase domain-containing protein [*Paracidovorax citrulli*]
fig|80869.196.peg.3418	T3S	uroporphyrinogen-III C-methyltransferase [*Paracidovorax citrulli*]
fig|80869.196.peg.3441	T3S	succinate-semialdehyde dehydrogenase/glutarate-semialdehyde dehydrogenase [*Paracidovorax citrulli*]
fig|80869.196.peg.3484	T3S	efflux transporter outer membrane subunit [*Paracidovorax citrulli*]

Prot	Pred	Description
fig|80869.196.peg.3495	T3S	TOBE domain-containing protein [*Paracidovorax citrulli*]
fig|80869.196.peg.3500	T3S	BCCT family transporter [*Paracidovorax citrulli*]
fig|80869.196.peg.3503	T3S	alpha-1,4-glucan--maltose-1-phosphate maltosyltransferase [*Paracidovorax citrulli*]
fig|80869.196.peg.3514	T3S	exodeoxyribonuclease VII small subunit [*Paracidovorax citrulli*]
fig|80869.196.peg.3519	T3S	SfnB family sulfur acquisition oxidoreductase [*Paracidovorax citrulli*]
fig|80869.196.peg.3520	T3S	SfnB family sulfur acquisition oxidoreductase [*Paracidovorax citrulli*]
fig|80869.196.peg.3526	T3S	AI-2E family transporter [*Paracidovorax citrulli*]
fig|80869.196.peg.3529	T3S	phosphoenolpyruvate carboxylase [*Paracidovorax citrulli*]
fig|80869.196.peg.3530	T3S	hydroxymethylbilane synthase [*Paracidovorax citrulli*]
fig|80869.196.peg.3532	T3S	uroporphyrinogen-III C-methyltransferase [*Paracidovorax citrulli*]
fig|80869.196.peg.3534	T3S	ABC-F family ATP-binding cassette domain-containing protein [*Paracidovorax citrulli*]
fig|80869.196.peg.3547	T3S	hypothetical protein [*Paracidovorax citrulli*]
fig|80869.196.peg.3556	T3S	CDP-6-deoxy-delta-3,4-glucoseen reductase [*Paracidovorax citrulli*]
fig|80869.196.peg.3586	T3S	argininosuccinate lyase [*Paracidovorax citrulli*]
fig|80869.196.peg.359	T3S	potassium-transporting ATPase subunit KdpB [*Paracidovorax citrulli*]
fig|80869.196.peg.3676	T3S	hypothetical protein [*Paracidovorax citrulli*]
fig|80869.196.peg.3680	T3S	protein of unknown function DUF1653 [*Paracidovorax citrulli* AAC00-1]
fig|80869.196.peg.3695	T3S	isoleucine--tRNA ligase [*Paracidovorax citrulli*]
fig|80869.196.peg.3745	T3S	PilZ domain-containing protein [*Paracidovorax citrulli*]
fig|80869.196.peg.3784	T3S	urease accessory protein [*Paracidovorax citrulli*]
fig|80869.196.peg.3785	T3S	urease accessory protein UreG [*Paracidovorax citrulli*]
fig|80869.196.peg.382	T3S	TPM domain-containing protein [*Paracidovorax citrulli*]
fig|80869.196.peg.3824	T3S	SAM-dependent methyltransferase [*Paracidovorax citrulli*]
fig|80869.196.peg.1047	T4S	Ig domain protein, group 1 domain protein [*Paracidovorax citrulli* AAC00-1]
fig|80869.196.peg.3223	T4S	major facilitator superfamily MFS_1 [*Paracidovorax citrulli* AAC00-1]
fig|80869.196.peg.3335	T4S	hypothetical protein [*Paracidovorax citrulli*]
fig|80869.196.peg.568	T4S	DUF3577 domain-containing protein [*Paracidovorax citrulli*]
fig|80869.196.peg.3420	T4S	acyl-CoA thioesterase [*Paracidovorax citrulli*]
fig|80869.196.peg.3063	T4S	STY0301 family protein [*Paracidovorax citrulli*]
fig|80869.196.peg.2476	T4S	ABC transporter ATP-binding protein [*Paracidovorax citrulli*]
fig|80869.196.peg.2950	T4S	Ku protein [*Paracidovorax citrulli*]
fig|80869.196.peg.1796	T4S	terminase small subunit [*Paracidovorax citrulli*]
fig|80869.196.peg.1313	T4S	molybdenum cofactor biosynthesis protein MoaE [*Paracidovorax citrulli*]
fig|80869.196.peg.4147	T4S	ferritin-like domain-containing protein [*Paracidovorax citrulli*]
fig|80869.196.peg.1747	T4S	isoaspartyl peptidase/L-asparaginase family protein [*Paracidovorax citrulli*]
fig|80869.196.peg.4046	T4S	abortive infection system antitoxin AbiGi family protein [*Paracidovorax citrulli*]
fig|80869.196.peg.3374	T4S	RNA recognition motif domain-containing protein [*Paracidovorax citrulli*]
fig|80869.196.peg.2768	T4S	hypothetical protein [*Paracidovorax citrulli*]
fig|80869.196.peg.2362	T4S	hypothetical protein Aave_3072 [*Paracidovorax citrulli* AAC00-1]
fig|80869.196.peg.2607	T4S	DUF1795 domain-containing protein [*Paracidovorax citrulli*]
fig|80869.196.peg.486	T4S	type III secretion system outer membrane ring subunit SctC [*Paracidovorax citrulli*]
fig|80869.196.peg.3359	T4S	hypothetical protein [*Paracidovorax citrulli*]

Prot	Pred	Description
fig|80869.196.peg.2915	T4S	MULTISPECIES: amino-acid N-acetyltransferase [Paracidovorax]
fig|80869.196.peg.4923	T4S	DUF924 family protein [*Paracidovorax citrulli*]
fig|80869.196.peg.3982	T4S	hypothetical protein [*Paracidovorax citrulli*]
fig|80869.196.peg.4861	T4S	50S ribosomal protein L11 [*Paracidovorax citrulli*]
fig|80869.196.peg.2947	T4S	hypothetical protein [*Paracidovorax citrulli*]
fig|80869.196.peg.3249	T4S	conserved hypothetical protein [*Paracidovorax citrulli* AAC00-1]
fig|80869.196.peg.3831	T4S	PP2C family protein-serine/threonine phosphatase [*Paracidovorax citrulli*]
fig|80869.196.peg.71	T4S	hypothetical protein [*Paracidovorax citrulli*]
fig|80869.196.peg.4842	T4S	cryptochrome/photolyase family protein [*Paracidovorax citrulli*]
fig|80869.196.peg.1391	T4S	phosphopyruvate hydratase [*Paracidovorax citrulli*]
fig|80869.196.peg.3774	T4S	hypothetical protein [*Paracidovorax citrulli*]
fig|80869.196.peg.2163	T4S	hypothetical protein [*Paracidovorax citrulli*]
fig|80869.196.peg.1966	T4S	AAA family ATPase [*Paracidovorax citrulli*]
fig|80869.196.peg.4628	T4S	DUF3025 domain-containing protein [*Paracidovorax citrulli*]
fig|80869.196.peg.3832	T4S	serine/threonine protein kinase [*Paracidovorax citrulli*]
fig|80869.196.peg.3453	T4S	inositol monophosphatase family protein [*Paracidovorax citrulli*]
fig|80869.196.peg.4555	T4S	N-acetylmuramoyl-L-alanine amidase [*Paracidovorax citrulli*]
fig|80869.196.peg.619	T4S	AraC family transcriptional regulator [*Paracidovorax citrulli*]
fig|80869.196.peg.1493	T4S	SMC-Scp complex subunit ScpB [*Paracidovorax citrulli*]
fig|80869.196.peg.2103	T4S	hypothetical protein [*Paracidovorax citrulli*]
fig|80869.196.peg.4959	T4S	Fic family protein [*Paracidovorax citrulli*]
fig|80869.196.peg.4053	T4S	4-hydroxy-3-methylbut-2-enyl diphosphate reductase [*Paracidovorax citrulli*]
fig|80869.196.peg.2043	T4S	SsrA-binding protein SmpB [*Paracidovorax citrulli*]
fig|80869.196.peg.2525	T4S	hypothetical protein [*Paracidovorax citrulli*]
fig|80869.196.peg.374	T4S	F0F1 ATP synthase subunit gamma [*Paracidovorax citrulli*]
fig|80869.196.peg.3606	T4S	hypothetical protein [*Paracidovorax citrulli*]
fig|80869.196.peg.1520	T4S	hypothetical protein [*Paracidovorax citrulli*]
fig|80869.196.peg.2815	T4S	transglycosylase SLT domain-containing protein [*Paracidovorax citrulli*]
fig|80869.196.peg.4419	T4S	hypothetical protein [*Paracidovorax citrulli*]
fig|80869.196.peg.1814	T4S	hypothetical protein [*Paracidovorax citrulli*]
fig|80869.196.peg.4262	T4S	5'-nucleotidase [*Paracidovorax citrulli*]
fig|80869.196.peg.3131	T4S	hypothetical protein [*Paracidovorax citrulli*]
fig|80869.196.peg.654	T4S	30S ribosomal protein S4 [*Paracidovorax citrulli*]
fig|80869.196.peg.2906	T4S	adenylate kinase [*Paracidovorax citrulli*]
fig|80869.196.peg.2182	T4S	hypothetical protein [*Paracidovorax citrulli*]
fig|80869.196.peg.3801	T4S	type IV pilin protein [*Paracidovorax citrulli*]
fig|80869.196.peg.4648	T4S	hypothetical protein [*Paracidovorax citrulli*]
fig|80869.196.peg.4148	T4S	BON domain-containing protein [*Paracidovorax citrulli*]
fig|80869.196.peg.2929	T4S	MetQ/NlpA family ABC transporter substrate-binding protein [*Paracidovorax citrulli*]
fig|80869.196.peg.4790	T4S	Bug family tripartite tricarboxylate transporter substrate binding protein [*Paracidovorax citrulli*]
fig|80869.196.peg.2734	T4S	hypothetical protein [*Paracidovorax citrulli*]
fig|80869.196.peg.2923	T4S	sulfate ABC transporter substrate-binding protein [*Paracidovorax citrulli*]
fig|80869.196.peg.4028	T4S	hypothetical protein [*Paracidovorax citrulli*]
fig|80869.196.peg.4922	T4S	OmpA family protein [*Paracidovorax citrulli*]

Prot	Pred	Description
fig|80869.196.peg.1832	T4S	glycoside hydrolase family protein [*Paracidovorax citrulli*]
fig|80869.196.peg.301	T4S	DNA-binding transcriptional LysR family regulator [*Paracidovorax citrulli*]
fig|80869.196.peg.3638	T4S	sigma-54-dependent transcriptional regulator [*Paracidovorax citrulli*]
fig|80869.196.peg.4263	T4S	EF-hand domain-containing protein [*Paracidovorax citrulli*]
